# Supplementary material for: Uncoupling of dynamin polymerization and GTPase activity revealed by the conformation-specific nanobody dynab
Source: eLife. 2017 Oct 12;6:e25197. doi: 10.7554/eLife.25197 (PMC5658065; doi:10.7554/eLife.25197)

**Figure 1-Source Data 1 panel D**

| dyn1 | dyn1+dynab | dyn2 | dyn2+dynab |
| --- | --- | --- | --- |
| 35.16142 | 20.42391 | 29.64327 | 21.43333 |
| 30.24892 | 36.17084 | 32.13317 | 25.471 |
| 16.79 | 25.87477 | 29.17221 | 19.48179 |
| 33.54635 | 35.29601 | 30.78728 | 25.67289 |
| 30.98916 | 29.2395 | 18.80884 | 27.08607 |

**Malachite Green Assay** GTPase activity expressed in µM Pi/min/µM dyn

**
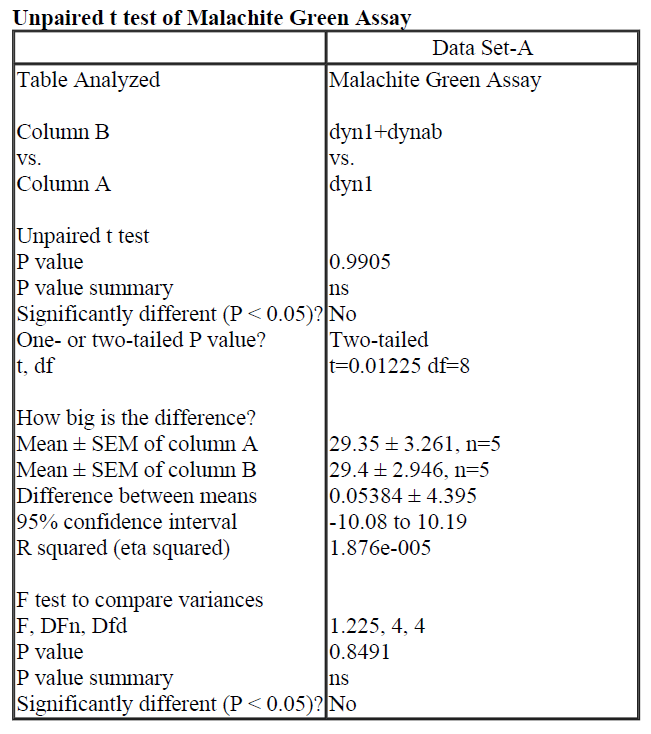
Statistical report**:


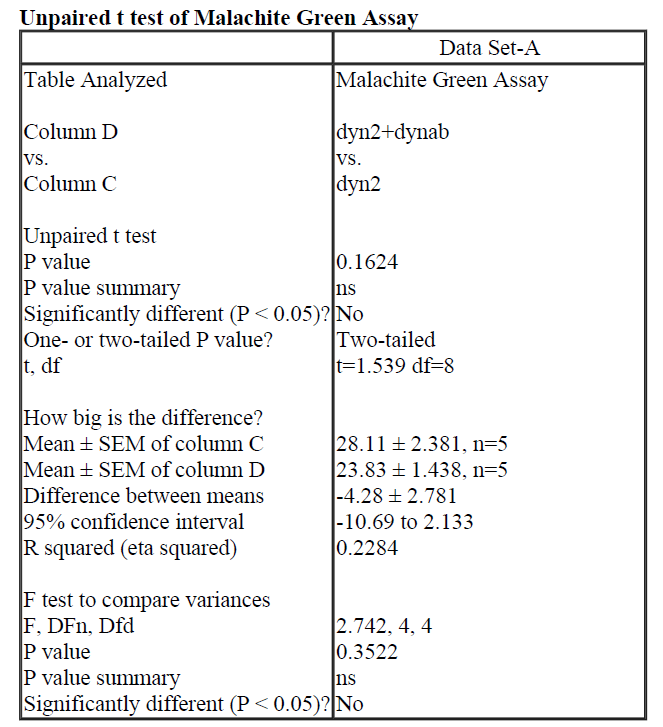

Supplement: Figure 1—source data 1. [file elife-25197-fig1-data1.docx]
