# Supplementary material for: Uncoupling of dynamin polymerization and GTPase activity revealed by the conformation-specific nanobody dynab
Source: eLife. 2017 Oct 12;6:e25197. doi: 10.7554/eLife.25197 (PMC5658065; doi:10.7554/eLife.25197)

**Figure 4-Source Data 1 (panel A)**

Comparison of duration of dynamin 1-2 events with and without dynab (expressed in seconds), and statistical report

| **TKO dyn1_(+dynab)_** | **TKO**  **dyn1** | **TKO**  **dyn2_(+dynab)_** | **TKO**  **dyn2** | **HeLa**  **dyn1_(+dynab)_** | **HeLa**  **dyn1** | **HeLa**  **dyn2_(+dynab)_** | **HeLa**  **dyn2** |
| --- | --- | --- | --- | --- | --- | --- | --- |
| 6.601239 | 4.524978 | 13.16721 | 5.07195 | 5.153392 | 2.144824 | 8.375882 | 6.405968 |
| 3.506503 | 61.07377 | 5.225769 | 28.95743 | 21.02213 | 26.46815 | 2.9534 | 40.80922 |
| 25.75487 | 39.21521 | 4.472581 | 5.159046 | 3.46464 | 2.573333 | 3.499187 | 99.35116 |
| 4.486841 | 4.492665 | 18.77499 | 9.477265 | 2.669299 | 7.66024 | 3.11143 | 13.41522 |
| 5.660996 | 15.0169 | 6.410894 | 11.74911 | 1.966739 | 4.753672 | 43.93438 | 11.97795 |
| 4.869589 | 10.53539 | 5.509642 | 5.901392 | 5.576277 | 6.827628 | 10.19902 | 4.112047 |
| 4.030273 | 3.572758 | 5.908015 | 2.771039 | 2.336081 | 2.708946 | 4.801547 | 4.393519 |
| 8.457853 | 16.489 | 19.98064 | 5.457288 | 3.729542 | 4.021509 | 1.742986 | 3.790491 |
| 10.73303 | 9.172715 | 9.586088 | 3.096717 | 2.668846 | 13.04346 | 4.314331 | 88.48233 |
| 3.651256 | 14.02737 | 12.98406 | 45.22517 | 7.346917 | 4.095624 | 12.35603 | 27.75022 |
| 2.66332 | 12.29389 | 10.40174 | 4.556369 | 3.4194 | 35.62475 | 3.207507 | 6.175061 |
| 5.039145 | 5.233296 | 3.67766 | 15.65644 | 7.462575 | 4.156187 | 6.774017 | 6.389721 |
| 10.24008 | 114.5331 | 21.75257 | 3.040854 | 6.249831 | 5.775817 | 11.03573 | 18.29161 |
| 4.021745 | 7.020952 | 9.933284 | 6.973437 | 7.93673 | 7.717783 | 1.451875 | 3.058198 |
| 2.70845 | 65.25372 | 51.28949 | 3.541927 | 4.941136 | 3.85916 | 3.276381 | 7.504569 |
| 5.686243 | 5.030779 | 4.001522 | 33.58496 | 32.2121 | 7.113542 | 1.694769 | 32.24419 |
| 7.84943 | 83.98646 | 9.01876 | 9.155047 | 3.574212 | 21.5701 | 18.34306 | 9.77023 |
| 6.901536 | 10.73813 | 78.14442 | 25.26704 | 3.123602 | 3.349467 | 3.15457 | 7.020776 |
| 5.480954 | 12.07504 | 4.330427 | 7.839634 | 2.319765 | 4.476027 | 2.048235 | 18.22128 |
| 9.649129 | 22.63752 | 263.7231 | 6.879243 | 2.916059 | 2.827184 | 3.957374 | 4.95233 |
| 7.433951 | 48.15188 | 9.386036 | 4.82994 | 0.06739786 | 3.015802 | 5.298111 | 8.016362 |
| 16.0564 | 8.778161 | 4.734596 | 27.11609 | 3.073704 | 5.217613 | 4.694141 | 21.52055 |
| 24.14206 | 30.52766 | 3.130485 | 0.1545791 | 4.543215 | 16.71506 | 2.172199 | 3.035362 |
| 9.926873 | 7.638529 | 17.77161 | 36.83458 | 2.877222 | 2.411548 | 12.03807 | 4.288622 |
| 3.896017 | 3.704702 | 3.315583 | 8.786293 | 2.47969 | 5.962378 | 4.469718 | 5.212765 |
| 5.566408 | 7.474898 | 74.65894 | 11.90029 | 4.009928 | 2.757293 | 8.353334 | 3.550654 |
| 3.358333 | 2.826365 | 12.16401 | 10.03329 | 10.56666 | 2.915682 | 2.486372 | 2.878723 |
| 3.545781 | 2.596108 | 17.52791 | 26.25038 | 2.136765 | 5.725104 | 2.860197 | 3.884783 |
| 5.607464 | 14.35611 | 4.594904 | 47.89875 | 3.383688 | 4.937236 | 2.169018 | 5.814022 |
| 7.274641 | 2.086164 | 13.81535 | 12.82685 | 11.32386 | 7.947765 | 4.000925 | 15.40464 |
| 3.932723 | 26.25962 | 18.83603 | 8.722117 | 2.40297 | 7.831464 | 2.708007 | 2.971021 |
| 2.67282 | 31.41717 | 3.447096 | 2.953783 | 4.799929 | 4.366054 | 3.80697 | 3.473541 |
| 31.1945 | 3.841589 | 4.606837 | 3.50872 | 5.047917 | 4.568108 | 3.540528 | 82.25118 |
| 9.171896 | 90.42442 | 3.709968 | 17.31584 | 6.635571 | 5.252299 | 10.53068 | 2.525675 |
| 16.58884 | 36.99866 | 99.56223 | 16.93782 | 11.35229 | 4.545159 | 2.919251 | 10.15042 |
| 3.156313 | 3.294497 | 123.4319 | 39.88328 | 2.631402 | 3.000289 | 5.872174 | 6.005116 |
| 41.0473 | 3.68653 | 21.3726 | 3.748488 | 2.133331 | 3.684414 | 2.758659 | 2.804662 |
| 10.08856 | 7.924175 | 18.16434 | 2.420346 | 4.18218 | 4.436196 | 3.401448 | 12.48456 |
| 34.46348 | 16.55578 | 3.697569 | 7.217022 | 2.883841 | 6.157252 | 4.532506 | 13.52044 |
| 3.487533 | 1.074756 | 28.34366 | 3.812683 | 2.750608 | 5.451055 | 23.70976 | 3.989378 |
| 4.08073 | 30.75924 | 6.514387 | 5.57926 | 2.272533 | 6.987157 | 16.39404 | 4.70635 |
| 289.6324 | 6.50694 | 20.46792 | 6.380898 | 3.518247 | 4.709641 | 10.98854 | 6.670433 |
| 5.037975 | 7.275963 | 4.067603 | 2.527562 | 8.421625 | 6.130937 | 13.86294 | 10.85981 |
| 4.276617 | 4.179772 | 9.408644 | 3.880657 | 3.275065 | 0.06870637 | 3.486256 | 4.788293 |
| 41.17758 | 2.28459 | 59.20146 | 3.211427 | 4.026922 | 6.403264 | 23.93331 | 3.406797 |
| 12.35903 | 3.424206 | 10.3697 | 2.574945 | 5.271332 | 3.530581 | 2.569069 | 11.6303 |
| 3.766881 | 6.555314 | 41.78732 | 5.612417 | 4.378217 | 2.474072 | 33.01205 | 4.669048 |
| 6.884367 | 5.766087 | 9.967542 | 4.956883 | 4.152141 | 4.022111 | 3.150086 | 3.590384 |
| 2.862266 | 24.56114 | 9.194146 | 18.92097 | 4.405723 | 36.73041 | 7.102449 | 5.844435 |
| 3.532404 | 5.7763 | 3.741719 | 11.28806 | 3.111307 | 3.230224 | 29.792 | 3.136909 |
| 9.303043 | 15.73776 | 22.89499 | 6.548053 | 3.275686 | 7.351224 | 4.203528 | 3.536311 |
| 9.286666 | 2.792866 | 39.23092 | 3.797534 | 3.620247 | 2.86339 | 15.46358 | 13.9309 |
| 6.574863 | 8.837864 | 5.270787 | 31.73936 | 2.813759 | 3.366234 | 21.65734 | 6.075599 |
| 3.725224 | 4.882405 | 27.79529 | 5.448238 | 7.141806 | 3.96477 | 3.161187 | 6.613861 |
| 32.38119 | 1.726688 | 54.38466 | 16.65612 | 3.561398 | 4.906816 | 22.43829 | 16.29689 |
| 5.241805 | 3.553562 | 29.48901 | 14.0693 | 3.40331 | 4.622643 | 3.679536 | 11.11827 |
| 21.49392 | 12.1937 | 7.636266 | 3.206531 | 5.523773 | 5.14742 | 6.37557 | 1.673852 |
| 15.66882 | 7.263401 | 11.81329 | 24.98887 | 11.35427 | 3.11337 | 4.262374 | 2.682931 |
| 3.625027 | 12.79814 | 6.566845 | 2.002778 | 2.972734 | 4.062367 | 7.993093 | 4.060643 |
| 9.899335 | 6.680822 | 9.172931 | 5.879461 | 3.665252 | 5.95493 | 2.451763 | 29.93503 |
| 6.338922 | 51.04818 | 8.72349 | 9.549813 | 2.593972 | 4.582242 | 12.22758 | 6.234528 |
| 9.374723 | 2.883902 | 4.852861 | 47.33489 | 2.481022 | 6.258424 | 2.753607 | 6.194256 |
| 11.20459 | 8.527285 | 15.15425 | 5.141756 | 9.977154 | 4.110735 | 14.1561 | 5.056983 |
| 8.429011 | 2.338108 | 34.15361 | 56.2324 | 2.394521 | 4.625299 | 17.20047 | 8.839954 |
| 11.9604 | 4.47057 | 13.85996 | 3.994671 | 4.565697 | 2.811487 | 4.134029 | 7.446941 |
| 8.546485 | 11.03173 | 7.063757 | 18.72345 | 5.524896 | 11.47723 | 2.392048 | 3.070573 |
| 3.887546 | 4.597144 | 29.63056 | 3.142082 | 2.951166 | 3.726056 | 2.282273 | 5.37977 |
| 6.406134 | 4.753874 | 3.591331 | 2.861092 | 2.143594 | 2.714955 | 5.473784 | 19.89631 |
| 9.86976 | 19.16633 | 4.623187 | 2.41911 | 2.906468 | 3.806467 | 3.252548 | 2.862087 |
| 3.987934 | 4.532532 | 11.80435 | 3.884327 | 3.716472 | 2.996128 | 4.545791 | 9.543842 |
| 5.298746 | 12.31446 | 5.918113 | 2.767108 | 1.549762 | 4.693609 | 5.516869 | 10.14163 |
| 2.612122 | 15.47363 | 4.924493 | 5.307009 | 2.86479 | 6.06681 | 2.519397 | 2.843841 |
| 8.544926 | 2.651967 | 4.365512 | 23.80107 | 3.458144 | 7.600008 | 10.21003 | 2.447031 |
| 5.606765 | 5.908823 | 8.295631 | 27.51543 | 4.479673 | 5.141276 | 14.19016 | 5.39683 |
| 5.037157 | 3.603675 | 3.647801 | 75.66692 | 2.888395 | 4.951772 | 2.229649 | 13.83844 |
| 8.375821 | 7.579117 | 12.42799 | 2.641575 | 3.597457 | 3.223876 | 4.212824 | 10.21129 |
| 34.89538 | 25.1204 | 4.31396 | 2.321022 | 3.003048 | 3.185033 | 2.476377 | 3.643292 |
| 13.42367 | 11.0786 | 25.31272 | 6.877375 | 2.743035 | 3.412887 | 2.136305 | 3.443023 |
| 4.329061 | 6.960547 | 5.321562 | 8.766913 | 2.140017 | 6.679461 | 10.58352 | 15.49583 |
| 3.032381 | 4.335007 | 6.615696 | 1.927259 | 7.517755 | 6.316198 | 5.67535 | 3.06611 |
| 3.326238 | 2.720503 | 7.846164 | 41.86075 | 4.135083 | 3.125772 | 5.668877 | 36.51184 |
| 24.30967 | 15.58393 | 6.145126 | 15.12029 | 3.338814 | 10.29217 | 4.889891 | 21.79845 |
| 4.915221 | 4.215854 | 6.205803 | 2.391846 | 3.629208 | 3.807346 | 3.659689 | 4.803349 |
| 8.05799 | 6.361451 | 5.79036 | 14.74944 | 3.021896 | 5.98702 | 4.455425 | 6.102267 |
| 3.215723 | 4.573369 | 4.727397 | 7.796513 | 3.831994 | 2.555582 | 14.88402 | 2.400132 |
| 16.34647 | 6.399834 | 6.126765 | 9.421255 | 2.499503 | 4.078238 | 82.02081 | 3.932879 |
| 24.67756 | 17.84053 | 6.84294 | 4.487885 | 5.624052 | 3.487392 | 11.92007 | 3.670624 |
| 11.34904 | 2.305266 | 3.256747 | 11.40437 | 2.241478 | 6.774752 | 15.21543 | 4.142236 |
| 17.43212 | 19.65774 | 3.86668 | 3.724805 | 3.009913 | 4.342951 | 4.882149 | 3.362953 |
| 4.318324 | 3.576428 | 6.7612 | 7.270838 | 5.99939 | 18.47267 | 2.471474 | 5.481699 |
| 6.85176 | 3.247959 | 25.58028 | 5.38427 | 5.714248 | 7.921349 | 4.746627 | 11.49904 |
| 35.80468 | 3.617101 | 15.60861 | 2.695361 | 3.530219 | 8.118904 | 3.962164 | 38.38627 |
| 17.6364 | 2.009984 | 3.80801 | 3.013678 | 19.84376 | 3.89229 | 3.164702 | 9.466091 |
| 8.018537 | 4.156 | 1.546486 | 3.554331 | 4.839716 | 4.026989 | 2.778726 | 24.69972 |
| 5.13004 | 39.75295 | 6.832849 | 15.35315 | 4.765405 | 2.64179 | 3.425226 | 3.44026 |
| 3.760289 | 141.5428 | 6.128088 | 2.56451 | 3.736364 | 8.009119 | 5.132301 | 13.09747 |
| 4.465593 | 14.22388 | 26.67641 | 10.59795 | 3.432893 | 2.979043 | 3.903958 | 6.878491 |
| 4.240802 | 36.38444 | 5.425948 | 4.494844 | 5.759855 | 3.809498 | 4.000445 | 1.721911 |
| 5.23154 | 12.39263 | 13.00051 | 15.22183 | 4.74423 | 3.393898 | 13.32255 | 3.71487 |
| 54.49449 | 4.195868 | 11.44847 | 9.628199 | 5.206048 | 4.852022 | 25.96698 | 2.969354 |
| 11.76827 | 63.40752 | 12.1472 | 16.31407 | 19.39507 | 1.408708 | 5.612551 | 17.60801 |
| 27.43649 | 59.38789 | 4.53909 | 2.073228 | 4.715054 | 29.84321 | 4.139998 | 19.58718 |
| 6.851221 | 2.998994 | 4.522498 | 2.419249 | 2.143698 | 7.421633 | 3.549013 | 2.94623 |
| 7.938857 | 25.42432 | 3.388105 | 14.61614 | 3.789955 | 8.625876 | 3.330629 | 7.483245 |
| 4.533852 | 25.60711 | 3.372577 | 20.27583 | 2.810685 | 11.36521 | 5.528918 | 4.10626 |
| 16.03319 | 17.44368 | 11.89888 | 6.460814 | 3.447537 | 4.724612 | 7.93918 | 34.32355 |
| 6.022697 | 34.32534 | 3.445469 | 5.949329 | 4.093304 | 39.93172 | 3.625626 | 5.792248 |
| 9.114733 | 4.236251 | 6.971035 | 6.164864 | 12.70554 | 5.191785 | 14.62553 | 10.16316 |
| 8.343234 | 4.060329 | 5.013208 | 4.102294 | 6.416002 | 3.619402 | 9.777839 | 9.314617 |
| 5.011595 | 4.154122 | 4.040196 | 15.7658 | 4.369156 | 2.520291 | 4.047268 | 5.227694 |
| 10.01492 | 3.098668 | 2.461752 | 2.691793 | 186.6253 | 15.7358 | 10.12175 | 2.813455 |
| 7.105522 | 6.524436 | 4.385135 | 13.0755 | 8.664303 | 5.913122 | 4.512361 | 7.85366 |
| 2.535899 | 9.470869 | 3.085811 | 4.997276 | 4.307275 | 4.858915 | 3.476244 | 5.369787 |
| 9.128949 | 6.316506 | 4.844326 | 10.29244 | 2.511629 | 15.80407 | 6.006478 | 3.012163 |
| 12.19291 | 2.841058 | 12.74232 | 3.969005 | 3.270992 | 7.755431 | 42.79696 | 3.067254 |
| 5.246868 | 2.312285 | 10.06827 | 41.9231 | 26.75703 | 3.761014 | 7.229988 | 3.14783 |
| 14.92023 | 21.05831 | 2.687056 | 31.75305 | 2.408058 | 3.705067 | 8.633982 | 62.7187 |
| 7.363852 | 5.322717 | 6.418931 | 3.017561 | 4.086239 | 9.865142 | 11.65017 | 9.30648 |
| 3.60881 | 36.60379 | 4.171983 | 50.90623 | 6.761764 | 7.433997 | 7.566673 | 9.962738 |
| 3.909614 | 10.74355 | 5.966401 | 33.06275 | 6.724588 | 6.000983 | 8.328494 | 4.95465 |
| 7.392715 | 0.1149603 | 3.216907 | 11.87156 | 3.450027 | 6.173136 | 15.30795 | 5.939599 |
| 11.26654 | 3.186147 | 8.244109 | 5.688238 | 7.148999 | 2.624433 | 4.333529 | 3.735198 |
| 29.2915 | 4.189323 | 24.91424 | 3.349796 | 6.329277 | 3.63738 | 14.11713 | 3.265586 |
| 33.35446 | 8.657123 | 8.793033 | 10.43686 | 4.21329 | 2.921723 | 8.143585 | 3.219385 |
| 18.54682 | 3.258835 | 5.682665 | 3.651125 | 6.814925 | 10.01203 | 3.320771 | 3.460705 |
| 15.58385 | 3.649267 | 11.61493 | 23.27868 | 3.516283 | 9.007093 | 6.557391 | 9.145532 |
| 6.9534 | 10.5422 | 5.599602 | 11.75842 | 3.157829 | 4.303628 | 6.785255 | 3.753415 |
| 10.03725 | 3.91295 | 8.79696 | 39.61569 | 2.59015 | 2.856867 | 4.383392 | 6.372641 |
| 9.759346 | 5.290249 | 3.013871 | 17.38471 | 4.55403 | 4.432014 | 15.46954 | 10.43718 |
| 4.315737 | 5.161783 | 3.222785 | 6.372666 | 11.45077 | 2.400511 | 11.99878 | 21.8711 |
| 7.993102 | 19.21811 | 9.111304 | 8.280103 | 2.900394 | 5.476508 | 15.73957 | 2.635087 |
| 11.18344 | 4.891324 | 7.976821 | 19.60979 | 5.512717 | 4.428822 | 3.126488 | 3.204732 |
| 4.858521 | 4.237313 | 4.208205 | 59.83601 | 2.437899 | 5.34399 | 15.50544 | 5.269246 |
| 16.86399 | 2.687548 | 3.920396 | 6.271286 | 5.19757 | 5.539018 | 23.01756 | 4.171438 |
| 3.620277 | 6.131513 | 26.24392 | 13.70907 | 2.282331 | 7.415412 | 4.347872 | 7.154926 |
| 3.609698 | 5.959666 | 69.56977 | 12.22029 | 8.623433 | 4.749955 | 32.6683 | 121.9377 |
| 6.399356 | 3.720082 | 5.475939 | 8.424189 | 2.408806 | 6.277563 | 3.93613 | 2.531043 |
| 6.70967 | 1.776995 | 4.069629 | 3.88265 | 5.271201 | 14.14169 | 13.87799 | 5.649222 |
| 7.247655 | 2.403636 | 5.982146 | 6.634278 | 3.258546 | 2.854375 | 5.432714 | 4.490389 |
| 62.51002 | 5.907444 | 7.38865 | 6.146689 | 4.236821 | 4.106934 | 3.54397 | 4.052115 |
| 3.920823 | 5.280457 | 4.110736 | 15.35409 | 2.585475 | 17.05579 | 3.780114 | 49.95034 |
| 4.194264 | 29.23986 | 12.95947 | 19.62396 | 9.628614 | 12.07971 | 2.628377 | 6.291851 |
| 4.74872 | 13.09481 | 5.769785 | 20.34213 | 3.17551 | 4.521058 | 4.599042 | 2.351246 |
| 9.810928 | 26.88969 | 9.708482 | 2.86182 | 6.424105 | 5.330828 | 12.69817 | 2.712874 |
| 2.997025 | 38.55923 | 2.455581 | 19.96775 | 8.316773 | 7.845568 | 4.497609 | 7.44878 |
| 2.053964 | 5.412281 | 6.565662 | 3.556487 | 4.998872 | 40.00477 | 3.852705 | 17.8961 |
| 11.6966 | 3.304034 | 5.189806 | 19.49516 | 3.05007 | 3.972383 | 2.760276 | 15.92524 |
| 6.851951 | 25.92568 | 4.171609 | 5.107965 | 26.55367 | 4.304214 | 3.539871 | 3.263043 |
| 3.934465 | 4.489869 | 174.4749 | 4.339009 | 4.050091 | 5.090861 | 5.09119 | 6.010877 |
| 34.49156 | 5.367065 | 16.14113 | 3.498008 | 3.791594 | 4.924802 | 18.6807 | 23.58813 |
| 9.434631 | 78.23957 | 4.27447 | 8.698269 | 4.405251 | 9.985527 | 7.489876 | 84.17985 |
| 5.318674 | 58.08512 | 4.247444 | 12.17228 | 11.53689 | 7.674038 | 14.28244 | 5.166012 |
| 9.612067 | 11.26362 | 4.82913 | 17.34433 | 5.51839 | 4.015499 | 9.252999 | 3.86337 |
| 4.542065 | 3.014999 | 3.79376 | 6.523607 | 8.747226 | 2.700009 | 4.155923 | 36.87582 |
| 3.664837 | 6.463201 | 48.91218 | 22.51049 | 5.55821 | 29.68462 | 7.407035 | 2.818008 |
| 7.910212 | 4.239202 | 7.231506 | 69.346 | 5.65337 | 4.443249 | 68.36897 | 2.58278 |
| 5.515055 | 3.331457 | 25.82561 | 2.349548 | 4.242633 | 8.951313 | 4.576773 | 3.273803 |
| 5.99265 | 3.657585 | 12.0744 | 23.5504 | 7.590431 | 3.240144 | 4.743496 | 51.69692 |
| 12.99139 | 3.003094 | 7.56512 | 3.563547 | 5.157208 | 4.761173 | 3.43235 | 5.749739 |
| 2.697125 | 4.986517 | 4.686913 | 42.82735 | 2.227666 | 5.582947 | 8.92502 | 12.53179 |
| 3.113596 | 25.98368 | 7.349073 | 4.035892 | 5.360243 | 14.23485 | 22.01432 | 4.874807 |
| 3.412664 | 10.17305 | 4.152588 | 9.514321 | 4.905808 | 11.81034 | 5.899964 | 21.15613 |
| 1.957097 | 5.071008 | 4.385338 | 51.44315 | 6.371337 | 1.823646 | 18.10568 | 2.663753 |
| 3.261529 | 6.509756 | 6.877095 | 3.581849 | 2.359765 | 6.824198 | 10.31615 | 2.819144 |
| 1.698347 | 6.339306 | 7.03721 | 16.19203 | 5.804431 | 4.55707 | 3.119625 | 8.235305 |
| 3.812539 | 4.552719 | 7.803881 | 3.838333 | 9.267389 | 4.274323 | 3.011083 | 4.95685 |
| 3.480757 | 3.029846 | 4.804303 | 5.478717 | 5.966358 | 5.076483 | 25.91432 | 6.831902 |
| 4.054862 | 3.094457 | 56.25779 | 17.18864 | 4.701252 | 7.503837 | 5.57855 | 3.682566 |
| 5.256277 | 6.321622 | 5.416386 | 4.270287 | 6.039544 | 3.126642 | 14.3919 | 5.480632 |
| 9.103899 | 24.98473 | 11.03415 | 12.52278 | 6.254511 | 17.01735 | 3.326533 | 1.64843 |
| 3.143964 | 3.669596 | 8.542464 | 174.2549 | 3.371107 | 4.947422 | 3.101223 | 3.414912 |
| 5.707292 | 16.79426 | 4.227625 | 7.556117 | 3.299872 | 7.668629 | 7.276956 | 4.377029 |
| 9.202372 | 3.102547 | 4.1771 | 15.46497 | 3.245867 | 3.533175 | 4.026817 | 29.41838 |
| 7.372858 | 96.53697 | 9.236404 | 19.06702 | 3.844421 | 5.629872 | 4.991699 | 5.819494 |
| 3.677961 | 4.638245 | 25.09481 | 4.946985 | 4.606849 | 2.625292 | 5.607867 | 5.624534 |
| 6.980219 | 22.57824 | 12.72817 | 48.5465 | 2.480192 | 3.517199 | 4.917695 | 3.22698 |
| 5.386555 | 3.080159 | 4.634752 | 7.995771 | 3.025101 | 3.208399 | 4.840033 | 3.960547 |
| 86.24833 | 5.977869 | 5.929452 | 4.493954 | 5.820499 | 1.768564 | 4.503224 | 4.351884 |
| 2.914724 | 5.156504 | 3.785853 | 56.93766 | 2.692196 | 2.851905 | 10.94715 | 5.528634 |
| 5.943244 | 13.09039 | 4.224237 | 2.433972 | 3.810178 | 2.557046 | 2.293502 | 4.435149 |
| 4.176275 | 4.335301 | 8.585425 | 14.87447 | 4.290545 | 3.126135 | 7.343382 | 3.152522 |
| 4.427634 | 21.53657 | 6.053139 | 53.62161 | 3.093176 | 7.151145 | 3.752802 | 15.11621 |
| 1.77803 | 21.52523 | 21.62441 | 2.662334 | 2.692436 | 13.38815 | 7.919574 | 3.351939 |
| 4.04854 | 21.43443 | 3.356513 | 46.61614 | 3.724098 | 6.830628 | 5.769078 | 3.741589 |
| 5.124496 | 3.728404 | 9.740605 | 14.50345 | 5.539906 | 5.674506 | 19.37253 | 3.938606 |
| 22.6047 | 3.997258 | 118.9276 | 55.04556 | 7.715841 | 7.534492 | 4.108689 | 2.461592 |
| 8.26875 | 6.590295 | 74.68453 | 5.703985 | 4.667504 | 6.080632 | 5.816577 | 3.162952 |
| 5.11018 | 8.507186 | 4.660533 | 11.01014 | 3.383423 | 5.123445 | 7.717924 | 30.77347 |
| 3.791409 | 14.36081 | 6.552241 | 4.135645 | 4.050063 | 4.419529 | 9.291615 | 2.865158 |
| 3.695879 | 3.121983 | 6.883797 | 104.8094 | 3.367145 | 3.953065 | 3.901431 | 3.166482 |
| 5.621859 | 2.944926 | 75.73339 | 51.1843 | 3.712569 | 5.863329 | 3.946452 | 20.47429 |
| 2.85839 | 55.3494 | 4.774509 | 20.19639 | 3.71932 | 3.252323 | 9.677586 | 4.909626 |
| 5.575926 | 17.55581 | 2.796174 | 27.79129 | 5.992081 | 4.753885 | 5.29237 | 3.848292 |
| 2.861722 | 5.255711 | 19.51279 | 23.81277 | 4.177862 | 7.745935 | 2.936264 | 6.508806 |
| 11.53907 | 3.717417 | 13.65409 | 6.789954 | 2.203545 | 25.02154 | 5.165866 | 11.94404 |
| 4.281544 | 4.480198 | 23.35519 | 8.129363 | 2.459509 | 59.72823 | 7.715729 | 3.682676 |
| 9.318782 | 6.242758 | 8.004214 | 14.34524 | 3.358088 | 3.96452 | 0.1387732 | 30.84683 |
| 5.213916 | 3.663193 | 17.858 | 163.1829 | 1.536456 | 26.3214 | 2.712561 | 3.400089 |
| 8.842998 | 3.450857 | 4.493874 | 10.05253 | 2.765333 | 7.493517 | 20.84559 | 8.126196 |
| 4.558892 | 6.86134 | 31.63354 | 6.245898 | 3.260368 | 3.038349 | 3.250531 | 4.988121 |
| 3.372264 | 3.700615 | 11.79336 | 4.385054 | 4.728901 | 2.935631 | 17.49891 | 20.4842 |
| 4.294738 | 3.037882 | 38.95685 | 9.133905 | 6.397839 | 7.305325 | 4.530854 | 5.535962 |
| 45.14282 | 11.78799 | 5.604908 | 2.538294 | 1.999051 | 2.92119 | 8.475431 | 43.99793 |
| 2.915927 | 3.726785 | 14.89115 | 9.129005 | 2.769737 | 6.516695 | 7.137334 | 2.161753 |
| 2.892428 | 4.866509 | 27.37437 | 19.79486 | 2.591619 | 4.723128 | 4.593011 | 3.839573 |
| 8.076951 | 3.983973 | 15.56407 | 9.45176 | 5.342558 | 4.693094 | 3.750421 | 18.16849 |
| 7.986841 | 18.45577 | 9.803103 | 11.99153 | 5.004059 | 5.233246 | 13.59964 | 3.64153 |
| 3.606245 | 61.55204 | 13.0093 | 2.533495 | 4.373154 | 1.869843 | 3.856683 | 6.173067 |
| 3.538414 | 15.42818 | 81.7196 | 4.673554 | 4.719927 | 4.276581 | 19.83306 | 9.279402 |
| 6.562732 | 6.099323 | 138.8298 | 58.74094 | 7.330477 | 2.889731 | 1.933115 | 5.555101 |
| 3.396733 | 10.45513 | 5.124485 | 2.885064 | 5.433887 | 18.02865 | 3.084258 | 4.237027 |
| 5.565812 | 49.527 | 36.20427 | 13.69416 | 4.873335 | 8.839471 | 7.003986 | 24.18179 |
| 5.589819 | 8.374563 | 16.10084 | 15.73566 | 3.69417 | 6.256117 | 2.480832 | 4.162966 |
| 4.99152 | 2.247113 | 6.55274 | 14.00199 | 2.059278 | 8.389642 | 2.95276 | 5.94512 |
| 3.322018 | 4.633859 | 10.8075 | 5.735371 | 3.934188 | 4.445441 | 7.623952 | 5.246056 |
| 7.695147 | 5.504248 | 8.570738 | 10.08262 | 4.446733 | 4.789996 | 4.192572 | 2.756066 |
| 6.118463 | 5.619516 | 19.9127 | 8.602406 | 6.680808 | 3.588179 | 2.559238 | 2.201195 |
| 8.313094 | 5.880748 | 13.38008 | 22.69375 | 5.627133 | 2.160058 | 5.720595 | 2.705256 |
| 4.751836 | 31.83582 | 7.71465 | 66.8978 | 3.415457 | 3.762202 | 4.167871 | 7.222757 |
| 6.649975 | 9.274588 | 172.588 | 17.03402 | 3.663166 | 3.67034 | 4.221669 | 20.73285 |
| 5.779444 | 26.89444 | 149.2239 | 14.11261 | 5.545373 | 4.479167 | 22.62977 | 4.343616 |
| 4.271715 | 11.67344 | 4.623735 | 17.75896 | 1.531749 | 6.326945 | 2.846246 | 9.61739 |
| 6.992066 | 4.889761 | 37.00671 | 40.82332 | 2.32317 | 6.837099 | 13.3436 | 5.198407 |
| 4.010108 | 1.608594 | 9.616763 | 4.361812 | 3.022662 | 3.260975 | 4.970001 | 2.579665 |
| 7.779538 | 13.72356 | 3.223971 | 2.627837 | 5.956467 | 2.892435 | 6.442256 | 19.72834 |
| 7.762373 | 10.30008 | 36.01799 | 22.0378 | 3.696216 | 3.442749 | 3.36192 | 5.633161 |
| 7.076436 | 17.86157 | 20.33345 | 43.97372 | 4.827001 | 4.080084 | 24.75628 | 2.964508 |
| 15.42269 | 2.917989 | 18.37951 |  | 3.742849 | 2.804419 | 17.67551 | 3.770308 |
| 9.142713 | 10.655 | 4.357965 |  | 5.056397 | 10.7849 | 4.036809 | 67.39694 |
| 10.43398 | 3.088376 | 17.44017 |  | 3.560583 | 8.704207 | 6.567292 | 4.727866 |
| 14.88354 | 11.91164 | 16.85471 |  | 18.29983 | 4.882364 | 2.58234 | 3.13575 |
| 4.301896 | 62.61166 | 2.049132 |  | 4.507099 | 25.47409 | 4.190949 | 2.676477 |
| 4.380212 | 4.925145 | 28.35785 |  | 4.150426 | 2.816858 | 3.466703 | 3.624316 |
| 0.04062336 | 2.99717 | 5.579033 |  | 5.884307 | 5.049921 | 20.89578 | 8.190829 |
| 40.07052 | 9.077663 | 11.31348 |  | 4.089399 | 7.532841 | 4.382726 | 4.039531 |
| 3.46478 | 4.171444 | 6.067636 |  | 3.464284 | 5.201306 | 5.089384 | 26.44421 |
| 5.660808 | 7.584134 | 5.137618 |  | 5.001092 | 8.185896 | 3.315695 | 3.013695 |
| 3.744282 | 9.365093 | 3.939713 |  | 3.432851 | 13.62173 | 4.003428 | 6.08334 |
| 2.977684 | 3.891996 | 25.52488 |  | 3.489057 | 3.414894 | 4.360392 | 3.232432 |
| 9.55021 | 24.91487 | 9.454031 |  | 11.79509 | 6.71077 | 14.32361 | 2.846121 |
| 4.624945 | 7.00696 | 13.95434 |  | 4.704708 | 27.73969 | 13.00394 | 11.55309 |
| 4.01183 | 5.059473 | 3.409006 |  | 10.87948 | 3.103208 | 5.807992 | 2.747956 |
| 4.724096 | 6.906458 | 12.3465 |  | 3.710453 | 3.149767 | 25.71277 | 4.759589 |
| 3.081964 | 3.534424 | 9.558196 |  | 3.089425 | 8.316833 | 4.379148 | 4.937865 |
| 8.767394 | 4.172008 | 5.104276 |  | 3.610353 | 3.984331 | 3.003331 | 3.478533 |
| 34.79025 | 7.131944 | 5.350594 |  | 7.669233 | 3.070344 | 13.94849 | 3.127417 |
| 4.70221 | 4.82299 | 19.79153 |  | 2.146665 | 4.703562 | 5.445119 | 6.457216 |
| 2.85287 | 2.70472 | 6.146181 |  | 3.495884 | 5.542558 | 2.734426 | 2.296202 |
| 3.337415 | 9.218713 | 7.878509 |  | 2.945311 | 4.046344 | 3.530206 | 3.713605 |
| 11.92834 | 4.83796 | 7.082289 |  | 4.776181 | 3.386736 | 8.583469 | 4.335729 |
| 14.16084 | 4.368351 | 13.29337 |  | 3.164632 | 7.45026 | 4.694687 | 13.46229 |
| 4.909853 | 11.11333 | 5.532242 |  | 3.192417 | 7.56013 | 3.280933 | 3.186194 |
| 4.732589 | 5.687887 | 9.043407 |  | 1.816985 | 2.293736 | 2.04876 | 3.879825 |
| 15.15814 | 7.506115 | 3.490898 |  | 4.544088 | 10.39397 | 2.491227 | 10.9485 |
| 7.118374 | 37.29823 | 4.045372 |  | 4.675646 | 5.780416 | 12.41006 | 5.034321 |
| 5.219141 | 5.404758 | 4.941267 |  | 7.360615 | 11.03368 | 1.751518 | 2.423001 |
| 9.680541 | 4.437149 | 39.52813 |  | 2.352886 | 3.934425 | 5.227752 | 17.74648 |
| 4.848881 | 8.967532 | 4.975129 |  | 2.949015 | 7.19836 | 3.692912 | 25.70249 |
| 4.253516 | 7.844133 | 4.022162 |  | 5.48388 | 7.975308 | 3.79247 | 3.222613 |
| 5.287558 | 3.101922 | 5.618433 |  | 8.157118 | 14.78178 | 9.100814 | 6.949225 |
| 4.449408 | 96.72095 | 4.505091 |  | 3.824744 | 4.144284 | 10.12015 | 9.342578 |
| 5.814721 | 3.105471 | 19.48909 |  | 5.785802 | 3.883245 | 21.57749 | 3.4506 |
| 8.279238 | 21.81481 | 5.035456 |  | 3.856106 | 17.76481 | 4.564982 | 3.681849 |
| 3.889213 | 10.87933 | 3.956656 |  | 4.541925 | 14.38948 | 11.952 | 25.1841 |
| 6.384449 | 33.80551 | 10.97533 |  | 2.937423 | 8.355851 | 56.1397 | 3.152689 |
| 5.323805 | 3.181604 | 4.303393 |  | 37.19357 | 5.251402 | 3.638467 | 3.326038 |
| 3.724129 | 3.173944 | 9.368612 |  | 5.978399 | 17.51774 | 9.355865 | 8.98818 |
| 5.60606 | 32.75328 | 7.656224 |  | 3.045578 | 4.641074 | 3.354674 | 3.396422 |
| 6.244517 | 44.73737 | 5.528324 |  | 3.999073 | 9.388584 | 1.884949 | 4.618799 |
| 5.511223 | 2.119855 | 40.73651 |  | 4.463556 | 5.49249 | 5.426148 | 19.40875 |
| 6.02435 | 20.73243 | 6.645213 |  | 2.351654 | 42.65221 | 2.267816 | 33.06165 |
| 3.165485 | 3.889752 | 7.167282 |  | 2.278873 | 10.20187 | 13.90564 | 3.385646 |
| 4.78589 | 2.596624 | 5.399017 |  | 6.594135 | 20.4922 | 24.02731 | 13.04066 |
| 3.12392 | 4.608073 | 3.777481 |  | 3.386564 | 9.724187 | 7.246894 | 3.354919 |
| 2.801918 | 12.90956 | 14.32438 |  | 2.468136 | 11.0429 | 1.680668 | 3.619482 |
| 12.25824 | 5.30991 | 3.649547 |  | 3.98004 | 2.605414 | 6.892484 | 5.820824 |
| 2.635849 | 3.922005 | 7.815723 |  | 3.698912 | 4.511626 | 9.920389 | 19.50852 |
| 8.376735 | 2.698988 | 5.510051 |  | 3.792272 | 4.156858 | 4.75388 | 5.100319 |
| 2.751365 | 4.16272 | 12.41447 |  | 2.723328 | 11.64612 | 3.581583 | 4.384118 |
| 5.99678 | 3.021487 | 32.96571 |  | 4.181459 | 3.900535 | 2.538024 | 13.63228 |
| 4.318438 | 3.908163 | 7.801152 |  | 7.315055 | 6.343748 | 4.588692 | 3.483868 |
| 7.541431 | 6.115508 | 5.221347 |  | 4.442387 | 5.814767 | 10.59531 | 3.266418 |
| 0.04285149 | 75.03742 | 4.508008 |  | 4.375016 | 2.940364 | 3.832807 | 8.6796 |
| 16.24216 | 6.243619 | 6.240302 |  | 2.754582 | 21.89614 | 1.690452 | 3.920511 |
| 2.410538 | 5.771584 | 3.975317 |  | 3.39264 | 7.056359 | 11.50213 | 6.080019 |
| 10.09539 | 7.161415 | 6.71226 |  | 2.759486 | 2.325864 | 5.27662 | 2.608176 |
| 8.639343 | 33.69891 | 5.62747 |  | 3.153381 | 10.04815 | 3.647406 | 4.58141 |
| 4.751951 | 4.018026 | 8.238041 |  | 2.470985 | 3.069656 | 2.714138 | 2.847871 |
| 4.80213 | 4.144284 | 28.68241 |  | 3.29425 | 37.68346 | 2.312293 | 3.042882 |
| 5.977094 | 17.92016 | 3.21931 |  | 4.696551 | 9.167322 | 3.561723 | 2.971871 |
| 8.660271 | 3.780844 | 4.165478 |  | 9.678536 | 13.98766 | 2.882371 | 3.884375 |
| 25.45924 | 4.213347 | 16.77053 |  | 5.479321 | 3.155122 | 2.931744 | 3.825826 |
| 12.39786 | 8.766254 | 13.26751 |  | 7.474984 | 18.85788 | 34.6 | 10.11783 |
| 3.761934 | 3.197341 | 22.15343 |  | 5.462753 | 3.186795 | 26.815 | 4.079399 |
| 6.011902 | 3.490417 | 9.039318 |  | 4.624375 | 3.064917 | 34.2886 | 2.241048 |
| 2.714024 | 8.443978 | 11.9528 |  | 6.632537 | 5.964297 | 5.263022 | 7.993267 |
| 3.017363 | 52.65351 | 2.956631 |  | 3.337058 | 4.574408 | 12.268 | 3.513768 |
| 2.991777 | 2.939155 | 10.84465 |  | 7.375797 | 13.36283 | 122.6037 | 4.710921 |
| 8.497955 | 4.362239 | 6.904407 |  | 2.640757 | 3.372995 | 41.52 | 3.492304 |
| 3.682882 | 3.929809 | 6.60074 |  | 2.130654 | 1.927835 | 10.38 | 3.015811 |
| 6.053695 | 2.382881 | 11.23325 |  | 3.024171 | 4.140435 | 4.201004 | 4.473824 |
| 11.87306 | 5.38815 | 24.16445 |  | 3.075987 | 13.34749 | 2.305608 | 3.435379 |
| 3.936437 | 2.782751 | 11.26212 |  | 2.988907 | 10.00648 | 3.012568 | 2.965067 |
| 3.923166 | 7.082828 | 17.55957 |  | 4.505031 | 3.580884 | 3.158052 | 7.742908 |
| 4.841562 | 2.430399 | 27.56569 |  | 4.543441 | 2.820358 | 14.3878 | 5.761216 |
| 2.778406 | 2.504275 | 16.48417 |  | 8.176086 | 3.69859 | 6.530656 | 16.86452 |
| 2.051529 | 11.39813 | 25.55696 |  | 2.852143 | 5.864445 | 15.76201 | 4.862608 |
| 4.008139 | 2.446103 | 10.46268 |  | 8.464497 | 18.48495 | 49.85256 | 4.94731 |
| 14.804 | 8.354468 | 8.901562 |  | 5.531025 | 18.35873 | 8.276536 | 3.175944 |
| 14.804 | 11.38963 | 52.04753 |  | 4.232918 | 1.938764 | 42.20562 | 25.61124 |
| 4.488493 | 2.882523 | 61.29009 |  | 3.916415 | 7.310855 | 2.45376 | 4.798148 |
| 3.383193 | 15.2505 | 18.45032 |  | 3.367305 | 14.96102 | 33.44004 | 3.82155 |
| 8.544699 | 4.549091 | 16.52413 |  | 4.903393 | 11.66412 | 6.573628 | 4.766987 |
| 6.534238 | 4.30981 | 63.04087 |  | 5.001179 | 6.442881 | 24.58772 | 2.755901 |
| 4.772032 | 4.508134 | 84.79265 |  | 8.251942 | 26.67761 | 6.2208 | 5.498069 |
| 3.880112 | 15.82235 | 24.96808 |  | 5.192432 | 2.371648 | 4.58507 | 2.040997 |
| 7.477232 | 3.211864 | 18.89517 |  | 6.144846 | 4.31569 | 3.489924 | 2.558163 |
| 3.117263 | 7.516621 | 121.3563 |  | 4.386652 | 7.535072 | 10.7111 | 3.918743 |
| 7.473566 | 4.19608 | 34.14069 |  | 2.875868 | 9.541608 | 6.716168 | 4.524467 |
| 3.922775 | 7.815163 | 17.23042 |  | 3.068693 | 5.03247 | 4.654028 | 4.10372 |
| 3.994827 | 6.448004 | 57.69523 |  | 5.393245 | 9.740879 | 20.83693 | 3.871013 |
| 8.719735 | 4.307669 | 7.929545 |  | 14.42248 | 19.10109 | 2.648051 | 18.05339 |
| 2.894929 | 11.39322 | 49.12331 |  | 4.734394 | 2.397921 | 4.082441 | 11.75105 |
| 8.870029 | 3.099889 | 3.287023 |  | 6.254828 | 12.61387 | 6.437026 | 2.326828 |
| 2.892849 | 4.060648 | 4.997186 |  | 4.269104 | 18.48928 | 44.91036 | 2.717504 |
| 15.88422 | 18.34868 | 9.83792 |  | 12.08486 | 4.960242 | 11.8678 | 8.965095 |
| 6.907551 | 4.861553 | 24.22433 |  | 3.161127 | 7.006405 | 4.7056 | 3.180729 |
| 4.387765 | 7.896549 | 4.581924 |  | 15.29136 | 5.420416 | 17.95191 | 4.804365 |
| 3.74782 | 27.93748 | 9.552371 |  | 7.737373 | 4.925776 | 21.24087 | 12.13246 |
| 3.009335 | 3.633438 | 4.704533 |  | 12.78456 | 38.84917 | 3.806607 | 9.208531 |
| 3.963594 | 9.863427 | 3.866858 |  | 3.125841 | 13.89323 | 3.834531 | 5.978179 |
| 5.193597 | 3.60865 | 5.265091 |  | 5.041691 | 66.94694 | 5.423293 | 3.153131 |
| 15.43111 | 3.072229 | 4.718308 |  | 20.12117 | 9.768591 | 45.30629 | 3.173545 |
| 29.5054 | 10.05735 | 18.23197 |  | 6.912 | 23.33001 | 24.77516 | 7.60603 |
| 2.828272 | 2.18679 | 3.430473 |  | 2.456142 | 8.36266 | 47.48323 | 3.342777 |
| 3.31834 | 5.499878 | 6.952669 |  | 4.955375 | 34.36063 | 47.56 | 3.135315 |
| 6.855222 | 23.93829 | 18.09745 |  | 2.553674 | 3.251916 | 18.86983 | 2.849362 |
| 5.401934 | 2.438204 | 5.885726 |  | 2.804007 | 7.130863 | 11.78605 | 4.042547 |
| 5.173198 | 48.21972 | 4.407341 |  | 2.686619 | 3.419461 | 33.80315 | 3.454718 |
| 4.515474 | 7.688878 | 3.892681 |  | 1.795628 | 9.605543 | 8.6903 | 6.620519 |
| 3.428305 | 2.580477 | 8.34601 |  | 2.554193 | 4.342253 | 10.38427 | 2.837401 |
| 21.66125 | 2.961843 | 18.72325 |  | 7.295734 | 4.54894 | 11.54189 | 6.180138 |
| 3.479407 | 7.765695 | 35.50417 |  | 6.912 | 99.3321 | 20.04875 | 7.018486 |
| 13.76061 | 6.994268 | 3.091796 |  | 5.748478 | 5.179369 | 4.093789 | 3.859199 |
| 4.9379 | 3.797914 | 8.240883 |  | 11.98452 | 7.955498 | 58.14246 | 5.294112 |
| 3.42004 | 9.033349 | 61.18681 |  | 7.018988 | 3.308934 | 28.536 | 4.92016 |
| 4.568493 | 5.271915 | 9.757773 |  | 12.03821 | 2.209904 | 21.74566 | 3.327905 |
| 3.025389 | 13.24397 | 21.29475 |  | 2.890218 | 12.99545 | 4.756 | 2.883142 |
| 9.906774 | 8.418025 | 17.25387 |  | 2.996777 | 3.940124 | 30.06599 | 3.491158 |
| 5.603872 | 4.226585 | 4.718218 |  | 15.25033 | 5.692883 | 3.100139 | 20.76682 |
| 5.686895 | 2.416046 | 8.45488 |  | 29.55895 | 4.334985 | 3.287922 | 5.03431 |
| 5.800248 | 7.819135 | 4.313939 |  | 4.88741 | 36.88445 | 12.69055 | 3.48911 |
| 3.618303 | 3.310627 | 31.75673 |  | 22.64 | 8.054515 | 16.46848 | 5.477624 |
| 2.77543 | 2.955251 | 6.357782 |  | 8.218758 | 3.82033 | 8.492529 | 15.75688 |
| 10.07808 | 0.06896158 | 3.824291 |  | 4.535957 | 5.193346 | 33.292 | 31.30639 |
| 1.678691 | 3.804 | 12.14396 |  | 4.582239 | 3.080328 | 9.820777 | 3.397991 |
| 2.919163 | 6.625786 | 12.36095 |  | 3.250575 | 27.22892 | 25.70532 | 3.06565 |
| 4.021283 | 18.20369 | 8.371486 |  | 3.557401 | 21.85021 | 20.48627 | 3.936523 |
| 6.82319 | 6.242353 | 7.517094 |  | 3.439445 | 3.45056 | 11.6506 | 5.901929 |
| 5.926712 | 6.218085 | 3.567012 |  | 2.433551 | 2.757213 | 5.100664 | 4.60055 |
| 3.183608 | 5.494723 | 5.755218 |  | 5.484277 | 2.698984 | 10.10359 | 4.219428 |
| 6.849532 | 3.259607 | 3.392108 |  | 5.677748 | 2.762234 | 14.21677 | 2.259138 |
| 8.014784 | 4.68135 | 7.211825 |  | 2.551856 | 1.877052 | 11.74525 | 3.322243 |
| 6.236865 | 3.786395 | 68.05651 |  | 4.362887 | 12.54328 | 19.25635 | 9.84432 |
| 5.915313 | 3.38498 | 15.99547 |  | 9.759302 | 6.913745 | 2.935901 | 3.552233 |
| 4.054087 | 35.12024 | 9.370538 |  | 3.193519 | 5.773662 | 7.461199 | 69.22868 |
| 3.61472 | 5.122873 | 21.65502 |  | 3.325091 | 6.248808 | 5.624152 | 2.356025 |
| 44.6707 | 9.285817 | 18.21679 |  | 15.37976 | 24.72051 | 28.11498 | 3.765311 |
| 3.446631 | 3.332436 | 14.95801 |  | 5.277694 | 2.042354 | 15.40432 | 4.533886 |
| 11.05151 | 4.081714 | 4.623738 |  | 2.238747 | 4.601288 | 5.468887 | 2.002125 |
| 35.53352 | 4.365991 | 7.794639 |  | 3.668824 | 3.652273 | 11.29071 | 14.00579 |
| 3.732332 | 5.738931 | 25.39615 |  | 9.628066 | 5.243873 | 6.650562 | 3.605207 |
| 7.183074 | 13.2006 | 3.924173 |  | 6.263067 | 3.501524 | 22.85333 | 3.594844 |
| 3.599945 | 9.432605 | 7.407055 |  | 7.98 | 5.845437 | 11.34255 | 4.658071 |
| 9.253977 | 8.499096 | 3.718872 |  | 9.938398 | 6.42453 | 11.23647 | 3.504675 |
| 13.26536 | 9.263871 | 5.968917 |  | 5.828238 | 6.53886 | 3.263785 | 4.017454 |
| 11.26065 | 15.00619 | 2.046474 |  | 4.865594 | 6.309383 | 9.151642 | 3.765775 |
| 3.331935 | 24.23985 | 5.611487 |  | 1.931879 | 3.941832 | 4.317423 | 6.138993 |
| 4.055642 | 4.740174 | 3.450339 |  | 5.428665 | 2.552446 | 13.65374 | 9.240981 |
| 7.203678 | 4.32512 | 5.874695 |  | 2.728605 | 6.036601 | 4.093367 | 5.181482 |
| 3.149247 | 9.194871 | 4.207989 |  | 14.74163 | 12.80383 | 56.48565 | 3.94523 |
| 6.244378 | 7.003255 | 8.792313 |  | 2.660927 | 4.184665 | 7.363136 | 7.279443 |
| 5.813245 | 19.14715 | 13.20237 |  | 3.448 | 16.78974 | 8.582711 | 7.290617 |
| 6.413207 | 11.30115 | 9.386143 |  | 3.345788 | 3.228724 | 14.0195 | 15.0576 |
| 4.45083 | 5.163513 | 3.541927 |  | 7.121679 | 2.257897 | 3.681243 | 13.97177 |
| 14.96353 | 30.36225 | 4.144959 |  | 5.143608 | 2.757744 | 10.356 | 4.226306 |
| 5.780738 | 1.510114 | 62.34666 |  | 57.42834 | 4.842371 | 5.618184 | 7.143871 |
| 2.839212 | 48.98288 | 5.995182 |  | 5.017775 | 2.840426 | 4.038611 | 3.198878 |
| 6.540807 | 5.626059 | 38.98681 |  | 0 | 3.289063 | 4.724098 | 3.001504 |
| 6.465089 | 26.23422 | 5.792067 |  | 6.912 | 3.809124 | 4.14787 | 3.663801 |
| 4.702223 | 4.372487 | 20.37074 |  | 3.963579 | 2.462851 | 3.301596 | 10.17875 |
| 3.767851 | 20.59423 | 6.270476 |  | 12.17701 | 10.01387 | 3.00911 | 5.079105 |
| 3.498233 | 3.244913 | 8.483398 |  | 2.220232 | 3.256711 | 3.347509 | 10.16876 |
| 2.695791 | 3.479226 | 29.24791 |  | 2.617632 | 3.109075 | 3.199267 | 14.81446 |
| 7.207214 | 10.21576 | 2.759224 |  | 6.912 | 3.099916 | 3.093 | 2.926863 |
| 5.993523 | 54.54324 | 4.939817 |  | 14.29862 | 7.640582 | 10.23245 | 2.441629 |
| 5.70216 | 2.793361 | 6.455538 |  | 8.3424 | 8.23277 | 2.256181 | 3.497032 |
| 15.56455 | 4.438578 | 22.02907 |  | 63.55521 | 2.483061 | 20.21056 | 3.636445 |
| 2.312072 | 5.197358 | 10.04154 |  | 3.89312 | 3.545739 | 5.117818 | 14.8106 |
| 3.247093 | 5.640617 | 10.22615 |  | 3.22841 | 3.062068 | 7.814362 | 13.32674 |
| 5.025277 | 8.572352 | 3.297222 |  | 2.727866 | 4.031626 | 3.229538 | 33.21849 |
| 4.691738 | 2.320787 | 10.15771 |  | 4.8664 | 2.776437 | 14.38093 | 1.896965 |
| 3.991433 | 4.378583 | 22.88596 |  | 6.792 | 5.903855 | 3.147649 | 7.219463 |
| 3.487693 | 26.95292 | 5.956315 |  | 20.664 | 3.730513 | 8.245511 | 4.722518 |
| 3.487693 | 6.2502 | 5.445234 |  | 9.825853 | 6.524784 | 4.082891 | 3.01802 |
| 7.810401 | 17.8216 | 2.821653 |  | 1.936576 | 4.998258 | 4.883058 | 16.8251 |
| 3.170651 | 3.836102 | 2.882238 |  | 3.321405 | 8.480976 | 2.955067 | 85.79481 |
| 3.432247 | 2.16494 | 16.75027 |  | 3.2 | 4.166406 | 1.631502 | 3.139169 |
| 10.81465 | 24.0111 | 4.520616 |  | 11.36352 | 5.461991 | 11.32264 | 2.680095 |
| 3.342091 | 3.657393 | 7.72842 |  | 4.788 | 3.858701 | 4.943254 | 8.872213 |
| 4.152256 | 4.909312 | 6.591961 |  | 2.738304 | 6.599034 | 4.769373 | 13.88609 |
| 3.821898 | 11.06408 | 5.131357 |  | 10.13336 | 6.748168 | 9.248603 | 14.13995 |
| 4.391503 | 9.38594 | 17.05534 |  | 3.694936 | 2.954294 | 8.585351 | 5.046254 |
| 4.084431 | 10.04965 | 5.103626 |  | 20.29995 | 3.679782 | 31.37928 | 4.606511 |
| 2.977895 | 7.487657 | 26.896 |  | 19.2 | 2.102921 | 7.449515 | 11.51684 |
| 2.820548 | 3.233633 | 1.758303 |  | 3.358026 | 4.70114 | 8.070379 | 5.447849 |
| 3.712028 | 3.574306 | 5.842261 |  | 1.69816 | 8.314847 | 9.660952 | 4.599756 |
| 7.50415 | 13.2102 | 4.882935 |  | 34.1977 | 5.979857 | 3.632943 | 5.399806 |
| 6.87218 | 3.184704 | 3.320371 |  | 8 | 3.099925 | 2.486411 | 3.383637 |
| 3.672481 | 21.86256 | 1.437197 |  | 1.573825 | 10.20326 | 3.955693 | 11.02317 |
| 6.511665 | 4.269762 | 7.282415 |  | 2.720311 | 8.441741 | 5.863921 | 22.06749 |
| 4.532604 | 13.77683 | 5.035426 |  | 10.344 | 3.715209 | 5.486964 | 3.494808 |
| 7.817123 | 28.97195 | 3.881126 |  | 9.260092 | 4.256725 | 3.529137 | 2.330608 |
| 5.429508 | 8.4341 | 6.44968 |  | 3.786906 | 4.969838 | 5.382365 | 9.820965 |
| 4.515573 | 8.40109 | 14.93165 |  | 2.085686 | 3.206274 | 4.352672 | 9.660328 |
| 4.707357 | 9.121158 | 3.084029 |  | 5.665702 | 9.179277 | 4.502095 | 3.004342 |
| 7.597175 | 67.95726 | 24.08 |  | 7.598702 | 2.419576 | 8.471011 | 3.993809 |
| 5.843291 | 9.657982 | 6.404175 |  | 5.478673 | 2.294947 | 10.46902 | 3.146001 |
| 41.48041 | 11.53415 | 4.434602 |  | 2.580285 | 5.743254 | 3.292712 | 9.721667 |
| 9.49891 | 20.74688 | 15.30805 |  | 6.466073 | 5.00383 | 5.233418 | 4.580411 |
| 3.750686 | 5.059209 | 6.664538 |  | 3.521565 | 5.341352 | 6.421883 | 4.581104 |
| 5.540888 | 8.59558 | 6.730703 |  | 12.61813 | 3.75486 | 4.230835 | 3.983544 |
| 2.905558 | 21.73782 | 7.240685 |  | 6.180536 | 16.11625 | 12.3656 | 4.839196 |
| 3.353715 | 5.911393 | 5.146069 |  | 8.517152 | 29.95249 | 14.21681 | 3.158312 |
| 6.176332 | 3.537694 | 3.865368 |  | 3.899792 | 8.696461 | 40.25879 | 5.883948 |
| 5.73714 | 8.930378 | 1.915428 |  | 8.175017 | 5.395777 | 16.91241 | 13.47307 |
| 44.22567 | 2.719483 | 5.232976 |  | 2.844584 | 5.596581 | 3.529137 | 3.374752 |
| 20.73592 | 4.622013 | 17.21086 |  | 5.494823 | 3.999548 | 5.382365 | 2.98784 |
| 6.012979 | 33.37043 | 5.55772 |  | 32.82863 | 4.377224 | 4.352672 | 5.75979 |
| 4.79398 | 3.801408 | 6.174628 |  | 4.889044 | 3.848172 | 4.502095 | 65.73899 |
| 5.500957 | 2.953743 | 11.58953 |  | 3.80153 | 3.777669 | 8.471011 | 3.05286 |
| 3.512461 | 28.35477 | 4.588564 |  | 12.81379 | 3.058585 | 10.46902 | 3.898861 |
| 5.825376 | 59.03278 | 15.58979 |  | 2.806319 | 5.410905 | 3.292712 | 5.640461 |
| 4.657805 | 8.256578 | 4.410421 |  | 1.741472 | 2.004197 | 5.233418 | 20.33318 |
| 31.83797 | 6.599083 | 5.794946 |  | 5.1072 | 6.676559 | 6.421883 | 4.789275 |
| 3.906773 | 3.02982 | 2.833885 |  | 2.839118 | 4.028359 | 4.230835 | 5.060061 |
| 46.73039 | 2.436074 | 10.58551 |  | 5.956603 | 104.1552 | 12.3656 | 5.894311 |
| 21.33045 | 18.10152 | 2.367634 |  | 13.7556 | 9.219864 | 14.21681 | 6.961161 |
| 3.033509 | 1.596452 | 3.633836 |  | 3.358026 | 9.669833 | 40.25879 | 4.211712 |
| 3.489828 | 3.302853 | 2.435094 |  | 4.557071 | 5.897705 | 16.91241 | 24.83911 |
| 6.771817 | 3.440039 | 19.06842 |  | 5.473796 | 7.132749 | 10.71997 | 2.48833 |
| 4.962059 | 23.95521 | 17.28538 |  | 5.528831 | 2.329915 | 27.30263 | 5.81553 |
| 4.137944 | 6.453716 | 6.19425 |  | 3.26451 | 2.999642 | 3.383904 | 42.31872 |
| 4.456973 | 4.12682 | 7.206724 |  | 4.393434 | 7.495128 | 2.42009 | 4.004118 |
| 13.15864 | 10.0864 | 9.946363 |  | 6.092135 | 3.907637 | 1.835944 | 3.190219 |
| 6.912034 | 4.472702 | 3.565188 |  | 24.108 | 6.868262 | 3.551778 | 4.548924 |
| 4.04625 | 3.033129 | 11.13893 |  | 6.037441 | 9.823902 | 17.61869 | 16.96276 |
| 5.632274 | 3.544584 | 5.406868 |  | 24.6269 | 4.323649 | 5.074654 | 15.32843 |
| 13.27835 | 24.849 | 4.377415 |  | 7.740785 | 2.922254 | 3.290667 | 8.888773 |
| 10.65645 | 3.089352 | 2.83542 |  | 0.0414996 | 2.219779 | 13.09328 | 12.72149 |
| 11.65532 | 12.99426 | 21.02284 |  | 10.0224 | 7.415084 | 11.46244 | 5.446975 |
| 5.677135 | 3.5169 | 4.637545 |  | 7.063283 | 4.180349 | 1.736524 | 3.30026 |
| 10.86407 | 6.401118 | 6.606395 |  | 3.203008 | 4.224028 | 6.036556 | 8.008796 |
| 4.939879 | 7.526647 | 10.74405 |  | 2.67944 | 4.315119 | 8.042381 | 3.639672 |
| 3.717939 | 4.816011 | 16.56602 |  | 1.902183 | 15.60447 | 5.722603 | 1.909682 |
| 4.019172 | 3.046661 | 8.765564 |  | 2.29982 | 3.964774 | 7.86734 | 3.462503 |
| 10.67367 | 3.695439 | 7.628095 |  | 5.8274 | 6.495729 | 37.58011 | 8.744313 |
| 5.608814 | 33.98009 | 2.149609 |  | 2.868628 | 4.047919 | 3.779006 | 8.58518 |
| 8.034668 | 3.206429 | 7.443069 |  | 4.1328 | 4.18549 | 4.650233 | 6.917371 |
| 4.674448 | 7.856254 | 3.7811 |  | 6.812217 | 6.016367 | 3.66892 | 6.099515 |
| 2.933528 | 3.958003 | 2.100406 |  | 2.589866 | 6.715923 | 33.17717 | 11.40873 |
| 3.779349 | 1.740294 | 13.81498 |  | 23.66896 | 2.689822 | 3.452 | 9.044627 |
| 9.951559 | 65.5142 | 5.524758 |  | 2.048273 | 12.30285 | 8.770119 | 2.459537 |
| 8.637225 | 12.74515 | 3.031031 |  | 2.018917 | 5.080617 | 2.605158 | 3.949497 |
| 5.29186 | 3.337525 | 3.014808 |  | 8.527147 | 4.999639 | 80.8926 | 8.416184 |
| 10.84915 | 4.352321 | 5.272357 |  | 2.887135 | 3.700237 | 17.10024 | 23.0803 |
| 3.662755 | 3.830362 | 2.637599 |  | 4.102511 |  | 18.85584 | 7.552681 |
| 5.166384 | 3.413924 | 2.641214 |  | 2.046643 |  | 7.9396 | 3.702324 |
| 9.280945 | 6.268585 | 4.012137 |  | 5.241838 |  | 2.706046 | 16.44361 |
| 5.926085 | 5.011176 | 5.111076 |  | 3.273678 |  | 3.452 | 3.949227 |
| 4.387254 | 3.95559 | 9.561972 |  | 2.982124 |  | 7.061175 | 5.196734 |
| 3.798937 | 4.547525 | 6.73466 |  | 7.840778 |  | 2.822476 | 4.622112 |
| 5.978378 | 9.287487 | 4.135839 |  | 10.344 |  | 55.48829 | 4.979665 |
| 8.28274 | 3.811497 | 15.21087 |  | 24.108 |  | 3.806 | 34.28492 |
| 2.665828 | 19.78808 | 11.13072 |  | 6.037441 |  | 2.498107 | 8.621267 |
| 5.471937 | 105.2322 | 3.421441 |  | 24.6269 |  | 2.678596 | 40.18476 |
| 5.545042 | 3.696376 | 3.797429 |  | 7.740785 |  | 4.818702 | 7.671071 |
| 6.895618 | 15.16089 | 3.545544 |  | 0.0414996 |  | 11.15002 | 12.25442 |
| 5.949197 | 4.719179 | 2.952581 |  | 10.0224 |  | 6.422252 | 5.37814 |
| 9.80904 | 4.925943 | 9.969897 |  | 7.063283 |  | 8.614656 | 7.773623 |
| 7.01393 | 3.746885 | 3.306368 |  | 3.203008 |  | 7.153411 | 12.51454 |
| 6.770592 | 11.05082 | 2.81161 |  | 2.67944 |  | 10.368 | 3.824953 |
| 4.143693 | 3.230436 | 12.32643 |  | 1.902183 |  | 32.42147 | 3.723294 |
| 8.106226 | 36.719 | 3.948617 |  | 2.29982 |  | 6.459958 | 5.773077 |
| 5.371335 | 3.480779 | 4.608798 |  | 5.8274 |  | 4.528 | 0.1051267 |
| 15.86675 | 5.210356 | 6.708756 |  | 2.868628 |  | 5.26073 | 2.900086 |
| 5.66953 | 35.25269 | 9.979081 |  | 4.1328 |  | 7.823114 | 3.073608 |
| 5.157721 | 16.49554 | 9.744092 |  | 6.812217 |  | 31.72277 | 4.616375 |
| 9.432612 | 4.258542 | 32.19768 |  | 2.589866 |  | 3.779006 | 4.614352 |
| 6.241935 | 17.13514 | 3.526099 |  | 23.66896 |  | 4.529159 | 2.910048 |
| 16.07511 | 3.525617 | 0.02596993 |  | 2.048273 |  | 6.989027 | 7.157727 |
| 5.785264 | 5.054423 | 2.263325 |  | 2.018917 |  | 13.62109 | 4.590946 |
| 3.835927 | 6.865592 | 8.641303 |  | 8.527147 |  | 10.36083 | 1.508782 |
| 5.578598 | 66.89509 | 4.111251 |  | 2.887135 |  | 2.234349 | 8.053018 |
| 4.149332 | 2.209742 | 3.669015 |  | 4.102511 |  | 3.03809 | 2.971951 |
| 25.07848 | 8.412565 | 1.181272 |  | 2.046643 |  | 41.424 | 4.819678 |
| 6.953586 | 50.43511 | 2.570337 |  | 5.241838 |  | 2.010795 | 6.828183 |
| 3.994372 | 4.810392 | 5.423892 |  | 3.273678 |  | 2.199527 | 3.369159 |
| 4.080151 | 4.95268 | 6.33233 |  | 2.982124 |  | 10.70601 | 4.036311 |
| 5.196534 | 3.268468 | 1.994472 |  | 7.840778 |  | 3.193554 | 3.787527 |
| 9.093566 | 8.954486 | 1.934708 |  | 10.344 |  | 5.140612 | 3.187546 |
| 8.520721 | 34.90224 | 3.419422 |  |  |  | 2.561346 | 3.198087 |
| 10.09129 | 6.442234 | 6.467288 |  |  |  |  | 2.760085 |
| 5.369148 | 8.049937 | 4.179129 |  |  |  |  | 5.634952 |
| 3.899227 | 43.74414 | 9.282991 |  |  |  |  | 7.473245 |
| 3.288833 | 43.96271 | 3.742463 |  |  |  |  | 65.86035 |
| 15.13825 | 9.21599 | 7.092382 |  |  |  |  | 5.369213 |
| 4.269036 | 52.79644 | 19.15493 |  |  |  |  | 7.654714 |
| 6.269823 | 2.981945 | 7.64037 |  |  |  |  | 2.387604 |
| 8.000979 | 14.85962 | 4.004523 |  |  |  |  | 2.915202 |
| 3.917735 | 18.82707 | 18.34915 |  |  |  |  | 2.948668 |
| 6.265456 | 44.46743 | 15.52836 |  |  |  |  | 4.757938 |
| 4.52019 | 2.99935 | 5.207326 |  |  |  |  | 7.169907 |
| 3.988838 | 41.77664 | 4.086136 |  |  |  |  | 9.537182 |
| 6.350046 | 3.854384 | 14.72635 |  |  |  |  | 17.62941 |
| 89.96603 | 6.538677 | 7.655752 |  |  |  |  | 5.283644 |
| 5.070765 | 3.816117 | 12.37389 |  |  |  |  | 14.95197 |
| 5.163982 | 5.046127 | 2.583326 |  |  |  |  | 2.910084 |
| 4.570003 | 17.42406 | 2.768072 |  |  |  |  | 4.033962 |
| 6.357672 | 2.85226 | 7.930387 |  |  |  |  | 4.03737 |
| 13.92427 | 2.92881 | 8.1 |  |  |  |  | 4.248317 |
| 11.85363 | 23.07815 | 6.0648 |  |  |  |  | 3.541577 |
| 7.017491 | 4.189727 | 32.20329 |  |  |  |  | 3.843687 |
| 8.189673 | 3.246032 | 13.83882 |  |  |  |  | 2.392049 |
| 4.297643 | 5.356739 | 7.199009 |  |  |  |  |  |
| 3.127875 | 4.994994 | 5.775777 |  |  |  |  |  |
| 3.757701 | 5.022386 | 5.255344 |  |  |  |  |  |
| 4.590179 | 6.898836 | 28.88483 |  |  |  |  |  |
| 4.75446 | 15.25591 | 12.236 |  |  |  |  |  |
| 3.59537 | 10.37277 | 7.255353 |  |  |  |  |  |
| 8.617045 | 3.086566 | 15.86093 |  |  |  |  |  |
| 5.078771 | 11.90281 | 3.795037 |  |  |  |  |  |
| 3.887242 | 8.636878 | 20.92921 |  |  |  |  |  |
| 4.366198 | 12.5352 | 3.733803 |  |  |  |  |  |
| 3.134444 | 7.364563 | 8.550655 |  |  |  |  |  |
| 29.83677 | 22.98108 | 64.45506 |  |  |  |  |  |
| 8.882443 | 4.588978 | 41.0285 |  |  |  |  |  |
| 5.52092 | 5.985979 | 20.21274 |  |  |  |  |  |
| 3.265674 | 11.40329 | 6.24144 |  |  |  |  |  |
| 7.111586 | 6.009433 | 7.029541 |  |  |  |  |  |
| 7.370396 | 17.99706 | 4.161457 |  |  |  |  |  |
| 3.504621 | 1.809898 | 25.90572 |  |  |  |  |  |
| 5.996718 | 18.39081 | 21.35663 |  |  |  |  |  |
| 8.808705 | 5.590884 | 4.764867 |  |  |  |  |  |
| 3.983127 | 1.932985 | 21.22981 |  |  |  |  |  |
| 9.837594 | 51.66588 | 7.572389 |  |  |  |  |  |
| 4.638844 | 1.218729 | 3.866492 |  |  |  |  |  |
| 12.03892 | 36.4704 | 4.616856 |  |  |  |  |  |
| 15.20701 | 6.013546 | 5.749688 |  |  |  |  |  |
| 7.209854 | 23.42511 | 29.68288 |  |  |  |  |  |
| 6.443994 | 16.52482 | 12.73736 |  |  |  |  |  |
| 3.950952 | 10.99585 | 13.15228 |  |  |  |  |  |
| 9.583839 | 7.183992 | 13.3 |  |  |  |  |  |
| 4.4227 | 10.70138 | 4.424279 |  |  |  |  |  |
| 4.518932 | 3.852371 | 5.546093 |  |  |  |  |  |
| 5.057417 | 4.725013 | 6.509099 |  |  |  |  |  |
| 4.890999 | 2.368429 | 19.71285 |  |  |  |  |  |
| 7.459964 | 22.42177 | 4.287576 |  |  |  |  |  |
| 14.57091 | 1.79485 | 18.48471 |  |  |  |  |  |
| 44.56948 | 11.11173 | 6.990124 |  |  |  |  |  |
| 5.784246 | 24.35147 | 10.08475 |  |  |  |  |  |
| 5.884908 | 20.00527 | 2.758242 |  |  |  |  |  |
| 13.49087 | 3.910952 | 4.004 |  |  |  |  |  |
| 5.404862 | 61.48747 | 5.686054 |  |  |  |  |  |
| 4.800424 | 3.179179 | 19.2192 |  |  |  |  |  |
| 3.568101 | 2.046374 | 21.8032 |  |  |  |  |  |
| 3.282156 | 4.090693 | 37.4794 |  |  |  |  |  |
| 14.96267 | 2.438234 | 26.5142 |  |  |  |  |  |
| 13.72325 | 18.75704 | 11.6208 |  |  |  |  |  |
| 75.46032 | 2.764293 | 7.627536 |  |  |  |  |  |
| 5.606713 | 3.713653 | 6.639006 |  |  |  |  |  |
| 2.944464 | 7.488418 | 3.597325 |  |  |  |  |  |
| 12.51294 | 18.07654 | 7.097958 |  |  |  |  |  |
| 43.40393 | 15.50631 | 4.786731 |  |  |  |  |  |
| 13.2431 | 7.180667 | 8.620864 |  |  |  |  |  |
| 7.254133 | 3.266517 | 15.36335 |  |  |  |  |  |
| 13.31503 | 5.851517 | 3.991398 |  |  |  |  |  |
| 3.058811 | 4.318466 | 4.347585 |  |  |  |  |  |
| 7.519434 | 2.372515 | 41.12809 |  |  |  |  |  |
| 14.23121 | 6.084192 | 11.128 |  |  |  |  |  |
| 3.712251 | 49.07799 | 18.74812 |  |  |  |  |  |
| 12.91703 | 6.818691 | 15.3626 |  |  |  |  |  |
| 6.579211 | 3.457648 | 6.290041 |  |  |  |  |  |
| 4.464413 | 60.57079 | 10.68853 |  |  |  |  |  |
| 7.042887 | 112.0916 | 9.072204 |  |  |  |  |  |
| 28.88652 | 16.87013 | 3.991815 |  |  |  |  |  |
| 5.767667 | 73.18973 | 50.2711 |  |  |  |  |  |
| 8.38583 | 5.423379 | 7.371676 |  |  |  |  |  |
| 5.760418 | 5.370831 | 3.28631 |  |  |  |  |  |
| 5.296046 | 3.643135 | 2.955358 |  |  |  |  |  |
| 4.910099 | 42.48977 | 6.762891 |  |  |  |  |  |
| 6.640303 | 4.777934 | 9.94489 |  |  |  |  |  |
| 5.18449 | 21.25561 | 129.189 |  |  |  |  |  |
| 7.328394 | 3.644065 | 8.346 |  |  |  |  |  |
| 4.740923 | 10.25739 | 74.96657 |  |  |  |  |  |
| 18.67084 | 4.159095 | 3.410299 |  |  |  |  |  |
| 3.727305 | 30.32317 | 98.44972 |  |  |  |  |  |
| 9.884307 | 9.0036 | 5.0076 |  |  |  |  |  |
| 7.249674 | 20.88169 | 60.97582 |  |  |  |  |  |
| 3.363778 | 2.854107 | 7.665493 |  |  |  |  |  |
| 16.1199 | 4.582346 | 4.33634 |  |  |  |  |  |
| 20.27212 | 13.36474 | 6.486368 |  |  |  |  |  |
| 6.927127 | 4.040388 | 4.580742 |  |  |  |  |  |
| 8.360592 | 4.805536 | 6.240781 |  |  |  |  |  |
| 5.705536 | 9.747685 | 15.60663 |  |  |  |  |  |
| 3.146782 | 5.345635 | 14.141 |  |  |  |  |  |
| 3.146782 | 1.33924 | 4.070293 |  |  |  |  |  |
| 7.818881 | 32.57814 | 9.048935 |  |  |  |  |  |
| 12.69541 | 15.89354 | 3.626447 |  |  |  |  |  |
| 53.58749 | 3.380137 | 3.085549 |  |  |  |  |  |
| 17.83091 | 49.14698 | 3.281679 |  |  |  |  |  |
| 18.40113 | 9.193369 | 6.424884 |  |  |  |  |  |
| 8.768107 | 3.189772 | 38.23961 |  |  |  |  |  |
| 63.81644 | 17.23838 | 49.456 |  |  |  |  |  |
| 7.781814 | 10.61583 | 49.456 |  |  |  |  |  |
| 5.997297 | 5.004201 | 28.4372 |  |  |  |  |  |
| 56.5883 | 36.44016 | 20.62954 |  |  |  |  |  |
| 8.332534 | 12.6616 | 10.78079 |  |  |  |  |  |
| 6.264312 | 23.26643 | 68.79215 |  |  |  |  |  |
| 9.456793 | 3.522317 | 50.60307 |  |  |  |  |  |
| 24.19925 | 3.817634 | 3.100131 |  |  |  |  |  |
| 4.554161 | 26.40362 | 5.103691 |  |  |  |  |  |
| 15.14759 | 38.98462 | 4.8347 |  |  |  |  |  |
| 7.498356 | 3.950313 | 5.606859 |  |  |  |  |  |
| 6.993415 | 3.631544 | 3.903974 |  |  |  |  |  |
| 6.178982 | 12.14231 | 6.7132 |  |  |  |  |  |
| 21.94665 | 3.403439 | 10.70665 |  |  |  |  |  |
| 3.19818 | 5.99777 | 71.2632 |  |  |  |  |  |
| 6.429802 | 7.041374 | 86.2388 |  |  |  |  |  |
| 4.577478 | 12.46714 | 22.88379 |  |  |  |  |  |
| 3.720988 | 9.486527 | 5.926877 |  |  |  |  |  |
| 5.104885 | 17.31706 | 11.3608 |  |  |  |  |  |
| 61.44449 | 35.23549 | 6.485462 |  |  |  |  |  |
| 15.10847 | 19.22031 | 16.95622 |  |  |  |  |  |
| 3.616411 | 4.713458 | 3.607636 |  |  |  |  |  |
| 16.98287 | 3.412507 | 8.2624 |  |  |  |  |  |
| 12.02279 | 3.821676 | 8.073169 |  |  |  |  |  |
| 9.355913 | 4.037948 | 16.17388 |  |  |  |  |  |
| 32.14264 | 5.515693 | 30.43753 |  |  |  |  |  |
| 4.39899 | 7.198881 | 13.59631 |  |  |  |  |  |
| 11.02491 | 7.040839 | 48.3608 |  |  |  |  |  |
| 51.69981 | 3.931173 | 5.093469 |  |  |  |  |  |
| 9.571022 | 2.886713 | 17.05418 |  |  |  |  |  |
| 7.47169 | 2.824367 | 25.21568 |  |  |  |  |  |
| 18.20335 | 4.984062 | 58.38 |  |  |  |  |  |
| 54.92893 | 33.83293 | 20.43105 |  |  |  |  |  |
| 3.625744 | 12.11301 | 17.87284 |  |  |  |  |  |
| 16.60115 | 7.430006 | 7.85733 |  |  |  |  |  |
| 5.40742 | 6.523908 | 76.6724 |  |  |  |  |  |
| 16.89458 | 89.66293 | 12.8436 |  |  |  |  |  |
| 4.520723 | 12.86937 | 5.928894 |  |  |  |  |  |
| 6.123772 | 5.435569 | 4.6704 |  |  |  |  |  |
| 7.139361 | 4.278169 | 5.332863 |  |  |  |  |  |
| 8.586899 | 4.402699 | 15.71512 |  |  |  |  |  |
| 2.900073 | 8.368858 | 4.116452 |  |  |  |  |  |
| 2.864314 | 35.29963 | 4.065777 |  |  |  |  |  |
| 3.941006 | 7.580554 | 3.34918 |  |  |  |  |  |
| 49.96193 | 35.70654 | 3.113218 |  |  |  |  |  |
| 37.64208 | 52.51822 | 4.069336 |  |  |  |  |  |
| 13.48265 | 2.321559 | 7.772616 |  |  |  |  |  |
| 17.03128 | 13.88149 | 3.418872 |  |  |  |  |  |
| 5.941634 | 8.026715 | 9.992296 |  |  |  |  |  |
| 4.480546 | 5.65508 | 2.088464 |  |  |  |  |  |
| 4.577491 | 24.04581 | 3.289236 |  |  |  |  |  |
| 7.778326 | 9.81054 | 20.99034 |  |  |  |  |  |
| 3.364701 | 75.95212 | 22.06056 |  |  |  |  |  |
| 3.622392 | 5.472573 | 9.043131 |  |  |  |  |  |
| 5.240562 | 2.291274 | 13.42363 |  |  |  |  |  |
| 0.09522608 | 2.736462 | 41.08545 |  |  |  |  |  |
| 4.901187 | 0.00341578 | 25.40248 |  |  |  |  |  |
| 5.63366 | 13.27623 | 12.66254 |  |  |  |  |  |
| 27.40097 | 3.405749 | 3.886573 |  |  |  |  |  |
| 19.52753 | 7.051288 | 4.522 |  |  |  |  |  |
| 11.711 | 7.83123 | 7.939446 |  |  |  |  |  |
| 39.12138 | 5.611701 | 10.64 |  |  |  |  |  |
| 57.75461 | 3.156962 | 13.3 |  |  |  |  |  |
| 7.923483 | 5.484257 | 6.735438 |  |  |  |  |  |
| 4.151923 | 24.32199 | 10.64 |  |  |  |  |  |
| 26.62657 | 32.49173 | 5.722635 |  |  |  |  |  |
| 4.427084 | 3.554024 | 4.119083 |  |  |  |  |  |
| 38.8207 | 11.42194 | 26.64891 |  |  |  |  |  |
| 50.43554 | 8.899591 | 6.8068 |  |  |  |  |  |
| 4.817661 | 101.3045 | 12.012 |  |  |  |  |  |
| 7.776881 | 2.906672 | 13.14185 |  |  |  |  |  |
| 3.870275 | 21.35118 | 12.012 |  |  |  |  |  |
| 4.263403 | 39.39407 | 26.34067 |  |  |  |  |  |
| 4.544561 | 3.723146 | 7.1766 |  |  |  |  |  |
| 7.397459 | 4.333514 | 18.67493 |  |  |  |  |  |
| 11.6776 | 8.387139 | 17.2484 |  |  |  |  |  |
| 15.92384 | 10.43347 | 11.128 |  |  |  |  |  |
| 2.904337 | 5.513686 | 66.05254 |  |  |  |  |  |
| 5.378879 | 96.83927 | 29.2672 |  |  |  |  |  |
| 23.66534 | 3.474241 | 10.21198 |  |  |  |  |  |
| 8.505718 | 3.030232 | 17.50273 |  |  |  |  |  |
| 15.83019 | 21.30377 | 9.4588 |  |  |  |  |  |
| 16.47956 | 2.722892 | 27.82 |  |  |  |  |  |
| 8.765643 | 12.30848 | 8.346 |  |  |  |  |  |
| 8.505023 | 6.117682 | 4.594518 |  |  |  |  |  |
| 2.37129 | 5.574274 | 11.128 |  |  |  |  |  |
| 7.366918 | 34.37192 | 19.92954 |  |  |  |  |  |
| 3.934615 | 5.59799 | 10.70946 |  |  |  |  |  |
| 12.19393 | 9.655872 | 12.92331 |  |  |  |  |  |
| 4.991903 | 5.459456 | 5.514114 |  |  |  |  |  |
| 7.564808 | 11.01609 | 12.2408 |  |  |  |  |  |
| 5.317897 | 3.887494 | 4.760377 |  |  |  |  |  |
| 57.41393 | 3.490295 | 31.48128 |  |  |  |  |  |
| 3.187746 | 16.71385 | 19.1068 |  |  |  |  |  |
| 2.266253 | 2.971632 | 3.56573 |  |  |  |  |  |
| 3.553876 | 3.820504 | 97.84954 |  |  |  |  |  |
| 3.166313 | 2.844502 | 13.04477 |  |  |  |  |  |
| 3.596329 | 6.95837 | 15.39263 |  |  |  |  |  |
| 5.251566 | 7.177802 | 15.38806 |  |  |  |  |  |
| 3.170763 | 3.038501 | 14.39648 |  |  |  |  |  |
| 3.04831 | 89.89688 | 7.049325 |  |  |  |  |  |
| 5.088977 | 8.969983 | 5.745909 |  |  |  |  |  |
| 7.923492 | 27.67023 | 2.896732 |  |  |  |  |  |
| 38.28894 | 21.00619 | 12.6638 |  |  |  |  |  |
| 28.47043 | 15.25947 | 13.3 |  |  |  |  |  |
| 11.07071 | 3.758587 | 12.16622 |  |  |  |  |  |
| 11.00985 | 3.493297 | 4.601426 |  |  |  |  |  |
| 13.6857 | 3.039788 | 41.25039 |  |  |  |  |  |
| 6.098674 | 2.912032 | 4.707246 |  |  |  |  |  |
| 3.459802 | 11.45681 | 6.735438 |  |  |  |  |  |
| 4.0392 | 3.964344 | 5.214302 |  |  |  |  |  |
| 5.625951 | 5.739546 | 14.84863 |  |  |  |  |  |
| 6.707875 | 2.82732 | 11.172 |  |  |  |  |  |
| 11.0373 | 3.556896 | 8.778 |  |  |  |  |  |
| 25.10037 | 79.72772 | 8.8088 |  |  |  |  |  |
| 6.099906 | 5.984613 | 3.356972 |  |  |  |  |  |
| 11.79258 | 39.63757 | 5.0058 |  |  |  |  |  |
| 4.189956 | 12.82383 | 4.948689 |  |  |  |  |  |
| 32.82163 | 28.91083 | 33.94444 |  |  |  |  |  |
| 8.976 | 64.19409 | 35.952 |  |  |  |  |  |
| 8.263522 | 7.961887 | 21.44567 |  |  |  |  |  |
| 4.524858 | 11.19521 | 15.617 |  |  |  |  |  |
| 19.32762 | 3.129476 | 45.68207 |  |  |  |  |  |
| 5.488419 | 13.04766 | 16.692 |  |  |  |  |  |
| 3.881476 | 3.805349 | 25.038 |  |  |  |  |  |
| 2.58386 | 30.09293 | 14.453 |  |  |  |  |  |
| 6.063435 | 5.822544 | 18.36223 |  |  |  |  |  |
| 6.979166 | 2.966609 | 5.564 |  |  |  |  |  |
| 7.675078 | 15.90146 | 38.61268 |  |  |  |  |  |
| 9.621656 | 3.885608 | 13.91 |  |  |  |  |  |
| 13.67922 | 12.28611 | 11.02592 |  |  |  |  |  |
| 3.518389 | 16.44451 | 10.18193 |  |  |  |  |  |
| 56.18448 | 4.941777 | 27.82 |  |  |  |  |  |
| 31.75047 | 4.336508 | 8.139148 |  |  |  |  |  |
| 7.156214 | 6.459642 | 8.139529 |  |  |  |  |  |
| 2.825372 | 4.766741 | 2.57261 |  |  |  |  |  |
| 4.61443 | 9.577413 | 16.31826 |  |  |  |  |  |
| 12.75397 | 6.660062 | 25.82 |  |  |  |  |  |
| 6.617643 | 5.994744 | 19.50028 |  |  |  |  |  |
| 3.827943 | 6.742178 | 40.05077 |  |  |  |  |  |
| 10.12907 | 23.92887 | 46.70561 |  |  |  |  |  |
| 7.5265 | 3.425848 | 7.853063 |  |  |  |  |  |
| 0.07177298 | 3.496768 | 14.47991 |  |  |  |  |  |
| 4.772245 | 11.43125 | 25.20143 |  |  |  |  |  |
| 2.85507 | 11.15273 | 3.867625 |  |  |  |  |  |
| 10.04119 | 9.859741 | 20.60898 |  |  |  |  |  |
| 16.016 | 3.620901 | 8.887937 |  |  |  |  |  |
| 30.60171 | 14.19262 | 29.08055 |  |  |  |  |  |
| 5.405274 | 6.171581 | 12.28962 |  |  |  |  |  |
| 17.46049 | 6.265694 | 3.852592 |  |  |  |  |  |
| 19.42763 | 92.29135 | 11.99538 |  |  |  |  |  |
| 3.40428 | 15.68251 | 11.89695 |  |  |  |  |  |
| 4.424554 | 12.35041 | 16.12297 |  |  |  |  |  |
| 65.38636 | 3.817006 | 4.992023 |  |  |  |  |  |
| 3.590609 | 15.54482 | 5.852017 |  |  |  |  |  |
| 4.123029 | 7.321622 | 39.38412 |  |  |  |  |  |
| 4.553813 | 3.115478 | 6.992213 |  |  |  |  |  |
| 10.352 | 9.393144 | 2.477729 |  |  |  |  |  |
| 14.64145 | 25.4712 | 2.356633 |  |  |  |  |  |
| 18.06204 | 4.765744 | 7.468687 |  |  |  |  |  |
| 5.721161 | 9.787042 | 7.714843 |  |  |  |  |  |
| 8.376062 | 3.891175 | 34.63702 |  |  |  |  |  |
| 5.798172 | 2.880102 | 36.036 |  |  |  |  |  |
| 3.796241 | 6.118518 | 3.797916 |  |  |  |  |  |
| 7.09309 | 5.164391 | 3.385752 |  |  |  |  |  |
| 7.730821 | 9.124377 | 32.50865 |  |  |  |  |  |
| 5.621686 | 4.931213 | 6.819384 |  |  |  |  |  |
| 6.9048 | 7.058344 | 15.89016 |  |  |  |  |  |
| 19.75756 | 5.118443 | 7.406056 |  |  |  |  |  |
| 4.629084 | 2.992512 | 14.77006 |  |  |  |  |  |
| 6.294513 | 21.83809 | 12.9474 |  |  |  |  |  |
| 6.838861 | 2.90866 | 7.135694 |  |  |  |  |  |
| 4.346194 | 36.65073 | 6.75713 |  |  |  |  |  |
| 7.407705 | 3.402782 | 8.070291 |  |  |  |  |  |
| 24.62118 | 2.58081 | 6.735658 |  |  |  |  |  |
| 7.556012 | 6.464368 | 21.2059 |  |  |  |  |  |
| 5.716094 | 5.201802 | 28.78667 |  |  |  |  |  |
| 11.53209 | 5.455727 | 7.288966 |  |  |  |  |  |
| 4.505009 | 2.418641 | 5.519564 |  |  |  |  |  |
| 9.148167 | 5.419573 | 29.25994 |  |  |  |  |  |
| 9.560942 | 7.757143 | 16.53374 |  |  |  |  |  |
| 6.099583 | 2.299875 | 5.985851 |  |  |  |  |  |
| 10.19533 | 15.7886 | 35.3073 |  |  |  |  |  |
| 4.759535 | 4.312352 | 4.47136 |  |  |  |  |  |
| 5.818312 | 6.645502 | 10.328 |  |  |  |  |  |
| 4.64007 | 3.045455 | 3.40665 |  |  |  |  |  |
| 4.758364 | 15.92809 | 6.09739 |  |  |  |  |  |
| 13.82356 | 10.35863 | 4.415764 |  |  |  |  |  |
| 13.44186 | 3.040448 | 20.60898 |  |  |  |  |  |
| 31.85034 | 2.532691 | 8.887937 |  |  |  |  |  |
| 8.598788 | 3.488186 | 29.08055 |  |  |  |  |  |
| 31.48333 | 2.521327 | 12.28962 |  |  |  |  |  |
| 39.11465 | 6.359415 | 3.852592 |  |  |  |  |  |
| 112.2198 | 6.28121 | 11.99538 |  |  |  |  |  |
| 9.748828 | 6.000768 | 11.89695 |  |  |  |  |  |
| 6.580091 | 5.23348 | 16.12297 |  |  |  |  |  |
| 64.50076 | 3.8159 | 4.992023 |  |  |  |  |  |
| 14.34 | 3.079022 | 5.852017 |  |  |  |  |  |
| 3.090047 | 6.005874 | 39.38412 |  |  |  |  |  |
| 143.4 | 3.596817 | 6.992213 |  |  |  |  |  |
| 24.30853 | 4.681026 | 2.477729 |  |  |  |  |  |
| 16.77349 | 3.573191 | 2.356633 |  |  |  |  |  |
| 7.351032 | 4.653801 | 7.468687 |  |  |  |  |  |
| 26.48271 | 8.562497 | 7.714843 |  |  |  |  |  |
| 8.761492 | 4.452857 | 34.63702 |  |  |  |  |  |
| 20.97755 | 2.962298 | 36.036 |  |  |  |  |  |
| 2.971794 | 4.818895 | 3.797916 |  |  |  |  |  |
| 3.574954 | 5.780465 | 3.385752 |  |  |  |  |  |
| 54.21898 | 3.96103 | 32.50865 |  |  |  |  |  |
| 6.703867 | 3.561101 | 6.819384 |  |  |  |  |  |
| 19.12 |  | 15.89016 |  |  |  |  |  |
| 5.365467 |  | 7.406056 |  |  |  |  |  |
| 28.632 |  | 14.77006 |  |  |  |  |  |
| 9.742657 |  | 12.9474 |  |  |  |  |  |
| 5.680687 |  | 7.135694 |  |  |  |  |  |
| 12.32811 |  | 6.75713 |  |  |  |  |  |
| 15.42549 |  | 8.070291 |  |  |  |  |  |
| 7.842874 |  | 6.735658 |  |  |  |  |  |
| 9.783489 |  | 21.2059 |  |  |  |  |  |
| 31.84036 |  | 28.78667 |  |  |  |  |  |
| 3.420384 |  | 7.288966 |  |  |  |  |  |
| 9.090735 |  | 5.519564 |  |  |  |  |  |
| 24.71876 |  | 29.25994 |  |  |  |  |  |
| 15.852 |  | 16.53374 |  |  |  |  |  |
| 27.73023 |  | 5.985851 |  |  |  |  |  |
| 12.89952 |  | 35.3073 |  |  |  |  |  |
| 6.545989 |  | 4.47136 |  |  |  |  |  |
| 12.72641 |  | 10.328 |  |  |  |  |  |
| 2.243803 |  | 3.40665 |  |  |  |  |  |
| 8.888236 |  | 6.09739 |  |  |  |  |  |
| 2.525815 |  | 4.415764 |  |  |  |  |  |
| 3.112362 |  |  |  |  |  |  |  |
| 5.033709 |  |  |  |  |  |  |  |
| 8.976 |  |  |  |  |  |  |  |
| 4.009582 |  |  |  |  |  |  |  |
| 8.714693 |  |  |  |  |  |  |  |
| 3.612712 |  |  |  |  |  |  |  |
| 5.240501 |  |  |  |  |  |  |  |
| 9.703269 |  |  |  |  |  |  |  |
| 6.732 |  |  |  |  |  |  |  |
| 13.464 |  |  |  |  |  |  |  |
| 12.012 |  |  |  |  |  |  |  |
| 25.2 |  |  |  |  |  |  |  |
| 20.7931 |  |  |  |  |  |  |  |
| 132.3983 |  |  |  |  |  |  |  |
| 8.754891 |  |  |  |  |  |  |  |
| 9.045466 |  |  |  |  |  |  |  |
| 2.910122 |  |  |  |  |  |  |  |
| 14.21413 |  |  |  |  |  |  |  |
| 46.76956 |  |  |  |  |  |  |  |
| 13.63115 |  |  |  |  |  |  |  |
| 26.85452 |  |  |  |  |  |  |  |
| 52.58191 |  |  |  |  |  |  |  |
| 18.35122 |  |  |  |  |  |  |  |
| 64.50076 |  |  |  |  |  |  |  |
| 14.34 |  |  |  |  |  |  |  |
| 3.090047 |  |  |  |  |  |  |  |
| 143.4 |  |  |  |  |  |  |  |
| 24.30853 |  |  |  |  |  |  |  |
| 16.77349 |  |  |  |  |  |  |  |
| 7.351032 |  |  |  |  |  |  |  |
| 26.48271 |  |  |  |  |  |  |  |
| 8.761492 |  |  |  |  |  |  |  |
| 20.97755 |  |  |  |  |  |  |  |
| 2.971794 |  |  |  |  |  |  |  |
| 3.574954 |  |  |  |  |  |  |  |
| 54.21898 |  |  |  |  |  |  |  |
| 6.703867 |  |  |  |  |  |  |  |
| 19.12 |  |  |  |  |  |  |  |
| 5.365467 |  |  |  |  |  |  |  |
| 28.632 |  |  |  |  |  |  |  |
| 9.742657 |  |  |  |  |  |  |  |
| 5.680687 |  |  |  |  |  |  |  |
| 12.32811 |  |  |  |  |  |  |  |
| 15.42549 |  |  |  |  |  |  |  |
| 7.842874 |  |  |  |  |  |  |  |
| 9.783489 |  |  |  |  |  |  |  |
| 31.84036 |  |  |  |  |  |  |  |
| 3.420384 |  |  |  |  |  |  |  |
| 9.090735 |  |  |  |  |  |  |  |
| 24.71876 |  |  |  |  |  |  |  |
| 15.852 |  |  |  |  |  |  |  |
| 27.73023 |  |  |  |  |  |  |  |
| 12.89952 |  |  |  |  |  |  |  |
| 6.545989 |  |  |  |  |  |  |  |
| 12.72641 |  |  |  |  |  |  |  |
| 2.243803 |  |  |  |  |  |  |  |
| 8.888236 |  |  |  |  |  |  |  |
| 2.525815 |  |  |  |  |  |  |  |
| 3.112362 |  |  |  |  |  |  |  |
| 5.033709 |  |  |  |  |  |  |  |
| 8.976 |  |  |  |  |  |  |  |
| 4.009582 |  |  |  |  |  |  |  |
| 8.714693 |  |  |  |  |  |  |  |
| 3.612712 |  |  |  |  |  |  |  |
| 5.240501 |  |  |  |  |  |  |  |
| 9.703269 |  |  |  |  |  |  |  |
| 6.732 |  |  |  |  |  |  |  |
| 13.464 |  |  |  |  |  |  |  |
| 12.012 |  |  |  |  |  |  |  |
| 25.2 |  |  |  |  |  |  |  |
| 20.7931 |  |  |  |  |  |  |  |
| 132.3983 |  |  |  |  |  |  |  |
| 8.754891 |  |  |  |  |  |  |  |
| 9.045466 |  |  |  |  |  |  |  |
| 2.910122 |  |  |  |  |  |  |  |
| 14.21413 |  |  |  |  |  |  |  |
| 46.76956 |  |  |  |  |  |  |  |
| 13.63115 |  |  |  |  |  |  |  |
| 26.85452 |  |  |  |  |  |  |  |
| 52.58191 |  |  |  |  |  |  |  |
| 18.35122 |  |  |  |  |  |  |  |
| 5.48058 |  |  |  |  |  |  |  |
| 11.15447 |  |  |  |  |  |  |  |
| 10.33178 |  |  |  |  |  |  |  |
| 22.51554 |  |  |  |  |  |  |  |
| 9.593803 |  |  |  |  |  |  |  |
| 29.01124 |  |  |  |  |  |  |  |
| 20.91443 |  |  |  |  |  |  |  |
| 24.73044 |  |  |  |  |  |  |  |
| 44.73753 |  |  |  |  |  |  |  |
| 39.33306 |  |  |  |  |  |  |  |
| 7.232731 |  |  |  |  |  |  |  |
| 12.63974 |  |  |  |  |  |  |  |
| 3.689465 |  |  |  |  |  |  |  |
| 24.73606 |  |  |  |  |  |  |  |
| 70.9991 |  |  |  |  |  |  |  |
| 6.63203 |  |  |  |  |  |  |  |
| 21.33088 |  |  |  |  |  |  |  |
| 10.79433 |  |  |  |  |  |  |  |
| 3.166266 |  |  |  |  |  |  |  |
| 7.363545 |  |  |  |  |  |  |  |
| 8.80104 |  |  |  |  |  |  |  |
| 17.7456 |  |  |  |  |  |  |  |
| 5.48058 |  |  |  |  |  |  |  |
| 11.15447 |  |  |  |  |  |  |  |
| 10.33178 |  |  |  |  |  |  |  |
| 22.51554 |  |  |  |  |  |  |  |
| 9.593803 |  |  |  |  |  |  |  |
| 29.01124 |  |  |  |  |  |  |  |
| 20.91443 |  |  |  |  |  |  |  |
| 24.73044 |  |  |  |  |  |  |  |
| 44.73753 |  |  |  |  |  |  |  |
| 39.33306 |  |  |  |  |  |  |  |
| 7.232731 |  |  |  |  |  |  |  |
| 12.63974 |  |  |  |  |  |  |  |
| 3.689465 |  |  |  |  |  |  |  |
| 24.73606 |  |  |  |  |  |  |  |
| 70.9991 |  |  |  |  |  |  |  |
| 6.63203 |  |  |  |  |  |  |  |
| 21.33088 |  |  |  |  |  |  |  |
| 10.79433 |  |  |  |  |  |  |  |
| 3.166266 |  |  |  |  |  |  |  |
| 7.363545 |  |  |  |  |  |  |  |
| 8.80104 |  |  |  |  |  |  |  |
| 17.7456 |  |  |  |  |  |  |  |
| 5.48058 |  |  |  |  |  |  |  |
| 11.15447 |  |  |  |  |  |  |  |
| 10.33178 |  |  |  |  |  |  |  |
| 22.51554 |  |  |  |  |  |  |  |
| 9.593803 |  |  |  |  |  |  |  |
| 29.01124 |  |  |  |  |  |  |  |
| 20.91443 |  |  |  |  |  |  |  |
| 24.73044 |  |  |  |  |  |  |  |
| 44.73753 |  |  |  |  |  |  |  |
| 39.33306 |  |  |  |  |  |  |  |
| 7.232731 |  |  |  |  |  |  |  |
| 12.63974 |  |  |  |  |  |  |  |
| 3.689465 |  |  |  |  |  |  |  |
| 24.73606 |  |  |  |  |  |  |  |
| 70.9991 |  |  |  |  |  |  |  |
| 6.63203 |  |  |  |  |  |  |  |
| 21.33088 |  |  |  |  |  |  |  |
| 10.79433 |  |  |  |  |  |  |  |
| 3.166266 |  |  |  |  |  |  |  |
| 7.363545 |  |  |  |  |  |  |  |
| 8.80104 |  |  |  |  |  |  |  |
| 17.7456 |  |  |  |  |  |  |  |
| 4.538838 |  |  |  |  |  |  |  |
| 35.47701 |  |  |  |  |  |  |  |
| 3.511098 |  |  |  |  |  |  |  |
| 14.71913 |  |  |  |  |  |  |  |
| 45.54 |  |  |  |  |  |  |  |
| 8.270689 |  |  |  |  |  |  |  |
| 5.284 |  |  |  |  |  |  |  |
| 6.510365 |  |  |  |  |  |  |  |
| 7.47 |  |  |  |  |  |  |  |
| 26.55574 |  |  |  |  |  |  |  |
| 4.858032 |  |  |  |  |  |  |  |
| 37.8535 |  |  |  |  |  |  |  |
| 10.16531 |  |  |  |  |  |  |  |
| 12.924 |  |  |  |  |  |  |  |
| 2.690765 |  |  |  |  |  |  |  |
| 5.904 |  |  |  |  |  |  |  |
| 5.95075 |  |  |  |  |  |  |  |
| 7.871084 |  |  |  |  |  |  |  |
| 6.405833 |  |  |  |  |  |  |  |
| 22.44 |  |  |  |  |  |  |  |
| 31.10665 |  |  |  |  |  |  |  |
| 4.470404 |  |  |  |  |  |  |  |
| 8.976 |  |  |  |  |  |  |  |
| 7.515708 |  |  |  |  |  |  |  |
| 31.57458 |  |  |  |  |  |  |  |
| 2.842654 |  |  |  |  |  |  |  |
| 13.92526 |  |  |  |  |  |  |  |
| 40.65477 |  |  |  |  |  |  |  |
| 17.19075 |  |  |  |  |  |  |  |
| 31.39905 |  |  |  |  |  |  |  |
| 12.76781 |  |  |  |  |  |  |  |
| 4.22209 |  |  |  |  |  |  |  |
| 10.19136 |  |  |  |  |  |  |  |
| 5.31691 |  |  |  |  |  |  |  |
| 11.67599 |  |  |  |  |  |  |  |
| 26.71162 |  |  |  |  |  |  |  |
| 11.27041 |  |  |  |  |  |  |  |
| 29.41058 |  |  |  |  |  |  |  |
| 12.21636 |  |  |  |  |  |  |  |
| 6.128218 |  |  |  |  |  |  |  |
| 8.273719 |  |  |  |  |  |  |  |
| 4.454422 |  |  |  |  |  |  |  |
| 7.971419 |  |  |  |  |  |  |  |
| 14.1584 |  |  |  |  |  |  |  |
| 5.915753 |  |  |  |  |  |  |  |
| 25.72 |  |  |  |  |  |  |  |
| 23.2136 |  |  |  |  |  |  |  |
| 8.903547 |  |  |  |  |  |  |  |
| 7.463037 |  |  |  |  |  |  |  |
| 13.68702 |  |  |  |  |  |  |  |
| 12.16049 |  |  |  |  |  |  |  |
| 43.75962 |  |  |  |  |  |  |  |
| 5.341736 |  |  |  |  |  |  |  |
| 14.0166 |  |  |  |  |  |  |  |
| 6.70051 |  |  |  |  |  |  |  |
| 15.69208 |  |  |  |  |  |  |  |
| 4.116901 |  |  |  |  |  |  |  |
| 13.86468 |  |  |  |  |  |  |  |
| 26.55574 |  |  |  |  |  |  |  |
| 13.32014 |  |  |  |  |  |  |  |
| 5.635706 |  |  |  |  |  |  |  |
| 5.255398 |  |  |  |  |  |  |  |
| 5.513445 |  |  |  |  |  |  |  |
| 2.288074 |  |  |  |  |  |  |  |
| 13.04613 |  |  |  |  |  |  |  |
| 13.464 |  |  |  |  |  |  |  |
| 4.220756 |  |  |  |  |  |  |  |
| 4.0573 |  |  |  |  |  |  |  |
| 22.44 |  |  |  |  |  |  |  |
| 19.67834 |  |  |  |  |  |  |  |
| 11.10553 |  |  |  |  |  |  |  |
| 9.553577 |  |  |  |  |  |  |  |
| 5.990993 |  |  |  |  |  |  |  |
| 24.62673 |  |  |  |  |  |  |  |
| 16.78279 |  |  |  |  |  |  |  |
| 3.262891 |  |  |  |  |  |  |  |
| 8.764089 |  |  |  |  |  |  |  |
| 10.244 |  |  |  |  |  |  |  |
| 15.76 |  |  |  |  |  |  |  |
| 8.249209 |  |  |  |  |  |  |  |
| 3.988128 |  |  |  |  |  |  |  |
| 3.541086 |  |  |  |  |  |  |  |
| 11.67233 |  |  |  |  |  |  |  |
| 12.4877 |  |  |  |  |  |  |  |
| 4.99896 |  |  |  |  |  |  |  |
| 5.294267 |  |  |  |  |  |  |  |
| 11.2796 |  |  |  |  |  |  |  |
| 19.17723 |  |  |  |  |  |  |  |
| 4.895277 |  |  |  |  |  |  |  |
| 6.262646 |  |  |  |  |  |  |  |
| 3.255149 |  |  |  |  |  |  |  |
| 48.52668 |  |  |  |  |  |  |  |
| 5.144 |  |  |  |  |  |  |  |
| 15.432 |  |  |  |  |  |  |  |

**Stat**
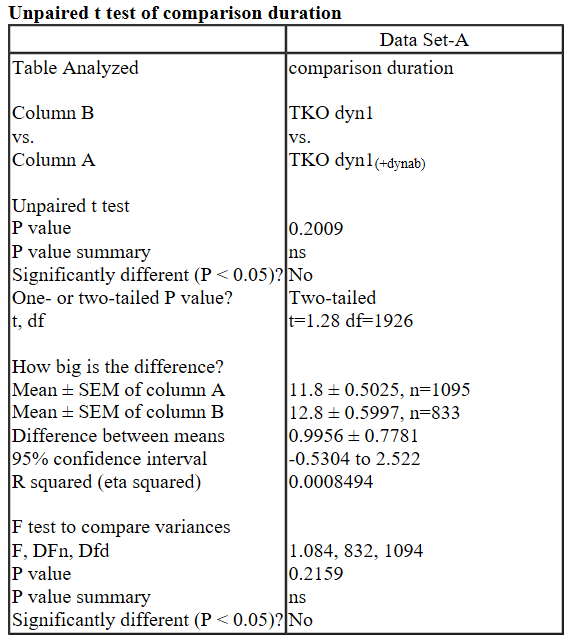
**
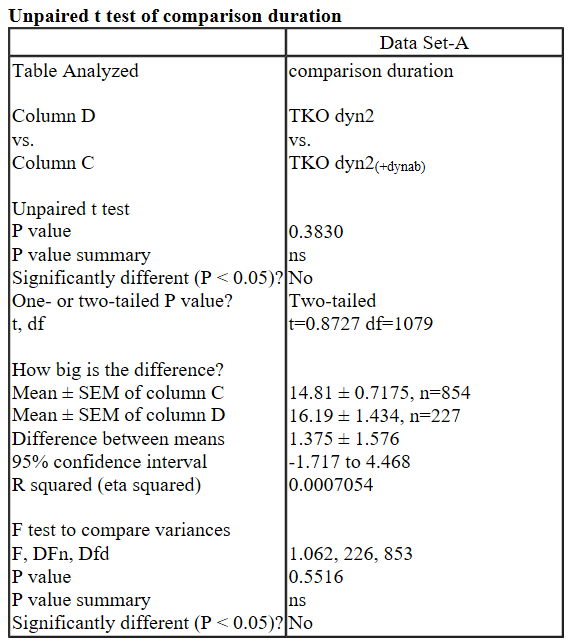
istical report:**


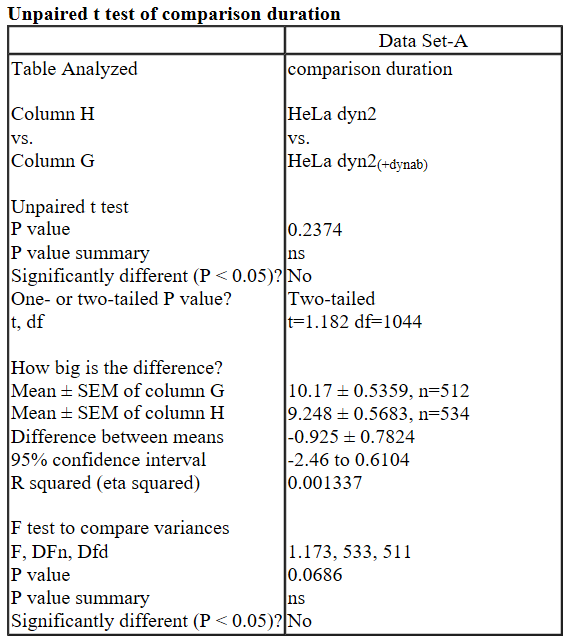

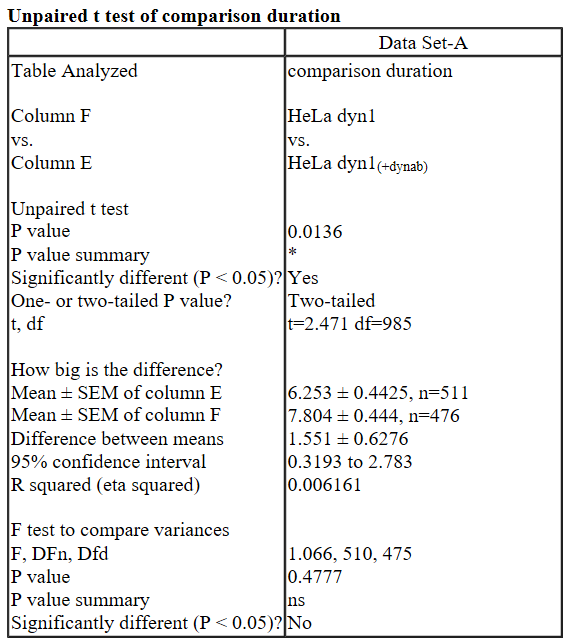

Supplement: Figure 4—source data 1. [file elife-25197-fig4-data1.docx]
