# Supplementary material for: Uncoupling of dynamin polymerization and GTPase activity revealed by the conformation-specific nanobody dynab
Source: eLife. 2017 Oct 12;6:e25197. doi: 10.7554/eLife.25197 (PMC5658065; doi:10.7554/eLife.25197)

**Figure 4-Source Data 2 (panel B)**

Comparison of duration of dynamin 1-2 events with dynab events in TKO cells (expressed in seconds), and statistical report

| dyn1 | dynab | dyn2 | dynab | dyn1_(mCherry)_ | dyn1_(EGFP_) |
| --- | --- | --- | --- | --- | --- |
| 6.601239 | 9.995221 | 13.16721 | 3.00286 | 7.203991 | 5.912338 |
| 3.506503 | 3.952905 | 5.225769 | 5.566829 | 9.660955 | 34.25826 |
| 25.75487 | 27.78848 | 4.472581 | 4.24764 | 17.74874 | 35.85027 |
| 4.486841 | 4.246546 | 18.77499 | 18.99726 | 26.65916 | 46.55021 |
| 5.660996 | 12.36173 | 6.410894 | 5.885002 | 4.397827 | 19.33025 |
| 4.869589 | 3.576422 | 5.509642 | 7.426197 | 4.242576 | 5.600904 |
| 4.030273 | 5.570869 | 5.908015 | 7.165129 | 16.63125 | 8.097821 |
| 8.457853 | 8.078739 | 19.98064 | 4.400104 | 18.29135 | 23.69734 |
| 10.73303 | 18.02338 | 9.586088 | 11.9612 | 7.643202 | 5.076652 |
| 3.651256 | 3.337142 | 12.98406 | 3.840402 | 6.621363 | 13.04953 |
| 2.66332 | 70.074 | 10.40174 | 8.419288 | 8.823449 | 5.847821 |
| 5.039145 | 3.659929 | 3.67766 | 4.572184 | 26.76375 | 6.648344 |
| 10.24008 | 3.090749 | 21.75257 | 3.746414 | 33.57137 | 17.9323 |
| 4.021745 | 17.90544 | 9.933284 | 7.787201 | 13.09273 | 32.16245 |
| 2.70845 | 4.509925 | 51.28949 | 5.083374 | 19.00513 | 4.562587 |
| 5.686243 | 18.6675 | 4.001522 | 5.934245 | 38.13901 | 29.65965 |
| 7.84943 | 12.48362 | 9.01876 | 10.44722 | 4.711988 | 15.09698 |
| 6.901536 | 9.314931 | 78.14442 | 71.86982 | 11.97361 | 40.27799 |
| 5.480954 | 12.1972 | 4.330427 | 6.052333 | 24.63629 | 20.04936 |
| 9.649129 | 3.227632 | 263.7231 | 35.7538 | 17.55672 | 4.391022 |
| 7.433951 | 6.967432 | 9.386036 | 24.3273 | 5.129579 | 3.674627 |
| 16.0564 | 5.639129 | 4.734596 | 6.078695 | 10.66077 | 3.889739 |
| 24.14206 | 6.174611 | 3.130485 | 4.395703 | 25.75778 | 6.696864 |
| 9.926873 | 4.636435 | 17.77161 | 16.1193 | 6.727761 | 9.340329 |
| 3.896017 | 4.433051 | 3.315583 | 3.658476 | 22.94514 | 31.09159 |
| 5.566408 | 3.71005 | 74.65894 | 48.23909 | 14.33171 | 11.16782 |
| 3.358333 | 10.61867 | 12.16401 | 18.97569 | 7.161775 | 7.556462 |
| 3.545781 | 10.26372 | 17.52791 | 69.16265 | 5.620329 | 5.406519 |
| 5.607464 | 14.29993 | 4.594904 | 2.528222 | 24.72344 | 12.78261 |
| 7.274641 | 5.458546 | 13.81535 | 9.106986 | 5.588624 | 8.709263 |
| 3.932723 | 8.975866 | 18.83603 | 14.15326 | 5.589542 | 1.488641 |
| 2.67282 | 11.56651 | 3.447096 | 8.408445 | 9.71182 | 7.867625 |
| 31.1945 | 4.328937 | 4.606837 | 4.680376 | 5.810025 | 2.992832 |
| 9.171896 | 4.040431 | 3.709968 | 13.24301 | 17.34874 | 7.493322 |
| 16.58884 | 7.686484 | 99.56223 | 129.0187 | 12.28341 | 13.00572 |
| 3.156313 | 3.337934 | 123.4319 | 63.02712 | 3.713656 | 4.979096 |
| 41.0473 | 7.565589 | 21.3726 | 108.7302 | 19.68364 | 9.393336 |
| 10.08856 | 4.018878 | 18.16434 | 19.99833 | 4.958287 | 5.049686 |
| 34.46348 | 26.05102 | 3.697569 | 4.757017 | 11.50149 | 2.724301 |
| 3.487533 | 3.586454 | 28.34366 | 4.730266 | 7.18887 | 3.869504 |
| 4.08073 | 5.286306 | 6.514387 | 4.447727 | 3.416616 | 11.28899 |
| 289.6324 | 22.04664 | 20.46792 | 4.244288 | 5.566361 | 13.94355 |
| 5.037975 | 4.650927 | 4.067603 | 5.938387 | 6.794577 | 25.92191 |
| 4.276617 | 1.694818 | 9.408644 | 3.801549 | 14.80653 | 14.54303 |
| 41.17758 | 6.948551 | 59.20146 | 88.93111 | 2.938381 | 16.07755 |
| 12.35903 | 7.475751 | 10.3697 | 2.541131 | 3.633917 | 8.238783 |
| 3.766881 | 7.482389 | 41.78732 | 12.35054 | 4.522598 | 8.766743 |
| 6.884367 | 6.024838 | 9.967542 | 5.608475 | 6.16872 | 9.673369 |
| 2.862266 | 45.30581 | 9.194146 | 2.829837 | 2.237648 | 10.08493 |
| 3.532404 | 4.25558 | 3.741719 | 3.124516 | 9.798207 | 4.597035 |
| 9.303043 | 5.516395 | 22.89499 | 6.969233 | 7.284353 | 32.79275 |
| 9.286666 | 15.9817 | 39.23092 | 65.53301 | 3.831719 | 4.361496 |
| 6.574863 | 5.519614 | 5.270787 | 3.795681 | 6.074435 | 9.733199 |
| 3.725224 | 8.028566 | 27.79529 | 18.62723 | 8.480995 | 11.61603 |
| 32.38119 | 3.076132 | 54.38466 | 4.383985 | 9.035067 | 8.038265 |
| 5.241805 | 17.78999 | 29.48901 | 3.22844 | 6.588844 | 4.042892 |
| 21.49392 | 3.987768 | 7.636266 | 3.467713 | 3.272231 | 2.242568 |
| 15.66882 | 7.308071 | 11.81329 | 15.04927 | 4.933147 | 7.03158 |
| 3.625027 | 9.653882 | 6.566845 | 3.550945 | 3.412879 | 3.021835 |
| 9.899335 | 26.546 | 9.172931 | 10.01306 | 7.34307 | 3.571763 |
| 6.338922 | 3.550131 | 8.72349 | 7.162758 | 7.182002 | 4.442941 |
| 9.374723 | 3.200125 | 4.852861 | 4.275064 | 3.893484 | 13.43533 |
| 11.20459 | 5.537598 | 15.15425 | 19.26046 | 17.39871 | 19.3263 |
| 8.429011 | 3.820305 | 34.15361 | 43.51838 | 5.529788 | 7.919153 |
| 11.9604 | 3.982621 | 13.85996 | 3.022636 | 7.008777 | 5.687383 |
| 8.546485 | 5.513272 | 7.063757 | 7.561601 | 7.690171 | 5.990962 |
| 3.887546 | 3.775442 | 29.63056 | 55.27802 | 8.956064 | 8.209237 |
| 6.406134 | 5.899497 | 3.591331 | 14.35976 | 10.10104 | 7.172067 |
| 9.86976 | 5.967999 | 4.623187 | 4.345072 | 3.951349 | 2.8024 |
| 3.987934 | 3.166309 | 11.80435 | 6.496219 | 4.374559 | 3.570605 |
| 5.298746 | 8.07861 | 5.918113 | 12.85369 | 3.600255 | 4.211978 |
| 2.612122 | 3.990624 | 4.924493 | 4.038845 | 3.250015 | 2.848321 |
| 8.544926 | 2.872143 | 4.365512 | 21.34609 | 8.776362 | 5.98788 |
| 5.606765 | 2.80518 | 8.295631 | 5.143912 | 5.991222 | 2.874709 |
| 5.037157 | 5.858604 | 3.647801 | 3.408214 | 2.997206 | 10.37935 |
| 8.375821 | 10.67699 | 12.42799 | 3.968732 | 4.767595 | 9.803012 |
| 34.89538 | 4.456241 | 4.31396 | 3.612554 | 22.04268 | 32.39635 |
| 13.42367 | 6.730992 | 25.31272 | 6.992793 | 10.14923 | 8.468175 |
| 4.329061 | 8.495546 | 5.321562 | 5.192022 | 6.664798 | 5.918873 |
| 3.032381 | 3.898234 | 6.615696 | 3.489594 | 3.339613 | 9.779738 |
| 3.326238 | 2.460617 | 7.846164 | 16.75618 | 3.012497 | 4.14943 |
| 24.30967 | 4.824198 | 6.145126 | 3.946956 | 16.29298 | 2.987504 |
| 4.915221 | 5.484093 | 6.205803 | 5.323943 | 7.235467 | 3.940669 |
| 8.05799 | 20.99577 | 5.79036 | 4.424428 | 8.190126 | 8.603503 |
| 3.215723 | 8.608434 | 4.727397 | 3.61549 | 3.96529 | 6.362504 |
| 16.34647 | 12.28761 | 6.126765 | 5.351804 | 8.659825 | 3.465396 |
| 24.67756 | 19.54838 | 6.84294 | 4.423292 | 4.684404 | 0.1017397 |
| 11.34904 | 2.637619 | 3.256747 | 3.722954 | 19.95139 | 8.790089 |
| 17.43212 | 3.057376 | 3.86668 | 9.492382 | 4.967065 | 5.105222 |
| 4.318324 | 16.63615 | 6.7612 | 4.515231 | 4.2371 | 29.42171 |
| 6.85176 | 11.42476 | 25.58028 | 9.480414 | 6.225089 | 6.446301 |
| 35.80468 | 43.76403 | 15.60861 | 8.325929 | 3.584754 | 16.38596 |
| 17.6364 | 3.296758 | 3.80801 | 4.08288 | 6.500743 | 4.217316 |
| 8.018537 | 13.72586 | 1.546486 | 15.60017 | 5.23428 | 8.922183 |
| 5.13004 | 12.2008 | 6.832849 | 9.647064 | 12.34342 | 20.22016 |
| 3.760289 | 8.66532 | 6.128088 | 3.539777 | 6.162771 | 4.342907 |
| 4.465593 | 4.20302 | 26.67641 | 6.263351 | 7.360387 | 7.023242 |
| 4.240802 | 3.594361 | 5.425948 | 3.027612 | 0.1166037 | 5.170791 |
| 5.23154 | 6.165955 | 13.00051 | 4.464825 | 3.180382 | 5.015769 |
| 54.49449 | 4.999727 | 11.44847 | 11.30347 | 0.1806578 | 4.036634 |
| 11.76827 | 1.656072 | 12.1472 | 5.577957 | 14.91284 | 12.64552 |
| 27.43649 | 4.644374 | 4.53909 | 3.340395 | 4.817195 | 8.018229 |
| 6.851221 | 23.94677 | 4.522498 | 3.218456 | 4.671888 | 3.874109 |
| 7.938857 | 3.679429 | 3.388105 | 4.317982 | 7.96347 | 10.22385 |
| 4.533852 | 10.01712 | 3.372577 | 12.69311 | 4.327872 | 3.556427 |
| 16.03319 | 4.833516 | 11.89888 | 2.233143 | 19.26354 | 4.938396 |
| 6.022697 | 8.561143 | 3.445469 | 5.884283 | 3.063119 | 3.418865 |
| 9.114733 | 6.191541 | 6.971035 | 5.918781 | 4.853183 | 10.1424 |
| 8.343234 | 10.51647 | 5.013208 | 15.20972 | 5.081871 | 18.67736 |
| 5.011595 | 7.301429 | 4.040196 | 3.657883 | 4.055011 | 5.37187 |
| 10.01492 | 5.489153 | 2.461752 | 5.425719 | 2.994469 | 9.753751 |
| 7.105522 | 4.574928 | 4.385135 | 3.484995 | 4.031634 | 5.242071 |
| 2.535899 | 10.66331 | 3.085811 | 3.586314 | 6.640475 | 4.941623 |
| 9.128949 | 4.56479 | 4.844326 | 7.31711 | 12.59833 | 21.44817 |
| 12.19291 | 4.802765 | 12.74232 | 4.542957 | 4.350469 | 12.81717 |
| 5.246868 | 3.313974 | 10.06827 | 12.24486 | 5.034447 | 9.96189 |
| 14.92023 | 8.292828 | 2.687056 | 5.458409 | 4.318938 | 10.53057 |
| 7.363852 | 4.541273 | 6.418931 | 2.238192 | 5.944736 | 9.167939 |
| 3.60881 | 3.295129 | 4.171983 | 2.38529 | 8.619822 | 8.823719 |
| 3.909614 | 3.324812 | 5.966401 | 4.850575 | 0.1513424 | 4.652854 |
| 7.392715 | 9.667679 | 3.216907 | 3.361805 | 8.414925 | 3.465042 |
| 11.26654 | 11.82602 | 8.244109 | 2.889852 | 5.609001 | 16.09507 |
| 29.2915 | 3.577582 | 24.91424 | 2.809573 | 5.313032 | 10.31277 |
| 33.35446 | 5.518491 | 8.793033 | 4.280822 | 5.389209 | 7.463441 |
| 18.54682 | 6.738194 | 5.682665 | 4.762993 | 5.597436 | 8.582078 |
| 15.58385 | 4.188734 | 11.61493 | 4.544875 | 4.387371 | 9.697627 |
| 6.9534 | 7.197972 | 5.599602 | 3.177604 | 2.745182 | 2.714043 |
| 10.03725 | 5.817337 | 8.79696 | 5.800674 | 12.52768 | 6.858794 |
| 9.759346 | 3.088107 | 3.013871 | 9.088357 | 1.924819 | 53.93187 |
| 4.315737 | 5.187456 | 3.222785 | 3.47218 | 10.52241 | 4.465017 |
| 7.993102 | 4.246253 | 9.111304 | 6.469969 | 4.46111 | 8.217895 |
| 11.18344 | 8.52097 | 7.976821 | 20.33106 | 9.674386 | 11.22701 |
| 4.858521 | 4.491039 | 4.208205 | 6.863726 | 11.46936 | 7.396626 |
| 16.86399 | 6.71068 | 3.920396 | 4.803069 | 2.232503 | 8.976362 |
| 3.620277 | 3.742536 | 26.24392 | 5.464766 | 0.2133081 | 2.923276 |
| 3.609698 | 18.08524 | 69.56977 | 31.21465 | 3.202511 | 4.032134 |
| 6.399356 | 5.893486 | 5.475939 | 13.01696 | 10.43219 | 3.145085 |
| 6.70967 | 7.59237 | 4.069629 | 6.468095 | 12.05884 | 6.449664 |
| 7.247655 | 5.261884 | 5.982146 | 5.75431 | 19.50431 | 8.138099 |
| 62.51002 | 15.54065 | 7.38865 | 20.65398 | 2.916345 | 6.896412 |
| 3.920823 | 3.3741 | 4.110736 | 6.859558 | 7.286245 | 3.859164 |
| 4.194264 | 12.02015 | 12.95947 | 8.727617 | 10.76007 | 43.6455 |
| 4.74872 | 5.631853 | 5.769785 | 4.737738 | 3.860962 | 5.484114 |
| 9.810928 | 4.409795 | 9.708482 | 4.813087 | 15.72605 | 5.782476 |
| 2.997025 | 7.026991 | 2.455581 | 5.599465 | 3.667882 | 3.406796 |
| 2.053964 | 10.83892 | 6.565662 | 5.149615 | 18.7059 | 12.46006 |
| 11.6966 | 5.980379 | 5.189806 | 3.992714 | 4.084969 | 9.513204 |
| 6.851951 | 7.226187 | 4.171609 | 3.676594 | 4.004164 | 2.990873 |
| 3.934465 | 4.290968 | 174.4749 | 16.78946 | 4.916543 | 3.195809 |
| 34.49156 | 5.052906 | 16.14113 | 4.648816 | 3.819557 | 4.105819 |
| 9.434631 | 8.382722 | 4.27447 | 3.605647 | 3.587351 | 6.057126 |
| 5.318674 | 3.406835 | 4.247444 | 5.387233 | 3.797103 | 6.433229 |
| 9.612067 | 4.97736 | 4.82913 | 5.084332 | 6.103544 | 3.847619 |
| 4.542065 | 2.75287 | 3.79376 | 17.62596 | 5.806458 | 3.332617 |
| 3.664837 | 6.415312 | 48.91218 | 6.805299 | 4.257567 | 2.545345 |
| 7.910212 | 3.03085 | 7.231506 | 4.919991 | 3.224964 | 4.123436 |
| 5.515055 | 6.898328 | 25.82561 | 2.502878 | 6.176242 | 7.958957 |
| 5.99265 | 1.986063 | 12.0744 | 4.746199 | 3.328197 | 4.185818 |
| 12.99139 | 15.43017 | 7.56512 | 8.716424 | 0.0932265 | 2.337415 |
| 2.697125 | 1.97061 | 4.686913 | 16.62514 | 4.728811 | 13.43781 |
| 3.113596 | 2.798659 | 7.349073 | 14.54676 | 7.528108 | 20.54613 |
| 3.412664 | 2.970696 | 4.152588 | 6.399143 | 8.048098 | 6.383532 |
| 1.957097 | 2.231327 | 4.385338 | 4.673839 | 16.32519 | 0.109315 |
| 3.261529 | 3.926644 | 6.877095 | 10.40266 | 14.52175 | 9.841592 |
| 1.698347 | 6.782716 | 7.03721 | 9.93595 | 3.790643 | 4.045259 |
| 3.812539 | 3.192208 | 7.803881 | 8.176987 | 4.094632 | 3.072945 |
| 3.480757 | 4.027878 | 4.804303 | 3.220098 | 10.86035 | 10.6557 |
| 4.054862 | 4.364467 | 56.25779 | 2.711426 | 10.21725 | 6.823501 |
| 5.256277 | 1.94212 | 5.416386 | 21.75804 | 9.377433 | 18.15837 |
| 9.103899 | 10.45072 | 11.03415 | 5.398554 | 32.31481 | 7.543936 |
| 3.143964 | 2.22037 | 8.542464 | 15.16951 | 9.049501 | 8.849155 |
| 5.707292 | 4.735583 | 4.227625 | 12.48772 | 5.409981 | 8.057001 |
| 9.202372 | 2.202417 | 4.1771 | 4.708012 | 4.416951 | 3.971075 |
| 7.372858 | 5.184757 | 9.236404 | 5.188549 | 13.4367 | 9.734019 |
| 3.677961 | 2.787957 | 25.09481 | 7.948218 | 8.102308 | 5.146005 |
| 6.980219 | 9.755283 | 12.72817 | 11.61873 | 4.183589 | 3.60554 |
| 5.386555 | 4.635779 | 4.634752 | 8.064754 | 7.002508 | 7.423266 |
| 86.24833 | 2.52845 | 5.929452 | 27.82038 | 6.39028 | 3.123581 |
| 2.914724 | 3.549255 | 3.785853 | 17.8609 | 4.858437 | 3.100842 |
| 5.943244 | 4.880096 | 4.224237 | 22.69719 | 4.503623 | 4.715435 |
| 4.176275 | 3.551858 | 8.585425 | 5.776719 | 4.849227 | 10.80878 |
| 4.427634 | 3.108851 | 6.053139 | 7.805488 | 3.233926 | 3.388127 |
| 1.77803 | 5.782612 | 21.62441 | 4.373094 | 3.888601 | 6.503881 |
| 4.04854 | 2.164789 | 3.356513 | 7.201933 | 22.79254 | 12.88129 |
| 5.124496 | 6.244315 | 9.740605 | 6.832132 | 6.093267 | 6.267832 |
| 22.6047 | 4.528274 | 118.9276 | 37.94382 | 4.164722 | 3.358773 |
| 8.26875 | 5.01341 | 74.68453 | 34.15209 | 23.8075 | 18.08704 |
| 5.11018 | 4.435997 | 4.660533 | 5.414307 | 9.575053 | 12.62044 |
| 3.791409 | 5.783412 | 6.552241 | 8.575116 | 15.32404 | 2.592728 |
| 3.695879 | 3.688581 | 6.883797 | 6.761725 | 10.64687 | 5.166028 |
| 5.621859 | 2.014166 | 75.73339 | 13.68829 | 13.11332 | 6.668077 |
| 2.85839 | 6.816367 | 4.774509 | 5.48108 | 6.040554 | 9.189458 |
| 5.575926 | 2.878861 | 2.796174 | 10.71084 | 5.041191 | 5.517229 |
| 2.861722 | 3.849234 | 19.51279 | 10.74059 | 18.15387 | 5.910661 |
| 11.53907 | 4.13294 | 13.65409 | 15.77418 | 3.824967 | 3.593236 |
| 4.281544 | 4.05669 | 23.35519 | 14.28754 | 9.352269 | 4.10284 |
| 9.318782 | 6.987356 | 8.004214 | 6.905202 | 4.546042 | 5.996986 |
| 5.213916 | 4.181626 | 17.858 | 24.51106 | 11.25963 | 12.76087 |
| 8.842998 | 6.377607 | 4.493874 | 4.044027 | 5.036432 | 3.983582 |
| 4.558892 | 4.718414 | 31.63354 | 50.91648 | 20.70276 | 4.805573 |
| 3.372264 | 2.51376 | 11.79336 | 12.40632 | 13.7311 | 7.688636 |
| 4.294738 | 3.924891 | 38.95685 | 6.966627 | 9.723073 | 5.363482 |
| 45.14282 | 26.11514 | 5.604908 | 5.438915 | 8.173783 | 5.273134 |
| 2.915927 | 4.374406 | 14.89115 | 4.055764 | 8.713862 | 3.963548 |
| 2.892428 | 1.848857 | 27.37437 | 12.3971 | 2.861108 | 4.81179 |
| 8.076951 | 6.994224 | 15.56407 | 6.451788 | 19.56106 | 5.097131 |
| 7.986841 | 4.697947 | 9.803103 | 2.684609 | 4.177951 | 12.97964 |
| 3.606245 | 3.007296 | 13.0093 | 5.36793 | 2.873285 | 3.083233 |
| 3.538414 | 2.924171 | 81.7196 | 4.741071 | 26.28133 | 20.2911 |
| 6.562732 | 4.962664 | 138.8298 | 22.61468 | 6.391114 | 22.70503 |
| 3.396733 | 2.919382 | 5.124485 | 4.615209 | 7.085511 | 5.022155 |
| 5.565812 | 4.757752 | 36.20427 | 35.30413 | 10.63976 | 4.951952 |
| 5.589819 | 5.491588 | 16.10084 | 6.658184 | 4.809445 | 18.47359 |
| 4.99152 | 9.170203 | 6.55274 | 6.482495 | 26.87084 | 9.425723 |
| 3.322018 | 7.601766 | 10.8075 | 12.44574 | 3.197124 | 4.034306 |
| 7.695147 | 3.402306 | 8.570738 | 34.16992 | 4.697808 | 3.304166 |
| 6.118463 | 6.329036 | 19.9127 | 5.170894 | 6.031368 | 5.279575 |
| 8.313094 | 9.919575 | 13.38008 | 12.00026 | 19.56508 | 23.12317 |
| 4.751836 | 1.671096 | 7.71465 | 4.277632 | 9.974898 | 2.605393 |
| 6.649975 | 5.168247 | 172.588 | 32.85774 | 19.15105 | 10.06243 |
| 5.779444 | 3.025195 | 149.2239 | 13.93456 | 5.097847 | 12.32966 |
| 4.271715 | 4.22414 | 4.623735 | 5.044135 | 7.409073 | 12.27284 |
| 6.992066 | 3.789164 | 37.00671 | 6.848146 | 7.975769 | 13.22257 |
| 4.010108 | 4.156733 | 9.616763 | 13.14946 | 4.678237 | 7.092587 |
| 7.779538 | 4.890309 | 3.223971 | 10.68952 | 11.76756 | 6.584446 |
| 7.762373 | 6.157542 | 36.01799 | 4.523424 | 0.1640225 | 3.646728 |
| 7.076436 | 6.204396 | 20.33345 | 35.57042 | 13.94547 | 10.89178 |
| 15.42269 | 5.152981 | 18.37951 | 5.982534 | 12.34782 | 14.64681 |
| 9.142713 | 5.088483 | 4.357965 | 23.659 | 16.85034 | 17.26615 |
| 10.43398 | 7.35474 | 17.44017 | 3.980607 | 16.33081 | 11.70638 |
| 14.88354 | 3.420711 | 16.85471 | 9.393925 | 26.59815 | 23.06943 |
| 4.301896 | 2.253207 | 2.049132 | 69.21637 | 4.047535 | 4.037228 |
| 4.380212 | 7.480888 | 28.35785 | 26.90232 | 18.18915 | 26.39402 |
| 0.04062336 | 32.77996 | 5.579033 | 7.878412 | 5.308237 | 3.146415 |
| 40.07052 | 4.185303 | 11.31348 | 5.246376 | 5.637125 | 0.1227073 |
| 3.46478 | 5.787594 | 6.067636 | 32.60891 | 12.99831 | 12.59656 |
| 5.660808 | 5.20326 | 5.137618 | 6.490778 | 3.814761 | 7.248858 |
| 3.744282 | 4.16084 | 3.939713 | 4.502408 | 33.7431 | 59.89217 |
| 2.977684 | 6.89929 | 25.52488 | 15.77298 | 6.990657 | 15.90619 |
| 9.55021 | 4.817154 | 9.454031 | 23.96472 | 5.068337 | 4.445911 |
| 4.624945 | 8.826451 | 13.95434 | 15.52127 | 5.548848 | 116.2066 |
| 4.01183 | 3.123179 | 3.409006 | 4.686258 | 2.228505 | 6.288209 |
| 4.724096 | 4.005858 | 12.3465 | 6.017286 | 49.345 | 6.504456 |
| 3.081964 | 3.21402 | 9.558196 | 5.384745 | 15.03372 | 5.551516 |
| 8.767394 | 5.072487 | 5.104276 | 4.703847 | 24.84307 | 45.25753 |
| 34.79025 | 3.376859 | 5.350594 | 9.777535 | 27.65682 | 3.087961 |
| 4.70221 | 2.571769 | 19.79153 | 30.57769 | 6.384366 | 33.53078 |
| 2.85287 | 3.295068 | 6.146181 | 5.180829 | 28.58174 | 10.19284 |
| 3.337415 | 18.84081 | 7.878509 | 5.913457 | 17.71469 | 15.73484 |
| 11.92834 | 6.739155 | 7.082289 | 8.527654 | 29.96337 | 8.969072 |
| 14.16084 | 9.195442 | 13.29337 | 8.104377 | 3.709776 | 5.160313 |
| 4.909853 | 15.87408 | 5.532242 | 14.39956 | 8.594212 | 5.329536 |
| 4.732589 | 11.67845 | 9.043407 | 4.435936 | 8.978473 | 4.716881 |
| 15.15814 | 3.343662 | 3.490898 | 4.716331 | 11.34607 | 10.99254 |
| 7.118374 | 3.341613 | 4.045372 | 6.331377 | 3.98998 | 6.273393 |
| 5.219141 | 5.248621 | 4.941267 | 52.6709 | 8.374973 | 6.500522 |
| 9.680541 | 3.286176 | 39.52813 | 25.29428 | 15.09974 | 14.09942 |
| 4.848881 | 2.982038 | 4.975129 | 4.332839 | 3.67455 | 8.313058 |
| 4.253516 | 3.089422 | 4.022162 | 5.618841 | 4.08734 | 5.069774 |
| 5.287558 | 2.951681 | 5.618433 | 20.98066 | 3.664604 | 3.656696 |
| 4.449408 | 5.329298 | 4.505091 | 5.567891 | 15.11892 | 4.457269 |
| 5.814721 | 4.136901 | 19.48909 | 17.04593 | 8.96996 | 5.524154 |
| 8.279238 | 4.692695 | 5.035456 | 9.524114 | 12.25808 | 5.35455 |
| 3.889213 | 2.494839 | 3.956656 | 5.871809 | 5.677988 | 4.78643 |
| 6.384449 | 5.098542 | 10.97533 | 11.39219 | 15.07876 | 13.41814 |
| 5.323805 | 4.497154 | 4.303393 | 16.04509 | 4.092567 | 6.469502 |
| 3.724129 | 9.833886 | 9.368612 | 9.766573 | 20.92869 | 6.895237 |
| 5.60606 | 4.265395 | 7.656224 | 21.06002 | 2.579894 | 5.155082 |
| 6.244517 | 4.484365 | 5.528324 | 3.17592 | 2.994583 | 4.290494 |
| 5.511223 | 4.459464 | 40.73651 | 8.149459 | 7.159578 | 6.356527 |
| 6.02435 | 14.19111 | 6.645213 | 6.153147 | 8.566877 | 3.274966 |
| 3.165485 | 3.468243 | 7.167282 | 9.13828 | 7.058427 | 14.77784 |
| 4.78589 | 2.931102 | 5.399017 | 8.921624 | 3.071647 | 2.795298 |
| 3.12392 | 4.245328 | 3.777481 | 4.248559 | 34.32684 | 19.23074 |
| 2.801918 | 3.944589 | 14.32438 | 7.614513 | 37.25924 | 6.238349 |
| 12.25824 | 3.158149 | 3.649547 | 8.016165 | 17.84637 | 17.10031 |
| 2.635849 | 5.318861 | 7.815723 | 6.036832 | 3.072798 | 4.966551 |
| 8.376735 | 4.724597 | 5.510051 | 8.695894 | 11.55986 | 14.71973 |
| 2.751365 | 5.912176 | 12.41447 | 4.203023 | 12.27828 | 4.319873 |
| 5.99678 | 3.604662 | 32.96571 | 188.1577 | 5.297438 | 4.387695 |
| 4.318438 | 5.232462 | 7.801152 | 13.73786 | 2.970151 | 6.534888 |
| 7.541431 | 4.130077 | 5.221347 | 10.03873 | 9.653846 | 10.0512 |
| 0.04285149 | 7.922652 | 4.508008 | 128.061 | 12.29324 | 5.301491 |
| 16.24216 | 3.112199 | 6.240302 | 4.688638 | 3.827018 | 18.36423 |
| 2.410538 | 3.717404 | 3.975317 | 5.312149 | 5.243604 | 4.539728 |
| 10.09539 | 4.288944 | 6.71226 | 8.993426 | 4.120117 | 14.01178 |
| 8.639343 | 2.498183 | 5.62747 | 4.317475 | 12.95101 | 5.323132 |
| 4.751951 | 3.421192 | 8.238041 | 5.392764 | 6.478272 | 6.738465 |
| 4.80213 | 4.777433 | 28.68241 | 12.24207 | 10.44133 | 18.14445 |
| 5.977094 | 6.547788 | 3.21931 | 3.817247 | 5.894994 | 11.35307 |
| 8.660271 | 4.558222 | 4.165478 | 8.236801 | 3.303262 | 3.753996 |
| 25.45924 | 3.947792 | 16.77053 | 10.64649 | 9.987407 | 25.27877 |
| 12.39786 | 2.988804 | 13.26751 | 8.405776 | 6.718028 | 5.254366 |
| 3.761934 | 2.831059 | 22.15343 | 11.82316 | 3.150414 | 3.246992 |
| 6.011902 | 3.831323 | 9.039318 | 4.81882 | 2.898106 | 6.141514 |
| 2.714024 | 47.71598 | 11.9528 | 7.706718 | 2.768815 | 5.684731 |
| 3.017363 | 3.765818 | 2.956631 | 6.01895 | 12.35461 | 7.392498 |
| 2.991777 | 6.343241 | 10.84465 | 5.813219 | 32.91492 | 7.080758 |
| 8.497955 | 34.18996 | 6.904407 | 8.310011 | 2.984753 | 26.6979 |
| 3.682882 | 3.622032 | 6.60074 | 5.796201 | 3.860082 | 3.084229 |
| 6.053695 | 5.898509 | 11.23325 | 9.220259 | 4.504798 | 5.901092 |
| 11.87306 | 2.841773 | 24.16445 | 31.1413 | 21.01298 | 4.56611 |
| 3.936437 | 2.887198 | 11.26212 | 11.96686 | 5.077432 | 6.006414 |
| 3.923166 | 3.364209 | 17.55957 | 41.91518 | 5.727711 | 11.85051 |
| 4.841562 | 21.61263 | 27.56569 | 14.79422 | 10.17865 | 7.930642 |
| 2.778406 | 11.21492 | 16.48417 | 18.33965 | 3.836276 | 4.68979 |
| 2.051529 | 3.215869 | 25.55696 | 24.86342 | 18.81617 | 17.51884 |
| 4.008139 | 3.098147 | 10.46268 | 8.705978 | 6.023368 | 20.45721 |
| 14.804 | 4.244556 | 8.901562 | 7.384763 | 9.915848 | 5.237718 |
| 14.804 | 3.021833 | 52.04753 | 10.21545 | 24.19432 | 28.27479 |
| 4.488493 | 5.843274 | 61.29009 | 14.16266 | 6.777548 | 3.611521 |
| 3.383193 | 3.949523 | 18.45032 | 11.03901 | 5.973516 | 6.309769 |
| 8.544699 | 5.189458 | 16.52413 | 43.38211 | 5.136202 | 9.241056 |
| 6.534238 | 4.015432 | 63.04087 | 20.06494 | 5.300067 | 2.526761 |
| 4.772032 | 4.741179 | 84.79265 | 6.979206 | 10.39952 | 14.40244 |
| 3.880112 | 4.061438 | 24.96808 | 37.80623 | 5.459425 | 4.908221 |
| 7.477232 | 4.847246 | 18.89517 | 15.87486 | 5.163482 | 10.42895 |
| 3.117263 | 64.99601 | 121.3563 | 9.654414 | 30.63061 | 19.44932 |
| 7.473566 | 2.846097 | 34.14069 | 21.54542 | 13.70864 | 4.383588 |
| 3.922775 | 4.706869 | 17.23042 | 16.26261 | 10.8274 | 4.7997 |
| 3.994827 | 7.228035 | 57.69523 | 33.99432 | 16.27295 | 6.863873 |
| 8.719735 | 8.566659 | 7.929545 | 10.52331 | 8.264498 | 5.437359 |
| 2.894929 | 5.551334 | 49.12331 | 3.971279 | 4.259457 | 0.07787053 |
| 8.870029 | 7.719409 | 3.287023 | 4.072053 | 21.00302 | 22.04697 |
| 2.892849 | 3.883691 | 4.997186 | 9.009845 | 2.313174 | 12.24999 |
| 15.88422 | 2.913967 | 9.83792 | 9.300607 | 3.330875 | 17.3141 |
| 6.907551 | 7.925595 | 24.22433 | 6.307758 | 4.601985 | 4.421738 |
| 4.387765 | 3.546309 | 4.581924 | 3.313572 | 20.15412 | 18.89754 |
| 3.74782 | 3.059538 | 9.552371 | 12.34666 | 10.87495 | 6.091283 |
| 3.009335 | 2.922587 | 4.704533 | 4.037169 | 15.85903 | 11.17504 |
| 3.963594 | 5.006411 | 3.866858 | 22.62418 | 56.8395 | 8.289217 |
| 5.193597 | 3.248075 | 5.265091 | 19.65661 | 4.476726 | 4.69757 |
| 15.43111 | 5.350456 | 4.718308 | 4.37529 | 12.52441 | 19.69945 |
| 29.5054 | 16.62849 | 18.23197 | 18.32852 | 20.92399 | 6.142985 |
| 2.828272 | 4.523005 | 3.430473 | 3.028467 | 9.321346 | 10.56766 |
| 3.31834 | 5.168972 | 6.952669 | 8.887322 | 4.076197 | 6.138518 |
| 6.855222 | 6.228554 | 18.09745 | 3.629558 | 6.52043 | 5.073715 |
| 5.401934 | 6.226956 | 5.885726 | 2.222147 | 4.839965 | 3.566385 |
| 5.173198 | 4.989695 | 4.407341 | 6.166889 | 4.107508 | 3.846257 |
| 4.515474 | 8.048595 | 3.892681 | 22.81236 | 7.725777 | 6.49946 |
| 3.428305 | 5.513733 | 8.34601 | 43.14532 | 5.300215 | 5.869689 |
| 21.66125 | 7.874993 | 18.72325 | 4.788541 | 17.44849 | 18.75608 |
| 3.479407 | 3.026189 | 35.50417 | 7.662522 | 4.375711 | 4.281593 |
| 13.76061 | 11.47616 | 3.091796 | 56.94389 | 4.13079 | 2.523284 |
| 4.9379 | 5.311348 | 8.240883 | 6.444412 | 12.05814 | 5.989805 |
| 3.42004 | 2.985175 | 61.18681 | 143.4961 | 6.811767 | 16.90366 |
| 4.568493 | 4.883586 | 9.757773 | 7.039172 | 6.624611 | 8.594232 |
| 3.025389 | 5.717724 | 21.29475 | 10.37229 | 15.33121 | 35.37368 |
| 9.906774 | 3.932604 | 17.25387 | 18.89058 | 5.674376 | 3.235983 |
| 5.603872 | 9.802825 | 4.718218 | 7.667043 | 3.970834 | 2.409877 |
| 5.686895 | 7.685868 | 8.45488 | 121.4702 | 2.733612 | 3.774618 |
| 5.800248 | 6.833573 | 4.313939 | 6.986473 | 24.42746 | 3.943278 |
| 3.618303 | 8.223179 | 31.75673 | 4.523606 | 16.18621 | 8.763257 |
| 2.77543 | 4.740474 | 6.357782 | 7.119955 | 8.065529 | 5.47469 |
| 10.07808 | 4.297806 | 3.824291 | 44.98547 | 13.7208 | 18.89484 |
| 1.678691 | 7.469112 | 12.14396 | 3.428623 | 4.072809 | 2.307886 |
| 2.919163 | 4.169969 | 12.36095 | 12.40913 | 4.135562 | 5.386833 |
| 4.021283 | 7.34842 | 8.371486 | 3.557207 | 16.35267 | 13.63833 |
| 6.82319 | 5.524205 | 7.517094 | 4.253328 | 4.959188 | 16.02893 |
| 5.926712 | 12.30272 | 3.567012 | 5.64988 | 3.23292 | 17.02527 |
| 3.183608 | 3.23244 | 5.755218 | 4.93929 | 14.70155 | 5.290557 |
| 6.849532 | 4.282531 | 3.392108 | 4.180897 | 5.967659 | 7.24931 |
| 8.014784 | 5.915498 | 7.211825 | 6.977488 | 22.88203 | 26.06702 |
| 6.236865 | 4.285129 | 68.05651 | 4.068015 | 5.198159 | 6.564859 |
| 5.915313 | 3.23171 | 15.99547 | 13.37295 | 9.357575 | 6.060497 |
| 4.054087 | 2.486214 | 9.370538 | 14.01315 | 3.693846 | 3.637211 |
| 3.61472 | 10.36528 | 21.65502 | 7.431468 | 3.436599 | 4.010841 |
| 44.6707 | 26.8134 | 18.21679 | 10.85539 | 8.450026 | 4.282989 |
| 3.446631 | 2.884272 | 14.95801 | 7.231862 | 20.16247 | 19.02158 |
| 11.05151 | 4.245173 | 4.623738 | 9.477262 | 10.21064 | 5.197194 |
| 35.53352 | 8.903322 | 7.794639 | 53.03349 | 5.607892 | 4.125441 |
| 3.732332 | 3.943369 | 25.39615 | 9.856755 | 2.311571 | 2.814868 |
| 7.183074 | 5.123961 | 3.924173 | 9.011875 | 3.036212 | 62.11022 |
| 3.599945 | 8.51711 | 7.407055 | 17.58656 | 5.024574 | 8.15672 |
| 9.253977 | 8.164023 | 3.718872 | 7.660856 | 22.50041 | 2.370506 |
| 13.26536 | 37.22824 | 5.968917 | 3.45072 | 7.319258 | 6.520428 |
| 11.26065 | 16.94607 | 2.046474 | 9.844733 | 28.41405 | 11.81897 |
| 3.331935 | 9.310483 | 5.611487 | 5.380564 | 4.219522 | 3.892011 |
| 4.055642 | 5.840214 | 3.450339 | 10.48195 | 17.49179 | 5.949182 |
| 7.203678 | 2.618083 | 5.874695 | 5.988513 | 6.739812 | 4.60286 |
| 3.149247 | 5.992238 | 4.207989 | 4.595556 | 24.59586 | 7.265418 |
| 6.244378 | 7.778807 | 8.792313 | 7.288596 | 23.9363 | 25.81404 |
| 5.813245 | 3.159068 | 13.20237 | 7.038424 | 16.76942 | 6.432441 |
| 6.413207 | 3.532253 | 9.386143 | 5.098794 | 7.488999 | 10.00153 |
| 4.45083 | 2.235866 | 3.541927 | 8.512965 | 5.653209 | 4.89891 |
| 14.96353 | 4.106241 | 4.144959 | 5.524678 | 4.047301 | 5.040224 |
| 5.780738 | 3.663046 | 62.34666 | 4.723688 | 5.305081 | 5.41207 |
| 2.839212 | 6.413461 | 5.995182 | 5.340068 | 11.88718 | 9.201279 |
| 6.540807 | 16.51406 | 38.98681 | 59.84644 | 25.94486 | 3.912384 |
| 6.465089 | 3.859621 | 5.792067 | 8.047113 | 4.988599 | 7.4646 |
| 4.702223 | 5.201938 | 20.37074 | 30.63691 | 11.91155 | 3.864697 |
| 3.767851 | 9.204883 | 6.270476 | 4.937456 | 27.77605 | 6.726001 |
| 3.498233 | 6.826147 | 8.483398 | 5.49388 | 4.515076 | 5.263221 |
| 2.695791 | 3.042089 | 29.24791 | 15.41544 | 4.810909 | 4.815952 |
| 7.207214 | 9.780593 | 2.759224 | 38.89029 | 7.629654 | 6.130703 |
| 5.993523 | 7.372277 | 4.939817 | 6.106998 | 12.21489 | 20.2127 |
| 5.70216 | 2.43126 | 6.455538 | 4.638684 | 14.77485 | 13.84875 |
| 15.56455 | 7.615608 | 22.02907 | 22.36194 | 6.192271 | 13.28209 |
| 2.312072 | 3.245922 | 10.04154 | 16.86234 | 6.898901 | 3.368993 |
| 3.247093 | 3.778426 | 10.22615 | 14.33572 | 11.91157 | 4.899499 |
| 5.025277 | 4.003801 | 3.297222 | 2.837573 | 9.019095 | 13.98449 |
| 4.691738 | 6.502602 | 10.15771 | 5.052948 | 3.953337 | 3.441571 |
| 3.991433 | 3.09009 | 22.88596 | 3.952734 | 3.481904 | 9.766563 |
| 3.487693 | 4.049957 | 5.956315 | 10.67901 | 5.14583 | 5.791276 |
| 3.487693 | 17.54503 | 5.445234 | 6.576869 | 2.889447 | 4.44717 |
| 7.810401 | 5.80759 | 2.821653 | 3.299389 | 5.968138 | 23.43869 |
| 3.170651 | 2.056036 | 2.882238 | 4.206872 | 3.506892 | 2.880833 |
| 3.432247 | 3.338966 | 16.75027 | 4.906305 | 4.425994 | 4.752355 |
| 10.81465 | 11.9482 | 4.520616 | 3.389528 | 15.45913 | 4.188067 |
| 3.342091 | 2.849822 | 7.72842 | 7.178779 | 8.13886 | 9.899613 |
| 4.152256 | 4.450214 | 6.591961 | 17.10455 | 17.88522 | 21.45097 |
| 3.821898 | 4.015094 | 5.131357 | 2.37878 | 9.310732 | 4.249927 |
| 4.391503 | 2.875093 | 17.05534 | 3.091671 | 37.71753 | 31.83961 |
| 4.084431 | 9.819323 | 5.103626 | 3.49873 | 3.557665 | 5.347838 |
| 2.977895 | 3.937736 | 26.896 | 3.227327 | 21.16319 | 17.44355 |
| 2.820548 | 6.498426 | 1.758303 | 9.789805 | 3.728936 | 3.504808 |
| 3.712028 | 6.07737 | 5.842261 | 7.570338 | 6.330469 | 17.41989 |
| 7.50415 | 3.281215 | 4.882935 | 3.602299 | 9.381261 | 14.824 |
| 6.87218 | 6.251493 | 3.320371 | 9.793236 | 4.548353 | 11.34139 |
| 3.672481 | 6.724485 | 1.437197 | 2.461304 | 9.937619 | 13.75421 |
| 6.511665 | 3.043493 | 7.282415 | 4.544334 | 10.3126 | 16.07472 |
| 4.532604 | 10.22191 | 5.035426 | 3.073501 | 12.99826 | 14.05999 |
| 7.817123 | 2.535881 | 3.881126 | 4.123766 | 13.36524 | 5.493281 |
| 5.429508 | 2.288817 | 6.44968 | 4.736034 | 34.22856 | 30.35499 |
| 4.515573 | 5.629223 | 14.93165 | 32.17725 | 9.084588 | 12.15449 |
| 4.707357 | 3.001875 | 3.084029 | 2.749446 | 13.88708 | 19.72115 |
| 7.597175 | 2.806723 | 24.08 | 3.372548 | 17.03631 | 15.73534 |
| 5.843291 | 3.606956 | 6.404175 | 18.93682 | 44.73362 | 25.74609 |
| 41.48041 | 3.329153 | 4.434602 | 5.135271 | 37.93594 | 51.60807 |
| 9.49891 | 4.112807 | 15.30805 | 2.841972 | 9.005071 | 4.119359 |
| 3.750686 | 3.350252 | 6.664538 | 3.539357 | 5.722256 | 15.73688 |
| 5.540888 | 4.344662 | 6.730703 | 4.853159 | 6.347635 | 8.081403 |
| 2.905558 | 3.798063 | 7.240685 | 23.69472 | 11.26002 | 3.141668 |
| 3.353715 | 2.195643 | 5.146069 | 14.79707 | 6.727789 | 4.781168 |
| 6.176332 | 10.1954 | 3.865368 | 4.870579 | 11.47671 | 13.47212 |
| 5.73714 | 17.4308 | 1.915428 | 2.265662 | 15.57796 | 7.823802 |
| 44.22567 | 12.27679 | 5.232976 | 3.571637 | 78.87098 | 12.70653 |
| 20.73592 | 2.541889 | 17.21086 | 7.987642 | 24.48509 | 6.522872 |
| 6.012979 | 3.74782 | 5.55772 | 3.406344 | 37.03443 | 7.192765 |
| 4.79398 | 3.567828 | 6.174628 | 6.863553 | 11.08696 | 5.003074 |
| 5.500957 | 7.328548 | 11.58953 | 43.79094 | 30.06758 | 5.09781 |
| 3.512461 | 3.625773 | 4.588564 | 15.28459 | 8.663286 | 12.64579 |
| 5.825376 | 6.380503 | 15.58979 | 4.822786 | 15.92917 | 6.109731 |
| 4.657805 | 4.718104 | 4.410421 | 6.582466 | 13.53032 | 13.86372 |
| 31.83797 | 39.01435 | 5.794946 | 3.931222 | 84.59837 | 4.271657 |
| 3.906773 | 3.987231 | 2.833885 | 4.625561 | 4.747602 | 11.83023 |
| 46.73039 | 78.02962 | 10.58551 | 2.175707 | 10.93416 | 4.560234 |
| 21.33045 | 8.669906 | 2.367634 | 2.401322 | 3.740109 | 49.47684 |
| 3.033509 | 3.009884 | 3.633836 | 3.207434 | 14.2929 | 12.68003 |
| 3.489828 | 4.382378 | 2.435094 | 3.237862 | 21.20553 | 13.35997 |
| 6.771817 | 7.671312 | 19.06842 | 51.95942 | 17.58685 | 9.431841 |
| 4.962059 | 6.45863 | 17.28538 | 9.167999 | 68.19137 | 85.23917 |
| 4.137944 | 4.741152 | 6.19425 | 10.7734 | 5.493583 | 30.05 |
| 4.456973 | 3.596986 | 7.206724 | 6.856003 | 114.4019 | 86.25416 |
| 13.15864 | 14.41895 | 9.946363 | 11.43671 | 11.76672 | 5.749672 |
| 6.912034 | 5.159563 | 3.565188 | 2.659144 | 3.971945 | 4.771506 |
| 4.04625 | 5.69948 | 11.13893 | 10.62977 | 13.77508 | 16.31792 |
| 5.632274 | 6.512489 | 5.406868 | 2.738696 | 13.35423 | 11.11019 |
| 13.27835 | 8.521687 | 4.377415 | 2.826052 | 7.760884 | 37.21235 |
| 10.65645 | 7.669886 | 2.83542 | 4.45457 | 24.59914 | 19.77784 |
| 11.65532 | 14.60717 | 21.02284 | 14.24137 | 8.742282 | 10.98608 |
| 5.677135 | 6.95629 | 4.637545 | 9.575703 | 4.841328 | 13.08233 |
| 10.86407 | 4.232699 | 6.606395 | 5.00497 | 20.04426 | 13.03326 |
| 4.939879 | 12.76199 | 10.74405 | 5.465244 | 32.43775 | 24.01525 |
| 3.717939 | 12.16879 | 16.56602 | 11.96305 | 10.83054 | 17.05446 |
| 4.019172 | 3.603963 | 8.765564 | 9.401584 | 4.389125 | 79.336 |
| 10.67367 | 6.384683 | 7.628095 | 11.53463 | 37.97546 | 46.09883 |
| 5.608814 | 5.097381 | 2.149609 | 11.11798 | 5.573921 | 13.07431 |
| 8.034668 | 5.652677 | 7.443069 | 11.46986 | 12.61612 | 2.988782 |
| 4.674448 | 8.119035 | 3.7811 | 2.893624 | 23.67298 | 29.80768 |
| 2.933528 | 6.075993 | 2.100406 | 14.61314 | 4.478416 | 2.702293 |
| 3.779349 | 4.249652 | 13.81498 | 12.10221 | 2.641719 | 4.716382 |
| 9.951559 | 3.91603 | 5.524758 | 46.01328 | 5.947605 | 11.01299 |
| 8.637225 | 2.508741 | 3.031031 | 10.50941 | 4.887423 | 4.466814 |
| 5.29186 | 6.27126 | 3.014808 | 9.896937 | 11.38371 | 15.61269 |
| 10.84915 | 5.741137 | 5.272357 | 4.636486 | 2.602443 | 63.31579 |
| 3.662755 | 4.79494 | 2.637599 | 2.589852 | 5.113162 | 2.895854 |
| 5.166384 | 5.926408 | 2.641214 | 10.41577 | 9.432882 | 6.238711 |
| 9.280945 | 4.035109 | 4.012137 | 2.690763 | 66.38895 | 50.07034 |
| 5.926085 | 6.116485 | 5.111076 | 4.141304 | 5.916684 | 3.825823 |
| 4.387254 | 7.653528 | 9.561972 | 39.22542 | 5.126392 | 9.33389 |
| 3.798937 | 3.419959 | 6.73466 | 6.812756 | 6.870531 | 6.927602 |
| 5.978378 | 4.865029 | 4.135839 | 4.929354 | 4.049976 | 5.16828 |
| 8.28274 | 5.056675 | 15.21087 | 23.66726 | 16.81418 | 13.23509 |
| 2.665828 | 6.470695 | 11.13072 | 3.184338 | 17.42984 | 19.07268 |
| 5.471937 | 5.450517 | 3.421441 | 0.03367846 | 53.72607 | 6.107775 |
| 5.545042 | 4.508639 | 3.797429 | 7.548313 | 12.82067 | 6.498844 |
| 6.895618 | 4.290335 | 3.545544 | 4.338082 | 10.51633 | 17.81157 |
| 5.949197 | 7.056271 | 2.952581 | 9.013952 | 19.05827 | 4.291412 |
| 9.80904 | 5.351337 | 9.969897 | 9.501262 | 6.382077 | 5.232518 |
| 7.01393 | 25.88634 | 3.306368 | 4.83678 | 0.13511 | 25.18352 |
| 6.770592 | 15.63265 | 2.81161 | 1.624034 | 27.48206 | 10.48665 |
| 4.143693 | 4.192303 | 12.32643 | 5.957664 | 7.632837 | 12.03543 |
| 8.106226 | 4.897307 | 3.948617 | 5.260802 | 34.50382 | 8.761678 |
| 5.371335 | 12.53464 | 4.608798 | 3.590684 | 7.040469 | 5.452338 |
| 15.86675 | 3.441421 | 6.708756 | 7.663614 | 6.161933 | 22.44845 |
| 5.66953 | 6.038749 | 9.979081 | 11.60632 | 10.83993 | 10.96555 |
| 5.157721 | 4.995753 | 9.744092 | 3.425441 | 6.016821 | 7.804584 |
| 9.432612 | 17.26562 | 32.19768 | 4.275489 | 3.681291 | 16.72644 |
| 6.241935 | 4.634354 | 3.526099 | 8.227035 | 36.21117 | 15.0125 |
| 16.07511 | 5.696563 | 0.02596993 | 6.232209 | 12.86901 | 12.85 |
| 5.785264 | 5.873406 | 2.263325 | 2.917897 | 154.2 | 154.4784 |
| 3.835927 | 3.22103 | 8.641303 | 4.226187 | 8.091263 | 4.323595 |
| 5.578598 | 5.984532 | 4.111251 | 3.252012 | 10.00031 | 13.50737 |
| 4.149332 | 5.287582 | 3.669015 | 3.280321 | 7.237647 | 6.837983 |
| 25.07848 | 2.238325 | 1.181272 | 7.939589 | 3.017884 | 15.56754 |
| 6.953586 | 5.856487 | 2.570337 | 3.718407 | 7.408208 | 10.9542 |
| 3.994372 | 11.89834 | 5.423892 | 5.504325 | 19.73924 | 10.91663 |
| 4.080151 | 5.385693 | 6.33233 | 6.026688 | 10.59285 | 14.88412 |
| 5.196534 | 6.265083 | 1.994472 | 3.647952 | 11.22419 | 7.909235 |
| 9.093566 | 6.373109 | 1.934708 | 3.443596 | 5.664762 | 4.313416 |
| 8.520721 | 3.590598 | 3.419422 | 3.447882 | 4.221482 | 5.704911 |
| 10.09129 | 4.379677 | 6.467288 | 2.440928 | 13.60713 | 20.40106 |
| 5.369148 | 4.159338 | 4.179129 | 6.570981 | 7.232738 | 9.269729 |
| 3.899227 | 9.595555 | 9.282991 | 4.285759 | 6.331585 | 3.416281 |
| 3.288833 | 7.907209 | 3.742463 | 13.67494 | 5.369593 | 4.38766 |
| 15.13825 | 4.459885 | 7.092382 | 21.86119 | 20.45162 | 20.34102 |
| 4.269036 | 8.640391 | 19.15493 | 9.436528 | 12.78741 | 5.138585 |
| 6.269823 | 4.683216 | 7.64037 | 11.58506 | 17.81077 | 19.31011 |
| 8.000979 | 4.432314 | 4.004523 | 6.468298 | 3.806243 | 8.885529 |
| 3.917735 | 10.98024 | 18.34915 | 2.829228 | 19.90426 | 30.68444 |
| 6.265456 | 3.712967 | 15.52836 | 4.036905 | 8.041727 | 7.000751 |
| 4.52019 | 8.924978 | 5.207326 | 8.882534 | 18.88853 | 22.23709 |
| 3.988838 | 21.9009 | 4.086136 | 6.329806 | 22.81209 | 5.984174 |
| 6.350046 | 18.38281 | 14.72635 | 11.87984 | 19.94348 | 8.633078 |
| 89.96603 | 5.295277 | 7.655752 | 4.36045 | 7.501607 | 7.48328 |
| 5.070765 | 4.036584 | 12.37389 | 4.218111 | 32.3808 | 32.3808 |
| 5.163982 | 8.562296 | 2.583326 | 4.158763 | 9.537981 | 13.524 |
| 4.570003 | 12.56135 | 2.768072 | 3.628298 | 5.448434 | 5.448434 |
| 6.357672 | 5.893093 | 7.930387 | 7.026806 | 58.68375 | 7.708711 |
| 13.92427 | 11.96758 | 8.1 | 5.558838 | 6.07751 | 3.806386 |
| 11.85363 | 4.578366 | 6.0648 | 6.115015 | 4.25506 | 2.54373 |
| 7.017491 | 10.93887 | 32.20329 | 4.72346 | 4.264263 | 2.93245 |
| 8.189673 | 5.224471 | 13.83882 | 7.798813 | 5.751704 | 5.751704 |
| 4.297643 | 3.315405 | 7.199009 | 4.903147 | 2.717202 | 4.453604 |
| 3.127875 | 5.724769 | 5.775777 | 3.635223 | 35.37894 | 16.88202 |
| 3.757701 | 6.143419 | 5.255344 | 5.2136 | 3.504549 | 6.756011 |
| 4.590179 | 4.172038 | 28.88483 | 26.6 | 7.039915 | 9.683589 |
| 4.75446 | 5.878282 | 12.236 | 12.47639 | 14.9209 | 8.167825 |
| 3.59537 | 6.399356 | 7.255353 | 4.070926 | 8.48786 | 29.02679 |
| 8.617045 | 6.71925 | 15.86093 | 9.30739 | 6.755234 | 9.760057 |
| 5.078771 | 15.16304 | 3.795037 | 6.123106 | 4.956803 | 4.704326 |
| 3.887242 | 3.432223 | 20.92921 | 19.47975 | 7.896566 | 11.62267 |
| 4.366198 | 3.123881 | 3.733803 | 2.794445 | 43.4464 | 3.602793 |
| 3.134444 | 5.365015 | 8.550655 | 25.13509 | 12.71407 | 3.276715 |
| 29.83677 | 3.795134 | 64.45506 | 29.83055 | 10.89686 | 4.149221 |
| 8.882443 | 4.545887 | 41.0285 | 7.079262 | 6.657761 | 5.883791 |
| 5.52092 | 3.629859 | 20.21274 | 44.82039 | 6.851784 | 13.75165 |
| 3.265674 | 4.12508 | 6.24144 | 10.39866 | 9.59308 | 10.3684 |
| 7.111586 | 6.171921 | 7.029541 | 8.174974 | 9.29912 | 5.17719 |
| 7.370396 | 3.503419 | 4.161457 | 11.43728 | 109.8477 | 48.48935 |
| 3.504621 | 6.132917 | 25.90572 | 6.935902 | 54.54206 | 46.22205 |
| 5.996718 | 3.737048 | 21.35663 | 4.706857 | 9.540025 | 8.648765 |
| 8.808705 | 7.46481 | 4.764867 | 13.02634 | 20.2055 | 4.858531 |
| 3.983127 | 4.082361 | 21.22981 | 21.08062 | 47.27679 | 44.69118 |
| 9.837594 | 9.783865 | 7.572389 | 4.339006 | 26.18329 | 11.1011 |
| 4.638844 | 4.010495 | 3.866492 | 4.413522 | 5.53112 | 5.563395 |
| 12.03892 | 8.600803 | 4.616856 | 4.253553 | 10.74993 | 11.33151 |
| 15.20701 | 4.67536 | 5.749688 | 29.13366 | 9.576052 | 20.13488 |
| 7.209854 | 7.145574 | 29.68288 | 10.13905 | 55.91184 | 86.40356 |
| 6.443994 | 9.485418 | 12.73736 | 14.49218 | 18.91011 | 183.4874 |
| 3.950952 | 4.722171 | 13.15228 | 7.214735 | 24.17099 | 33.864 |
| 9.583839 | 3.994048 | 13.3 | 11.66962 | 45.18219 | 60.96828 |
| 4.4227 | 7.745134 | 4.424279 | 5.929689 | 24.52 | 20.11476 |
| 4.518932 | 12.21135 | 5.546093 | 13.16288 | 8.886425 | 7.250155 |
| 5.057417 | 3.611681 | 6.509099 | 5.114682 | 5.187289 | 5.725898 |
| 4.890999 | 5.060022 | 19.71285 | 3.796072 | 3.841988 | 3.7207 |
| 7.459964 | 3.479877 | 4.287576 | 5.656683 | 10.2165 | 7.075255 |
| 14.57091 | 2.936452 | 18.48471 | 15.25183 | 7.578993 | 28.59837 |
| 44.56948 | 6.675977 | 6.990124 | 7.827027 | 5.14 | 4.191802 |
| 5.784246 | 4.491576 | 10.08475 | 9.035424 | 9.48 | 18.30013 |
| 5.884908 | 8.06465 | 2.758242 | 4.226728 | 4.74 | 3.917882 |
| 13.49087 | 3.440389 | 4.004 | 4.113102 | 11.85 | 13.90507 |
| 5.404862 | 13.61149 | 5.686054 | 10.97139 | 13.3557 | 14.22 |
| 4.800424 | 6.031175 | 19.2192 | 19.23204 | 3.792 | 11.85 |
| 3.568101 | 4.80412 | 21.8032 | 14.81395 | 7.11 | 7.66239 |
| 3.282156 | 4.190273 | 37.4794 | 20.50839 | 26.01954 | 13.59708 |
| 14.96267 | 4.600403 | 26.5142 | 12.96206 | 6.162 | 7.470978 |
| 13.72325 | 4.028592 | 11.6208 | 9.829953 | 5.169583 | 5.358952 |
| 75.46032 | 3.220932 | 7.627536 | 5.490342 | 3.6972 | 3.696399 |
| 5.606713 | 6.361741 | 6.639006 | 6.100737 | 6.039126 | 6.160745 |
| 2.944464 | 4.632361 | 3.597325 | 6.398995 | 5.739353 | 3.5957 |
| 12.51294 | 4.014754 | 7.097958 | 10.88577 | 3.168263 | 3.847341 |
| 43.40393 | 5.928788 | 4.786731 | 7.552636 | 3.966588 | 9.618905 |
| 13.2431 | 6.919051 | 8.620864 | 10.50433 | 21.67319 | 15.88725 |
| 7.254133 | 10.91076 | 15.36335 | 6.599765 | 8.622086 | 4.881338 |
| 13.31503 | 3.241769 | 3.991398 | 9.567559 | 3.151983 | 8.865116 |
| 3.058811 | 13.2724 | 4.347585 | 7.664616 | 23.64 | 24.98174 |
| 7.519434 | 13.77332 | 41.12809 | 67.62939 | 12.8362 | 12.56502 |
| 14.23121 | 8.861746 | 11.128 | 16.692 | 7.171813 | 13.87348 |
| 3.712251 | 3.903898 | 18.74812 | 14.37359 | 6.656 | 6.144 |
| 12.91703 | 30.9318 | 15.3626 | 16.40274 | 5.236561 | 20.86978 |
| 6.579211 | 3.198911 | 6.290041 | 6.290041 | 18.69218 | 11.50886 |
| 4.464413 | 3.13072 | 10.68853 | 21.78073 | 8.704001 | 10.24 |
| 7.042887 | 21.19782 | 9.072204 | 6.768936 | 3.584 | 3.584 |
| 28.88652 | 7.725965 | 3.991815 | 6.310985 | 23.03568 | 15.26074 |
| 5.767667 | 4.453608 | 50.2711 | 64.61509 | 19.04435 | 7.442978 |
| 8.38583 | 10.97357 | 7.371676 | 7.428291 | 11.38558 | 18.792 |
| 5.760418 | 3.784977 | 3.28631 | 3.28276 | 6.793417 | 9.580861 |
| 5.296046 | 5.384765 | 2.955358 | 4.312069 | 17.32053 | 11.27 |
| 4.910099 | 8.854219 | 6.762891 | 2.474656 | 7.922125 | 17.48662 |
| 6.640303 | 7.820976 | 9.94489 | 4.880774 | 8.38488 | 8.403748 |
| 5.18449 | 5.00702 | 129.189 | 121.6882 | 4.532374 | 16.62851 |
| 7.328394 | 11.89704 | 8.346 | 8.304685 | 11.27 | 12.66596 |
| 4.740923 | 3.547658 | 74.96657 | 81.99937 | 0.142425 | 9.575036 |
| 18.67084 | 7.813263 | 3.410299 | 12.17714 | 4.508 | 5.4096 |
| 3.727305 | 3.775178 | 98.44972 | 16.41326 | 14.72542 | 11.10563 |
| 9.884307 | 33.22493 | 5.0076 | 4.949235 | 18.8176 | 19.14767 |
| 7.249674 | 4.658245 | 60.97582 | 21.72611 | 7.428 | 4.952 |
| 3.363778 | 6.690426 | 7.665493 | 7.2332 | 31.06228 | 21.41865 |
| 16.1199 | 2.926539 | 4.33634 | 5.009278 | 29.712 | 36.60004 |
| 20.27212 | 18.42923 | 6.486368 | 5.095561 | 29.75561 | 29.2168 |
| 6.927127 | 7.833258 | 4.580742 | 3.523669 | 15.05571 | 17.6361 |
| 8.360592 | 17.52849 | 6.240781 | 10.57871 | 8.71552 | 8.743033 |
| 5.705536 | 7.742287 | 15.60663 | 16.692 | 17.54436 | 4.680206 |
| 3.146782 | 8.225352 | 14.141 | 11.128 | 4.566013 | 30.85726 |
| 3.146782 | 7.975917 | 4.070293 | 3.993383 | 12.47654 | 4.28235 |
| 7.818881 | 4.406194 | 9.048935 | 7.307633 | 5.635744 | 14.22 |
| 12.69541 | 3.222716 | 3.626447 | 5.625251 | 3.089788 | 4.74 |
| 53.58749 | 7.614212 | 3.085549 | 13.75978 | 18.96 | 20.74789 |
| 17.83091 | 104.6515 | 3.281679 | 27.80982 | 6.057134 | 7.11 |
| 18.40113 | 4.639362 | 6.424884 | 8.204018 | 3.754936 | 8.037606 |
| 8.768107 | 6.162014 | 38.23961 | 8.525408 | 4.353314 | 7.830717 |
| 63.81644 | 7.096842 | 49.456 | 55.104 | 10.84294 | 16.94287 |
| 7.781814 | 4.172075 | 49.456 | 16.26115 | 2.54898 | 9.350424 |
| 5.997297 | 13.97726 | 28.4372 | 26.0116 | 4.74 | 3.345671 |
| 56.5883 | 15.44958 | 20.62954 | 36.92969 | 8.482795 | 13.58958 |
| 8.332534 | 3.3037 | 10.78079 | 14.30354 | 7.837297 | 6.02 |
| 6.264312 | 2.970249 | 68.79215 | 8.87355 | 2.999065 | 3.816738 |
| 9.456793 | 4.634175 | 50.60307 | 50.0908 | 9.73177 | 9.34469 |
| 24.19925 | 16.61072 | 3.100131 | 5.164 | 3.355892 | 3.461377 |
| 4.554161 | 19.45523 | 5.103691 | 15.52104 | 14.95881 | 13.51731 |
| 15.14759 | 3.820508 | 4.8347 | 5.020266 | 8.007101 | 13.15149 |
| 7.498356 | 4.321276 | 5.606859 | 9.157009 | 4.934728 | 4.19915 |
| 6.993415 | 4.449892 | 3.903974 | 3.674103 | 9.456 | 14.184 |
| 6.178982 | 6.634039 | 6.7132 | 7.075107 | 11.82 | 12.50466 |
| 21.94665 | 20.26827 | 10.70665 | 4.813219 | 25.6 | 32.43051 |
| 3.19818 | 6.362115 | 71.2632 | 71.57832 | 12.7661 | 19.23603 |
| 6.429802 | 8.625274 | 86.2388 | 84.76623 | 8.786527 | 6.290881 |
| 4.577478 | 8.587559 | 22.88379 | 18.59237 | 16.35221 | 16.44924 |
| 3.720988 | 12.85029 | 5.926877 | 7.885232 | 6.974611 | 6.048395 |
| 5.104885 | 3.458195 | 11.3608 | 12.30887 | 3.654334 | 3.798161 |
| 61.44449 | 3.348824 | 6.485462 | 46.18576 | 20.88 | 15.66 |
| 15.10847 | 5.063378 | 16.95622 | 5.259387 | 5.22 | 10.44 |
| 3.616411 | 12.57291 | 3.607636 | 3.332656 | 14.41778 | 19.04811 |
| 16.98287 | 19.89634 | 8.2624 | 8.311408 | 36.064 | 38.00362 |
| 12.02279 | 18.93575 | 8.073169 | 7.276958 | 27.24566 | 19.03267 |
| 9.355913 | 4.932835 | 16.17388 | 5.931774 | 18.032 | 14.80862 |
| 32.14264 | 11.64017 | 30.43753 | 19.46 | 29.0815 | 9.382502 |
| 4.39899 | 4.662263 | 13.59631 | 14.17169 | 35.942 | 11.81747 |
| 11.02491 | 7.98428 | 48.3608 | 35.26403 | 3.519518 | 2.957373 |
| 51.69981 | 4.391029 | 5.093469 | 2.417088 | 18.16965 | 6.90096 |
| 9.571022 | 24.01802 | 17.05418 | 19.08188 | 4.862372 | 3.644279 |
| 7.47169 | 8.026252 | 25.21568 | 11.676 | 13.11208 | 33.81029 |
| 18.20335 | 16.53658 | 58.38 | 60.46284 | 27.18652 | 27.18648 |
| 54.92893 | 11.60773 | 20.43105 | 22.5736 | 15.8464 | 14.856 |
| 3.625744 | 3.494881 | 17.87284 | 2.680429 | 14.856 | 14.856 |
| 16.60115 | 4.359837 | 7.85733 | 3.250883 | 24.76 | 19.808 |
| 5.40742 | 5.865931 | 76.6724 | 76.75679 | 38.06233 | 32.96919 |
| 16.89458 | 7.320373 | 12.8436 | 13.07032 | 37.82608 | 33.40788 |
| 4.520723 | 6.227544 | 5.928894 | 18.936 | 12.45977 | 5.140955 |
| 6.123772 | 6.145288 | 4.6704 | 5.266404 | 9.823058 | 18.17738 |
| 7.139361 | 5.044764 | 5.332863 | 5.33204 |  |  |
| 8.586899 | 3.626135 | 15.71512 | 14.46456 |  |  |
| 2.900073 | 4.288296 | 4.116452 | 3.345503 |  |  |
| 2.864314 | 4.252208 | 4.065777 | 6.044958 |  |  |
| 3.941006 | 6.266698 | 3.34918 | 29.94749 |  |  |
| 49.96193 | 8.959811 | 3.113218 | 2.300613 |  |  |
| 37.64208 | 3.23674 | 4.069336 | 4.123367 |  |  |
| 13.48265 | 4.678938 | 7.772616 | 7.476484 |  |  |
| 17.03128 | 6.56987 | 3.418872 | 6.676693 |  |  |
| 5.941634 | 6.90417 | 9.992296 | 14.65672 |  |  |
| 4.480546 | 8.938427 | 2.088464 | 4.390992 |  |  |
| 4.577491 | 4.311442 | 3.289236 | 3.19699 |  |  |
| 7.778326 | 6.786838 | 20.99034 | 6.224485 |  |  |
| 3.364701 | 4.526818 | 22.06056 | 10.12135 |  |  |
| 3.622392 | 7.993213 | 9.043131 | 16.62545 |  |  |
| 5.240562 | 2.945315 | 13.42363 | 10.8 |  |  |
| 0.09522608 | 4.03246 | 41.08545 | 26.6 |  |  |
| 4.901187 | 10.02595 | 25.40248 | 5.209383 |  |  |
| 5.63366 | 3.093299 | 12.66254 | 8.045919 |  |  |
| 27.40097 | 19.59023 | 3.886573 | 20.13095 |  |  |
| 19.52753 | 23.55227 | 4.522 | 3.825091 |  |  |
| 11.711 | 11.72089 | 7.939446 | 8.583413 |  |  |
| 39.12138 | 19.088 | 10.64 | 7.875891 |  |  |
| 57.75461 | 14.85414 | 13.3 | 5.056262 |  |  |
| 7.923483 | 3.602168 | 6.735438 | 5.045227 |  |  |
| 4.151923 | 5.178053 | 10.64 | 4.97621 |  |  |
| 26.62657 | 19.30971 | 5.722635 | 10.21189 |  |  |
| 4.427084 | 37.37927 | 4.119083 | 4.62213 |  |  |
| 38.8207 | 4.887051 | 26.64891 | 4.004 |  |  |
| 50.43554 | 79.25063 | 6.8068 | 6.006 |  |  |
| 4.817661 | 13.27673 | 12.012 | 9.122757 |  |  |
| 7.776881 | 8.30486 | 13.14185 | 3.033096 |  |  |
| 3.870275 | 4.951323 | 12.012 | 12.012 |  |  |
| 4.263403 | 3.931956 | 26.34067 | 5.55144 |  |  |
| 4.544561 | 7.193595 | 7.1766 | 6.343339 |  |  |
| 7.397459 | 6.777876 | 18.67493 | 35.96724 |  |  |
| 11.6776 | 4.785252 | 17.2484 | 6.723105 |  |  |
| 15.92384 | 6.627901 | 11.128 | 9.4588 |  |  |
| 2.904337 | 3.456079 | 66.05254 | 13.31607 |  |  |
| 5.378879 | 7.351102 | 29.2672 | 20.5868 |  |  |
| 23.66534 | 5.008796 | 10.21198 | 20.9872 |  |  |
| 8.505718 | 3.664286 | 17.50273 | 5.164511 |  |  |
| 15.83019 | 3.87235 | 9.4588 | 17.27721 |  |  |
| 16.47956 | 3.093088 | 27.82 | 25.37804 |  |  |
| 8.765643 | 9.675689 | 8.346 | 5.564 |  |  |
| 8.505023 | 5.48117 | 4.594518 | 4.4512 |  |  |
| 2.37129 | 3.211751 | 11.128 | 10.97474 |  |  |
| 7.366918 | 6.191027 | 19.92954 | 6.646068 |  |  |
| 3.934615 | 4.615782 | 10.70946 | 16.692 |  |  |
| 12.19393 | 4.708207 | 12.92331 | 6.773998 |  |  |
| 4.991903 | 11.6345 | 5.514114 | 5.530314 |  |  |
| 7.564808 | 5.79612 | 12.2408 | 11.20404 |  |  |
| 5.317897 | 7.869236 | 4.760377 | 11.93251 |  |  |
| 57.41393 | 3.256766 | 31.48128 | 9.603522 |  |  |
| 3.187746 | 8.921408 | 19.1068 | 15.492 |  |  |
| 2.266253 | 1.735955 | 3.56573 | 9.140196 |  |  |
| 3.553876 | 5.184891 | 97.84954 | 41.27969 |  |  |
| 3.166313 | 2.624405 | 13.04477 | 14.84933 |  |  |
| 3.596329 | 7.666827 | 15.39263 | 5.536371 |  |  |
| 5.251566 | 5.32695 | 15.38806 | 15.1788 |  |  |
| 3.170763 | 3.305399 | 14.39648 | 10.11116 |  |  |
| 3.04831 | 2.391605 | 7.049325 | 5.553087 |  |  |
| 5.088977 | 8.976 | 5.745909 | 5.924538 |  |  |
| 7.923492 | 5.982597 | 2.896732 | 3.13503 |  |  |
| 38.28894 | 7.833583 | 12.6638 | 12.07794 |  |  |
| 28.47043 | 18.90052 | 13.3 | 12.09708 |  |  |
| 11.07071 | 5.911319 | 12.16622 | 17.30107 |  |  |
| 11.00985 | 19.04918 | 4.601426 | 5.137054 |  |  |
| 13.6857 | 5.103043 | 41.25039 | 7.506638 |  |  |
| 6.098674 | 3.732049 | 4.707246 | 4.848312 |  |  |
| 3.459802 | 3.760651 | 6.735438 | 4.560528 |  |  |
| 4.0392 | 3.873125 | 5.214302 | 3.636242 |  |  |
| 5.625951 | 12.94721 | 14.84863 | 13.3 |  |  |
| 6.707875 | 4.369463 | 11.172 | 10.64 |  |  |
| 11.0373 | 7.130264 | 8.778 | 8.540434 |  |  |
| 25.10037 | 2.925928 | 8.8088 | 14.41542 |  |  |
| 6.099906 | 3.55437 | 3.356972 | 3.990588 |  |  |
| 11.79258 | 129.6549 | 5.0058 | 4.058727 |  |  |
| 4.189956 | 3.456409 | 4.948689 | 3.622042 |  |  |
| 32.82163 | 17.69817 | 33.94444 | 12.012 |  |  |
| 8.976 | 6.3695 | 35.952 | 29.96 |  |  |
| 8.263522 | 3.393793 | 21.44567 | 16.692 |  |  |
| 4.524858 | 10.11354 | 15.617 | 13.88903 |  |  |
| 19.32762 | 16.13506 | 45.68207 | 8.346 |  |  |
| 5.488419 | 5.623337 | 16.692 | 10.36996 |  |  |
| 3.881476 | 3.391397 | 25.038 | 18.71981 |  |  |
| 2.58386 | 10.91769 | 14.453 | 3.402515 |  |  |
| 6.063435 | 4.895155 | 18.36223 | 20.73589 |  |  |
| 6.979166 | 9.85 | 5.564 | 13.91 |  |  |
| 7.675078 | 2.899123 | 38.61268 | 28.9328 |  |  |
| 9.621656 | 14.90416 | 13.91 | 13.91 |  |  |
| 13.67922 | 30.95645 | 11.02592 | 6.547778 |  |  |
| 3.518389 | 5.74527 | 10.18193 | 4.841527 |  |  |
| 56.18448 | 4.855779 | 27.82 | 16.692 |  |  |
| 31.75047 | 9.740287 | 8.139148 | 6.511605 |  |  |
| 7.156214 | 3.862456 | 8.139529 | 5.110605 |  |  |
| 2.825372 | 2.870694 | 2.57261 | 7.793439 |  |  |
| 4.61443 | 4.437554 | 16.31826 | 6.164998 |  |  |
| 12.75397 | 14.49131 | 25.82 | 6.7132 |  |  |
| 6.617643 | 5.250884 | 19.50028 | 15.492 |  |  |
| 3.827943 | 11.76404 | 40.05077 | 7.203349 |  |  |
| 10.12907 | 6.258704 | 46.70561 | 50.94439 |  |  |
| 7.5265 | 7.312989 | 7.853063 | 8.26978 |  |  |
| 0.07177298 | 4.825749 | 14.47991 | 11.83824 |  |  |
| 4.772245 | 8.071497 | 25.20143 | 23.352 |  |  |
| 2.85507 | 5.931703 | 3.867625 | 12.18983 |  |  |
| 10.04119 | 3.573266 | 20.60898 | 5.188789 |  |  |
| 16.016 | 3.259974 | 8.887937 | 4.170428 |  |  |
| 30.60171 | 25.6348 | 29.08055 | 7.101987 |  |  |
| 5.405274 | 5.162124 | 12.28962 | 3.361265 |  |  |
| 17.46049 | 8.482033 | 3.852592 | 4.096106 |  |  |
| 19.42763 | 10.3851 | 11.99538 | 9.370854 |  |  |
| 3.40428 | 1.966017 | 11.89695 | 4.912131 |  |  |
| 4.424554 | 4.03866 | 16.12297 | 6.857477 |  |  |
| 65.38636 | 64.25839 | 4.992023 | 18.15071 |  |  |
| 3.590609 | 3.813332 | 5.852017 | 3.841602 |  |  |
| 4.123029 | 6.648582 | 39.38412 | 10.19064 |  |  |
| 4.553813 | 17.107 | 6.992213 | 3.345327 |  |  |
| 10.352 | 9.020862 | 2.477729 | 4.074068 |  |  |
| 14.64145 | 15.73847 | 2.356633 | 5.332583 |  |  |
| 18.06204 | 7.613011 | 7.468687 | 3.977735 |  |  |
| 5.721161 | 5.04 | 7.714843 | 5.955289 |  |  |
| 8.376062 | 7.498801 | 34.63702 | 2.60294 |  |  |
| 5.798172 | 4.354468 | 36.036 |  |  |  |
| 3.796241 | 4.468767 | 3.797916 | 4.718691 |  |  |
| 7.09309 | 16.10415 | 3.385752 | 5.288513 |  |  |
| 7.730821 | 6.595573 | 32.50865 | 5.2052 |  |  |
| 5.621686 | 7.172278 | 6.819384 | 5.875989 |  |  |
| 6.9048 | 6.934726 | 15.89016 | 3.677031 |  |  |
| 19.75756 | 6.899801 | 7.406056 | 11.61241 |  |  |
| 4.629084 | 7.405317 | 14.77006 | 4.004 |  |  |
| 6.294513 | 6.002146 | 12.9474 | 4.7936 |  |  |
| 6.838861 | 3.391815 | 7.135694 | 4.356882 |  |  |
| 4.346194 | 4.636661 | 6.75713 | 4.4864 |  |  |
| 7.407705 | 3.30513 | 8.070291 | 5.608 |  |  |
| 24.62118 | 4.458845 | 6.735658 | 9.089486 |  |  |
| 7.556012 | 13.2425 | 21.2059 | 5.95348 |  |  |
| 5.716094 | 5.365832 | 28.78667 | 8.207667 |  |  |
| 11.53209 | 4.318534 | 7.288966 | 7.838838 |  |  |
| 4.505009 | 4.842427 | 5.519564 | 63.15979 |  |  |
| 9.148167 | 3.538846 | 29.25994 | 12.33455 |  |  |
| 9.560942 | 5.147232 | 16.53374 | 5.164 |  |  |
| 6.099583 | 4.929928 | 5.985851 | 34.80087 |  |  |
| 10.19533 | 15.47213 | 35.3073 | 11.61046 |  |  |
| 4.759535 | 6.214883 | 4.47136 | 5.73204 |  |  |
| 5.818312 | 7.214575 | 10.328 | 5.265714 |  |  |
| 4.64007 | 17.07111 | 3.40665 | 4.140615 |  |  |
| 4.758364 | 3.504515 | 6.09739 | 3.816679 |  |  |
| 13.82356 | 4.490898 | 4.415764 | 3.21106 |  |  |
| 13.44186 | 4.35064 | 20.60898 | 4.05733 |  |  |
| 31.85034 | 6.731202 | 8.887937 | 3.59922 |  |  |
| 8.598788 | 4.559427 | 29.08055 | 4.286955 |  |  |
| 31.48333 | 8.271092 | 12.28962 | 11.81709 |  |  |
| 39.11465 | 21.01715 | 3.852592 | 2.651256 |  |  |
| 112.2198 | 29.75424 | 11.99538 | 4.165768 |  |  |
| 9.748828 | 9.666403 | 11.89695 | 4.986564 |  |  |
| 6.580091 | 8.706525 | 16.12297 | 6.384 |  |  |
| 64.50076 | 7.843994 | 4.992023 | 3.392376 |  |  |
| 14.34 | 11.5185 | 5.852017 | 3.745255 |  |  |
| 3.090047 | 4.648118 | 39.38412 | 10.64 |  |  |
| 143.4 | 33.64764 | 6.992213 | 3.048203 |  |  |
| 24.30853 | 3.517688 | 2.477729 | 2.877418 |  |  |
| 16.77349 | 17.10041 | 2.356633 | 5.32532 |  |  |
| 7.351032 | 6.882964 | 7.468687 | 8.008 |  |  |
| 26.48271 | 10.09548 | 7.714843 | 6.579959 |  |  |
| 8.761492 | 2.892868 | 34.63702 | 4.97277 |  |  |
| 20.97755 | 13.09262 | 36.036 | 4.004 |  |  |
| 2.971794 | 6.578024 | 3.797916 | 7.329263 |  |  |
| 3.574954 | 5.348691 | 3.385752 | 8.070436 |  |  |
| 54.21898 | 9.862228 | 32.50865 | 5.199735 |  |  |
| 6.703867 | 3.392416 | 6.819384 | 3.839462 |  |  |
| 19.12 | 7.859213 | 15.89016 | 4.970326 |  |  |
| 5.365467 | 10.54362 | 7.406056 | 3.647673 |  |  |
| 28.632 | 16.36223 | 14.77006 | 4.295191 |  |  |
| 9.742657 | 2.700847 | 12.9474 | 5.600474 |  |  |
| 5.680687 | 3.444495 | 7.135694 | 3.758048 |  |  |
| 12.32811 | 4.3238 | 6.75713 | 5.096151 |  |  |
| 15.42549 | 44.79027 | 8.070291 | 8.412 |  |  |
| 7.842874 | 14.74491 | 6.735658 | 3.976584 |  |  |
| 9.783489 | 84.11727 | 21.2059 | 5.976844 |  |  |
| 31.84036 | 5.313765 | 28.78667 | 6.427996 |  |  |
| 3.420384 | 7.158697 | 7.288966 | 5.294951 |  |  |
| 9.090735 | 4.710096 | 5.519564 | 3.581263 |  |  |
| 24.71876 | 4.210667 | 29.25994 | 22.64503 |  |  |
| 15.852 | 8.394235 | 16.53374 | 7.746 |  |  |
| 27.73023 | 3.393939 | 5.985851 | 2.223855 |  |  |
| 12.89952 | 5.284 | 35.3073 | 16.80908 |  |  |
| 6.545989 | 4.173647 | 4.47136 | 5.746884 |  |  |
| 12.72641 | 17.80051 | 10.328 | 3.050666 |  |  |
| 2.243803 | 12.98936 | 3.40665 | 3.674036 |  |  |
| 8.888236 | 2.736524 | 6.09739 | 5.225085 |  |  |
| 2.525815 | 5.066653 | 4.415764 | 1.914916 |  |  |
| 3.112362 | 4.768945 |  |  |  |  |
| 5.033709 | 5.473256 |  |  |  |  |
| 8.976 | 8.08745 |  |  |  |  |
| 4.009582 | 4.488 |  |  |  |  |
| 8.714693 | 7.408699 |  |  |  |  |
| 3.612712 | 3.307441 |  |  |  |  |
| 5.240501 | 5.6099 |  |  |  |  |
| 9.703269 | 10.15673 |  |  |  |  |
| 6.732 | 4.488 |  |  |  |  |
| 13.464 | 21.79637 |  |  |  |  |
| 12.012 | 4.004 |  |  |  |  |
| 25.2 | 9.741592 |  |  |  |  |
| 20.7931 | 3.138221 |  |  |  |  |
| 132.3983 | 17.37075 |  |  |  |  |
| 8.754891 | 10.43819 |  |  |  |  |
| 9.045466 | 7.496226 |  |  |  |  |
| 2.910122 | 2.861132 |  |  |  |  |
| 14.21413 | 12.6 |  |  |  |  |
| 46.76956 | 7.56 |  |  |  |  |
| 13.63115 | 3.492614 |  |  |  |  |
| 26.85452 | 10.288 |  |  |  |  |
| 52.58191 | 10.40113 |  |  |  |  |
| 18.35122 | 4.65574 |  |  |  |  |
| 64.50076 | 7.843994 |  |  |  |  |
| 14.34 | 4.78 |  |  |  |  |
| 3.090047 | 9.785197 |  |  |  |  |
| 143.4 | 53.85744 |  |  |  |  |
| 24.30853 | 24.99617 |  |  |  |  |
| 16.77349 | 17.08501 |  |  |  |  |
| 7.351032 | 4.06744 |  |  |  |  |
| 26.48271 | 7.613417 |  |  |  |  |
| 8.761492 | 4.063668 |  |  |  |  |
| 20.97755 | 2.838805 |  |  |  |  |
| 2.971794 | 8.381334 |  |  |  |  |
| 3.574954 | 12.39965 |  |  |  |  |
| 54.21898 | 10.84786 |  |  |  |  |
| 6.703867 | 2.921046 |  |  |  |  |
| 19.12 | 5.947627 |  |  |  |  |
| 5.365467 | 12.95981 |  |  |  |  |
| 28.632 | 7.018693 |  |  |  |  |
| 9.742657 | 8.425139 |  |  |  |  |
| 5.680687 | 4.772 |  |  |  |  |
| 12.32811 | 13.57218 |  |  |  |  |
| 15.42549 | 48.06125 |  |  |  |  |
| 7.842874 | 15.0104 |  |  |  |  |
| 9.783489 | 7.428136 |  |  |  |  |
| 31.84036 | 17.22927 |  |  |  |  |
| 3.420384 | 14.27351 |  |  |  |  |
| 9.090735 | 5.284 |  |  |  |  |
| 24.71876 | 5.97768 |  |  |  |  |
| 15.852 | 6.150343 |  |  |  |  |
| 27.73023 | 2.274845 |  |  |  |  |
| 12.89952 | 10.568 |  |  |  |  |
| 6.545989 | 7.960318 |  |  |  |  |
| 12.72641 | 5.057739 |  |  |  |  |
| 2.243803 | 9.777769 |  |  |  |  |
| 8.888236 | 1.89738 |  |  |  |  |
| 2.525815 | 3.896943 |  |  |  |  |
| 3.112362 | 4.434965 |  |  |  |  |
| 5.033709 | 3.70784 |  |  |  |  |
| 8.976 | 2.617738 |  |  |  |  |
| 4.009582 | 4.568082 |  |  |  |  |
| 8.714693 | 3.179187 |  |  |  |  |
| 3.612712 | 3.111719 |  |  |  |  |
| 5.240501 | 2.751357 |  |  |  |  |
| 9.703269 | 3.982452 |  |  |  |  |
| 6.732 | 3.95358 |  |  |  |  |
| 13.464 | 9.300525 |  |  |  |  |
| 12.012 | 3.2032 |  |  |  |  |
| 25.2 | 6.552 |  |  |  |  |
| 20.7931 | 13.85742 |  |  |  |  |
| 132.3983 | 30.85322 |  |  |  |  |
| 8.754891 | 4.032 |  |  |  |  |
| 9.045466 | 3.462605 |  |  |  |  |
| 2.910122 | 31.12446 |  |  |  |  |
| 14.21413 | 4.756877 |  |  |  |  |
| 46.76956 | 10.08 |  |  |  |  |
| 13.63115 | 3.240703 |  |  |  |  |
| 26.85452 | 5.534364 |  |  |  |  |
| 52.58191 | 13.16848 |  |  |  |  |
| 18.35122 | 20.03212 |  |  |  |  |
| 5.48058 | 9.174884 |  |  |  |  |
| 11.15447 | 16.14564 |  |  |  |  |
| 10.33178 | 11.74629 |  |  |  |  |
| 22.51554 | 3.089057 |  |  |  |  |
| 9.593803 | 2.988967 |  |  |  |  |
| 29.01124 | 21.95503 |  |  |  |  |
| 20.91443 | 4.684126 |  |  |  |  |
| 24.73044 | 84.65289 |  |  |  |  |
| 44.73753 | 7.399218 |  |  |  |  |
| 39.33306 | 4.482 |  |  |  |  |
| 7.232731 | 7.47 |  |  |  |  |
| 12.63974 | 7.092559 |  |  |  |  |
| 3.689465 | 5.3136 |  |  |  |  |
| 24.73606 | 2.595618 |  |  |  |  |
| 70.9991 | 35.904 |  |  |  |  |
| 6.63203 | 3.200411 |  |  |  |  |
| 21.33088 | 8.0784 |  |  |  |  |
| 10.79433 | 6.780121 |  |  |  |  |
| 3.166266 | 4.0392 |  |  |  |  |
| 7.363545 | 4.488 |  |  |  |  |
| 8.80104 | 4.401052 |  |  |  |  |
| 17.7456 | 6.590577 |  |  |  |  |
| 5.48058 | 8.604 |  |  |  |  |
| 11.15447 | 7.582699 |  |  |  |  |
| 10.33178 | 28.71517 |  |  |  |  |
| 22.51554 | 3.807033 |  |  |  |  |
| 9.593803 | 4.111547 |  |  |  |  |
| 29.01124 | 10.626 |  |  |  |  |
| 20.91443 | 7.476312 |  |  |  |  |
| 24.73044 | 3.59543 |  |  |  |  |
| 44.73753 | 4.935338 |  |  |  |  |
| 39.33306 | 5.295628 |  |  |  |  |
| 7.232731 | 7.711237 |  |  |  |  |
| 12.63974 | 4.98 |  |  |  |  |
| 3.689465 | 3.649758 |  |  |  |  |
| 24.73606 | 6.555518 |  |  |  |  |
| 70.9991 | 2.244 |  |  |  |  |
| 6.63203 | 3.416639 |  |  |  |  |
| 21.33088 | 8.07971 |  |  |  |  |
| 10.79433 | 8.976 |  |  |  |  |
| 3.166266 | 5.979146 |  |  |  |  |
| 7.363545 | 16.58486 |  |  |  |  |
| 8.80104 | 5.845817 |  |  |  |  |
| 17.7456 | 4.956917 |  |  |  |  |
| 5.48058 | 3.717534 |  |  |  |  |
| 11.15447 | 9.56 |  |  |  |  |
| 10.33178 | 4.142131 |  |  |  |  |
| 22.51554 | 3.906686 |  |  |  |  |
| 9.593803 | 18.71836 |  |  |  |  |
| 29.01124 | 11.1488 |  |  |  |  |
| 20.91443 | 3.584788 |  |  |  |  |
| 24.73044 | 2.53 |  |  |  |  |
| 44.73753 | 49.20118 |  |  |  |  |
| 39.33306 | 7.718398 |  |  |  |  |
| 7.232731 | 9.114647 |  |  |  |  |
| 12.63974 | 4.483096 |  |  |  |  |
| 3.689465 | 4.018829 |  |  |  |  |
| 24.73606 | 2.3616 |  |  |  |  |
| 70.9991 | 2.520628 |  |  |  |  |
| 6.63203 | 4.14984 |  |  |  |  |
| 21.33088 | 4.327279 |  |  |  |  |
| 10.79433 | 3.082412 |  |  |  |  |
| 3.166266 | 4.172072 |  |  |  |  |
| 7.363545 | 5.43712 |  |  |  |  |
| 8.80104 | 5.544 |  |  |  |  |
| 17.7456 | 20.06499 |  |  |  |  |
| 4.538838 | 4.35035 |  |  |  |  |
| 35.47701 | 10.23846 |  |  |  |  |
| 3.511098 | 4.215248 |  |  |  |  |
| 14.71913 | 4.102655 |  |  |  |  |
| 45.54 | 15.18 |  |  |  |  |
| 8.270689 | 6.421201 |  |  |  |  |
| 5.284 | 4.633053 |  |  |  |  |
| 6.510365 | 5.847377 |  |  |  |  |
| 7.47 | 3.682876 |  |  |  |  |
| 26.55574 | 4.537488 |  |  |  |  |
| 4.858032 | 17.83284 |  |  |  |  |
| 37.8535 | 8.689477 |  |  |  |  |
| 10.16531 | 6.494211 |  |  |  |  |
| 12.924 | 2.5848 |  |  |  |  |
| 2.690765 | 2.140853 |  |  |  |  |
| 5.904 | 5.497025 |  |  |  |  |
| 5.95075 | 4.659292 |  |  |  |  |
| 7.871084 | 3.920514 |  |  |  |  |
| 6.405833 | 6.639903 |  |  |  |  |
| 22.44 | 5.232877 |  |  |  |  |
| 31.10665 | 21.84073 |  |  |  |  |
| 4.470404 | 6.730275 |  |  |  |  |
| 8.976 | 2.244 |  |  |  |  |
| 7.515708 | 8.235779 |  |  |  |  |
| 31.57458 | 8.976 |  |  |  |  |
| 2.842654 | 3.483234 |  |  |  |  |
| 13.92526 | 12.28569 |  |  |  |  |
| 40.65477 | 3.948141 |  |  |  |  |
| 17.19075 | 3.94 |  |  |  |  |
| 31.39905 | 2.792825 |  |  |  |  |
| 12.76781 | 32.45074 |  |  |  |  |
| 4.22209 | 6.563505 |  |  |  |  |
| 10.19136 | 3.193121 |  |  |  |  |
| 5.31691 | 3.760202 |  |  |  |  |
| 11.67599 | 3.119191 |  |  |  |  |
| 26.71162 | 33.88276 |  |  |  |  |
| 11.27041 | 5.106949 |  |  |  |  |
| 29.41058 | 32.68112 |  |  |  |  |
| 12.21636 | 6.650092 |  |  |  |  |
| 6.128218 | 15.204 |  |  |  |  |
| 8.273719 | 4.256269 |  |  |  |  |
| 4.454422 | 4.663272 |  |  |  |  |
| 7.971419 | 29.77771 |  |  |  |  |
| 14.1584 | 10.76194 |  |  |  |  |
| 5.915753 | 13.89732 |  |  |  |  |
| 25.72 | 3.887982 |  |  |  |  |
| 23.2136 | 19.03215 |  |  |  |  |
| 8.903547 | 20.42156 |  |  |  |  |
| 7.463037 | 3.94272 |  |  |  |  |
| 13.68702 | 3.461715 |  |  |  |  |
| 12.16049 | 6.218089 |  |  |  |  |
| 43.75962 | 17.71 |  |  |  |  |
| 5.341736 | 6.119913 |  |  |  |  |
| 14.0166 | 3.829342 |  |  |  |  |
| 6.70051 | 3.558106 |  |  |  |  |
| 15.69208 | 4.389522 |  |  |  |  |
| 4.116901 | 4.622195 |  |  |  |  |
| 13.86468 | 6.474 |  |  |  |  |
| 26.55574 | 4.98 |  |  |  |  |
| 13.32014 | 5.800625 |  |  |  |  |
| 5.635706 | 4.98 |  |  |  |  |
| 5.255398 | 8.459119 |  |  |  |  |
| 5.513445 | 2.515551 |  |  |  |  |
| 2.288074 | 2.65411 |  |  |  |  |
| 13.04613 | 8.856 |  |  |  |  |
| 13.464 | 8.976 |  |  |  |  |
| 4.220756 | 4.235799 |  |  |  |  |
| 4.0573 | 4.862266 |  |  |  |  |
| 22.44 | 11.22 |  |  |  |  |
| 19.67834 | 14.79869 |  |  |  |  |
| 11.10553 | 7.202232 |  |  |  |  |
| 9.553577 | 3.479463 |  |  |  |  |
| 5.990993 | 4.966193 |  |  |  |  |
| 24.62673 | 18.19192 |  |  |  |  |
| 16.78279 | 3.283651 |  |  |  |  |
| 3.262891 | 3.152 |  |  |  |  |
| 8.764089 | 10.87708 |  |  |  |  |
| 10.244 | 10.47076 |  |  |  |  |
| 15.76 | 14.06716 |  |  |  |  |
| 8.249209 | 4.445994 |  |  |  |  |
| 3.988128 | 7.1808 |  |  |  |  |
| 3.541086 | 2.370521 |  |  |  |  |
| 11.67233 | 9.072 |  |  |  |  |
| 12.4877 | 23.17575 |  |  |  |  |
| 4.99896 | 5.115104 |  |  |  |  |
| 5.294267 | 25.2 |  |  |  |  |
| 11.2796 | 4.924594 |  |  |  |  |
| 19.17723 | 6.662699 |  |  |  |  |
| 4.895277 | 5.870389 |  |  |  |  |
| 6.262646 | 6.745923 |  |  |  |  |
| 3.255149 | 4.663272 |  |  |  |  |
| 48.52668 | 19.53186 |  |  |  |  |
| 5.144 | 5.579441 |  |  |  |  |
| 15.432 | 10.288 |  |  |  |  |

**Statistical report
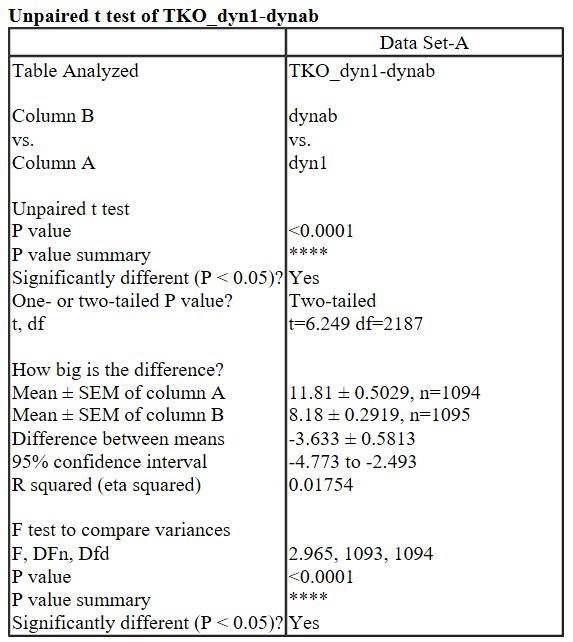

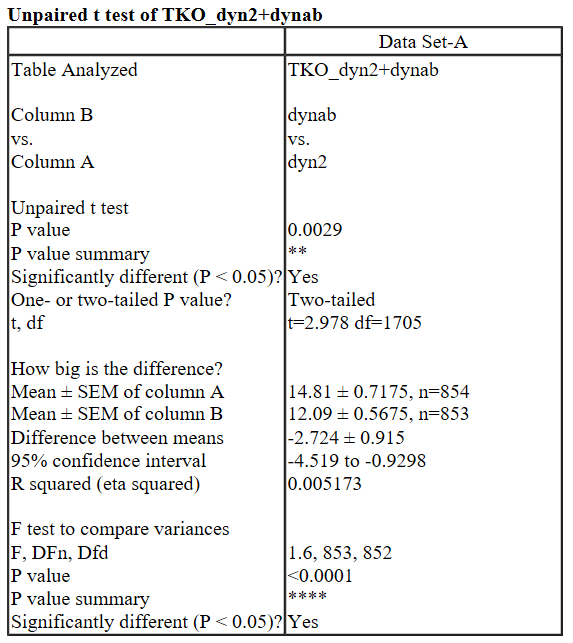
:**


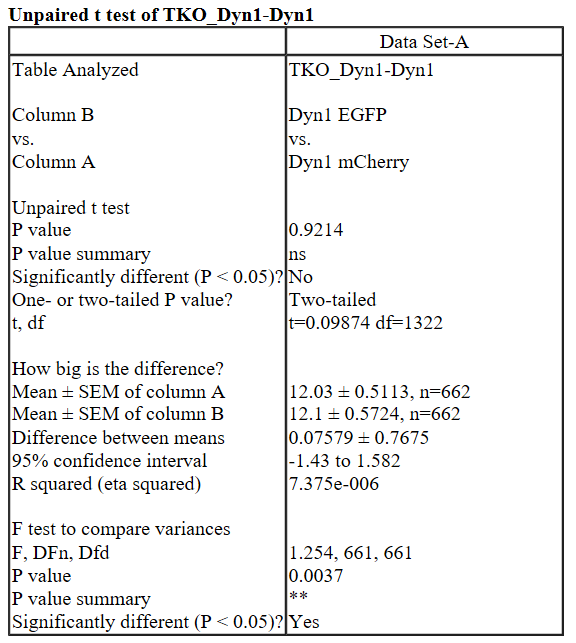

Supplement: Figure 4—source data 2. [file elife-25197-fig4-data2.docx]
