# Supplementary material for: Uncoupling of dynamin polymerization and GTPase activity revealed by the conformation-specific nanobody dynab
Source: eLife. 2017 Oct 12;6:e25197. doi: 10.7554/eLife.25197 (PMC5658065; doi:10.7554/eLife.25197)
Supplement: Figure 4—source data 3. [file elife-25197-fig4-data3.docx]

**Figure 4-Source Data 3 (panel B)**

Comparison of duration of dynamin 1-2 events with dynab events in Hela cells (expressed in seconds), and statistical report

| dyn1 | dynab | dyn2 | dynab | dyn1_(mCherry)_ | dyn1_(EGFP_) |
| --- | --- | --- | --- | --- | --- |
| 5.153392 | 3.2507 | 8.375882 | 17.172 | 2.194404 | 2.126823 |
| 21.02213 | 16.3906 | 2.9534 | 6.056063 | 6.450992 | 2.938741 |
| 3.46464 | 2.6231 | 3.499187 | 2.791681 | 5.096952 | 4.534386 |
| 2.669299 | 3.3539 | 3.11143 | 4.168888 | 22.78138 | 14.17593 |
| 1.966739 | 5.2705 | 43.93438 | 38.06 | 4.582179 | 4.458642 |
| 5.576277 | 11.3969 | 10.19902 | 32.35597 | 10.87572 | 14.00176 |
| 2.336081 | 3.3472 | 4.801547 | 2.407943 | 3.739994 | 3.636468 |
| 3.729542 | 4.5752 | 1.742986 | 5.820634 | 2.654145 | 4.660133 |
| 2.668846 | 2.9859 | 4.314331 | 3.741458 | 2.574675 | 2.233031 |
| 7.346917 | 7.5937 | 12.35603 | 2.574109 | 3.561563 | 3.778339 |
| 3.4194 | 8.799 | 3.207507 | 6.082115 | 2.36961 | 8.133725 |
| 7.462575 | 2.659 | 6.774017 | 4.487502 | 17.56144 | 17.94218 |
| 6.249831 | 2.9731 | 11.03573 | 7.589362 | 8.989394 | 34.12344 |
| 7.93673 | 1.9537 | 1.451875 | 3.07291 | 12.59379 | 5.363855 |
| 4.941136 | 3.5145 | 3.276381 | 4.145289 | 4.189827 | 5.463455 |
| 32.2121 | 25.806 | 1.694769 | 24.60526 | 3.87337 | 3.669402 |
| 3.574212 | 3.578 | 18.34306 | 7.155515 | 9.895746 | 15.82321 |
| 3.123602 | 2.9804 | 3.15457 | 2.688184 | 5.161471 | 5.840162 |
| 2.319765 | 3.2753 | 2.048235 | 2.82138 | 33.14486 | 3.5688 |
| 2.916059 | 2.8921 | 3.957374 | 4.620021 | 34.36273 | 39.80625 |
| 0.06739786 | 2.8679 | 5.298111 | 5.661797 | 16.13028 | 8.862431 |
| 3.073704 | 2.7199 | 4.694141 | 2.746423 | 2.339986 | 9.050142 |
| 4.543215 | 3.0088 | 2.172199 | 2.091533 | 2.325866 | 3.130342 |
| 2.877222 | 3.5842 | 12.03807 | 2.153907 | 2.321127 | 2.66278 |
| 2.47969 | 2.5172 | 4.469718 | 4.706992 | 4.262254 | 9.666424 |
| 4.009928 | 2.6835 | 8.353334 | 3.08484 | 5.491342 | 3.233378 |
| 10.56666 | 7.8785 | 2.486372 | 2.45944 | 12.61808 | 8.138399 |
| 2.136765 | 3.1096 | 2.860197 | 2.663493 | 50.1097 | 48.05218 |
| 3.383688 | 3.6139 | 2.169018 | 4.515638 | 4.410581 | 6.401544 |
| 11.32386 | 2.2185 | 4.000925 | 1.910844 | 6.747657 | 27.22223 |
| 2.40297 | 1.8156 | 2.708007 | 4.456081 | 6.570705 | 5.841282 |
| 4.799929 | 5.1693 | 3.80697 | 2.898458 | 5.143683 | 6.760744 |
| 5.047917 | 5.9351 | 3.540528 | 5.247594 | 8.652358 | 4.432757 |
| 6.635571 | 4.9618 | 10.53068 | 9.395838 | 2.154185 | 2.741796 |
| 11.35229 | 3.9187 | 2.919251 | 2.686292 | 13.64044 | 5.706443 |
| 2.631402 | 2.0974 | 5.872174 | 3.858577 | 4.057637 | 4.169017 |
| 2.133331 | 2.5837 | 2.758659 | 1.891849 | 4.284874 | 4.070286 |
| 4.18218 | 11.6606 | 3.401448 | 1.582334 | 7.9977 | 8.480724 |
| 2.883841 | 3.9939 | 4.532506 | 12.94402 | 7.339446 | 3.528292 |
| 2.750608 | 3.819 | 23.70976 | 12.48788 | 12.43783 | 2.358541 |
| 2.272533 | 3.616 | 16.39404 | 9.502556 | 6.680711 | 3.292895 |
| 3.518247 | 3.6452 | 10.98854 | 14.19981 | 4.910703 | 3.750997 |
| 8.421625 | 4.692 | 13.86294 | 2.508829 | 3.154736 | 2.630424 |
| 3.275065 | 3.4659 | 3.486256 | 3.860436 | 10.90591 | 2.902975 |
| 4.026922 | 3.8386 | 23.93331 | 5.323095 | 17.67958 | 3.176283 |
| 5.271332 | 3.0604 | 2.569069 | 14.02025 | 5.890729 | 5.625033 |
| 4.378217 | 3.8338 | 33.01205 | 5.065692 | 9.475368 | 27.51334 |
| 4.152141 | 4.3056 | 3.150086 | 2.955157 | 11.00731 | 6.088031 |
| 4.405723 | 3.8339 | 7.102449 | 7.366568 | 3.34893 | 5.290556 |
| 3.111307 | 3.7243 | 29.792 | 3.663945 | 5.759883 | 6.033851 |
| 3.275686 | 2.6467 | 4.203528 | 6.504393 | 6.673083 | 16.32412 |
| 3.620247 | 2.3607 | 15.46358 | 2.228931 | 45.66777 | 9.158909 |
| 2.813759 | 3.297 | 21.65734 | 2.162881 | 20.49096 | 12.82633 |
| 7.141806 | 3.832 | 3.161187 | 2.94082 | 3.829791 | 6.258176 |
| 3.561398 | 2.4715 | 22.43829 | 4.72784 | 24.21753 | 33.35047 |
| 3.40331 | 2.6294 | 3.679536 | 1.288295 | 5.738598 | 5.524536 |
| 5.523773 | 4.0404 | 6.37557 | 4.404789 | 3.158997 | 2.780999 |
| 11.35427 | 2.6146 | 4.262374 | 3.816584 | 20.33064 | 15.18325 |
| 2.972734 | 4.634 | 7.993093 | 2.804781 | 6.513619 | 14.96752 |
| 3.665252 | 10.3219 | 2.451763 | 6.5026 | 4.481178 | 6.634652 |
| 2.593972 | 3.1346 | 12.22758 | 6.076929 | 9.054815 | 8.375318 |
| 2.481022 | 2.5856 | 2.753607 | 4.08722 | 3.652671 | 11.28199 |
| 9.977154 | 11.9288 | 14.1561 | 2.587652 | 19.02172 | 15.26633 |
| 2.394521 | 3.2728 | 17.20047 | 2.038432 | 5.196219 | 13.2826 |
| 4.565697 | 5.3492 | 4.134029 | 2.079777 | 3.198212 | 3.90384 |
| 5.524896 | 3.0182 | 2.392048 | 4.754454 | 16.28539 | 4.011493 |
| 2.951166 | 16.8731 | 2.282273 | 2.88419 | 4.274764 | 3.049948 |
| 2.143594 | 1.8914 | 5.473784 | 2.699658 | 7.348234 | 4.460167 |
| 2.906468 | 4.8736 | 3.252548 | 2.070895 | 3.916127 | 3.90448 |
| 3.716472 | 3.0444 | 4.545791 | 5.157223 | 3.785207 | 2.595777 |
| 1.549762 | 2.3924 | 5.516869 | 16.78951 | 1.923765 | 3.034069 |
| 2.86479 | 3.7831 | 2.519397 | 0.003206325 | 58.2705 | 35.21489 |
| 3.458144 | 2.9232 | 10.21003 | 2.86796 | 5.351681 | 6.727251 |
| 4.479673 | 16.8457 | 14.19016 | 2.301713 | 11.70023 | 13.36361 |
| 2.888395 | 4.325 | 2.229649 | 2.984785 | 2.470781 | 4.607374 |
| 3.597457 | 3.4621 | 4.212824 | 2.888667 | 4.686849 | 4.581847 |
| 3.003048 | 2.7154 | 2.476377 | 3.825573 | 3.520281 | 5.832267 |
| 2.743035 | 2.2304 | 2.136305 | 2.956583 | 6.126534 | 12.81343 |
| 2.140017 | 2.5507 | 10.58352 | 4.861919 | 23.35421 | 21.98768 |
| 7.517755 | 2.8549 | 5.67535 | 3.160354 | 4.719943 | 6.59749 |
| 4.135083 | 5.119 | 5.668877 | 3.281569 | 5.390662 | 5.587132 |
| 3.338814 | 4.7833 | 4.889891 | 4.498534 | 11.1097 | 12.08443 |
| 3.629208 | 3.8355 | 3.659689 | 4.390335 | 4.806454 | 4.169954 |
| 3.021896 | 2.5343 | 4.455425 | 5.008399 | 10.47151 | 9.533881 |
| 3.831994 | 6.8678 | 14.88402 | 21.32539 | 7.188862 | 3.300696 |
| 2.499503 | 3.2559 | 82.02081 | 3.822094 | 26.87651 | 2.858016 |
| 5.624052 | 3.4949 | 11.92007 | 18.13502 | 3.52575 | 4.39649 |
| 2.241478 | 1.8569 | 15.21543 | 35.69757 | 13.89488 | 13.54844 |
| 3.009913 | 3.032 | 4.882149 | 4.938747 | 35.88612 | 20.87824 |
| 5.99939 | 2.5104 | 2.471474 | 4.128389 | 83.73074 | 10.32375 |
| 5.714248 | 3.9766 | 4.746627 | 4.021718 | 5.752132 | 4.155843 |
| 3.530219 | 2.9874 | 3.962164 | 2.502754 | 7.088556 | 2.900821 |
| 19.84376 | 1.6932 | 3.164702 | 2.560214 | 10.79992 | 3.817126 |
| 4.839716 | 2.7983 | 2.778726 | 5.342325 | 7.850344 | 5.835366 |
| 4.765405 | 4.3261 | 3.425226 | 11.27031 | 7.686922 | 8.013082 |
| 3.736364 | 4.2715 | 5.132301 | 6.053129 | 5.126674 | 13.23722 |
| 3.432893 | 9.0031 | 3.903958 | 3.814303 | 3.173075 | 5.658182 |
| 5.759855 | 3.1552 | 4.000445 | 9.616897 | 6.422258 | 5.006459 |
| 4.74423 | 2.5058 | 13.32255 | 3.354005 | 14.09487 | 9.232283 |
| 5.206048 | 4.3936 | 25.96698 | 15.49737 | 5.346365 | 3.840963 |
| 19.39507 | 3.4636 | 5.612551 | 5.344138 | 4.717344 | 2.53512 |
| 4.715054 | 2.1377 | 4.139998 | 7.537406 | 6.832364 | 6.320388 |
| 2.143698 | 1.695 | 3.549013 | 2.752215 | 12.87311 | 4.666117 |
| 3.789955 | 7.6869 | 3.330629 | 4.565411 | 5.765936 | 8.358672 |
| 2.810685 | 4.9943 | 5.528918 | 6.373403 | 4.668294 | 4.426555 |
| 3.447537 | 5.1208 | 7.93918 | 21.87653 | 3.384434 | 3.433021 |
| 4.093304 | 6.4953 | 3.625626 | 3.194066 | 22.29394 | 26.6089 |
| 12.70554 | 17.5654 | 14.62553 | 4.692009 | 18.85841 | 17.87317 |
| 6.416002 | 8.8818 | 9.777839 | 4.806442 | 5.022133 | 7.884023 |
| 4.369156 | 1.4286 | 4.047268 | 10.50008 | 30.29066 | 12.06298 |
| 186.6253 | 14.1037 | 10.12175 | 4.039572 | 3.830043 | 4.92089 |
| 8.664303 | 7.1719 | 4.512361 | 3.835192 | 5.63057 | 5.557546 |
| 4.307275 | 2.8599 | 3.476244 | 3.017698 | 3.613633 | 3.839526 |
| 2.511629 | 4.2103 | 6.006478 | 2.58583 | 13.20409 | 7.682094 |
| 3.270992 | 3.7885 | 42.79696 | 2.643951 | 4.44271 | 2.250829 |
| 26.75703 | 2.4942 | 7.229988 | 3.31879 | 4.360414 | 6.106578 |
| 2.408058 | 3.7755 | 8.633982 | 5.74668 | 9.578508 | 22.63917 |
| 4.086239 | 5.1203 | 11.65017 | 42.50786 | 3.333101 | 5.874083 |
| 6.761764 | 5.4846 | 7.566673 | 6.323227 | 6.357573 | 3.241025 |
| 6.724588 | 2.7809 | 8.328494 | 7.506189 | 3.345069 | 13.02424 |
| 3.450027 | 2.5097 | 15.30795 | 55.5977 | 6.365385 | 6.854127 |
| 7.148999 | 13.2546 | 4.333529 | 27.53536 | 2.73469 | 5.991093 |
| 6.329277 | 5.9972 | 14.11713 | 6.104754 | 4.708682 | 4.804633 |
| 4.21329 | 4.2174 | 8.143585 | 9.085353 | 4.748077 | 3.134627 |
| 6.814925 | 3.3037 | 3.320771 | 6.464961 | 7.59075 | 4.603972 |
| 3.516283 | 3.5891 | 6.557391 | 3.503045 | 4.988672 | 4.528472 |
| 3.157829 | 9.0625 | 6.785255 | 6.499377 | 3.514866 | 15.95805 |
| 2.59015 | 1.4367 | 4.383392 | 19.52016 | 9.158821 | 4.951257 |
| 4.55403 | 1.6506 | 15.46954 | 3.658871 | 15.72194 | 8.063757 |
| 11.45077 | 9.1936 | 11.99878 | 4.145642 | 5.075081 | 5.120076 |
| 2.900394 | 2.3672 | 15.73957 | 3.350758 | 9.436723 | 15.31832 |
| 5.512717 | 4.1732 | 3.126488 | 6.305733 | 3.214841 | 4.900944 |
| 2.437899 | 3.1309 | 15.50544 | 14.9917 | 5.030793 | 4.347386 |
| 5.19757 | 7.6285 | 23.01756 | 4.572967 | 3.47247 | 11.64164 |
| 2.282331 | 4.0862 | 4.347872 | 4.764379 | 16.81724 | 9.645289 |
| 8.623433 | 4.9838 | 32.6683 | 4.294433 | 36.71043 | 11.16095 |
| 2.408806 | 4.6467 | 3.93613 | 3.361438 | 7.845779 | 5.584797 |
| 5.271201 | 4.9796 | 13.87799 | 4.528103 | 3.858465 | 3.950247 |
| 3.258546 | 2.9978 | 5.432714 | 16.55739 | 13.63097 | 9.733123 |
| 4.236821 | 1.7982 | 3.54397 | 1.901269 | 9.413512 | 3.235259 |
| 2.585475 | 2.533 | 3.780114 | 3.064206 | 5.914004 | 4.220065 |
| 9.628614 | 4.4071 | 2.628377 | 4.643636 | 12.23189 | 5.567842 |
| 3.17551 | 3.803 | 4.599042 | 4.095201 | 29.60808 | 20.87569 |
| 6.424105 | 6.7527 | 12.69817 | 4.065469 | 27.93355 | 18.74543 |
| 8.316773 | 4.609 | 4.497609 | 6.265898 | 6.016808 | 11.23529 |
| 4.998872 | 4.4626 | 3.852705 | 0.09080004 | 12.41221 | 5.572715 |
| 3.05007 | 3.0563 | 2.760276 | 5.291389 | 5.883519 | 12.95984 |
| 26.55367 | 3.9389 | 3.539871 | 3.723679 | 31.71058 | 8.388764 |
| 4.050091 | 3.1031 | 5.09119 | 4.459932 | 16.08946 | 14.78553 |
| 3.791594 | 4.9365 | 18.6807 | 4.198834 | 4.611754 | 10.2622 |
| 4.405251 | 4.7383 | 7.489876 | 27.49116 | 3.407037 | 3.883159 |
| 11.53689 | 2.4969 | 14.28244 | 19.04031 | 5.329889 | 4.205042 |
| 5.51839 | 4.0187 | 9.252999 | 3.820621 | 4.874268 | 10.28508 |
| 8.747226 | 4.2204 | 4.155923 | 3.870361 | 3.021406 | 5.462257 |
| 5.55821 | 7.1682 | 7.407035 | 4.723246 | 11.39661 | 8.013126 |
| 5.65337 | 8.1563 | 68.36897 | 19.66227 | 3.902359 | 6.587281 |
| 4.242633 | 4.4125 | 4.576773 | 4.252768 | 10.82475 | 4.460379 |
| 7.590431 | 6.326 | 4.743496 | 4.874856 | 4.485281 | 8.483536 |
| 5.157208 | 4.3667 | 3.43235 | 12.85376 | 9.880201 | 8.39217 |
| 2.227666 | 5.895 | 8.92502 | 4.577822 | 7.657469 | 8.32336 |
| 5.360243 | 26.034 | 22.01432 | 24.09051 | 3.691924 | 6.436691 |
| 4.905808 | 2.3729 | 5.899964 | 4.703793 | 3.809488 | 4.14642 |
| 6.371337 | 15.6569 | 18.10568 | 4.680122 | 2.231888 | 6.659196 |
| 2.359765 | 3.1721 | 10.31615 | 19.06166 | 36.45481 | 31.07437 |
| 5.804431 | 1.4506 | 3.119625 | 8.384455 | 5.774116 | 11.94903 |
| 9.267389 | 6.6959 | 3.011083 | 4.51355 | 6.981641 | 4.376108 |
| 5.966358 | 3.9924 | 25.91432 | 54.49693 | 6.58957 | 7.085127 |
| 4.701252 | 4.3151 | 5.57855 | 8.183635 | 5.685001 | 3.802875 |
| 6.039544 | 3.2074 | 14.3919 | 4.042146 | 4.674493 | 3.655115 |
| 6.254511 | 9.0631 | 3.326533 | 6.874153 | 3.467374 | 3.448537 |
| 3.371107 | 5.349 | 3.101223 | 1.705714 | 3.894562 | 6.586611 |
| 3.299872 | 2.6775 | 7.276956 | 8.224205 | 5.794768 | 6.640167 |
| 3.245867 | 3.9746 | 4.026817 | 6.001052 | 7.801761 | 29.64406 |
| 3.844421 | 3.3476 | 4.991699 | 3.76979 | 3.765101 | 14.02124 |
| 4.606849 | 4.3301 | 5.607867 | 6.531778 | 8.091245 | 5.000095 |
| 2.480192 | 3.647 | 4.917695 | 5.117062 | 2.661861 | 2.465088 |
| 3.025101 | 3.7589 | 4.840033 | 5.685566 | 25.45206 | 16.80764 |
| 5.820499 | 1.7595 | 4.503224 | 47.17096 | 15.24679 | 22.42371 |
| 2.692196 | 2.715 | 10.94715 | 7.255761 | 3.168757 | 2.448907 |
| 3.810178 | 13.4131 | 2.293502 | 2.317706 | 13.03831 | 31.0104 |
| 4.290545 | 1.9121 | 7.343382 | 3.983326 | 3.125726 | 12.96963 |
| 3.093176 | 3.419 | 3.752802 | 4.433854 | 17.17183 | 13.82097 |
| 2.692436 | 3.787 | 7.919574 | 3.612901 | 10.61538 | 4.73525 |
| 3.724098 | 5.0016 | 5.769078 | 15.13368 | 3.641974 | 6.24532 |
| 5.539906 | 4.4895 | 19.37253 | 3.864938 | 8.136837 | 17.83729 |
| 7.715841 | 4.3782 | 4.108689 | 4.270686 | 10.76594 | 6.722395 |
| 4.667504 | 2.4541 | 5.816577 | 5.304395 | 4.032613 | 3.839426 |
| 3.383423 | 3.3099 | 7.717924 | 3.963045 | 4.66359 | 4.106987 |
| 4.050063 | 4.9453 | 9.291615 | 3.34932 | 9.949429 | 2.503756 |
| 3.367145 | 5.5832 | 3.901431 | 28.67824 | 9.111622 | 21.70871 |
| 3.712569 | 4.6238 | 3.946452 | 4.010656 | 74.58733 | 7.644056 |
| 3.71932 | 3.636 | 9.677586 | 8.200527 | 5.830577 | 6.688882 |
| 5.992081 | 3.4552 | 5.29237 | 8.21024 | 4.275766 | 8.233774 |
| 4.177862 | 2.1947 | 2.936264 | 3.419157 | 7.346385 | 3.67883 |
| 2.203545 | 2.7858 | 5.165866 | 6.582075 | 21.25972 | 7.222318 |
| 2.459509 | 3.1036 | 7.715729 | 5.250042 | 4.350658 | 3.56934 |
| 3.358088 | 2.1706 | 0.1387732 | 15.19305 | 10.10343 | 30.3522 |
| 1.536456 | 3.1119 | 2.712561 | 7.161597 | 5.166625 | 2.81568 |
| 2.765333 | 2.0715 | 20.84559 | 19.78668 | 9.344379 | 4.545237 |
| 3.260368 | 3.1978 | 3.250531 | 12.73195 | 3.262193 | 24.19977 |
| 4.728901 | 3.8463 | 17.49891 | 19.87166 | 16.59111 | 20.73629 |
| 6.397839 | 2.9968 | 4.530854 | 4.303087 | 11.84527 | 16.51995 |
| 1.999051 | 2.8843 | 8.475431 | 5.767759 | 33.10352 | 52.25155 |
| 2.769737 | 2.8512 | 7.137334 | 1.212075 | 9.637152 | 23.11361 |
| 2.591619 | 2.6586 | 4.593011 | 39.80854 | 2.900172 | 3.936298 |
| 5.342558 | 2.6548 | 3.750421 | 23.63222 | 6.602915 | 26.60704 |
| 5.004059 | 3.0952 | 13.59964 | 4.902205 | 9.27013 | 13.30193 |
| 4.373154 | 4.7392 | 3.856683 | 2.368857 | 1.421757 | 5.212755 |
| 4.719927 | 1.9032 | 19.83306 | 2.641262 | 8.232222 | 6.491824 |
| 7.330477 | 10.0204 | 1.933115 | 4.158681 | 8.582707 | 4.249012 |
| 5.433887 | 4.4482 | 3.084258 | 3.042324 | 3.505277 | 4.518551 |
| 4.873335 | 1.783 | 7.003986 | 3.04279 | 4.430575 | 5.366783 |
| 3.69417 | 4.4463 | 2.480832 | 2.311031 | 5.95049 | 10.53116 |
| 2.059278 | 5.5643 | 2.95276 | 4.060144 | 8.772995 | 10.62727 |
| 3.934188 | 4.0489 | 7.623952 | 4.264447 | 6.914191 | 10.55078 |
| 4.446733 | 28.8273 | 4.192572 | 3.278828 | 7.221707 | 4.490625 |
| 6.680808 | 0.0298 | 2.559238 | 3.030665 | 4.145797 | 3.849883 |
| 5.627133 | 1.8195 | 5.720595 | 15.27689 | 4.723519 | 2.955287 |
| 3.415457 | 2.7845 | 4.167871 | 8.014299 | 4.752302 | 6.99032 |
| 3.663166 | 4.521 | 4.221669 | 12.11718 | 7.101891 | 6.343116 |
| 5.545373 | 3.2671 | 22.62977 | 5.17255 | 8.258392 | 29.15786 |
| 1.531749 | 2.0921 | 2.846246 | 3.677704 | 11.29332 | 8.215576 |
| 2.32317 | 3.5065 | 13.3436 | 4.87153 | 14.09889 | 5.033337 |
| 3.022662 | 3.6964 | 4.970001 | 7.897417 | 5.074673 | 4.366702 |
| 5.956467 | 3.7451 | 6.442256 | 4.418777 | 4.304395 | 4.634373 |
| 3.696216 | 9.6071 | 3.36192 | 17.34375 | 3.668596 | 10.06831 |
| 4.827001 | 5.8356 | 24.75628 | 3.341005 | 4.308399 | 6.5567 |
| 3.742849 | 3.8194 | 17.67551 | 26.80026 | 8.485793 | 15.20399 |
| 5.056397 | 3.7628 | 4.036809 | 4.668465 | 10.91716 | 12.13789 |
| 3.560583 | 3.3921 | 6.567292 | 8.139456 | 29.92154 | 28.00814 |
| 18.29983 | 2.4712 | 2.58234 | 3.469808 | 16.43591 | 6.364595 |
| 4.507099 | 10.3569 | 4.190949 | 6.960547 | 4.067092 | 4.264896 |
| 4.150426 | 5.9762 | 3.466703 | 3.419588 | 4.345058 | 5.320508 |
| 5.884307 | 5.4905 | 20.89578 | 3.361737 | 4.177668 | 6.144075 |
| 4.089399 | 3.7374 | 4.382726 | 4.212087 | 6.096133 | 2.386133 |
| 3.464284 | 2.6324 | 5.089384 | 9.745234 | 10.87362 | 4.074453 |
| 5.001092 | 2.1147 | 3.315695 | 7.764764 | 6.315451 | 5.835875 |
| 3.432851 | 4.7887 | 4.003428 | 3.646064 | 3.602842 | 5.114177 |
| 3.489057 | 4.1679 | 4.360392 | 3.326624 | 10.73009 | 5.38105 |
| 11.79509 | 3.5249 | 14.32361 | 37.2701 | 11.662 | 7.115429 |
| 4.704708 | 2.2218 | 13.00394 | 4.191746 | 11.64058 | 4.421508 |
| 10.87948 | 2.246 | 5.807992 | 5.922046 | 5.53035 | 3.241999 |
| 3.710453 | 3.9758 | 25.71277 | 112.5328 | 4.965197 | 3.785318 |
| 3.089425 | 4.9426 | 4.379148 | 2.95409 | 17.27235 | 20.58285 |
| 3.610353 | 2.1988 | 3.003331 | 2.787262 | 14.79427 | 19.50496 |
| 7.669233 | 2.7876 | 13.94849 | 17.57019 | 20.09166 | 7.482446 |
| 2.146665 | 2.1241 | 5.445119 | 17.53898 | 4.929734 | 5.396514 |
| 3.495884 | 2.9939 | 2.734426 | 2.597585 | 3.645953 | 10.89761 |
| 2.945311 | 1.5504 | 3.530206 | 3.627171 | 4.396085 | 4.226799 |
| 4.776181 | 5.6642 | 8.583469 | 19.71781 | 3.292179 | 3.655786 |
| 3.164632 | 2.6745 | 4.694687 | 2.495993 | 15.61225 | 32.52185 |
| 3.192417 | 3.0176 | 3.280933 | 3.860092 | 24.14052 | 7.501953 |
| 1.816985 | 2.9012 | 2.04876 | 2.776068 | 6.101756 | 4.381368 |
| 4.544088 | 4.1944 | 2.491227 | 2.607549 | 3.388388 | 4.539322 |
| 4.675646 | 5.2583 | 12.41006 | 2.116329 | 8.367985 | 10.08629 |
| 7.360615 | 2.4476 | 1.751518 | 6.332537 | 48.26688 | 42.59321 |
| 2.352886 | 4.1631 | 5.227752 | 8.040738 | 12.0239 | 11.48976 |
| 2.949015 | 3.813 | 3.692912 | 1.766524 | 0.1000479 | 6.957306 |
| 5.48388 | 4.6271 | 3.79247 | 5.143998 | 12.9645 | 13.65312 |
| 8.157118 | 12.0144 | 9.100814 | 2.785687 | 12.60935 | 10.8152 |
| 3.824744 | 3.9257 | 10.12015 | 7.974314 | 9.25084 | 10.86205 |
| 5.785802 | 4.0801 | 21.57749 | 13.29809 | 34.41075 | 3.260122 |
| 3.856106 | 5.6897 | 4.564982 | 11.09436 | 7.733219 | 10.4805 |
| 4.541925 | 6.284 | 11.952 | 6.927771 | 3.850642 | 28.56308 |
| 2.937423 | 2.1395 | 56.1397 | 20.5143 | 10.30042 | 21.94876 |
| 37.19357 | 2.7545 | 3.638467 | 7.082851 | 15.68503 | 19.22855 |
| 5.978399 | 2.885 | 9.355865 | 3.368064 | 5.834963 | 3.573538 |
| 3.045578 | 2.6051 | 3.354674 | 1.605845 | 24.29811 | 2.27526 |
| 3.999073 | 4.9125 | 1.884949 | 13.92409 | 14.22045 | 11.02094 |
| 4.463556 | 3.5207 | 5.426148 | 7.307867 | 1.400089 | 29.27227 |
| 2.351654 | 2.0936 | 2.267816 | 5.231213 | 21.42853 | 6.466946 |
| 2.278873 | 2.3721 | 13.90564 | 4.128361 | 3.639462 | 4.4876 |
| 6.594135 | 2.6276 | 24.02731 | 4.070545 | 11.54538 | 2.289405 |
| 3.386564 | 2.1996 | 7.246894 | 9.686795 | 18.83279 | 12.98249 |
| 2.468136 | 4.7876 | 1.680668 | 4.086534 | 27.3601 | 20.712 |
| 3.98004 | 2.7726 | 6.892484 | 4.774606 | 2.886921 | 19.39147 |
| 3.698912 | 2.6653 | 9.920389 | 7.516503 | 27.4055 | 60.69506 |
| 3.792272 | 4.4975 | 4.75388 | 2.364228 | 6.075356 | 3.262322 |
| 2.723328 | 2.9445 | 3.581583 | 2.876323 | 3.19115 | 2.698302 |
| 4.181459 | 3.4742 | 2.538024 | 6.770258 | 12.24409 | 12.082 |
| 7.315055 | 9.1182 | 4.588692 | 2.801895 | 18.12283 | 19.71334 |
| 4.442387 | 3.2667 | 10.59531 | 5.86172 | 11.69322 | 14.30255 |
| 4.375016 | 2.7689 | 3.832807 | 2.032688 | 6.253852 | 7.161478 |
| 2.754582 | 6.0696 | 1.690452 | 3.092461 | 7.827749 | 5.931112 |
| 3.39264 | 2.0157 | 11.50213 | 9.07427 | 2.193625 | 2.092877 |
| 2.759486 | 4.099 | 5.27662 | 2.628117 | 8.399831 | 4.179115 |
| 3.153381 | 2.3263 | 3.647406 | 2.810709 | 5.283678 | 2.507418 |
| 2.470985 | 2.9479 | 2.714138 | 2.848015 | 2.458129 | 2.603578 |
| 3.29425 | 1.5948 | 2.312293 | 2.842434 | 18.48148 | 13.49997 |
| 4.696551 | 4.1094 | 3.561723 | 4.234253 | 8.814628 | 4.390662 |
| 9.678536 | 22.5126 | 2.882371 | 2.759045 | 2.998637 | 3.187108 |
| 5.479321 | 2.9148 | 2.931744 | 2.894589 | 2.961807 | 5.261087 |
| 7.474984 | 2.7666 | 34.6 | 34.6 | 6.498827 | 4.463363 |
| 5.462753 | 3.1765 | 26.815 | 26.81879 | 6.790539 | 6.660637 |
| 4.624375 | 4.8406 | 34.2886 | 34.31143 | 3.702462 | 10.38993 |
| 6.632537 | 3.6398 | 5.263022 | 10.83673 | 10.11985 | 36.71875 |
| 3.337058 | 3.0209 | 12.268 | 16.26604 | 20.66738 | 19.18804 |
| 7.375797 | 3.594 | 122.6037 | 121.1 | 6.398901 | 9.509241 |
| 2.640757 | 1.838 | 41.52 | 42.9358 | 7.094086 | 5.298757 |
| 2.130654 | 3.4526 | 10.38 | 10.38 | 7.6608 | 7.955147 |
| 3.024171 | 4.3297 | 4.201004 | 5.234029 | 12.83461 | 7.267525 |
| 3.075987 | 3.8902 | 2.305608 | 8.743627 | 37.31021 | 38.304 |
| 2.988907 | 3.3735 | 3.012568 | 3.924214 | 4.024367 | 4.407303 |
| 4.505031 | 4.1065 | 3.158052 | 2.439226 | 48.57594 | 14.1916 |
| 4.543441 | 3.1237 | 14.3878 | 13.98478 | 6.602985 | 3.457459 |
| 8.176086 | 3.3225 | 6.530656 | 29.30209 | 3.417864 | 6.091705 |
| 2.852143 | 3.9719 | 15.76201 | 13.824 | 15.00817 | 31.6689 |
| 8.464497 | 9.4774 | 49.85256 | 40.199 | 3.218629 | 2.958489 |
| 5.531025 | 2.6556 | 8.276536 | 4.780379 | 29.95112 | 39.38865 |
| 4.232918 | 3.6686 | 42.20562 | 28.3353 | 31.56038 | 28.728 |
| 3.916415 | 3.82 | 2.45376 | 2.483564 | 3.698178 | 11.3169 |
| 3.367305 | 3.7036 | 33.44004 | 88.37949 | 21.37059 | 18.6682 |
| 4.903393 | 6.9708 | 6.573628 | 14.20017 | 10.34465 | 24.81157 |
| 5.001179 | 2.8234 | 24.58772 | 1.397219 | 4.489932 | 13.35154 |
| 8.251942 | 6.795 | 6.2208 | 6.438021 | 15.37161 | 10.22367 |
| 5.192432 | 24.8048 | 4.58507 | 5.046157 | 16.2792 | 17.13896 |
| 6.144846 | 6.0063 | 3.489924 | 2.949677 | 7.979509 | 7.608307 |
| 4.386652 | 2.854 | 10.7111 | 6.048226 | 4.46608 | 6.51901 |
| 2.875868 | 2.9693 | 6.716168 | 2.623347 | 14.364 | 17.31479 |
| 3.068693 | 1.8817 | 4.654028 | 3.78179 | 9.453576 | 9.741921 |
| 5.393245 | 2.8093 | 20.83693 | 7.631215 | 6.277905 | 3.662648 |
| 14.42248 | 2.1405 | 2.648051 | 54.38999 | 7.376207 | 6.284968 |
| 4.734394 | 4.1664 | 4.082441 | 5.381107 | 8.625428 | 8.6184 |
| 6.254828 | 7.6904 | 6.437026 | 6.4356 | 12.72871 | 3.491464 |
| 4.269104 | 4.088 | 44.91036 | 29.33417 | 8.1396 | 5.61042 |
| 12.08486 | 19.3264 | 11.8678 | 11.88831 | 4.63674 | 3.456999 |
| 3.161127 | 3.3189 | 4.7056 | 4.735936 | 3.598142 | 4.880698 |
| 15.29136 | 21.9665 | 17.95191 | 8.203052 | 9.785198 | 10.86307 |
| 7.737373 | 3.9156 | 21.24087 | 22.64 | 8.68057 | 11.95524 |
| 12.78456 | 3.6717 | 3.806607 | 4.688275 | 18.44372 | 35.53802 |
| 3.125841 | 3.1258 | 3.834531 | 5.080833 | 3.125667 | 11.11117 |
| 5.041691 | 2.6806 | 5.423293 | 13.33723 | 104.8 | 104.8 |
| 20.12117 | 5.7548 | 45.30629 | 46.75941 | 19.54328 | 21.58072 |
| 6.912 | 11.9349 | 24.77516 | 22.64315 | 23.58 | 24.101 |
| 2.456142 | 2.0041 | 47.48323 | 45.28 | 16.78745 | 26.04315 |
| 4.955375 | 3.7281 | 47.56 | 59.20239 | 11.6633 | 6.849021 |
| 2.553674 | 2.4366 | 18.86983 | 5.633678 | 31.44 | 44.65802 |
| 2.804007 | 15.0088 | 11.78605 | 3.942905 | 3.864225 | 3.344292 |
| 2.686619 | 2.7371 | 33.80315 | 12.41604 | 15.72 | 28.19522 |
| 1.795628 | 2.4104 | 8.6903 | 9.023605 | 7.23886 | 5.190454 |
| 2.554193 | 3.161 | 10.38427 | 12.81127 | 5.62484 | 27.18488 |
| 7.295734 | 1.7419 | 11.54189 | 58.26698 | 14.05835 | 5.752425 |
| 6.912 | 4.4362 | 20.04875 | 33.292 | 23.78762 | 18.02064 |
| 5.748478 | 5.6036 | 4.093789 | 3.744001 | 104.8 | 115.94 |
| 11.98452 | 3.3731 | 58.14246 | 4.663998 | 3.288977 | 4.851826 |
| 7.018988 | 2.6054 | 28.536 | 4.560559 | 11.04868 | 12.17028 |
| 12.03821 | 5.2409 | 21.74566 | 65.67837 | 4.727744 | 4.281328 |
| 2.890218 | 2.1192 | 4.756 | 2.854653 | 2.802454 | 2.371888 |
| 2.996777 | 5.6303 | 30.06599 | 28.20178 | 5.094601 | 7.939787 |
| 15.25033 | 11.0318 | 3.100139 | 4.499602 | 52.4 | 57.65045 |
| 29.55895 | 4.6592 | 3.287922 | 3.414959 | 10.88965 | 7.86 |
| 4.88741 | 4.8321 | 12.69055 | 6.802944 | 5.327309 | 3.843181 |
| 22.64 | 18.112 | 16.46848 | 14.43803 | 9.749148 | 10.68592 |
| 8.218758 | 3.1792 | 8.492529 | 10.59346 | 8.35953 | 13.392 |
| 4.535957 | 7.3777 | 33.292 | 28.88515 | 15.72 | 18.50206 |
| 4.582239 | 4.8686 | 9.820777 | 3.043217 | 13.69134 | 15.72 |
| 3.250575 | 4.9698 | 25.70532 | 3.068878 | 28.87898 | 31.05553 |
| 3.557401 | 2.8898 | 20.48627 | 4.942371 | 8.155665 | 10.75137 |
| 3.439445 | 1.9792 | 11.6506 | 12.97283 | 94.32 | 114.8088 |
| 2.433551 | 4.1689 | 5.100664 | 4.080915 | 18.34 | 18.45294 |
| 5.484277 | 4.1479 | 10.10359 | 3.215511 | 18.11912 | 82.66842 |
| 5.677748 | 3.1145 | 14.21677 | 6.385443 | 2.523021 | 4.683916 |
| 2.551856 | 1.9444 | 11.74525 | 14.0952 | 4.94235 | 13.47468 |
| 4.362887 | 4.3629 | 19.25635 | 5.795566 | 19.79869 | 19.65129 |
| 9.759302 | 2.0507 | 2.935901 | 22.20722 | 9.956975 | 2.894613 |
| 3.193519 | 3.1733 | 7.461199 | 26.40738 | 5.176429 | 47.4445 |
| 3.325091 | 2.3559 | 5.624152 | 31.93346 | 20.56708 | 20.96 |
| 15.37976 | 6.0984 | 28.11498 | 36.95961 | 6.536693 | 72.10896 |
| 5.277694 | 2.6383 | 15.40432 | 5.716159 | 4.512649 | 6.699312 |
| 2.238747 | 4.1358 | 5.468887 | 11.6 | 6.596065 | 22.17701 |
| 3.668824 | 7.1915 | 11.29071 | 5.185051 | 42.81627 | 8.545804 |
| 9.628066 | 6.755 | 6.650562 | 4.566713 | 69.37469 | 26.95373 |
| 6.263067 | 6.0779 | 22.85333 | 18.16357 | 41.92 | 47.21339 |
| 7.98 | 2.3091 | 11.34255 | 11.31928 | 24.104 | 24.34568 |
| 9.938398 | 2.0688 | 11.23647 | 6.875034 | 11.85794 | 12.18754 |
| 5.828238 | 3.4218 | 3.263785 | 6.172172 | 23.71052 | 18.85046 |
| 4.865594 | 3.1489 | 9.151642 | 5.564797 | 37.69864 | 41.92 |
| 1.931879 | 3.9545 | 4.317423 | 12.20327 | 6.997093 | 4.008664 |
| 5.428665 | 2.6895 | 13.65374 | 2.534455 | 10.99551 | 2.565303 |
| 2.728605 | 2.0959 | 4.093367 | 2.621538 | 5.002076 | 5.44991 |
| 14.74163 | 22.7501 | 56.48565 | 7.440255 | 14.79817 | 10.48 |
| 2.660927 | 15.285 | 7.363136 | 7.490087 | 20.86041 | 22.91486 |
| 3.448 | 3.823 | 8.582711 | 5.426985 | 15.6925 | 8.287561 |
| 3.345788 | 3.7618 | 14.0195 | 38.14431 | 19.55123 | 7.651988 |
| 7.121679 | 9.5948 | 3.681243 | 6.641241 | 5.637322 | 8.907678 |
| 5.143608 | 3.903 | 10.356 | 4.943624 | 3.559543 | 2.966001 |
| 57.42834 | 2.4621 | 5.618184 | 3.540576 | 21.2995 | 26.9591 |
| 5.017775 | 11.3161 | 4.038611 | 20.62184 | 108.9265 | 21.46039 |
| 0 | 2.0422 | 4.724098 | 6.951902 | 6.576391 | 15.98849 |
| 6.912 | 2.7465 | 4.14787 | 4.525776 | 23.94 | 25.18848 |
| 3.963579 | 3.0758 | 3.301596 | 5.207004 | 23.07803 | 65.70152 |
| 12.17701 | 7.0486 | 3.00911 | 2.456735 | 67.48552 | 66.65338 |
| 2.220232 | 5.7598 | 3.347509 | 4.186285 | 7.488061 | 12.88209 |
| 2.617632 | 6.0937 | 3.199267 | 2.516143 | 9.317923 | 5.847487 |
| 6.912 | 3.2669 | 3.093 | 2.039892 | 36.88481 | 34.65503 |
| 14.29862 | 4.2884 | 10.23245 | 14.87417 | 26.7195 | 29.80286 |
| 8.3424 | 10.428 | 2.256181 | 2.15596 | 10.04902 | 5.734363 |
| 63.55521 | 12.151 | 20.21056 | 3.457387 | 5.241351 | 3.85759 |
| 3.89312 | 3.476 | 5.117818 | 6.920414 | 3.452 | 3.242165 |
| 3.22841 | 7.6775 | 7.814362 | 2.827056 | 2.041199 | 2.476577 |
| 2.727866 | 4.1365 | 3.229538 | 4.289584 | 2.46231 | 5.520265 |
| 4.8664 | 4.5188 | 14.38093 | 14.53407 | 64.54614 | 8.828007 |
| 6.792 | 4.8791 | 3.147649 | 5.276363 | 9.576 | 2.980238 |
| 20.664 | 2.7059 | 8.245511 | 4.17855 | 3.561221 | 4.928498 |
| 9.825853 | 3.1289 | 4.082891 | 4.41585 | 5.307453 | 4.781891 |
| 1.936576 | 3.0996 | 4.883058 | 7.96413 | 14.364 | 14.364 |
| 3.321405 | 2.109 | 2.955067 | 14.9389 | 8.092696 | 13.58019 |
| 3.2 | 4.1872 | 1.631502 | 15.42514 | 5.400084 | 6.911832 |
| 11.36352 | 1.8651 | 11.32264 | 10.71903 | 4.192 | 4.517727 |
| 4.788 | 3.8535 | 4.943254 | 4.119087 | 10.48 | 10.76621 |
| 2.738304 | 3.2395 | 4.769373 | 12.19399 | 16.38962 | 21.26379 |
| 10.13336 | 2.199 | 9.248603 | 3.738565 | 8.524077 | 7.768938 |
| 3.694936 | 2.3783 | 8.585351 | 12.86397 | 4.951919 | 3.784734 |
| 20.29995 | 9.9959 | 31.37928 | 45.26386 | 54.53456 | 31.82344 |
| 19.2 | 8.4461 | 7.449515 | 6.444702 | 18.43153 | 15.72 |
| 3.358026 | 2.9393 | 8.070379 | 7.868469 | 7.008271 | 10.00299 |
| 1.69816 | 3.3809 | 9.660952 | 11.59282 | 20.3312 | 20.34328 |
| 34.1977 | 12.4411 | 3.632943 | 3.36488 | 11.98683 | 4.400796 |
| 8 | 10.9104 | 2.486411 | 2.644346 | 14.31069 | 10.48 |
| 1.573825 | 5.782 | 3.955693 | 3.049701 | 7.410784 | 3.19499 |
| 2.720311 | 2.8354 | 5.863921 | 14.84253 | 8.636512 | 12.79606 |
| 10.344 | 3.355 | 5.486964 | 2.702539 | 9.348271 | 36.92099 |
| 9.260092 | 7.1817 | 3.529137 | 3.444749 | 28.728 | 28.57876 |
| 3.786906 | 2.5265 | 5.382365 | 2.45862 | 55.39901 | 16.758 |
| 2.085686 | 2.0736 | 4.352672 | 3.326393 | 16.758 | 11.04168 |
| 5.665702 | 5.184 | 4.502095 | 3.037866 | 11.03516 | 9.576 |
| 7.598702 | 3.0769 | 8.471011 | 4.40824 | 37.16011 | 46.89233 |
| 5.478673 | 4.1859 | 10.46902 | 4.852696 | 8.326423 | 10.21983 |
| 2.580285 | 3.3137 | 3.292712 | 4.463066 | 28.728 | 32.0796 |
| 6.466073 | 7.5609 | 5.233418 | 28.38799 | 17.03032 | 25.78384 |
| 3.521565 | 5.184 | 6.421883 | 16.05524 | 14.364 | 14.364 |
| 12.61813 | 4.3945 | 4.230835 | 16.6935 | 3.176077 | 8.522224 |
| 6.180536 | 8.9901 | 12.3656 | 6.335285 | 4.945804 | 4.927797 |
| 8.517152 | 26.2162 | 14.21681 | 3.409565 | 10.71772 | 5.62631 |
| 3.899792 | 3.5452 | 40.25879 | 10.72246 | 3.896059 | 3.596996 |
| 8.175017 | 3.1284 | 16.91241 | 3.365073 | 14.42391 | 19.37819 |
| 2.844584 | 3.6111 | 3.529137 | 16.26064 | 5.087801 | 2.980238 |
| 5.494823 | 4.3038 | 5.382365 | 3.853545 | 3.979028 | 4.928498 |
| 32.82863 | 3.444 | 4.352672 | 13.58729 | 36.8576 | 6.124216 |
| 4.889044 | 9.0021 | 4.502095 | 2.77841 | 4.522843 | 5.861135 |
| 3.80153 | 3.6638 | 8.471011 | 4.707877 | 8.092696 | 23.5451 |
| 12.81379 | 3.4902 | 10.46902 | 9.566106 | 6.126151 | 4.358956 |
| 2.806319 | 6.2809 | 3.292712 | 12.56672 | 19.08781 | 19.22214 |
| 1.741472 | 10.7663 | 5.233418 | 4.047766 | 13.74186 | 9.193792 |
| 5.1072 | 5.4264 | 6.421883 | 2.966025 | 21.50934 | 36.02395 |
| 2.839118 | 6.218 | 4.230835 | 10.28531 | 5.961657 | 8.258353 |
| 5.956603 | 35.5398 | 12.3656 | 6.181683 | 15.72 | 15.72 |
| 13.7556 | 6.1463 | 14.21681 | 4.421493 | 26.2 | 26.2 |
| 3.358026 | 2.8363 | 40.25879 | 19.78915 | 7.86 | 7.959581 |
| 4.557071 | 0.96 | 16.91241 | 3.201107 | 20.96 | 20.43055 |
| 5.473796 | 3.161 | 10.71997 | 9.289862 | 68.14359 | 53.57064 |
| 5.528831 | 5.44 | 27.30263 | 34.32917 | 4.460581 | 4.460581 |
| 3.26451 | 2.7355 | 3.383904 | 5.447194 | 9.647473 | 5.727086 |
| 4.393434 | 6.2064 | 2.42009 | 3.73152 | 3.910899 | 3.820036 |
| 6.092135 | 6.3309 | 1.835944 | 1.107111 | 4.913946 | 6.101494 |
| 24.108 | 2.6639 | 3.551778 | 10.93162 | 63.43947 | 23.07911 |
| 6.037441 | 5.4994 | 17.61869 | 10.00842 | 10.79028 | 10.46867 |
| 24.6269 | 6.2208 | 5.074654 | 3.736212 | 26.06103 | 17.60927 |
| 7.740785 | 12.5688 | 3.290667 | 3.2832 | 18.1944 | 16.758 |
| 0.0414996 | 14.5065 | 13.09328 | 9.669409 | 4.185968 | 17.4015 |
| 10.0224 | 9.849 | 11.46244 | 4.103673 | 44.98119 | 66.10938 |
| 7.063283 | 8.6315 | 1.736524 | 3.788744 | 6.113364 | 7.120799 |
| 3.203008 | 2.6039 | 6.036556 | 7.742755 | 16.64906 | 32.44286 |
| 2.67944 | 3.0135 | 8.042381 | 4.836401 | 16.758 | 17.2368 |
| 1.902183 | 3.6062 | 5.722603 | 3.542949 | 7.182 | 7.182 |
| 2.29982 | 3.125 | 7.86734 | 8.0852 | 6.661942 | 15.74102 |
| 5.8274 | 2.4214 | 37.58011 | 3.809833 |  |  |
| 2.868628 | 5.166 | 3.779006 | 8.879211 |  |  |
| 4.1328 | 4.1328 | 4.650233 | 3.419712 |  |  |
| 6.812217 | 6.1336 | 3.66892 | 12.36487 |  |  |
| 2.589866 | 2.9785 | 33.17717 | 10.53389 |  |  |
| 23.66896 | 6.888 | 3.452 | 2.473013 |  |  |
| 2.048273 | 3.7139 | 8.770119 | 10.356 |  |  |
| 2.018917 | 6.4107 | 2.605158 | 2.34736 |  |  |
| 8.527147 | 10.3074 | 80.8926 | 41.69542 |  |  |
| 2.887135 | 2.6371 | 17.10024 | 3.624008 |  |  |
| 4.102511 | 3.1391 | 18.85584 | 8.760658 |  |  |
| 2.046643 | 2.8694 | 7.9396 | 6.904 |  |  |
| 5.241838 | 3.3317 | 2.706046 | 2.183041 |  |  |
| 3.273678 | 4.2903 | 3.452 | 3.452 |  |  |
| 2.982124 | 7.0377 | 7.061175 | 5.046023 |  |  |
| 7.840778 | 2.3015 | 2.822476 | 6.003527 |  |  |
| 10.344 | 0.5172 | 55.48829 | 31.77737 |  |  |
| 24.108 | 2.6519 | 3.806 | 4.186863 |  |  |
| 6.037441 | 3.835 | 2.498107 | 2.765736 |  |  |
| 24.6269 | 7.5379 | 2.678596 | 2.6642 |  |  |
| 7.740785 | 2.479 | 4.818702 | 2.888728 |  |  |
| 0.0414996 | 2.6655 | 11.15002 | 3.292812 |  |  |
| 10.0224 | 2.1936 | 6.422252 | 4.779482 |  |  |
| 7.063283 | 5.1889 | 8.614656 | 2.131859 |  |  |
| 3.203008 | 2.7622 | 7.153411 | 3.6183 |  |  |
| 2.67944 | 3.2104 | 10.368 | 11.45115 |  |  |
| 1.902183 | 4.7769 | 32.42147 | 9.617666 |  |  |
| 2.29982 | 5.0762 | 6.459958 | 4.029834 |  |  |
| 5.8274 | 4.2568 | 4.528 | 3.77218 |  |  |
| 2.868628 | 3.1322 | 5.26073 | 6.241338 |  |  |
| 4.1328 | 8.4378 | 7.823114 | 5.343068 |  |  |
| 6.812217 | 4.8503 | 31.72277 | 36.60764 |  |  |
| 2.589866 | 2.238 | 3.779006 | 5.93465 |  |  |
| 23.66896 | 2.2785 | 4.529159 | 7.164807 |  |  |
| 2.048273 | 29.6054 | 6.989027 | 9.470302 |  |  |
| 2.018917 | 4.4539 | 13.62109 | 4.176031 |  |  |
| 8.527147 | 4.0829 | 10.36083 | 3.452 |  |  |
| 2.887135 | 5.6872 | 2.234349 | 21.91487 |  |  |
| 4.102511 | 3.0922 | 3.03809 | 3.769963 |  |  |
| 2.046643 | 3.1485 | 41.424 | 34.52 |  |  |
| 5.241838 | 2.756 | 2.010795 | 6.986239 |  |  |
| 3.273678 | 3.3606 | 2.199527 | 2.4164 |  |  |
| 2.982124 | 3.3299 | 10.70601 | 17.26 |  |  |
| 7.840778 | 2.6177 | 3.193554 | 3.17584 |  |  |
| 10.344 | 5.172 | 5.140612 | 3.549198 |  |  |
|  |  | 2.561346 | 2.913561 |  |  |

**Statistical report
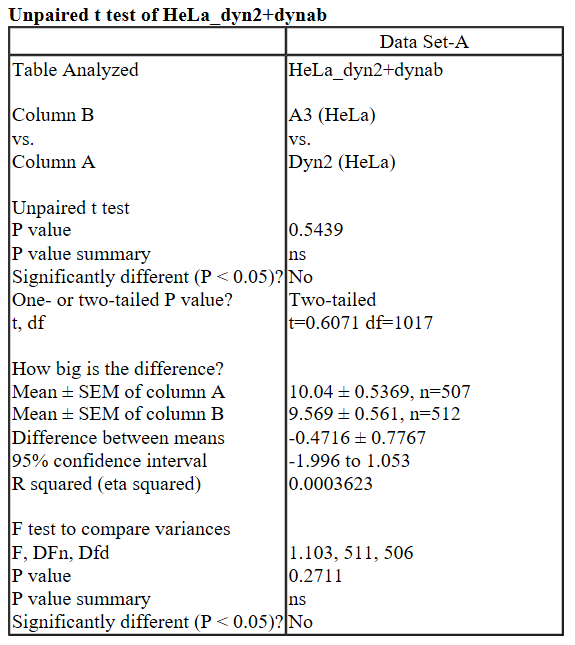

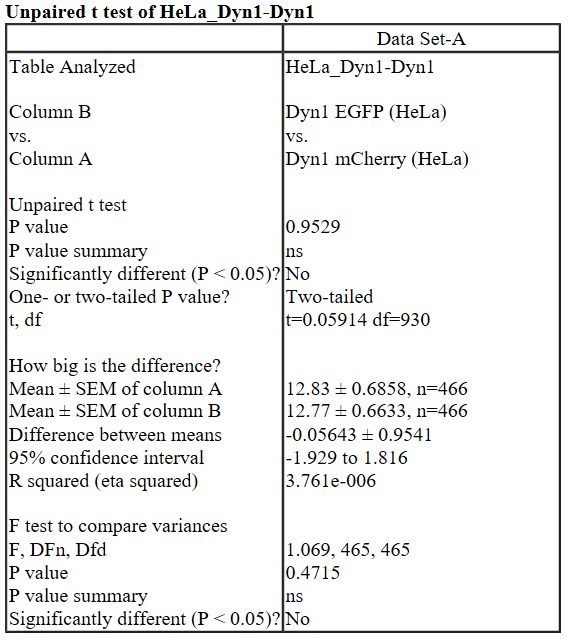

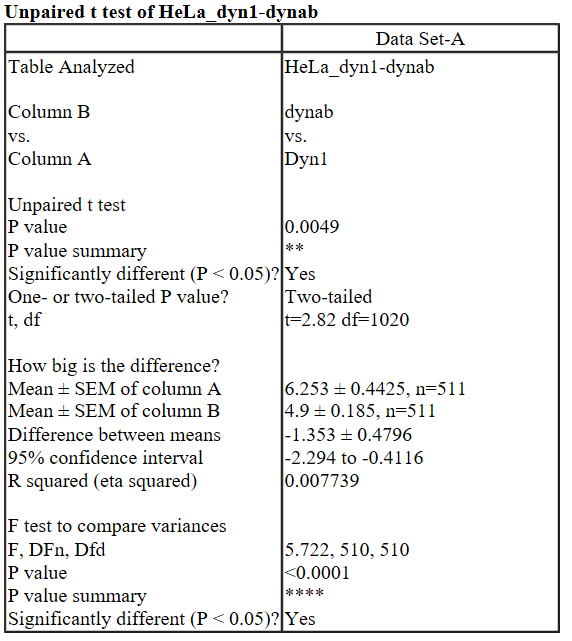
:**
