# Supplementary material for: Uncoupling of dynamin polymerization and GTPase activity revealed by the conformation-specific nanobody dynab
Source: eLife. 2017 Oct 12;6:e25197. doi: 10.7554/eLife.25197 (PMC5658065; doi:10.7554/eLife.25197)
Supplement: Figure 4—source data 4. [file elife-25197-fig4-data4.docx]

**Figure 4-Source Data 4 (panel C)**

Cumulative probability of time difference between *t*(dynab)-*t*(dyn1-2) and DYNctrl in TKO cells, and statistical report

| *t*(dynab)-*t*(dyn1) | *t*(dynab)-*t*(dyn2) | *t*(dyn1_EGFP)-*t*(dyn1_mCherry) |
| --- | --- | --- |
| -0.75 | -0.5 | -0.5 |
| 0 | 1.5 | -0.75 |
| -0.25 | -0.25 | 0.25 |
| 0 | 0.25 | 0.5 |
| 1.25 | 0 | -0.75 |
| -0.75 | -0.25 | 1 |
| 0.75 | 0.5 | 1.25 |
| 0 | 0.5 | 2.25 |
| 1 | -0.25 | -0.5 |
| -2 | -0.25 | 0 |
| 1 | -1.75 | 1.25 |
| -2.25 | -1.25 | 0.5 |
| -0.75 | 0.5 | -2 |
| 0.75 | 0.5 | 0 |
| 1.5 | 1 | -3.75 |
| -3.25 | 1 | 1 |
| 2.25 | -1 | 0 |
| 1 | -1.5 | 1.25 |
| -0.75 | 1.5 | 1.25 |
| 1.75 | -1.5 | 0 |
| 0.5 | 0.5 | 0.75 |
| 0.75 | -0.5 | 0.75 |
| 2.25 | 1.5 | 0.5 |
| -0.5 | 0 | 0.5 |
| -5.25 | 0 | 0.25 |
| -0.25 | -2 | 0.25 |
| 4 | 0.25 | 0 |
| 0.5 | -1 | 0.5 |
| 1.25 | 0.25 | -0.75 |
| 5.5 | -0.75 | 0.25 |
| 0.5 | 1.5 | 0.5 |
| -1.5 | -0.25 | 0.75 |
| 0.75 | 0.25 | 0.75 |
| 2.75 | 3 | 1.25 |
| -1 | 2.25 | 1 |
| -2.25 | -0.5 | -0.25 |
| -3.5 | 0.5 | 0 |
| -0.25 | -3.25 | 0.25 |
| -1.75 | -0.5 | -1.25 |
| 3 | 0.25 | 0.5 |
| 0.25 | -0.25 | -0.25 |
| 4.5 | 1.25 | 1.25 |
| -2.25 | 0 | 0.75 |
| 2 | 0.5 | 0.5 |
| 0.5 | 0 | 0.75 |
| 0.5 | 3.5 | 0.5 |
| -5 | 0.5 | -2.75 |
| 0.25 | 1 | 0.5 |
| -0.5 | 1.5 | 0.25 |
| 1.25 | 0.25 | 1.5 |
| 1 | -3.5 | -1 |
| 2.5 | 0 | 0.25 |
| 0.25 | 0.25 | -0.25 |
| 0.75 | 0.25 | 1.25 |
| -1.25 | -0.5 | 0.25 |
| 0.5 | -0.25 | 0.25 |
| -3 | -1.5 | 0.25 |
| 1 | 0 | 0.5 |
| -3.25 | -0.5 | 0 |
| -4.25 | 0.25 | 0.75 |
| 5.75 | -0.25 | 1 |
| 1.25 | 0 | 0.25 |
| -0.75 | -6 | 0.25 |
| 7 | -1.25 | 1 |
| -0.5 | 0.5 | -0.25 |
| 0 | 0 | 0.5 |
| 0.5 | 1 | 0.25 |
| 2.5 | -1.25 | 1 |
| 1.5 | 0 | 0 |
| -0.25 | 3.5 | 0.25 |
| 0.25 | 0.5 | 0 |
| 2.25 | 0 | 0.5 |
| -1.5 | 2.75 | 0.75 |
| -2.75 | 1.25 | 0.5 |
| 3.5 | 0 | 0.25 |
| 0.5 | -0.5 | 0 |
| 0.5 | 0.25 | 0 |
| -2.5 | 2 | 0.25 |
| 2 | 0 | 0.75 |
| -0.75 | -0.5 | -0.25 |
| -2.25 | 3 | -0.25 |
| 0 | 0 | 0 |
| 2.75 | -0.25 | 0.75 |
| -1.5 | 1 | 0.5 |
| -2.5 | 0.25 | -0.5 |
| -0.75 | 0 | 0.5 |
| -2 | -0.75 | 1 |
| -1.5 | 0.25 | 0.25 |
| -1.5 | -1 | 1.25 |
| 0 | -0.5 | 0.25 |
| -1 | -3.75 | 0 |
| -6.25 | 0.25 | 0 |
| -2 | -2 | 0.5 |
| -0.75 | 2.25 | 0.5 |
| -1.25 | 0.5 | 0.25 |
| -5.75 | -0.75 | 0.75 |
| 1.25 | -0.5 | 0.5 |
| 1 | 2 | 1.25 |
| -3 | -1.25 | -1 |
| 0 | -1.5 | 0.25 |
| 1 | 0.5 | 0.5 |
| -0.5 | 1.75 | 0.75 |
| -3 | 0 | 3.25 |
| -1 | -2.25 | 0 |
| -0.5 | 0.25 | 1.25 |
| -1.5 | 0 | 1.25 |
| -1.75 | 0.25 | 0.25 |
| -0.5 | 3.25 | -0.5 |
| -1 | 2.5 | 1 |
| -0.75 | 0.75 | 3.5 |
| 0.5 | 0.75 | 0.5 |
| 0.25 | 0.75 | 0.75 |
| -3 | -0.5 | 0.25 |
| -0.5 | -0.5 | 1 |
| 1 | 0 | 0.5 |
| 0 | 0.5 | 0.25 |
| -1.75 | 0.5 | 0.75 |
| 0.75 | -1 | 0.25 |
| 0 | 1.25 | 0.5 |
| 0 | -0.25 | 0.75 |
| 1.75 | 1.25 | 0.25 |
| -0.25 | 1 | 0.25 |
| 2 | 1.5 | 0.75 |
| -7 | -1.25 | 0.25 |
| -1.5 | 1 | 0.25 |
| 0.25 | -1 | -0.25 |
| -0.25 | 0.75 | 0.75 |
| -0.5 | 0.5 | -0.75 |
| 1.5 | -0.5 | -0.25 |
| -2.75 | -0.75 | 2.75 |
| -1 | -0.5 | 0 |
| 0 | -0.25 | 1 |
| 0.25 | -3.75 | 0.5 |
| -1.5 | -1.5 | 0 |
| 0 | -3.5 | 0.75 |
| -0.25 | -5 | -0.5 |
| 2.5 | -2.25 | 0.75 |
| -0.25 | -2.25 | 0 |
| 0 | -1.25 | 1 |
| 1.25 | 0.5 | 0.25 |
| -0.5 | 0.5 | -0.25 |
| 1 | 0.75 | 0 |
| -0.5 | 0.25 | 0.5 |
| 0.25 | 0 | 0.5 |
| -0.25 | 0.5 | 0.25 |
| -0.25 | 0 | 0 |
| 0.75 | 0 | -1.25 |
| 0.75 | -1 | 0 |
| -2.75 | 1.75 | 0.5 |
| 1 | -0.25 | 1 |
| -0.25 | -1.75 | 0.25 |
| 0 | 0.25 | 1.25 |
| -0.5 | 0 | 0.5 |
| 0.25 | -0.75 | 0.5 |
| 3 | 2.75 | 0.75 |
| 1 | -0.5 | 0.75 |
| 0.25 | 0.25 | -1 |
| -9.25 | 0.75 | 0.25 |
| 0.5 | 1.5 | 0.75 |
| 0.75 | -1.25 | 0.5 |
| -1 | -0.25 | 0.75 |
| -9.5 | 0 | 0 |
| -0.25 | 1 | -1.5 |
| 0 | -1 | 0.75 |
| 2 | 0.75 | 1.5 |
| 0.75 | 1 | -0.25 |
| 0.5 | 1.75 | 0.5 |
| 0.25 | 1.75 | -1.5 |
| -0.25 | 0.25 | 0.25 |
| 0.5 | 1 | -0.75 |
| -0.25 | -0.25 | 1.25 |
| 4 | -4.5 | 0 |
| 1.75 | -0.25 | 0.5 |
| 0.25 | 0.25 | 0.75 |
| 0.5 | 1.25 | 0 |
| -1.25 | 0.5 | 0.75 |
| 0 | 0.5 | 0.25 |
| -0.5 | -7.75 | 1 |
| 0 | 2 | 0.25 |
| 0.75 | 4.25 | 0.25 |
| 0 | 0.25 | -1 |
| 0.25 | -0.5 | 1.5 |
| -1.5 | 2.25 | 0.5 |
| 0 | -0.25 | 0.25 |
| 0.25 | -0.25 | 0.5 |
| 0.25 | 2 | 1.25 |
| 1.75 | -2.25 | 1.25 |
| 0.75 | 0.25 | 0.25 |
| -0.25 | -0.5 | -0.25 |
| 0.5 | -0.25 | 1.25 |
| 0.25 | 0 | 0.75 |
| 1.25 | 0 | -0.75 |
| -0.5 | 0.25 | -0.5 |
| 0.25 | -0.75 | 0 |
| 0.75 | 0.25 | -0.25 |
| 2 | 0.75 | 0 |
| 0.5 | 0.25 | 0.25 |
| 0.25 | -1.25 | 0.25 |
| -0.5 | -5.25 | 1.5 |
| -0.25 | -1.5 | 1.25 |
| 0 | 2 | 1.25 |
| 0 | 0.75 | 1.5 |
| 0.5 | -0.5 | 0.5 |
| 0.25 | 0.75 | 2.75 |
| -2 | 1.25 | 1 |
| -0.75 | 1 | -0.25 |
| 0 | -2.5 | 0.25 |
| 0 | 0 | -0.75 |
| 0 | 1.5 | 3 |
| -0.5 | -0.5 | 0 |
| 0 | 0 | 1 |
| 0.75 | -1.5 | 1 |
| 0.75 | -0.25 | 1.5 |
| -0.75 | 0 | -2 |
| -0.75 | -0.75 | 1.25 |
| 0.5 | 0 | -0.25 |
| -0.25 | 1.5 | 0.25 |
| -1.25 | 0.5 | 0.25 |
| -0.75 | 2.5 | 1.5 |
| 0 | 5.5 | 1.25 |
| 0.5 | 2.75 | -1.25 |
| -0.25 | 0 | 0.25 |
| 0 | -1.75 | 0.75 |
| 0 | -1.75 | 1.5 |
| 0 | 0.5 | -0.25 |
| 0.25 | 0.5 | 1.75 |
| 0 | 6.25 | 1 |
| 1 | 2.25 | -0.5 |
| 0.25 | 0.75 | 0 |
| 0.75 | 0.5 | 0.75 |
| 0.75 | 0.75 | 1.5 |
| -1 | 2 | 0.5 |
| -0.5 | -0.5 | -3 |
| 1 | -2 | 0.75 |
| -0.75 | 0.75 | 1.75 |
| 0.75 | 1.5 | 0 |
| -0.5 | 0 | -0.25 |
| -0.5 | 1.25 | -0.75 |
| 1 | 2.5 | 1.25 |
| 0.25 | -1.5 | 0.5 |
| 0.75 | 1 | 0 |
| 0.5 | -0.25 | 0.5 |
| 0.25 | 1.25 | 1.5 |
| 0.25 | 0.25 | 1.5 |
| 1 | 0.25 | 2 |
| -0.5 | -0.25 | 0 |
| -2 | 1.5 | 0.75 |
| 0.25 | 0.5 | 1.5 |
| 0 | -0.25 | 0.5 |
| -0.5 | -0.25 | -2 |
| -0.75 | 0.25 | 0.25 |
| -1.5 | 0 | 0.25 |
| 0.25 | 1.75 | 1 |
| 1 | -0.25 | 1 |
| -0.25 | -0.25 | 0.25 |
| 0.25 | 2.5 | -0.25 |
| 0.75 | -3 | 1.25 |
| 1.75 | -0.25 | 0.25 |
| 2.25 | -0.75 | 1 |
| -0.5 | 1.5 | 0.5 |
| 0 | 1 | 1.25 |
| 0.5 | -0.25 | 0.75 |
| 0.25 | 0 | 0.75 |
| -0.25 | 1.75 | 1 |
| 2.75 | -0.75 | -0.75 |
| 3 | 0.5 | 0.25 |
| -1.75 | 0.25 | 2 |
| -0.25 | -0.25 | 0.75 |
| 2.5 | 1 | -0.25 |
| 0.25 | 3.5 | 0.5 |
| 1 | -0.75 | 1 |
| -0.75 | -0.25 | 2.5 |
| 0.25 | -0.5 | 0 |
| 0.25 | -1.75 | -0.5 |
| 2.25 | -1 | 0 |
| 1 | 1.25 | -0.25 |
| 1.25 | -0.25 | 1.25 |
| 0 | -1.25 | -1.25 |
| 0.5 | -0.5 | 0.25 |
| 0 | -0.75 | 0.25 |
| -1 | 1.5 | 0.75 |
| 1 | -1 | 0 |
| 1 | 3.75 | -2.5 |
| -1.25 | 0.75 | 1.5 |
| -1 | 0.25 | 5 |
| 0.25 | -1.5 | -1.25 |
| 0.25 | -1.75 | 0.25 |
| -2 | 0 | 0.25 |
| -0.75 | 1.75 | 3 |
| 1 | 0.25 | 0.25 |
| -1 | 0.5 | 0.25 |
| 0.25 | -0.25 | 0.25 |
| -2 | -1.75 | 0.5 |
| 0.5 | -0.75 | 0 |
| -0.75 | 0 | 1.75 |
| 0.25 | 5.5 | -0.25 |
| -0.25 | 0 | 0.75 |
| 0.25 | -2 | -0.25 |
| -6.75 | -0.25 | 0.75 |
| 0.25 | -0.25 | -1.5 |
| -2 | -0.25 | 1 |
| 0.25 | -3.5 | 0 |
| -2.75 | 0 | 0.25 |
| 2 | -0.5 | 0 |
| 0 | 0 | 0.25 |
| -2.75 | 1 | 0.75 |
| -1.75 | -0.5 | 2 |
| -0.5 | 0.5 | -0.75 |
| 0.75 | 1.75 | -0.5 |
| 0.25 | 1 | -1 |
| -0.5 | 1.25 | -0.75 |
| 1.5 | 0 | 0.25 |
| -0.75 | 0.5 | -1.25 |
| -1 | 0.5 | -0.5 |
| 0.5 | -1 | 0.75 |
| -0.25 | 0.25 | 0.25 |
| 0.75 | 0.75 | -0.75 |
| 1.75 | 1.75 | 1 |
| 0.75 | -0.75 | 0.75 |
| 0.25 | 0.25 | 1.25 |
| 0.25 | -0.5 | 1.25 |
| 0.25 | 0 | 1.5 |
| -0.5 | 0 | 0.5 |
| -1 | 1.25 | 0 |
| 1 | 1.25 | 1.25 |
| 0.5 | 2.75 | 0.75 |
| 0 | -0.5 | 0.5 |
| 0.5 | -0.5 | 0.25 |
| -0.5 | -0.25 | 2 |
| -0.25 | -1 | 1.5 |
| -2.75 | -1.25 | 1.75 |
| 1.25 | -0.5 | 1.5 |
| 0.75 | 0 | 2 |
| 3.5 | 0.25 | -0.5 |
| 2.5 | 1 | -3.5 |
| 1.25 | 0.5 | 0.75 |
| 0 | -1 | 0.75 |
| 0.75 | -1.75 | 0.5 |
| -1.75 | -0.5 | 0 |
| 0.75 | -0.5 | 0.5 |
| 0.75 | -1.25 | 0.25 |
| -0.75 | 0.75 | -0.25 |
| 0.5 | 1.25 | -0.25 |
| 0.5 | 0.25 | 1.25 |
| 1.25 | 0.5 | -0.25 |
| -3.5 | 0 | -0.5 |
| -0.75 | 1.25 | 0.75 |
| 2.5 | 0.25 | -0.75 |
| 1.5 | 0.25 | -0.5 |
| 0 | 0 | -0.25 |
| -0.5 | 1 | 0.25 |
| -1.5 | -0.75 | -1.5 |
| 0.25 | -0.5 | -1.5 |
| -0.25 | 2 | 0.75 |
| -0.75 | 2 | 0.25 |
| 1.25 | 0.25 | -1.25 |
| -0.25 | 2.75 | 0 |
| 0.75 | 1.5 | 1 |
| -1 | 0 | 0.5 |
| 3.5 | -0.5 | -7 |
| 0.75 | 1.25 | 0.25 |
| -0.25 | -0.25 | 0.25 |
| -1.25 | -0.25 | -0.5 |
| -3 | 0.75 | 1.5 |
| -1 | -1.25 | -0.25 |
| 1 | -1.25 | 0 |
| 0.5 | 1.5 | 0.75 |
| -2.5 | -0.25 | -1 |
| 0.25 | 0.75 | 0.5 |
| 2.75 | -0.5 | 0.75 |
| 1.5 | 0.25 | 0 |
| -0.25 | 1.25 | -0.25 |
| 0 | -0.75 | 0.25 |
| 0.25 | 0 | 0.5 |
| 1 | 0.25 | 0.75 |
| 0.25 | -0.75 | 0.25 |
| -1.5 | 1 | 0.5 |
| 0.75 | 0 | 0.25 |
| 0.5 | 1.75 | 1.75 |
| -1.75 | 1.5 | 0.5 |
| -4 | -0.25 | 1 |
| -1 | -0.25 | 0.5 |
| -5.25 | 0.25 | 0.25 |
| 2.25 | -1 | 0 |
| 0.5 | 2.75 | 0.5 |
| -0.25 | -0.25 | 0 |
| 0 | 1 | -0.25 |
| 1 | -0.25 | -0.25 |
| 1.25 | 2.25 | 0 |
| 1.75 | 0.75 | 1 |
| -0.5 | 0.25 | 0.75 |
| 1.75 | 0.75 | 1.25 |
| -0.25 | 0.75 | 0.5 |
| 1 | -0.75 | -0.5 |
| -1 | 1.5 | 0 |
| 0.5 | 0.25 | -2.5 |
| 3.75 | 1 | 0.75 |
| 0.5 | 0 | 0.75 |
| 2.5 | -0.25 | 0.5 |
| -0.5 | 0.5 | -0.75 |
| -0.75 | 0.75 | 1 |
| 1.75 | 0 | 1 |
| 1 | 0 | -1.75 |
| 0 | 0.5 | 1.75 |
| -1 | 1.25 | -0.25 |
| 0 | 0.5 | 1.25 |
| 0 | 0.25 | 0.25 |
| 0.25 | 1.75 | 0.25 |
| 2.5 | -3 | -0.25 |
| 0 | 1.75 | -0.5 |
| 0.5 | -0.25 | 1.75 |
| 0 | 3.25 | 1.25 |
| 0.75 | 0.75 | 0.75 |
| 0 | 0.5 | 0.25 |
| -9.75 | 0.25 | 0.75 |
| -3 | 1.25 | 0 |
| -1 | 1.25 | -0.75 |
| 1.25 | 0.25 | 1.5 |
| 0.5 | -0.5 | -0.25 |
| -3 | 0 | 0.5 |
| -0.5 | 0.25 | 0 |
| -0.5 | 1 | 0.5 |
| 1.5 | -0.25 | -0.75 |
| 0.25 | 2 | -0.75 |
| 0.5 | 0.75 | 0.25 |
| -0.5 | 1 | -0.25 |
| -2 | 0.25 | 0.75 |
| -0.75 | 0 | 2.25 |
| -3.25 | -0.25 | 0 |
| 1.5 | 3.75 | 1.25 |
| -0.5 | 2.25 | 1.25 |
| -0.25 | 0 | 0.5 |
| 2.25 | 1.25 | 1.5 |
| -2 | 0.75 | 0 |
| -1 | 0.5 | 0.25 |
| -0.25 | -0.25 | 1 |
| 1 | 0.25 | 1.25 |
| -0.5 | 1.25 | 1.5 |
| 0.75 | 0.5 | 1 |
| 0.5 | 1 | -2.75 |
| 0.75 | 0.75 | 1.25 |
| 0.25 | 1.25 | -1.75 |
| 0.25 | 1 | 0.25 |
| 0.25 | -1.25 | 0.75 |
| 2 | 0 | 4.75 |
| 0.25 | -0.25 | 1 |
| -0.25 | 0 | 2 |
| 1.25 | 1.75 | 0.75 |
| 0.75 | 0.25 | 1.25 |
| 0 | 1.5 | 0.25 |
| 0.5 | 0.75 | 1.25 |
| -2 | 2.25 | -2 |
| 0.25 | 0 | 6.5 |
| 0.25 | -0.5 | 7.5 |
| 1 | 0.75 | 1.25 |
| -0.25 | 0.25 | 0.75 |
| -0.25 | 0.75 | 0 |
| 0.5 | 1.75 | 1.5 |
| 0 | 0 | -0.25 |
| -2 | 0.5 | 1.75 |
| -0.25 | -0.75 | -0.25 |
| 0.25 | -0.25 | -0.25 |
| -4.75 | 0 | 0.75 |
| -1.25 | -0.25 | 1 |
| 1.75 | 1.5 | 0.75 |
| 0.75 | 0.25 | 0.25 |
| -5.75 | 0.75 | 0.25 |
| 0 | 1 | 1.25 |
| -1 | 0.5 | -1.25 |
| 1 | 0.5 | 1 |
| 0.75 | 0.25 | 0 |
| -0.25 | 1 | -0.25 |
| -2.25 | -0.25 | 1 |
| -3.5 | 0.25 | 0.75 |
| 0.75 | -0.25 | 1.25 |
| -7.25 | 0.5 | -0.25 |
| -1 | -0.25 | 0.5 |
| 0.5 | -2.5 | 0.5 |
| -1.25 | 0.75 | -1 |
| -1.25 | 0.5 | -1 |
| 1.25 | 0.25 | 0.75 |
| -2.25 | 0.5 | 0.25 |
| -1.75 | 1.5 | 0 |
| -2.25 | 1 | -0.25 |
| 0.75 | 0.75 | 1.25 |
| 0.25 | 0 | -0.5 |
| 0 | 1.25 | -0.5 |
| -1.75 | -0.25 | -0.5 |
| -3.25 | -0.5 | 2.25 |
| 0.75 | -1.75 | 1 |
| -2.25 | 0.25 | 0.25 |
| -10.75 | 0 | 0 |
| 0 | 0.25 | 0.5 |
| 1 | -0.75 | 1.25 |
| -2.25 | 1 | 0.75 |
| 1.75 | 0.75 | 0.5 |
| 3.5 | 0.75 | -0.25 |
| -2.25 | 0.5 | 0 |
| -2 | 1 | -0.75 |
| -1.25 | 1 | -0.5764406 |
| -7.25 | 0.5 | -0.5 |
| -1.75 | 0.25 | 0.75 |
| -0.75 | -0.5 | -0.5 |
| 0.25 | 1 | 0 |
| -0.25 | 0.75 | -0.07441911 |
| -7.5 | -0.25 | 0.5501264 |
| 0 | 0.5 | -0.8009531 |
| -1.75 | 0.5 | -1.376759 |
| 0 | 0.75 | 0.06106839 |
| 0 | -0.75 | 0.4764821 |
| -1.5 | -0.25 | 0.3669562 |
| -0.75 | 0.25 | 0.01864358 |
| -2 | 1.25 | -1.49373 |
| -13.5 | 5 | -1.158053 |
| -1.25 | -0.5 | 0 |
| -2 | 0 | -1.108304 |
| -0.25 | 3.25 | 0.1196539 |
| -1.5 | 1.75 | 0.1924777 |
| 0 | -1.5 | 0.1235086 |
| 0.75 | 0.75 | 0.3271972 |
| -1 | 0.5 | 1.634052 |
| -1.75 | 3.75 | 1 |
| 2.75 | -0.5 | -0.5211162 |
| -1.25 | 0.25 | 0.5 |
| -4.5 | 0.5 | 0.5119804 |
| -0.75 | 0 | 0 |
| -0.75 | 2.25 | 0 |
| -1.5 | -0.25 | 0 |
| -2.75 | 1.5 | 0 |
| -5.25 | 0 | 1.12027 |
| 0.75 | 0 | 4.00557 |
| -0.5 | 0 | 0.8313016 |
| -2 | -2.824237 | 0.6958371 |
| -0.25 | 0.3792484 | 0 |
| -4.75 | -1.337697 | 0.5788913 |
| 12.25 | 0 | 0 |
| -2.5 | 0 | -0.004341594 |
| 0 | 0 | 1.272655 |
| 2.5 | 1 | -0.8325725 |
| -1.5 | 0 | -0.01132504 |
| -1.5 | -0.321531 | 2.177848 |
| -1.75 | -0.2480543 | -0.75 |
| -0.75 | 0.06277175 | 0.282946 |
| -0.75 | 0.07733179 | -0.7076656 |
| -0.25 | -12.47206 | 0.3652964 |
| -4.5 | -0.4032951 | -0.2742737 |
| 1.5 | -0.6688066 | 0.4518942 |
| 0.75 | 1.549349 | -1.019764 |
| -2 | -0.4529407 | 0 |
| -1.5 | -1.09298 | 0.1966299 |
| 1 | 1.501684 | 2.720398 |
| -3 | -0.0791491 | 0.1953907 |
| -0.25 | 0.4086779 | -0.04358058 |
| -0.5 | -0.4890216 | 1.234916 |
| 1 | 0 | -3.125821 |
| -3.5 | -0.5717012 | -0.4173019 |
| 1.25 | 9.347529 | -5 |
| -4 | 0.05491476 | -0.4285592 |
| 5 | -0.1413196 | 2 |
| -1.75 | 1.993506 | 0 |
| -2.25 | -0.0541375 | 0.06497461 |
| -1 | 0.5101524 | -0.75 |
| 0.75 | 0 | 1.544456 |
| -1.25 | -0.5440951 | 0 |
| -1.75 | -0.2386072 | 0 |
| 0 | 0 | 0.286415 |
| -0.5 | 0.5734777 | -0.3124517 |
| 0 | 0.1923684 | 0.7597711 |
| -1 | 0 | 0.6016282 |
| -1.5 | -1.067235 | 1 |
| -0.25 | 1.674466 | 0 |
| -1.5 | -2.209209 | 1 |
| 2.25 | 0 | -0.5 |
| 0.25 | -2.140365 | 1.5 |
| -1.25 | 0 | -1 |
| -2.25 | -3 | 0.02368977 |
| 0.25 | 0.3272251 | 0.9672429 |
| -3 | 0.75 | -0.03528014 |
| 4 | -0.5 | 0 |
| -0.75 | 0 | 1.5 |
| -0.25 | -0.75 | 0.75 |
| -1.5 | 0.6109035 | 0.2014141 |
| -2.75 | 0.5682002 | 0.6772096 |
| 1 | -0.6002493 | -0.4232557 |
| 1.75 | -1.143239 | 0.6275116 |
| 0.25 | 0 | 1.237902 |
| -3.5 | -1 | 0.01510578 |
| -1.5 | 0.5322049 | -0.25 |
| 0 | 1.220015 | 0.6134785 |
| 0.5 | 1 | -0.5 |
| 0.25 | -0.2922841 | 1 |
| 0.25 | 0.4790461 | -1.546014 |
| -1.5 | -0.0081155 | 0.75 |
| -1.25 | 7.437528 | 1 |
| 0.5 | 1.924726 | 0.9 |
| 0.75 | -0.7525567 | -0.25 |
| 0 | 1.186671 | 1.5 |
| 2.75 | 1.118939 | -0.25 |
| 1.25 | 0 | 0.5 |
| -3 | 0 | 0.01220214 |
| 0.75 | 155.75 | -1.25 |
| 0.25 | 0.4207747 | -1.25 |
| -0.25 | -3 | 1.335555 |
| -0.25 | 0 | 0 |
| 0.25 | 0 | -1 |
| -0.5 | 2.578743 | 0.6080493 |
| 0 | 0.2040572 | 0.6105297 |
| -1 | 0 | -0.5 |
| 2 | 0.04886844 | -0.1 |
| 0 | 1 | 0.75 |
| 2.5 | 0.0100611 | -2 |
| 2.75 | -0.0793207 | 1.25 |
| 1 | 0.4324554 | 0.1722378 |
| -2.5 | -0.8433091 | -0.1179225 |
| -0.5 | -0.2391857 | 1.725856 |
| -2.25 | 1 | 1.25 |
| 1 | 0.1607256 | 0.04413394 |
| -3.75 | 1.476775 | 0.75 |
| -5.25 | 0 | 1.5 |
| 0.5 | 0 | -1.605747 |
| -1.25 | -0.1077203 | 0.3161576 |
| 0.75 | 0 | 0 |
| -1.25 | 2.414764 | 0.5 |
| -8.25 | 1.75 | 0.3007147 |
| 0.75 | 1.25 | 0 |
| 1.5 | 1 | 0.7152175 |
| -0.25 | -0.0592399 | 0.2784831 |
| -2.5 | 1 | 0.9779472 |
| -0.75 | -1.616747 | 0 |
| -0.25 | 0 | 0.50389 |
| 0.75 | 0 | -0.03549191 |
| -2 | 0.06643286 | 0.2886852 |
| 0.5 | 0.4678962 | 0.25 |
| -1.25 | -0.1586718 | 0 |
| 2.25 | 3.741344 | 1 |
| -1 | -0.25 | 0 |
| 1.5 | 0.1034354 | 3.617127 |
| -0.5 | 0.75 | 3 |
| 0.5 | 0.5 | 0.1448367 |
| 0 | -1.077714 | 1.41121 |
| 0 | 0.3942733 | 1 |
| 1.5 | 1.25 | 0.25 |
| 4 | 1.218415 | 0.6836363 |
| 1.25 | 0.5518364 | 1 |
| -0.75 | 0.5 | -0.8776144 |
| -2.75 | 0.5 | 0.4038164 |
| 13.25 | 0.4938987 | 1 |
| 1.25 | -1.105408 | -0.5344319 |
| 1.25 | 0 | -1.123017 |
| 0.5 | 1.04005 | -0.25 |
| 0.5 | 0.8346648 | -0.01715567 |
| -3.75 | 0 | -0.6078227 |
| -1 | 0.3354835 | -0.25 |
| 0.25 | 0.5 | 0 |
| -1.25 | 0 | 2.75 |
| -4.25 | -1.5 | -0.05 |
| -0.25 | 0.0206387 | 0 |
| -4 | -0.7714933 | -1 |
| 0.25 | -3 | 0.6452724 |
| 0 | -0.5285517 | 1.185389 |
| 0 | 2.258247 | 2 |
| -2.5 | 1.305441 | 1.065889 |
| 0.25 | -0.5 |  |
| 0.75 | 0.04762795 |  |
| 0.5 | 0 |  |
| 2 | 0 |  |
| 0.5 | -1.420011 |  |
| -7.25 | 0 |  |
| -2.25 | -0.2702566 |  |
| 1 | 0.5037514 |  |
| 0.75 | -0.0620836 |  |
| 1.75 | 2 |  |
| -4.5 | 0.9954339 |  |
| 0 | 1.238236 |  |
| 0 | 0.7342986 |  |
| 0 | 1.347302 |  |
| 3 | -0.7777119 |  |
| -0.5 | 0.8345891 |  |
| -0.5 | 0.4219227 |  |
| -0.54 | 9.440085 |  |
| -0.33 | 0.5574735 |  |
| -1.5 | -0.8970138 |  |
| 3.49 | -1.75 |  |
| 0.5 | -3.523498 |  |
| -3.75 | 1 |  |
| 0.02 | -0.5030148 |  |
| 0.21 | -0.7592204 |  |
| 0.6 | 0 |  |
| 0.26 | 0 |  |
| 2.02 | -0.4977461 |  |
| 1.03 | 1 |  |
| 0.36 | -0.425 |  |
| 0.08 | 1 |  |
| 3.08 | -0.7721508 |  |
| -0.09 | -1 |  |
| 0.29 | 1.75 |  |
| -0.16 | 1.25 |  |
| -0.06 | 0.8483896 |  |
| 0.03 | 1 |  |
| 1.49 | 0 |  |
| -0.05 | 1.75 |  |
| 0.43 | 0.2 |  |
| 6.32 | -2.210822 |  |
| 0.21 | 0 |  |
| -0.75 | 0 |  |
| 3.24 | 0 |  |
| 0.59 | 0 |  |
| 0.53 | 0 |  |
| 0.01 | 0.0195652 |  |
| -12.24 | -0.75 |  |
| 0.19 | 0 |  |
| 0.05 | -0.5444596 |  |
| 1.48 | 1 |  |
| 0.09 | -0.25 |  |
| -0.62 | -1.405787 |  |
| -0.68 | 3 |  |
| 0.02 | -0.5 |  |
| 0.27 | 0.8285466 |  |
| -1.06 | 1.517796 |  |
| -0.03 | 0.7188067 |  |
| -12.25 | -0.5 |  |
| -0.65 | -1.565234 |  |
| 0.08 | -0.441816 |  |
| 0.04 | -1 |  |
| 0.25 | -0.3816137 |  |
| 1.16 | 0.09536401 |  |
| -2.91 | -0.1393185 |  |
| 0.72 | 0.8 |  |
| 1.38 | -0.6335037 |  |
| -0.56 | -10.5844 |  |
| -0.76 | 0.2795833 |  |
| -1.18 | 0 |  |
| 0.49 | 0.02754137 |  |
| 0 | -1.25 |  |
| -0.79 | 0.398995 |  |
| 0.33 | 0.2 |  |
| 0.54 | 1.294344 |  |
| 4.1 | -0.5 |  |
| -0.37 | 0.1128666 |  |
| -2.22 | 0.3801819 |  |
| -5.37 | 0.25 |  |
| -2.33 | 0 |  |
| -1 | 1 |  |
| 0.84 | 1.206253 |  |
| -0.33 | 0.6566545 |  |
| 2.86 | 1 |  |
| 3.18 | 0 |  |
| 0.57 | 1.25 |  |
| -0.19 | -1.910934 |  |
| -0.7 | 0.09817895 |  |
| -0.25 | 1 |  |
| 0.85 | 1 |  |
| -0.73 | 1 |  |
| 3.32 | -0.4577273 |  |
| 1.39 | 0.75 |  |
| -2.21 | 5 |  |
| -2.79 | 0.2388157 |  |
| -1.32 | 0.25 |  |
| -2.36 | 0.3399997 |  |
| 0.43 | 0.1272713 |  |
| 0 | -1 |  |
| 0.89 | -1.25 |  |
| -0.47 | 1.595259 |  |
| -0.79 | 0.8640217 |  |
| -0.29 | -0.25 |  |
| 2.16 | -0.495479 |  |
| 0.04 | 1.75 |  |
| 1.55 | 0.2046978 |  |
| 0.86 | -1.866457 |  |
| 3.75 | -2 |  |
| 1.67 | -1.885826 |  |
| 2.91 | -9.25 |  |
| 1.93 | -3 |  |
| 0.5 | -1.224453 |  |
| -1.06 | -1 |  |
| 0 | -4.780955 |  |
| 5.1 | 0.2226778 |  |
| -0.44 | -0.6565596 |  |
| 3.15 | -4.345987 |  |
| 0.39 | -1.080998 |  |
| 2 | -1.75 |  |
| 0.12 | 2.946262 |  |
| -1.49 | -1.270503 |  |
| -0.5 | -1.5 |  |
| 2.92 | -3.807237 |  |
| -0.1 | -2 |  |
| -1.5 | -2.80085 |  |
| 2.79 | -0.25 |  |
| -2.16 | -4.52 |  |
| -0.71 | -0.5811428 |  |
| -0.75 | -4 |  |
| -1.03 | -4.519986 |  |
| -0.23 | -1.03 |  |
| 0.5 | -1.75 |  |
| -2 | -1.75 |  |
| -1.15 | -2.75 |  |
| -3.05 | -3.5 |  |
| -3.11 | 2.42872 |  |
| -0.81 | 0 |  |
| -1.25 | -0.8489704 |  |
| 0.8 | -1.451316 |  |
| -1.98 | 0.9750066 |  |
| -2 | -3.75 |  |
| -0.67 | -0.75 |  |
| 1.25 | -1.821353 |  |
| -1.5 | -7.716267 |  |
| 0 | -0.5 |  |
| 0.35 | -3 |  |
| -2.7 | 0.4605296 |  |
| 0 | -2 |  |
| 2.27 | -0.9573958 |  |
| -1.64 | 0.25 |  |
| -4.25 | 1.641209 |  |
| 1.25 | 0.493661 |  |
| -3 | -2.204021 |  |
| 0 | 2.839436 |  |
| 0 | 1.45125 |  |
| -1 | 2.697797 |  |
| 0.76 | 0.75 |  |
| -10 | 3.5 |  |
| -0.63 | 1.18733 |  |
| -1.05 | 0.75 |  |
| -23.5 | 1 |  |
| -2.25 | 1.098535 |  |
| -8.14 | -0.5 |  |
| -2.75 | 3.51 |  |
| -0.32 | 5.017366 |  |
| -3 | 0.5 |  |
| -2.75 | -0.05 |  |
| -5.18 | 2.75 |  |
| -7.45 | 4.996281 |  |
| 3.49 | 1.25 |  |
| -0.5 | 2 |  |
| -2.57 | 3.276197 |  |
| -2.57 | -1 |  |
| -3.54 | 1.5 |  |
| -4.25 | 3 |  |
| -3.75 | 2.218373 |  |
| -10 | 0.5 |  |
| -20.5 | 0 |  |
| -4.62 | 4.25 |  |
| 1.11 | 3.5 |  |
| -7.75 | 6.722048 |  |
| -2.5 | 1.75 |  |
| -1.75 | 4.485174 |  |
| -1.75 | 0.8338989 |  |
| -3 | 3.933197 |  |
| -0.91 | 1.75 |  |
| -6.5 | 0.6741736 |  |
| -3.75 | 6.25 |  |
| -5.68 | 0.7740446 |  |
| -5.1 | 3.5 |  |
| -4.6 | 1.15225 |  |
| -1.68 | 2 |  |
| -2.55 |  |  |
| -9.6 |  |  |
| -0.48 |  |  |
| -5.5 |  |  |
| -1.5 |  |  |
| -0.25 |  |  |
| -3.25 |  |  |
| -2.17 |  |  |
| -6.5 |  |  |
| -7.75 |  |  |
| -1 |  |  |
| -2 |  |  |
| -6.5 |  |  |
| -4 |  |  |
| -4.64 |  |  |
| -0.69 |  |  |
| -3 |  |  |
| -3.08 |  |  |
| 4.43 |  |  |
| -1.25 |  |  |
| -1.75 |  |  |
| -1.5 |  |  |
| -8.7 |  |  |
| -10.22 |  |  |
| 3 |  |  |
| 1.4 |  |  |
| 17.24 |  |  |
| 1.1 |  |  |
| -1 |  |  |
| 0.85 |  |  |
| 4.75 |  |  |
| 1.73 |  |  |
| 4.85 |  |  |
| -1 |  |  |
| 6.33 |  |  |
| 8.95 |  |  |
| 1.76 |  |  |
| 0.5 |  |  |
| 6 |  |  |
| 1.5 |  |  |
| 1.07 |  |  |
| 3.1 |  |  |
| -0.24 |  |  |
| -0.87 |  |  |
| 0.5 |  |  |
| 14.5 |  |  |
| 0.5 |  |  |
| 2.12 |  |  |
| 2.5 |  |  |
| 0.86 |  |  |
| 0.41 |  |  |
| 1.95 |  |  |
| -0.25 |  |  |
| 0.36 |  |  |
| 0.22 |  |  |
| 0 |  |  |
| 1.75 |  |  |
| 1.75 |  |  |
| 0.43 |  |  |
| -4 |  |  |
| 4 |  |  |
| -1 |  |  |
| 1.5 |  |  |
| 2 |  |  |
| 3.85 |  |  |
| 2 |  |  |
| -1.5 |  |  |
| 0.68 |  |  |
| 5 |  |  |
| 7 |  |  |
| 2.77 |  |  |
| 13.69 |  |  |
| -0.39 |  |  |
| 2.69 |  |  |
| 4.46 |  |  |
| 1.92 |  |  |
| 11.43 |  |  |
| 1.33 |  |  |
| 1.5 |  |  |
| 3.56 |  |  |
| -0.73 |  |  |
| -1.5 |  |  |
| -7.75 |  |  |
| -12 |  |  |
| -4 |  |  |
| -8.75 |  |  |
| -8.19 |  |  |
| -3 |  |  |
| -5.36 |  |  |
| -7.92 |  |  |
| -4.55 |  |  |
| -0.75 |  |  |
| -1.84 |  |  |
| -9.32 |  |  |
| 1.25 |  |  |
| 1.25 |  |  |
| -4.19 |  |  |
| -3.5 |  |  |
| -6.57 |  |  |
| -8.75 |  |  |
| -3 |  |  |
| -3.25 |  |  |
| -12.54 |  |  |
| 2.9 |  |  |
| -0.8 |  |  |
| -2.69 |  |  |
| -1.29 |  |  |
| -3.42 |  |  |
| 1.25 |  |  |
| -0.43 |  |  |
| 0.75 |  |  |
| -1.5 |  |  |
| -2.03 |  |  |
| 3.36 |  |  |
| 1 |  |  |
| -4.17 |  |  |
| 4.6 |  |  |
| 6.2 |  |  |
| -2.25 |  |  |
| 0.95 |  |  |
| -1 |  |  |
| -3.5 |  |  |
| 2.57 |  |  |
| 0.19 |  |  |
| -8.43 |  |  |
| 6 |  |  |
| 6 |  |  |
| 2.75 |  |  |
| 1.58 |  |  |
| 0.84 |  |  |
| 6.75 |  |  |
| 2.92 |  |  |
| 3.25 |  |  |
| 3.77 |  |  |
| 1.52 |  |  |
| 7.46 |  |  |
| 3.12 |  |  |
| -0.48 |  |  |
| 7.55 |  |  |
| 9.25 |  |  |
| -0.02 |  |  |
| 4.75 |  |  |
| 4.25 |  |  |
| 0.2 |  |  |
| 6.75 |  |  |
| 4.5 |  |  |
| -1.38 |  |  |
| 0.21 |  |  |
| -1.15 |  |  |
| 0 |  |  |
| -3.59 |  |  |
| -1 |  |  |
| -0.36 |  |  |
| 0.13 |  |  |
| 0.29 |  |  |
| 0 |  |  |
| 0 |  |  |
| 0.24 |  |  |
| 1.09 |  |  |
| -0.41 |  |  |
| 0 |  |  |
| 0 |  |  |
| -0.5 |  |  |
| 1 |  |  |
| -0.06 |  |  |
| 0.67 |  |  |
| -6 |  |  |
| -0.25 |  |  |
| -0.3 |  |  |
| 1.25 |  |  |
| 0.27 |  |  |
| 1.64 |  |  |
| 1.94 |  |  |
| -0.36 |  |  |
| -0.3 |  |  |
| -3.93 |  |  |
| 0.7 |  |  |
| 0.6 |  |  |
| 0.65 |  |  |
| 0.21 |  |  |
| -1.09 |  |  |
| 2.2 |  |  |
| -1.07 |  |  |
| -0.54 |  |  |
| -0.05 |  |  |
| 2.31 |  |  |
| -0.25 |  |  |
| 0.17 |  |  |
| 0.27 |  |  |
| -0.34 |  |  |
| -0.25 |  |  |
| -1.13 |  |  |
| 0.25 |  |  |
| -0.95 |  |  |
| 1.81 |  |  |
| -0.25 |  |  |
| 0.75 |  |  |
| 0.85 |  |  |
| -4.25 |  |  |
| 1.21 |  |  |
| -0.14 |  |  |
| -1.04 |  |  |
| -0.3 |  |  |
| -0.25 |  |  |
| 0 |  |  |
| 0.09 |  |  |
| 0 |  |  |
| -0.75 |  |  |
| -1.83 |  |  |
| 0 |  |  |
| 0.35 |  |  |
| -0.5 |  |  |
| -1 |  |  |
| -0.08 |  |  |
| -0.24 |  |  |
| 1 |  |  |
| -0.95 |  |  |
| -1.47 |  |  |
| -0.24 |  |  |
| 0.19 |  |  |
| -0.5 |  |  |
| 0.5 |  |  |
| -1.25 |  |  |
| -0.5 |  |  |
| -1.42 |  |  |
| -0.05 |  |  |
| 3.45 |  |  |
| -0.9 |  |  |
| -2 |  |  |
| 1.5 |  |  |
| -0.71 |  |  |
| 0.94 |  |  |
| 6.5 |  |  |
| 0.46 |  |  |
| 0.58 |  |  |
| 0.08 |  |  |
| 0.83 |  |  |
| 0.16 |  |  |
| 5.73 |  |  |
| 0 |  |  |
| -1 |  |  |

**Statistical report**
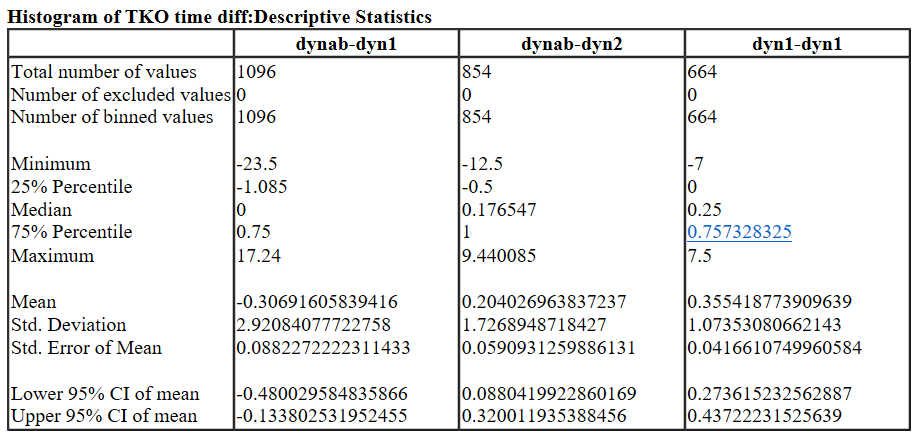
**:**
