# Supplementary material for: Uncoupling of dynamin polymerization and GTPase activity revealed by the conformation-specific nanobody dynab
Source: eLife. 2017 Oct 12;6:e25197. doi: 10.7554/eLife.25197 (PMC5658065; doi:10.7554/eLife.25197)
Supplement: Figure 4—source data 5. [file elife-25197-fig4-data5.docx]

**Figure 4-Source Data 5 (panel C)**

Cumulative probability of time difference between *t*(dynab)-*t*(dyn1-2) and DYNctrl in HeLa cells, and statistical report

| *t*(dynab)-*t*(dyn1) | *t*(dynab)-*t*(dyn2) | *t*(dyn1_EGFP)-*t*(dyn1_mCherry) |
| --- | --- | --- |
| 0.5 | -2 | 0.25 |
| 0.5 | -1.25 | -1 |
| 1 | -0.5 | 0 |
| 2.25 | 0.25 | 0.75 |
| 2.25 | 2 | -0.5 |
| -3.75 | -2 | 2.25 |
| -0.75 | -1.75 | 0 |
| 1.75 | 2.75 | 0.75 |
| 0 | 0 | 0.25 |
| -6.75 | 0 | -0.25 |
| -5.75 | 1.5 | 0.5 |
| 1.5 | 1 | 0.75 |
| 0 | -0.5 | -0.5 |
| -7.5 | 0.5 | 2 |
| -0.5 | -0.5 | 0.25 |
| -0.25 | 0.25 | 0.5 |
| -8.5 | 0.25 | -2 |
| 0 | 0 | 0 |
| 0 | 0.5 | 1.75 |
| 1.5 | -1.25 | 1.75 |
| -0.5 | 1.25 | 1.25 |
| -1.5 | -0.25 | 0.75 |
| 2.25 | 0 | -0.75 |
| -0.25 | 1.25 | 0.25 |
| 0 | 0.25 | -0.5 |
| -1.25 | 0.25 | 0 |
| 0.5 | -0.25 | 1 |
| -1.25 | 0.5 | -5 |
| 0.25 | -1 | -0.75 |
| -0.25 | -3.75 | -0.25 |
| 0.25 | 0.75 | 0 |
| -0.25 | 3 | 0.75 |
| 0.75 | 2 | 1 |
| -1.75 | 0.5 | 0 |
| 1.25 | 0.75 | -2.75 |
| 0 | 0 | 0.25 |
| 1.75 | -2 | 0.5 |
| 2.5 | 0.75 | 0.5 |
| -0.25 | -0.75 | -1.5 |
| 0.5 | 4 | 2.75 |
| -1.75 | 2.25 | 0 |
| -0.5 | 0.75 | 0.25 |
| 1.25 | 0.5 | 0.25 |
| 2 | 0.75 | 1 |
| 0 | -1.75 | -1 |
| 1 | -1.25 | -0.25 |
| 1.25 | -0.25 | 0.75 |
| 0.25 | 0.5 | -0.5 |
| -0.5 | -0.5 | 2 |
| -0.25 | -1.75 | 0.5 |
| 0.25 | -0.75 | -0.5 |
| -0.25 | 1 | -1.25 |
| 0.25 | 2 | 0.75 |
| 0.75 | -0.5 | -2.25 |
| 0 | -2 | 0 |
| -1.5 | 0 | 0 |
| 0.25 | -3.25 | 0 |
| -1.75 | -0.25 | 1 |
| 0 | 0.75 | 0.75 |
| -1.25 | 0.5 | -1 |
| -0.25 | 1.75 | 0.5 |
| 0 | 0 | -0.75 |
| 0.25 | 0.75 | 0.5 |
| 0.25 | -0.75 | 0.25 |
| -1.5 | -0.5 | -0.25 |
| -0.5 | 1 | 1.25 |
| 0.25 | -10 | 4 |
| 4.75 | 0.25 | 0.5 |
| -1.25 | 0 | 0 |
| 0 | -2 | 0 |
| -4.5 | 0 | 0 |
| -1 | 0 | -1.25 |
| 0 | -0.75 | -1.5 |
| -2.5 | -0.25 | 0.75 |
| 0.25 | -0.25 | 0.75 |
| 2 | -1.25 | 0.25 |
| 0.5 | 0.5 | 2.25 |
| 3 | -0.25 | 1.25 |
| 0.25 | 1.5 | 1.5 |
| -2.75 | -0.75 | 0 |
| 1.75 | -0.75 | 0.5 |
| 0.5 | 0.25 | 0.5 |
| 0.5 | 0.75 | -0.5 |
| -0.5 | -0.25 | 0.75 |
| -1.25 | 0.75 | 0.25 |
| 0 | -2.25 | 3.5 |
| 0.25 | -0.75 | 2.75 |
| -0.75 | -1.75 | 1.25 |
| 0.75 | 0.5 | 0 |
| 1.75 | 0.75 | -3.5 |
| 1 | 0.5 | 0 |
| 3.5 | -0.25 | 2 |
| -4 | -0.25 | 1.25 |
| -1.25 | -0.25 | 0.5 |
| -1.75 | 0 | 0 |
| -1 | 0 | -0.25 |
| 0.25 | 0.25 | 0.5 |
| 0.75 | 1.5 | 1.25 |
| -1.25 | 0.5 | 1.5 |
| 0.75 | -2.25 | 0.75 |
| -0.5 | 0 | 0.25 |
| 2.25 | -1.75 | -0.25 |
| -2.5 | -0.25 | 0 |
| -2 | -0.25 | -0.25 |
| 0 | -0.25 | -0.25 |
| -0.25 | 0 | 0 |
| -0.5 | 0.25 | 1 |
| -0.25 | -0.75 | 0.75 |
| 0.75 | -1.5 | 0 |
| -0.5 | 0.5 | 1.75 |
| -1.25 | -1 | 0 |
| 2.25 | -1.75 | 0.25 |
| -1.75 | -1.5 | 0 |
| 0.5 | 1.25 | 1.75 |
| 1.5 | 1 | 0.25 |
| -1 | 0.75 | 0.25 |
| 0.25 | 0.5 | -1 |
| 1 | -0.5 | 0 |
| 0.5 | -0.25 | -0.25 |
| 0 | 0 | 1.5 |
| -3.25 | 0.25 | -2.25 |
| -1.25 | 2 | -0.5 |
| 1.25 | 0.75 | -0.25 |
| 0.25 | 1.75 | 0 |
| 1.25 | -1 | 1.75 |
| 0 | -0.5 | 0 |
| 1.25 | 0.5 | -1 |
| 0 | -1.25 | 0.25 |
| -0.25 | -1.75 | 0.25 |
| 2.75 | -0.25 | 0.25 |
| -1.75 | -0.75 | 1.25 |
| -1.25 | -0.5 | 0 |
| 0 | 1.5 | 0 |
| -1.25 | 0 | 2 |
| 0 | -0.5 | -0.5 |
| 1 | 0.25 | -0.5 |
| 0.5 | 0.5 | -0.5 |
| -0.75 | -0.75 | -0.25 |
| -2.25 | 0 | 2 |
| 0.75 | 1.25 | 1.25 |
| 0.25 | 0 | 0 |
| -0.75 | -0.25 | 0 |
| 0.75 | 0 | -3.25 |
| 1.5 | 3 | -1 |
| -0.25 | 1.75 | 0.75 |
| 0.25 | -0.75 | -0.25 |
| 0.75 | -1.5 | -1.25 |
| -0.5 | 0.25 | -0.25 |
| 0.5 | -0.25 | 1.5 |
| 0.25 | 0.25 | 0.5 |
| 1 | -1.5 | -0.75 |
| 2.5 | 0 | 0.5 |
| -0.25 | -0.5 | 2 |
| 2.5 | -1.75 | 0 |
| 0 | 0.5 | 1.25 |
| 4.75 | 0 | -0.75 |
| 3 | -0.25 | 0 |
| -4.25 | -0.25 | 0.5 |
| -0.75 | 0.75 | 0.75 |
| 3.75 | 0 | -0.75 |
| -2.5 | -1.25 | -5.75 |
| 0.25 | 0.5 | -0.25 |
| 0.25 | 0.5 | -0.25 |
| 2 | 0.75 | 0.75 |
| -0.25 | 0.75 | 0.25 |
| -7.75 | -0.75 | 0.75 |
| 0.75 | 1.5 | 0 |
| -2.5 | -1.75 | 1 |
| 0.5 | -0.25 | 0.5 |
| 1.5 | -1.75 | 1 |
| -1.5 | -1.5 | 3.25 |
| -0.5 | 1.5 | 1.25 |
| 0 | 0 | 2.5 |
| -0.25 | -0.25 | 0.25 |
| 1 | 1.5 | 0.75 |
| 1.75 | 0 | 0 |
| -0.25 | -0.25 | -0.25 |
| 0.25 | -1 | 0.5 |
| -0.25 | 0.75 | 0 |
| -2.5 | -0.75 | 1.75 |
| 1.5 | 0.75 | -0.25 |
| 2.5 | -0.75 | 0.25 |
| 0.25 | -0.25 | 0.75 |
| -1 | -0.25 | 0.75 |
| -0.25 | -1.75 | 3 |
| 0.25 | -1.25 | -0.5 |
| 0.5 | -0.25 | 0 |
| 1.75 | 1.25 | -1.25 |
| 1.75 | 0.25 | 2 |
| -0.25 | -0.5 | -0.75 |
| 0.5 | 0 | 1.25 |
| 0.25 | -0.25 | 0.5 |
| 1.5 | -4 | -0.5 |
| 0 | -1 | 1.75 |
| -1.75 | 0.25 | 1.5 |
| 1 | -0.25 | -1.25 |
| -3.5 | -1.75 | -1 |
| 1.5 | -1.25 | -0.5 |
| 0.5 | 1.75 | 0.5 |
| 0 | 1 | 1.25 |
| -0.5 | -0.25 | 1.75 |
| 1.5 | 0 | 1 |
| -2 | 5 | -0.5 |
| -1.5 | -0.25 | 1.5 |
| -0.75 | 2.5 | 0.5 |
| -0.75 | -0.5 | 0.5 |
| 0 | 0.75 | 0.25 |
| -0.25 | 0.75 | 0.25 |
| -1 | 0.5 | -0.25 |
| 0 | 1 | 0.75 |
| -0.75 | -0.25 | 3.75 |
| -0.5 | 1 | -2.25 |
| -0.75 | 0.25 | 2 |
| -0.25 | -0.5 | 0 |
| 1 | 0.25 | 1.25 |
| -3.5 | -0.75 | 0.5 |
| 1.25 | 2.5 | 1.75 |
| 2 | 0.75 | -1.5 |
| 2 | -0.75 | -0.25 |
| -0.5 | -5.5 | 1.5 |
| 2.25 | 0.75 | -1.75 |
| 3 | -0.25 | -0.25 |
| -1 | 1.25 | 1.25 |
| 1.25 | 0.25 | 0.5 |
| 0 | 2 | 1.75 |
| -2.75 | -1.75 | 0 |
| -0.75 | -0.5 | -0.5 |
| 0 | 2.5 | -0.5 |
| 0.75 | 0.75 | 0.75 |
| 0.25 | -0.5 | 1 |
| 1 | -1.5 | 1.75 |
| -1.25 | -2 | 0 |
| 0.25 | -0.25 | -4.5 |
| 0.25 | 2 | 0.5 |
| -0.25 | 1.75 | -1 |
| -0.5 | 0.25 | -1 |
| 1 | -0.5 | 1.5 |
| -1.5 | 1.25 | 0.5 |
| 0.75 | 0.25 | 2.5 |
| 0.5 | 0.25 | -0.75 |
| 0.5 | 0.25 | 1.75 |
| 0.25 | -0.5 | 1 |
| 0.75 | -1 | -0.25 |
| 0.5 | 0 | 0.25 |
| 0.5 | -0.75 | -1 |
| -1.5 | 0.25 | 0 |
| 0 | 2.5 | 0 |
| -0.75 | 0 | 1.5 |
| 0.5 | 0 | 0.25 |
| -0.25 | 0 | 0 |
| 0.75 | -0.75 | 0.25 |
| 2.5 | 0 | 0 |
| 1 | 4 | 0.25 |
| -0.25 | -0.5 | -0.25 |
| -1.25 | 1.75 | 0.5 |
| -2.75 | 2 | -0.25 |
| -1 | -0.25 | -0.5 |
| -2.75 | 0.75 | 0.25 |
| 0.25 | 1.75 | 0.75 |
| 1.25 | 0.75 | -1 |
| 0.75 | -4.25 | 0.75 |
| 1.5 | 1.25 | 0.75 |
| -1.75 | 1 | 1.5 |
| -0.25 | 1.75 | 0.75 |
| 0 | -1 | 1 |
| -0.25 | 0.75 | -0.25 |
| -0.75 | 0 | 0.5 |
| -2.25 | 0.5 | 0 |
| 0.25 | -1.25 | 0.75 |
| 0.75 | 0.5 | 0.25 |
| 0 | 3.75 | 2.25 |
| -0.25 | 0.75 | 0 |
| -4.25 | 1.75 | -0.9469412 |
| -0.5 | 0 | 0.4186984 |
| -1.5 | 0.75 | 2.75 |
| -0.25 | 0.25 | 0.4242972 |
| 0 | 2.5 | 1.238191 |
| 0 | -1.75 | 0.25 |
| 0.5 | -0.25 | -0.25 |
| -0.5 | 0.25 | 1 |
| 0.5 | 0 | -0.1482364 |
| 0 | 1.25 | -1 |
| -2.25 | 0 | -0.4160672 |
| 1 | 0 | -0.5625665 |
| 0.75 | -0.25 | -0.1756906 |
| 0 | -0.25 | 0.75 |
| 3.5 | 2.25 | 0.8439016 |
| -0.5 | -2 | -0.1148255 |
| 2.25 | 0.25 | -0.9814968 |
| -0.25 | -0.5 | -0.5903553 |
| -0.25 | 0.5 | -0.6952534 |
| 4.25 | -0.25 | 0.2124074 |
| 1.25 | 0 | -0.06270017 |
| 0.75 | -1 | 0.1322158 |
| -0.5 | -2 | -0.2796964 |
| -1 | -0.5280429 | 0 |
| -1.25 | -3 | 0.1359872 |
| 0.25 | 0 | 0.1103043 |
| 0.25 | 0 | 1 |
| -1.5 | 0 | 0 |
| -0.5 | -0.1246696 | -0.75 |
| -1.5 | 1.133243 | 0 |
| 1.25 | -0.08929839 | -0.4752611 |
| 0.25 | -0.141598 | 3.216172 |
| 0.25 | -0.2825057 | 1 |
| 2.5 | 0.8139903 | -0.4643657 |
| -0.5 | -1 | 1.966649 |
| -1.75 | 0.8210926 | 0.7468432 |
| -0.75 | 0.5010045 | 1.26934 |
| -2 | -2 | -1 |
| 1.75 | -0.25 | -0.3998643 |
| -1 | 0.8866546 | -1.411075 |
| 0.75 | -0.4453034 | 1.555849 |
| 0 | -0.5585497 | 0.6425455 |
| 0 | 0 | -0.6995084 |
| -1.75 | 0.3190508 | 0 |
| -1.75 | 0 | -0.6307546 |
| 0.75 | 1 | -0.25 |
| 6.75 | -2.25 | 2 |
| 0 | -0.05431557 | -0.4612089 |
| 0.5 | 1.719828 | 1.205488 |
| -0.25 | -1.447881 | 0.683511 |
| -0.5 | 0.8591608 | 0 |
| -0.25 | -0.1485188 | 0.7276642 |
| 0.25 | 1.974071 | 0 |
| 0.8360894 | -0.75 | 0.02982008 |
| -0.1235702 | 0.75 | 0.5390334 |
| -2.656009 | 0.3391811 | -1.33955 |
| -0.3113059 | 0.8 | -0.08660022 |
| -0.6269232 | -1.154836 | -3.192336 |
| -1 | 0.1104519 | -1.558551 |
| 0.9780719 | -1.71449 | -2 |
| 0.02309282 | -1.241147 | -0.6932384 |
| -0.25 | -0.4454092 | 2 |
| 0.079547 | 2 | -0.2686132 |
| 0.1153009 | -0.75 | 1 |
| -0.5 | -0.7965335 | 0 |
| -5.026948 | 0.2933303 | 1 |
| -0.03275595 | 0.2789711 | 1.473419 |
| -1.641994 | 0.8687676 | 0 |
| -2.5 | -0.8366031 | 0.924335 |
| -0.4397953 | -0.1559832 | -0.833994 |
| 2 | -0.75 | 1.047671 |
| 0.4460564 | 2.636426 | 1 |
| 1.559006 | -2.936028 | 0.3895804 |
| -0.7339799 | -0.75 | 1.179221 |
| -1.643652 | 0.04618725 | 1.567 |
| -0.75 | 1.25 | 0.6892517 |
| 1.696902 | 0 | 0.3824363 |
| -0.484363 | -0.7901759 | 1 |
| -0.2978664 | -1.05 | 0 |
| -0.01897029 | 2 | 0.3597724 |
| 0 | 2.134867 | 0.453657 |
| 1.570734 | -0.3832651 | -1.106856 |
| 3.581526 | 0.25 | -0.5977443 |
| -0.1788812 | 0.3926748 | 0 |
| 0.7969323 | 0.5924667 | 0.9396979 |
| -0.7258335 | 0.01299557 | 0.5 |
| 0.1769148 | 0.3435563 | 1.25 |
| -0.5600298 | 0.04548873 | -2 |
| 0.5070611 | -0.3231098 | -0.4051405 |
| 0.7141271 | 0.4498988 | 1 |
| -0.1557675 | -0.05 | -0.949933 |
| 0.05725932 | 1.268165 | -1 |
| -0.7646236 | 1.132951 | 0.7335416 |
| -1.656033 | 0.449311 | -0.5986105 |
| 1.114494 | -0.2481092 | 1 |
| 2.3401 | 0.2195091 | 0.5055271 |
| -0.7056565 | 0.9420894 | -0.1816456 |
| -3.379875 | 0.7336976 | 0.2916716 |
| -1.270128 | 0.5641618 | -1.988825 |
| 0.957177 | 2.5 | 0.1705952 |
| -1.751245 | -0.3680083 | 0.25 |
| 0 | 1.714996 | -2.25 |
| 0.7300277 | -0.75 | 0.9451242 |
| -0.7150905 | -0.7395784 | -0.7807985 |
| 0.06854975 | -0.3095568 | 0 |
| -3.469602 | -0.4257194 | -0.4545903 |
| 0.4398472 | 0.646526 | 1.826462 |
| 0.2084715 | -0.01012448 | -0.2474311 |
| 1.289841 | -0.9949834 | 0.75 |
| -2.428375 | -1.789742 | -0.3197325 |
| -1 | -0.1035266 | 0.6325804 |
| 0.9935456 | 0.1925771 | 0.3053186 |
| -0.1428033 | -0.00253235 | 0.07670777 |
| 1.087534 | -1 | 1.043279 |
| -0.2969619 | 0.4861811 | -0.7846581 |
| -0.6504064 | 0.6454504 | -0.09550946 |
| 0.75 | -0.09056315 | -0.1596048 |
| 1 | 0.4278059 | 1.5 |
| -1 | -0.04790499 | 2.116178 |
| 1.419207 | -0.2574193 | -0.25 |
| -0.5129811 | 0.236074 | 0.06445256 |
| -1 | -0.25 | 0.5387696 |
| 0 | 0.583168 | 1.197315 |
| -0.1753722 | -0.07672637 | -0.911956 |
| -3 | -0.75 | 0.2260027 |
| -1.51504 | 1.519267 | -3.732755 |
| 0 | 0.1112081 | 0.5 |
| -1.5 | 2.509602 | 0.1043316 |
| -0.25 | 0.3473413 | 0.7329912 |
| -1 | 0.7597751 | 3.770587 |
| -1 | -0.3797675 | 0 |
| -2.25 | 1.277043 | -0.03072366 |
| 0.0188316 | -0.25 | -0.75 |
| 0.25 | -0.05332903 | -1 |
| -0.3534162 | 1.469439 | -1.777485 |
| 0.3181112 | 0.350964 | 0.09783931 |
| 0.75 | 0.25 | 0.75 |
| 0.25 | -1 | -0.6575068 |
| -0.6784023 | 0.3684516 | 0.75 |
| 1.605918 | 1.556079 | -0.1473022 |
| 1.304364 | 0.00993815 | 0.25 |
| -1.93411 | -1.203221 | -0.770375 |
| 1.25 | 0.5075265 | -1.149723 |
| -1 | 0.6682526 | 0.1347755 |
| -1.552822 | -0.2389499 | 0 |
| -2.397422 | 0.2424172 | 1.034079 |
| -2 | 1 | 1.004303 |
| 0.3432463 | 0.3388766 | 0.7249423 |
| 0 | -0.6312497 | -0.1504009 |
| 0 | 1.229417 | 0.6205906 |
| 0.1905007 | 1.5 | -1.320455 |
| 4.994796 | -3.358876 | 0.8710755 |
| -1.5 | -1 | 0 |
| 0 | -0.75 | 0.4330769 |
| -0.5657553 | 0.5 | 5.5 |
| -0.2816705 | 8.975632 | -0.4244059 |
| 0.1865784 | -2 | -1 |
| 0.6741956 | -6.013974 | -0.25 |
| 2 | -2.367657 | -1 |
| -1.188745 | -1.231116 | 0 |
| 2.145305 | -1.039342 | -5.247551 |
| 2.540705 | -5.501458 | -0.6329038 |
| 0.5007477 | -9.184391 | 0.1821435 |
| 0.459801 | -0.3653936 | 0.6112998 |
| 1.064049 | 7.146474 | -0.75 |
| -0.7143058 | 2.25 | -0.1690204 |
| -1 | 5.073922 | 0.3036085 |
| 2.560764 | 3.92033 | 0.1202123 |
| -0.2395445 | 3.467842 | -0.09827725 |
| -0.8653451 | 3.5 | 0.5 |
| 0.75 | 1.294565 | -0.5684892 |
| 0.8239507 | 1.25 | 0.75 |
| -2 | 1.25 | 1.442229 |
| -1.565286 | 2.5 | -0.25 |
| -1.373497 | 2.083723 | 0 |
| -7.12627 | 0.09872983 | 0 |
| 0.1414048 | -0.25 | -0.25 |
| 1.25 | 3 | 0.001134928 |
| 0.7510206 | 0.7597019 | 0.5017366 |
| -3.75 | -2.267289 | 0 |
| 0.75 | -1.5 | 0 |
| 0.9997145 | -0.6972148 | -0.5 |
| 2.344941 | -9.18454 | 0.5 |
| -3 | -0.22214 | 1.76739 |
| 0.5537834 | 0.4001119 | 0.25 |
| -7.25 | 1 | -1.213182 |
| -2.916646 | -0.6549163 | -1 |
| -1.637028 | 0.2445379 | 3.25 |
| -0.4445459 | 2.5 | 1.988231 |
| -2.394618 | -0.6492894 | -0.25 |
| -1.837185 | 0.5610458 | 0.25 |
| -1.502119 | 1.5 | 0 |
| -3.231429 | -1.694513 | 1 |
| -0.3556348 | 0 | 0.4530073 |
| -0.6016373 | 3.25 |  |
| -3 | 1 |  |
| -5 | 0.3743982 |  |
| -2.400603 | -1.431725 |  |
| -1.084088 | -1.072619 |  |
| -0.5 | 2.25 |  |
| -5 | -0.2182303 |  |
| -3.908808 | 0.84 |  |
| -3.265014 | 1 |  |
| -4.75 | 1.323199 |  |
| -4.444744 | 0.02632413 |  |
| -2 | 0 |  |
| -2.021402 | 0.039962 |  |
| -2.837033 | 0 |  |
| -2.322066 | -0.6292383 |  |
| -1.983472 | -0.8323342 |  |
| -1 | 0.5875504 |  |
| 3.2 | -0.7697836 |  |
| 3.5 | -0.01016268 |  |
| 2 | -0.170479 |  |
| 1 | -1.129023 |  |
| 3.646993 | -2.26702 |  |
| 2.75 | -0.192729 |  |
| 2.181647 | -0.25 |  |
| 0.25 | -1.089502 |  |
| 0.3736773 | -0.2776935 |  |
| 0 | -0.3715804 |  |
| 2.250304 | 0.08889091 |  |
| 1.5 | -0.4558035 |  |
| 0.25 | -0.5 |  |
| -1 | 0.05268155 |  |
| 1 | -1.836043 |  |
| 1 | 1.510718 |  |
| 3.4 | 0.5 |  |
| 0.09150199 | 0.75 |  |
| 1 | -1.5 |  |
| 0.75 | 1.8 |  |
| 0.8381577 | -2 |  |
| 0.75 | -0.04279896 |  |
| 1.541581 | 0 |  |
| 0.2800472 | 0.25 |  |
| -0.1627458 | -0.25 |  |
| 0.25 | -0.25 |  |
| 0.25 | 0 |  |
| 4 | 0 |  |
|  | 0.1046743 |  |

**Statistical report:
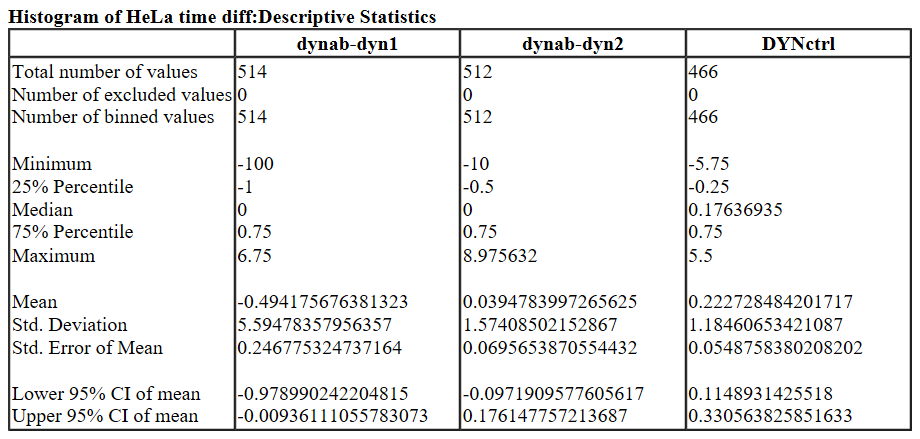
**
