# Supplementary material for: Uncoupling of dynamin polymerization and GTPase activity revealed by the conformation-specific nanobody dynab
Source: eLife. 2017 Oct 12;6:e25197. doi: 10.7554/eLife.25197 (PMC5658065; doi:10.7554/eLife.25197)
Supplement: Figure 4—source data 6. [file elife-25197-fig4-data6.docx]

**Figure 4-Source Data 6 (panel C)**

Relative frequency *Δt* values; *Δt* distribution expressed in percentage of relative dynamin duration.

**TKO cells**

|  | *t*(dynab)-*t*(dyn1) | *t*(dynab)-*t*(dyn2) | *t*(dyn1_EGFP)-*t*(dyn1_mCherry) |
| --- | --- | --- | --- |
| Over -100% | 2.697841727 | 0.588928151 | 0.306748466 |
| -95-100% | 0.1798561 | 0 | 0 |
| -85-95% | 3.327338 | 0.8244994 | 0.4601227 |
| -75-85% | 0.8093525 | 0.3533569 | 0 |
| -65-75% | 1.348921 | 0.4711425 | 0 |
| -55-65% | 1.708633 | 0.2355713 | 0.1533742 |
| -45-55% | 1.528777 | 0.2355713 | 0.1533742 |
| -35-45% | 2.697842 | 1.413428 | 0.3067485 |
| -25-35% | 4.406475 | 1.88457 | 1.07362 |
| -15-25% | 6.92446 | 5.182568 | 2.760736 |
| -5-15% | 15.55755 | 13.54535 | 10.27607 |
| ±5% | 26.43885 | 41.22497 | 40.18405 |
| -5-15% | 16.54676 | 17.43227 | 30.21472 |
| -15-25% | 6.47482 | 7.773851 | 8.435583 |
| -25-35% | 3.057554 | 3.180212 | 4.141104 |
| -35-45% | 1.888489 | 1.648999 | 0.9202454 |
| -45-55% | 1.618705 | 1.88457 | 0.4601227 |
| -55-65% | 0.8992806 | 0.4711425 | 0.1533742 |
| -65-75% | 0.7194245 | 0.8244994 | 0.3067485 |
| -75-85% | 0.7194245 | 0.5889282 | 0 |
| -85-95% | 0.4496403 | 0 | 0.1533742 |
| -95-100% | 0 | 0.2355713 | 0.1533742 |
| Over 100% | 1.528776978 | 1.413427562 | 1.380368098 |

**HeLa cells**

|  | *t*(dynab)-*t*(dyn1) | *t*(dynab)-*t*(dyn2) | *t*(dyn1_EGFP)-*t*(dyn1_mCherry) |
| --- | --- | --- | --- |
| Over -100% | 3.402646503 | 0.980392157 | 0.642398287 |
| -95-100% | 0.3780718 | 0 | 0 |
| -85-95% | 5.293006 | 1.372549 | 0.6423983 |
| -75-85% | 0.9451796 | 0.1960784 | 0 |
| -65-75% | 1.512287 | 0.5882353 | 0.2141328 |
| -55-65% | 1.701323 | 0.5882353 | 0.2141328 |
| -45-55% | 2.268431 | 1.960784 | 0.4282655 |
| 35-45% | 3.591682 | 2.745098 | 0.2141328 |
| -25-35% | 5.293006 | 3.529412 | 1.284797 |
| -15-25% | 5.10397 | 6.27451 | 3.640257 |
| -5-15% | 12.28733 | 14.31373 | 14.13276 |
| ±5% | 18.90359 | 33.72549 | 40.04283 |
| -5-15% | 15.87902 | 16.66667 | 25.69593 |
| -15-25% | 8.884688 | 8.039216 | 7.280514 |
| -25-35% | 3.969754 | 2.745098 | 2.35546 |
| -35-45% | 2.835539 | 2.745098 | 1.713062 |
| -45-55% | 1.701323 | 1.568628 | 0 |
| -55-65% | 1.512287 | 0.9803922 | 0.6423983 |
| -65-75% | 0.5671077 | 0.1960784 | 0 |
| -75-85% | 1.701323 | 0.1960784 | 0.6423983 |
| -85-95% | 0 | 0.3921569 | 0.2141328 |
| -95-100% | 0 | 0.1960784 | 0 |
| Over 100% | 2.268431002 | 1.37254902 | 0.642398287 |
