# Supplementary material for: Uncoupling of dynamin polymerization and GTPase activity revealed by the conformation-specific nanobody dynab
Source: eLife. 2017 Oct 12;6:e25197. doi: 10.7554/eLife.25197 (PMC5658065; doi:10.7554/eLife.25197)
Supplement: Figure 4—source data 7. [file elife-25197-fig4-data7.docx]

**Figure 4-Source Data 7 (panel D&F)**

Maximum intensity values of dynab peaks vs dynamin peaks

**TKO cells**

| max dyn1 | max dynab |  | max dyn2 | max dynab |  | max dyn1_cherry | max dyn1_EGFP |
| --- | --- | --- | --- | --- | --- | --- | --- |
| 9918.06 | 8271.3 |  | 8655.66 | 5849.68 |  | 2402.35 | 7580.51 |
| 18987.53 | 16503.29 |  | 3790.98 | 2066.98 |  | 5485.99 | 28625.24 |
| 8098.92 | 4944.91 |  | 4473.02 | 3246.06 |  | 3081.66 | 18898.08 |
| 5224.6 | 2861.74 |  | 5039.38 | 3807 |  | 4758.59 | 6143.75 |
| 15929.31 | 13158.43 |  | 5153.2 | 6197.35 |  | 8927.96 | 12370.16 |
| 11312.99 | 11727.33 |  | 2606.35 | 2086.41 |  | 8522.93 | 7765.6 |
| 9503.8 | 4912.08 |  | 5589.2 | 5368.3 |  | 2036.41 | 11814.53 |
| 21124.99 | 19100.38 |  | 3770.99 | 3453.77 |  | 1794.82 | 5222.25 |
| 18027.6 | 11974.03 |  | 1629.6 | 3430.82 |  | 2784.8 | 10223.92 |
| 4649.84 | 5567.21 |  | 13387.78 | 7642.89 |  | 3134.18 | 8440.05 |
| 20762.71 | 11707.43 |  | 2640.79 | 2950.17 |  | 9369.55 | 13726.08 |
| 8357.481 | 8966.47 |  | 2897.77 | 2331.98 |  | 2773.8 | 10886.23 |
| 20192.9 | 13472.64 |  | 3835.39 | 3631.08 |  | 4112.48 | 20611.92 |
| 9189.74 | 7926.77 |  | 6038.56 | 4906.74 |  | 7352.29 | 15567.35 |
| 4457.87 | 4766.87 |  | 12151.37 | 6769.04 |  | 5487.32 | 24293.49 |
| 23099.06 | 10996.73 |  | 964.41 | 1850.19 |  | 5441.53 | 11583.93 |
| 7856.71 | 14452.48 |  | 1947.81 | 2612.61 |  | 3362.49 | 9974.17 |
| 2381.93 | 2793.5 |  | 14099.13 | 6526.61 |  | 6256.66 | 20829.14 |
| 2379.06 | 3267.48 |  | 7188.4 | 4156.27 |  | 2591.75 | 5809.76 |
| 17835.78 | 11713.74 |  | 3575 | 2504.75 |  | 9637.57 | 13406.62 |
| 5107.41 | 6647.28 |  | 5837.9 | 4002.08 |  | 6159.72 | 12066.74 |
| 4608.63 | 9320.43 |  | 10132.81 | 6172.17 |  | 3322.06 | 13321.4 |
| 8886.37 | 8509.84 |  | 2410.69 | 1579.15 |  | 5661.14 | 10356.93 |
| 13879.79 | 10791 |  | 6514.39 | 2599.37 |  | 9707.231 | 25054.66 |
| 15293.4 | 10104.95 |  | 10846.21 | 6569.18 |  | 3626.22 | 8084.14 |
| 5431.54 | 4923.54 |  | 5328.85 | 3510.77 |  | 2483.81 | 9308 |
| 22009.1 | 11503 |  | 11990.45 | 6253.53 |  | 4073.61 | 13040.51 |
| 11241.18 | 8458.481 |  | 7858.1 | 3772.54 |  | 3349.24 | 7710.72 |
| 2503.02 | 3323.45 |  | 8515.44 | 5941.7 |  | 5638.43 | 11880.52 |
| 13035.01 | 8941.28 |  | 2940.08 | 1894.83 |  | 5757.43 | 9719.94 |
| 9571.39 | 12202.27 |  | 10735.93 | 6042.85 |  | 5142.16 | 8535.69 |
| 7922.68 | 5492.96 |  | 2886.18 | 2679.93 |  | 3680.73 | 10584.87 |
| 21123.01 | 11438.55 |  | 3488.05 | 3058.57 |  | 2220.54 | 14194.51 |
| 10719.52 | 8146.11 |  | 4325.89 | 2576.06 |  | 3882.68 | 8047.44 |
| 17134.7 | 13727.59 |  | 5645.48 | 4587.62 |  | 2480.4 | 7098.51 |
| 12262.41 | 11180.92 |  | 5793.92 | 4181.82 |  | 5979.01 | 9586.33 |
| 9623.69 | 10609.29 |  | 12475.12 | 7307.26 |  | 2621.05 | 7420.6 |
| 6988.47 | 3896.87 |  | 9599.85 | 7555.53 |  | 1623.32 | 7194.71 |
| 7699.89 | 6543.53 |  | 2218.22 | 3096.76 |  | 5665.84 | 9638.17 |
| 5425.09 | 4419 |  | 5459.38 | 2929.8 |  | 9519.14 | 14563.13 |
| 11218.06 | 8023.41 |  | 5400.18 | 5566.27 |  | 3994.96 | 19326.15 |
| 6742.59 | 8699.49 |  | 7704.69 | 6236.13 |  | 1486.81 | 6351.87 |
| 13796.37 | 7782.02 |  | 8583.62 | 6904.09 |  | 2969.07 | 20877.1 |
| 11973.06 | 10741.72 |  | 2605.24 | 2796.19 |  | 3496.64 | 14250.09 |
| 6610.18 | 3763.15 |  | 20248.98 | 9140.56 |  | 2065.51 | 12721.68 |
| 1263.12 | 1577.96 |  | 8519.34 | 4532.66 |  | 2738.02 | 5663.34 |
| 2135.44 | 2667.85 |  | 4099.63 | 3844.97 |  | 3939.44 | 14500.3 |
| 4582.85 | 3538.78 |  | 4316.99 | 2565.88 |  | 2932.35 | 14389.35 |
| 10855.11 | 8793.34 |  | 4822.4 | 3781.36 |  | 2675.16 | 7756.72 |
| 4602.64 | 2722.4 |  | 3795.1 | 2472.59 |  | 3867.73 | 13867.14 |
| 2131 | 1996.38 |  | 5372.4 | 7645.69 |  | 8043.7 | 22504.2 |
| 9703.91 | 8869.731 |  | 5879.48 | 2665.4 |  | 2657.9 | 16682.39 |
| 2958.66 | 5054.2 |  | 2960.06 | 2281.66 |  | 3335.2 | 14272.55 |
| 1625.14 | 2266.71 |  | 7422.19 | 6666.44 |  | 1222 | 11139.09 |
| 4329.14 | 3639.98 |  | 5812.34 | 6246.9 |  | 4811.77 | 18157.15 |
| 3724 | 7473.83 |  | 6982.85 | 3436.53 |  | 1982.76 | 4770.89 |
| 3285.47 | 4196.34 |  | 5486.38 | 3344.98 |  | 1933.03 | 9433.37 |
| 4194.26 | 7888.75 |  | 8249.52 | 5945.86 |  | 3871 | 19420.06 |
| 2043.13 | 1691.56 |  | 5342.85 | 3471.8 |  | 1783.7 | 11089.51 |
| 5826.5 | 3467.05 |  | 5008.28 | 6069.2 |  | 3055.02 | 11028.25 |
| 10115.99 | 6346.87 |  | 4121.28 | 3870.55 |  | 3721.63 | 12815.96 |
| 3078.86 | 6269.45 |  | 5219.88 | 4890.32 |  | 3639.04 | 12039.92 |
| 2806.62 | 2567.73 |  | 5350.64 | 4949.85 |  | 2947.75 | 7339.58 |
| 6621.95 | 4395.08 |  | 4351.34 | 2827.7 |  | 5064.45 | 21362.83 |
| 2120.96 | 2298.69 |  | 4222.8 | 2319.51 |  | 1904.17 | 11748.01 |
| 2152.05 | 3463.7 |  | 3495.75 | 4128.98 |  | 1384.62 | 12859.06 |
| 3632.21 | 2907.97 |  | 9937.76 | 3041.06 |  | 3060.19 | 18095.17 |
| 9851.42 | 9585.53 |  | 5264.38 | 3865.48 |  | 2988.75 | 16685.04 |
| 12227.69 | 10256.75 |  | 4088.26 | 4507.77 |  | 2399.95 | 6421.85 |
| 8889.94 | 12211.92 |  | 8722.34 | 5166.34 |  | 1305.96 | 7506.74 |
| 7373.64 | 6963.7 |  | 2004.62 | 2887.12 |  | 4567.43 | 12416.87 |
| 7564.16 | 6693.39 |  | 7111.24 | 4498.61 |  | 3518.19 | 9182.93 |
| 5266.39 | 5621.49 |  | 2847.3 | 2632.15 |  | 2928.65 | 11446.18 |
| 19389.55 | 16728.23 |  | 6926.83 | 4186.19 |  | 1346.43 | 7053.09 |
| 8908.79 | 6057.61 |  | 3888.86 | 2912.59 |  | 4925.49 | 15684.79 |
| 18645.19 | 10344.28 |  | 3718.5 | 3067.48 |  | 4124.97 | 13511.3 |
| 22155.11 | 11508.47 |  | 6930.13 | 5353.09 |  | 4069.73 | 19214.94 |
| 14949.02 | 11233.2 |  | 6267.21 | 3421.88 |  | 2791.56 | 10999.97 |
| 7755.7 | 8440.24 |  | 4518.51 | 3151.12 |  | 2029.9 | 11635.72 |
| 3946.06 | 9278.21 |  | 2724.48 | 4759.76 |  | 3898.23 | 11264.08 |
| 8049.23 | 7869.3 |  | 6279.15 | 3910.76 |  | 2452.25 | 4990.51 |
| 9979.83 | 17158.22 |  | 4924.25 | 4436.22 |  | 1824.23 | 9496.83 |
| 12213.88 | 8965.54 |  | 1661.79 | 2372.28 |  | 1452.94 | 4602.47 |
| 12407.63 | 8123.45 |  | 2257.28 | 2435.35 |  | 2519.4 | 20651.04 |
| 8251.06 | 14370.04 |  | 4533.09 | 2892.45 |  | 1716.14 | 8860.15 |
| 14992.48 | 8490.53 |  | 5675.33 | 4680.18 |  | 788.87 | 7474.37 |
| 6627.83 | 9500.99 |  | 4735.71 | 3788.36 |  | 4382.48 | 20497.69 |
| 12262.98 | 10760.11 |  | 3975.06 | 3298.31 |  | 1687.89 | 11584.17 |
| 19240.99 | 14033.36 |  | 2215.71 | 1915.2 |  | 1447.36 | 11801.47 |
| 20794.93 | 16521.43 |  | 4088.25 | 2975.21 |  | 1934.78 | 8488.71 |
| 1312.98 | 2786.69 |  | 13149.98 | 12039.26 |  | 6563.35 | 19349.13 |
| 31146.7 | 19948.11 |  | 10096.6 | 9903.29 |  | 1866.35 | 12035.23 |
| 6451.73 | 3505.79 |  | 18407.55 | 13991.78 |  | 3507.36 | 17600.45 |
| 4596.66 | 4787.1 |  | 5560.31 | 6396.64 |  | 1659.13 | 7935.4 |
| 9857.99 | 12373.17 |  | 2117.93 | 6602.31 |  | 5700.88 | 22518.13 |
| 6158.98 | 6377.26 |  | 16950.11 | 11610.6 |  | 3412.93 | 22452.92 |
| 9289.61 | 7009.27 |  | 2772.61 | 5332.6 |  | 2177.09 | 8599.33 |
| 16911.73 | 14301.47 |  | 4273.63 | 4605.33 |  | 1897.57 | 13573.97 |
| 5649.17 | 5021.09 |  | 2772.24 | 4104.53 |  | 5963.56 | 19520.58 |
| 16439.43 | 10075.49 |  | 3192.36 | 4878.83 |  | 1859.97 | 8857.29 |
| 11272.38 | 7713.37 |  | 12563.53 | 8436.62 |  | 2210.11 | 7733.34 |
| 12550.14 | 10433.81 |  | 7962.93 | 6323.04 |  | 5910.45 | 25864.57 |
| 12518.03 | 11959.16 |  | 5235.87 | 4557.53 |  | 5838.17 | 16873.52 |
| 3456.63 | 5593.71 |  | 9438.12 | 5241.75 |  | 4597.99 | 17238.99 |
| 3962.64 | 5914.52 |  | 4847.34 | 4341.81 |  | 2461.97 | 17513.58 |
| 12962.24 | 8917.481 |  | 13319.51 | 10178.72 |  | 3517.86 | 14050.37 |
| 12225.48 | 6740.77 |  | 10996.16 | 6640.91 |  | 3910 | 13369.93 |
| 5222.25 | 7164.91 |  | 19431.32 | 19010.22 |  | 5720.43 | 25354.93 |
| 12892.23 | 8470.53 |  | 5292.35 | 5308.03 |  | 2577.73 | 11787.27 |
| 6579.73 | 4668.17 |  | 7408.45 | 8182.47 |  | 2975.66 | 20749.14 |
| 6673.63 | 6023.5 |  | 4290.19 | 5306.46 |  | 873.11 | 16492.31 |
| 6172.14 | 5063.67 |  | 5142.74 | 4972.86 |  | 1276.8 | 9488.52 |
| 5745.87 | 5632.58 |  | 5454.22 | 7673.5 |  | 2016.57 | 12549.55 |
| 5978.31 | 4026.86 |  | 7549.47 | 7337.41 |  | 2289.6 | 11475.52 |
| 5336.25 | 4128.2 |  | 8618.21 | 6540.03 |  | 2288.25 | 10767.78 |
| 4514.04 | 4851.25 |  | 9640.74 | 7358.13 |  | 3027.37 | 15938.96 |
| 7824.53 | 6709.99 |  | 5829.11 | 5097.12 |  | 4504.1 | 18622.73 |
| 5585.81 | 6260.87 |  | 9965.97 | 6834.48 |  | 1289.13 | 12429.44 |
| 10932.75 | 8562.07 |  | 1531.01 | 4994.23 |  | 2715.34 | 11622.13 |
| 7557.37 | 6478.51 |  | 5952.4 | 5365.47 |  | 4483.03 | 17721.61 |
| 10468.87 | 5700.41 |  | 8088.85 | 6974.09 |  | 1199.36 | 12105.29 |
| 6337.28 | 4490.28 |  | 3982.29 | 7261.79 |  | 2660.47 | 16359.1 |
| 14175.05 | 10281.43 |  | 6240.3 | 4967.58 |  | 7130.01 | 20087.63 |
| 6034.22 | 4944.42 |  | 3650.03 | 5431.19 |  | 1613.49 | 4598.79 |
| 4683.82 | 4384.98 |  | 10896.04 | 8162.69 |  | 3435.2 | 20871.2 |
| 7650.01 | 3902.55 |  | 2105.03 | 4690.52 |  | 1780.24 | 13520.86 |
| 3978.78 | 3184.95 |  | 1550.45 | 2814 |  | 2106.58 | 11580.63 |
| 7208.62 | 8784.96 |  | 2684.13 | 4006.38 |  | 1778.4 | 10583.07 |
| 2706.8 | 3190.29 |  | 14059.34 | 7190.7 |  | 3490.03 | 14992.12 |
| 4976.85 | 3434.77 |  | 2753.47 | 5282.89 |  | 1379.77 | 7506.03 |
| 17513.23 | 9623.87 |  | 2594.57 | 2557.54 |  | 1887.53 | 7104.57 |
| 12302.23 | 7973.8 |  | 749.4 | 1420.87 |  | 5479.82 | 22605.5 |
| 6194.43 | 8293.44 |  | 2991.31 | 2797.12 |  | 4568.23 | 14985.48 |
| 7422.15 | 7048.55 |  | 3178.94 | 7611.54 |  | 1746.55 | 9509.13 |
| 7300.02 | 13153.32 |  | 4225.81 | 3396.18 |  | 2830.47 | 10558.42 |
| 9633.72 | 5743.67 |  | 1498.77 | 5847.13 |  | 1645.81 | 6824.74 |
| 6648.45 | 7316.18 |  | 2385.12 | 2974.6 |  | 1788.89 | 15470.27 |
| 7295.51 | 6442.99 |  | 2152.42 | 4542.31 |  | 2129.38 | 12178.42 |
| 7898.63 | 7484.69 |  | 1163.7 | 1987.99 |  | 3549.77 | 23091.65 |
| 9516.19 | 8429.19 |  | 2882.34 | 3797.48 |  | 3093.11 | 12965.43 |
| 9630.71 | 12217 |  | 1369.7 | 2622.58 |  | 2015.21 | 12396.22 |
| 7314.7 | 4034.19 |  | 524.11 | 2022.37 |  | 3717.42 | 16516.85 |
| 6840.18 | 8525.03 |  | 1993.06 | 7280.05 |  | 1419.6 | 9370.85 |
| 11060.87 | 11914.87 |  | 2162.16 | 3349.73 |  | 2666.56 | 14675.04 |
| 4093.71 | 6293.08 |  | 2309.54 | 3026.33 |  | 1917.62 | 6451.54 |
| 10573.37 | 6181.03 |  | 954.79 | 2586.65 |  | 2754.17 | 10469.14 |
| 12149.54 | 12268.13 |  | 918.45 | 2093.34 |  | 5071.72 | 20323.94 |
| 1716 | 2255.07 |  | 3501.2 | 3041.9 |  | 2593.23 | 16100.79 |
| 11477.12 | 8932 |  | 13981.9 | 3910.48 |  | 5256.34 | 17912.85 |
| 6703.66 | 5822.57 |  | 4983.22 | 2142.62 |  | 4011.09 | 13480.34 |
| 5272.56 | 2703.54 |  | 8408.71 | 6241.71 |  | 2287.61 | 15346.27 |
| 8280.54 | 5877.57 |  | 2454.87 | 3446.46 |  | 5125.09 | 14683.29 |
| 3245.37 | 4628.4 |  | 8467.83 | 6440.6 |  | 4580.09 | 15008.4 |
| 3636.53 | 7127.16 |  | 6165.05 | 3269.37 |  | 2767.29 | 10914.88 |
| 2687.89 | 3823.49 |  | 13675.85 | 7404.14 |  | 2534.65 | 7054.01 |
| 3098.76 | 4057.14 |  | 6064.55 | 3858.24 |  | 2064.65 | 8831.981 |
| 4505.52 | 3223 |  | 12465.73 | 6912.09 |  | 1687.5 | 3775.44 |
| 13823.52 | 12746.94 |  | 11425.41 | 5728.51 |  | 1713.63 | 8919.21 |
| 14935.65 | 7934.59 |  | 3926.89 | 5076.96 |  | 1422.15 | 12438.32 |
| 6003.79 | 6102.2 |  | 21189.16 | 10253.08 |  | 4824.1 | 18695.01 |
| 3762.89 | 4254.54 |  | 9083.45 | 6287.2 |  | 2461.12 | 15504.22 |
| 1690.79 | 5022.65 |  | 2833.15 | 3627.89 |  | 1997.82 | 10192.36 |
| 2462.45 | 6391.45 |  | 3843.59 | 3330.62 |  | 4424.63 | 10398.33 |
| 2398.63 | 4291.87 |  | 5842.86 | 5176.56 |  | 5329.43 | 16726.99 |
| 8583.51 | 5190.52 |  | 8173.24 | 4139.06 |  | 3265.51 | 9032.4 |
| 6700.6 | 5134.44 |  | 8511.13 | 5154.76 |  | 3839.52 | 19442.4 |
| 8705.59 | 5493.95 |  | 4081.81 | 2889.57 |  | 2294.56 | 10838.11 |
| 1672.99 | 1871.96 |  | 15500.14 | 11164.13 |  | 1948.29 | 14927.54 |
| 6827.85 | 3627.34 |  | 2147.59 | 4278.92 |  | 5205.74 | 15906.83 |
| 5372.98 | 5585.35 |  | 11886.46 | 7827.78 |  | 1369.49 | 12935.74 |
| 5621.36 | 7647.73 |  | 8572.4 | 6164.27 |  | 1622.1 | 7160.03 |
| 13028.37 | 12495.17 |  | 11385.97 | 7259.45 |  | 2077.23 | 15585.5 |
| 14172.17 | 8230.6 |  | 4499.28 | 4889.32 |  | 1065.67 | 7559.99 |
| 6546.54 | 5108.96 |  | 1087.15 | 1295.81 |  | 2861.64 | 10550.37 |
| 6490.54 | 5882.14 |  | 6367.24 | 7294.09 |  | 4821.1 | 9048.22 |
| 11205.11 | 6712.49 |  | 4210.27 | 3932.57 |  | 8207.77 | 17927.17 |
| 13552.02 | 7449.73 |  | 9365.9 | 4875.06 |  | 12726.77 | 15401 |
| 16585.51 | 13778.19 |  | 7943.7 | 5503.18 |  | 877.69 | 2648.54 |
| 1820 | 2642.16 |  | 11390.4 | 8166.02 |  | 4667.17 | 6807.92 |
| 7060.21 | 7061.65 |  | 4538.22 | 3181.13 |  | 1089.1 | 3051.43 |
| 2853.62 | 6466.49 |  | 4294.16 | 2829.79 |  | 5392.14 | 9121.37 |
| 2260.94 | 2484.9 |  | 2874.92 | 2930.27 |  | 3207.92 | 3536.16 |
| 11522 | 13365.69 |  | 13371.79 | 6888.08 |  | 3752.83 | 6229.58 |
| 4196.41 | 3003.85 |  | 2722.84 | 2701.26 |  | 4114.72 | 7446.12 |
| 4687.9 | 5723.67 |  | 6303.4 | 4948.45 |  | 7187.8 | 16548.35 |
| 2241.87 | 3001.34 |  | 11778.86 | 12689.25 |  | 10356.03 | 15471.11 |
| 9192.53 | 4738.49 |  | 10153.53 | 6128.21 |  | 13775.92 | 14820.69 |
| 3543.93 | 6350.12 |  | 3075.78 | 3231.46 |  | 7653.56 | 11573.85 |
| 10157.7 | 9850.58 |  | 8807.91 | 8204.25 |  | 4008.75 | 7020.87 |
| 7678.23 | 3489.18 |  | 1953.16 | 2671.03 |  | 6340.5 | 7694.02 |
| 4010.46 | 2090.85 |  | 8369.37 | 5606.12 |  | 7127.23 | 9585.52 |
| 4086.39 | 5829.57 |  | 3327.97 | 4758.18 |  | 9240.47 | 14877 |
| 8479.06 | 4889.32 |  | 3538.5 | 3606.7 |  | 8101.54 | 5623.87 |
| 3960.24 | 4005.69 |  | 4591.92 | 4962.36 |  | 2191.67 | 7483.13 |
| 4582.58 | 4337.1 |  | 14083.04 | 9281.26 |  | 4143.81 | 10273.05 |
| 7520.84 | 4263.62 |  | 1171.4 | 2516.43 |  | 2156.79 | 9305.3 |
| 2303.69 | 2199.7 |  | 12463.14 | 7881.91 |  | 4743.96 | 4850.79 |
| 5015.07 | 6079.37 |  | 9349.74 | 6984.56 |  | 4040.55 | 7834.11 |
| 2586.35 | 1993.64 |  | 2777.55 | 3152.93 |  | 11051.53 | 8587.13 |
| 3775.89 | 5849.66 |  | 14710.23 | 10214.94 |  | 11222.63 | 17459.62 |
| 5245.39 | 4994.15 |  | 4407.59 | 3133.87 |  | 5474.44 | 10937.22 |
| 3035.42 | 3943.36 |  | 4135.44 | 4572.95 |  | 8074.73 | 8298.24 |
| 21284.53 | 11791.1 |  | 4597.08 | 4527.21 |  | 5241.14 | 6123.88 |
| 3252.55 | 8187.45 |  | 3704.22 | 3139.51 |  | 12158.31 | 8976.72 |
| 2427.13 | 1782.34 |  | 13593.75 | 10373.07 |  | 4187.93 | 6976.88 |
| 2774.57 | 3054.29 |  | 9472.61 | 8357 |  | 2305.35 | 5787.85 |
| 3390.75 | 2216.79 |  | 3806.25 | 3605.3 |  | 5439.46 | 7026.24 |
| 6995.11 | 4023.3 |  | 3897.39 | 3905.85 |  | 2905.8 | 5372.15 |
| 3937.61 | 3400.04 |  | 11066.28 | 11973.95 |  | 11079.45 | 21928.15 |
| 5670.48 | 5378.64 |  | 11696.36 | 6021.77 |  | 3409.04 | 5848.67 |
| 1320.25 | 4345.54 |  | 1141.91 | 2993.93 |  | 4855.25 | 8805.39 |
| 4906.2 | 2739.12 |  | 5757.47 | 6654.35 |  | 3446.19 | 9460.67 |
| 2491.39 | 3660.86 |  | 5936.71 | 3587.53 |  | 4973.25 | 6407.86 |
| 2580.64 | 2477.96 |  | 3646.83 | 4716.26 |  | 5377.43 | 7376.7 |
| 1872.46 | 1514.19 |  | 3023.67 | 2204.01 |  | 6664.8 | 12850.3 |
| 4468.19 | 4005.72 |  | 1846.25 | 1975.17 |  | 6212.39 | 5320.91 |
| 4361.24 | 2631.72 |  | 4665.57 | 4617.06 |  | 6051.01 | 11042.09 |
| 3612.89 | 1735.81 |  | 7802.01 | 6250.72 |  | 4667.62 | 10368.23 |
| 10457.15 | 6649.54 |  | 11945.64 | 7287.81 |  | 1490.81 | 7055.74 |
| 4673.59 | 3348.89 |  | 16215.59 | 11206.4 |  | 6253.1 | 5146.15 |
| 3012.01 | 3460.42 |  | 7413.1 | 4463.08 |  | 3279.44 | 6714.1 |
| 3662.81 | 3254.02 |  | 1872.89 | 4444.59 |  | 3256.27 | 11408.38 |
| 2633.77 | 5090.89 |  | 14454.25 | 13061.78 |  | 1881.3 | 11655.06 |
| 3335.76 | 3549.97 |  | 2230.1 | 2782.45 |  | 3090.74 | 3606.95 |
| 2990.2 | 3277.04 |  | 1902.89 | 1753.87 |  | 2136.42 | 5937.42 |
| 2861.41 | 2999.42 |  | 3230.51 | 5684.97 |  | 3494.88 | 7593.65 |
| 1291.14 | 1466.7 |  | 6215.54 | 2955.73 |  | 6983.23 | 10005.97 |
| 3567.46 | 3492.17 |  | 4222.93 | 3786.99 |  | 12869.08 | 11437.54 |
| 3254.48 | 4130.05 |  | 5748.17 | 4471.13 |  | 5350.16 | 9121.12 |
| 3430.3 | 2741.73 |  | 15570.66 | 8787.68 |  | 8138.15 | 9650.03 |
| 3456.36 | 6124.16 |  | 7289.8 | 8419.76 |  | 14311.74 | 9773.92 |
| 4289.94 | 3801.1 |  | 2817.9 | 3622.18 |  | 13516.18 | 10118.41 |
| 3223.43 | 2145.94 |  | 6158.65 | 7825.51 |  | 8720.7 | 8227.06 |
| 17332.74 | 6756.48 |  | 1766.92 | 2482.85 |  | 8437.65 | 11862.12 |
| 3125.65 | 3660.21 |  | 3241.37 | 3143.36 |  | 4479.21 | 9355.37 |
| 3362.93 | 3750.17 |  | 5404.88 | 6440.37 |  | 12588.37 | 9165.69 |
| 2145.72 | 2944.71 |  | 5838.32 | 4635.91 |  | 13852.43 | 10564.3 |
| 2104.08 | 4051.87 |  | 3569.94 | 2795.02 |  | 16343.46 | 21207.3 |
| 9824.76 | 11051.6 |  | 16730.95 | 9188.93 |  | 16589.76 | 13359.56 |
| 4708.05 | 5517.79 |  | 9794.51 | 9397.45 |  | 6231.86 | 11973.36 |
| 1151.79 | 2113.67 |  | 11045.2 | 5599.83 |  | 6143.29 | 11882.69 |
| 2073.63 | 5657.82 |  | 3344.96 | 6557.29 |  | 14496.47 | 16177.92 |
| 8136.34 | 5899.73 |  | 3103.96 | 4725.77 |  | 9807.84 | 10847.08 |
| 2818.47 | 2141.21 |  | 6102.52 | 6118.81 |  | 14983.39 | 18538.27 |
| 19913.15 | 13692.35 |  | 3815.53 | 4160.36 |  | 16489.93 | 24598.94 |
| 3154.68 | 4675.99 |  | 4281.23 | 3274.16 |  | 11300.79 | 13651.28 |
| 4062.74 | 5551.8 |  | 5077.22 | 4527.63 |  | 15871.3 | 9209.93 |
| 1484.52 | 2963.21 |  | 1865.45 | 3467.02 |  | 3238.86 | 14611.94 |
| 15759.65 | 7463.71 |  | 5311.91 | 4721.89 |  | 14970.66 | 11052.23 |
| 2120.57 | 5328.88 |  | 2209.93 | 3197.08 |  | 14957.45 | 9467.35 |
| 5247.23 | 6813.91 |  | 4603.27 | 3042.63 |  | 10807.82 | 11351.64 |
| 4754.4 | 4366.34 |  | 6173.37 | 5574.86 |  | 8874.03 | 14745.69 |
| 6481.99 | 3070.82 |  | 3791.34 | 3463.21 |  | 10551.19 | 8813.53 |
| 3554.09 | 2854.26 |  | 1757.92 | 2681.38 |  | 6890.07 | 8498.88 |
| 3081.96 | 6221.7 |  | 11472.3 | 8193.88 |  | 7613.62 | 15391.89 |
| 2801.08 | 4426.49 |  | 7135.65 | 4612.71 |  | 5023.62 | 10302.09 |
| 1628.03 | 2289.95 |  | 11413.29 | 14062.94 |  | 7405.75 | 9577.15 |
| 2883.57 | 5223 |  | 3767.25 | 2708.91 |  | 6480.94 | 8255.61 |
| 2668.22 | 2165.32 |  | 3788.61 | 2270.28 |  | 5833.29 | 10124.63 |
| 2657.32 | 6742.92 |  | 4996 | 3066.25 |  | 11619.27 | 9990.59 |
| 1868.83 | 4823.63 |  | 2391.71 | 2003.52 |  | 12581.9 | 20973.75 |
| 2045.15 | 2388.62 |  | 2734.49 | 3934.29 |  | 11671.61 | 10118.92 |
| 12689.95 | 7534.8 |  | 2094.34 | 1593.75 |  | 10133.03 | 11207.13 |
| 4277.6 | 5098.78 |  | 1847.48 | 3533.94 |  | 4579.32 | 9794.9 |
| 7796.47 | 7794.29 |  | 4464.95 | 3190.08 |  | 5666.02 | 9390.58 |
| 4447.04 | 3895.18 |  | 7289.41 | 4229.7 |  | 10870.59 | 12765.52 |
| 9707.4 | 5168.75 |  | 3348.47 | 6172.79 |  | 9310.96 | 15402.86 |
| 2720.82 | 5388.76 |  | 2127.17 | 2549.83 |  | 4621.61 | 10850.89 |
| 13061.7 | 7080.58 |  | 4572.96 | 5077.35 |  | 2865.31 | 5474.32 |
| 2448.49 | 4231.22 |  | 9645.21 | 6795.16 |  | 4862.06 | 9565.78 |
| 1881.09 | 2603.3 |  | 3175.26 | 4453.31 |  | 2922.99 | 6459.68 |
| 1155.3 | 7245.27 |  | 2375.51 | 2409.58 |  | 15048.9 | 19183.95 |
| 1801.81 | 4072.22 |  | 5563.5 | 3914.4 |  | 6105.07 | 7821.61 |
| 4439.66 | 4584.64 |  | 2526.57 | 2394.8 |  | 10123.43 | 9218.09 |
| 2099.06 | 4023.53 |  | 2240.25 | 5696.52 |  | 3903.52 | 6221.66 |
| 1220.36 | 3256.06 |  | 2723.5 | 4045.25 |  | 2294.04 | 10412.3 |
| 4045.5 | 8452.51 |  | 3091.78 | 1664.29 |  | 2401.48 | 4127.87 |
| 3800.53 | 4034.18 |  | 2297.62 | 2946.28 |  | 5690.99 | 7071.88 |
| 1189.93 | 4137.61 |  | 5090.5 | 4356.14 |  | 5091.96 | 8497.5 |
| 1780.79 | 4595.09 |  | 4532.85 | 7604.81 |  | 8922.91 | 9891.12 |
| 6550.92 | 4171.77 |  | 4339.31 | 3788.01 |  | 12416.78 | 8708.03 |
| 5352.14 | 4296.9 |  | 5682.66 | 2814.95 |  | 14055.44 | 16898.77 |
| 3967.08 | 3516.24 |  | 11126.85 | 6047.74 |  | 14251.42 | 19340.21 |
| 2056 | 3906.03 |  | 3037.27 | 4611.43 |  | 3298.6 | 5781.56 |
| 1598.12 | 3479.16 |  | 2422.12 | 3400.74 |  | 14109.27 | 15097.93 |
| 1356.53 | 3481.04 |  | 4682.84 | 4590.02 |  | 10309.35 | 14544.79 |
| 5974.47 | 4575.29 |  | 12275.06 | 7865.45 |  | 10463.63 | 9646.731 |
| 8531.731 | 4618.65 |  | 3691.46 | 2710.7 |  | 7112.52 | 11785.78 |
| 3284.54 | 3627.61 |  | 4285.85 | 6091.94 |  | 5214.2 | 8972.33 |
| 1632.85 | 4307.11 |  | 2234.82 | 4730.69 |  | 9008.45 | 6459.29 |
| 4015.03 | 4712.64 |  | 1972.54 | 3042.71 |  | 6953.97 | 6790 |
| 19274 | 12863.22 |  | 9685.6 | 8595.66 |  | 3831.01 | 9543.63 |
| 4504.35 | 5222.93 |  | 7276.57 | 5043.37 |  | 4129.16 | 7635.18 |
| 12650.22 | 7178.02 |  | 8560.28 | 5411.13 |  | 7250.79 | 6206.6 |
| 4409.36 | 5382.39 |  | 5147.67 | 4620.7 |  | 7689.99 | 8609.37 |
| 2729.49 | 5406.72 |  | 9463.88 | 7329.75 |  | 7381.56 | 7145.99 |
| 3287.96 | 3846.22 |  | 3149.81 | 4273.94 |  | 10200.78 | 14194.21 |
| 2871.19 | 3812.03 |  | 3118.17 | 2332.41 |  | 6752.93 | 9747.08 |
| 15976.77 | 16899.71 |  | 3616.53 | 4576.99 |  | 12485.95 | 13976.92 |
| 2478.16 | 4108.33 |  | 4469.28 | 2738.33 |  | 8327.88 | 10970.66 |
| 13933.91 | 8754.33 |  | 1625.93 | 3436.07 |  | 2798.25 | 12786.03 |
| 18954.67 | 9887.22 |  | 1472.12 | 1408.77 |  | 5917.49 | 7815.7 |
| 4145.75 | 6702.84 |  | 2140.04 | 1618.12 |  | 15174.87 | 14989.01 |
| 4203.81 | 6237.99 |  | 4259.1 | 3026.94 |  | 14820.13 | 14473.26 |
| 4248.67 | 3910.68 |  | 2541.52 | 1792.09 |  | 8096.96 | 15774.21 |
| 5343.9 | 5414.83 |  | 1430.26 | 1182.17 |  | 13148.95 | 16432.5 |
| 2649.33 | 5426.2 |  | 600.9 | 704.24 |  | 20241.07 | 23366.28 |
| 3372.21 | 5846.06 |  | 2380.41 | 1433.11 |  | 10835.24 | 10854.6 |
| 4326.28 | 3864.14 |  | 1429.46 | 1425.03 |  | 6282.86 | 8016.14 |
| 13917.8 | 5298.76 |  | 3237.08 | 2051.62 |  | 4978.73 | 9076.981 |
| 2292.41 | 2053.28 |  | 7523.73 | 3994.74 |  | 4710.63 | 4687.68 |
| 9438.2 | 8018.95 |  | 2313.2 | 2081.41 |  | 8585.92 | 17753.75 |
| 5475.64 | 3212.98 |  | 3220.41 | 1537.06 |  | 3005.05 | 5585.61 |
| 3115.59 | 7199.43 |  | 1253.51 | 1809.05 |  | 4742.37 | 6799.43 |
| 6618.77 | 11828.37 |  | 1455.24 | 1192.28 |  | 19452.96 | 16048.53 |
| 4868.94 | 3822.36 |  | 4893.27 | 2499.39 |  | 11133.28 | 15343.24 |
| 4769.8 | 4992.52 |  | 576.8 | 1264.9 |  | 10125.27 | 10542.04 |
| 7559.35 | 6686.96 |  | 3972.2 | 2615.6 |  | 14848.59 | 17885.38 |
| 6237.44 | 14190.06 |  | 401.86 | 369.57 |  | 17130.18 | 19494.2 |
| 4540.22 | 3560.45 |  | 3297.99 | 2350.66 |  | 15962.41 | 21899.22 |
| 9635.14 | 11095.1 |  | 4015.32 | 3122.88 |  | 18739.63 | 21605.79 |
| 3801.21 | 6696.08 |  | 2826.46 | 1665.18 |  | 4127.91 | 7031.33 |
| 7362.2 | 9289.96 |  | 4089.73 | 3416.64 |  | 10515.14 | 10002.55 |
| 4319.39 | 5192.42 |  | 3678.38 | 3086.62 |  | 15208.67 | 15608.88 |
| 3951.8 | 2942.05 |  | 3563.88 | 1986.98 |  | 9573.66 | 8264.42 |
| 7622.09 | 11119.65 |  | 2887.34 | 1469.48 |  | 18386.47 | 14674.47 |
| 1768.33 | 2973.33 |  | 6715.91 | 4397.56 |  | 3477.76 | 4644.05 |
| 11663.8 | 8023.62 |  | 4522.96 | 2612.7 |  | 5073.06 | 8463.82 |
| 5283.7 | 3930.32 |  | 4523.6 | 1941.36 |  | 6755.56 | 10832.89 |
| 2287.77 | 3097.46 |  | 717.46 | 1122.75 |  | 8503.981 | 12034.61 |
| 3699.01 | 6280.78 |  | 6290.38 | 3212.63 |  | 8591.94 | 8178.76 |
| 3380.81 | 5393.17 |  | 1539.24 | 1490.95 |  | 11390.48 | 6834.74 |
| 4819.29 | 7893.12 |  | 4093.13 | 3462.41 |  | 11472.8 | 20548.91 |
| 15059.52 | 8327.2 |  | 5680.91 | 2346.32 |  | 4789.18 | 10002.55 |
| 7510.72 | 5948.35 |  | 1814.3 | 4163.91 |  | 13827.56 | 16288.67 |
| 2246.37 | 3361.09 |  | 1172.92 | 823.78 |  | 3366.97 | 5173.49 |
| 5541.27 | 10402.71 |  | 3948.14 | 3832.57 |  | 5840.13 | 7712.78 |
| 1991.29 | 1960.97 |  | 4325.93 | 3052.62 |  | 11213.86 | 8116.09 |
| 4217.29 | 5856.04 |  | 960.45 | 2648.14 |  | 10565.81 | 10534.18 |
| 2538.3 | 3592.07 |  | 5568.18 | 3186.38 |  | 9576.07 | 12517.01 |
| 4070.96 | 5700.65 |  | 3273.23 | 1526.3 |  | 7708.96 | 18009.53 |
| 5480.31 | 5187.49 |  | 1912.87 | 1880.98 |  | 17959.84 | 16975.33 |
| 7004.88 | 6124.17 |  | 4362.46 | 3349.33 |  | 3309.54 | 5702.33 |
| 11472.63 | 6648.05 |  | 4671.68 | 3032.07 |  | 13339.98 | 14175.55 |
| 10371.28 | 12327.08 |  | 5544.93 | 3445.68 |  | 13182.45 | 14701.89 |
| 6763.59 | 6687.88 |  | 3966.62 | 2258.25 |  | 7808.59 | 6617.37 |
| 2512.16 | 4895.06 |  | 4526.55 | 2522.71 |  | 6049.52 | 7554.54 |
| 5369.61 | 4284.03 |  | 4525.85 | 3735.2 |  | 24601.54 | 20691.92 |
| 2946.11 | 1652.23 |  | 7059.68 | 3662.57 |  | 3772.79 | 4835 |
| 3629.17 | 5028.38 |  | 826.26 | 1265.81 |  | 6710.44 | 8662.46 |
| 5972.51 | 4848.2 |  | 4857.83 | 2670.71 |  | 18557.46 | 17318.91 |
| 2503.39 | 2703.91 |  | 1836.49 | 1111.01 |  | 12919.69 | 9652.43 |
| 3949.96 | 4893.91 |  | 6221.32 | 3011.76 |  | 13750.38 | 17436.96 |
| 3957.01 | 3917.47 |  | 1308.45 | 1473.22 |  | 17486.13 | 20952.96 |
| 5420.17 | 3257.77 |  | 5275.76 | 3719.12 |  | 20111.03 | 18018.96 |
| 5128.41 | 3474.79 |  | 3427.83 | 1977.07 |  | 7513.84 | 11539.54 |
| 3542.33 | 4823.21 |  | 3008.12 | 1716.3 |  | 4759.29 | 6299.47 |
| 3760.06 | 2410.61 |  | 1830.18 | 1281.15 |  | 6070.33 | 12410.68 |
| 4205.3 | 2711.01 |  | 3628.58 | 4927.95 |  | 8427.04 | 8120.97 |
| 6302.27 | 3348.56 |  | 1019.42 | 1166.82 |  | 18696.75 | 14162.48 |
| 5851.58 | 9846.35 |  | 7400.83 | 5702.34 |  | 17648.67 | 25905.69 |
| 5578.28 | 4671.71 |  | 2988.69 | 3426.67 |  | 11361.16 | 17605.56 |
| 4472.89 | 3010.28 |  | 994.39 | 1206.07 |  | 13758.92 | 8485.85 |
| 7397.41 | 5979.02 |  | 6037.45 | 3262.6 |  | 11810.9 | 7482.14 |
| 3868.52 | 4497.45 |  | 4660.3 | 3423.42 |  | 15505.22 | 18495.15 |
| 1892.54 | 5568.6 |  | 775.11 | 1586.21 |  | 6061.12 | 7470.12 |
| 1577.13 | 3039.32 |  | 2944.37 | 1976.6 |  | 11587.05 | 11432.15 |
| 14520.68 | 9168.79 |  | 5991.72 | 3157.14 |  | 8653.82 | 12237.73 |
| 5139.68 | 5193.97 |  | 2329.76 | 2361.26 |  | 19429.08 | 19998.13 |
| 12809.6 | 9836.71 |  | 700.82 | 2119.71 |  | 5200.72 | 5693.72 |
| 14491.73 | 13917.72 |  | 6132.85 | 3696.54 |  | 9467.62 | 8911.49 |
| 1789.47 | 2782.84 |  | 4895.99 | 2605.37 |  | 12316.28 | 8755.11 |
| 2228.85 | 4474.87 |  | 1606.65 | 1315.56 |  | 16688.91 | 17754.01 |
| 4989.06 | 3014.68 |  | 3538.54 | 2184.82 |  | 4664.99 | 5844.6 |
| 2265.07 | 3652.82 |  | 5018.64 | 2763.25 |  | 12859.46 | 17329.53 |
| 6032.96 | 6581.02 |  | 2535.22 | 2118.04 |  | 3278.76 | 8769.86 |
| 14881.37 | 6330.33 |  | 1814.21 | 958.65 |  | 18658.26 | 17656.39 |
| 11251.74 | 7533.15 |  | 1699.74 | 1335.93 |  | 8796.21 | 6268.38 |
| 7716.5 | 5199.65 |  | 1635.15 | 1412.75 |  | 6373.7 | 6482.38 |
| 1067.33 | 3342.75 |  | 2381.2 | 1954.56 |  | 9129.31 | 13744.11 |
| 4265.56 | 7659.03 |  | 3641.5 | 2312.71 |  | 13408.87 | 11683.72 |
| 4176.74 | 3490.99 |  | 3934.49 | 2103.37 |  | 11064.99 | 13259.61 |
| 3382.73 | 1947.94 |  | 4715.34 | 3418.06 |  | 7026.35 | 9270.95 |
| 4636.69 | 5047.84 |  | 1436.04 | 2234.13 |  | 20826.91 | 16788.96 |
| 3042.31 | 5203.94 |  | 3542.58 | 2280.86 |  | 3648.9 | 9075.99 |
| 1767 | 4389.18 |  | 1037.64 | 1721.27 |  | 5589.23 | 7690.36 |
| 9049.86 | 9935.79 |  | 2771.84 | 2744.05 |  | 4691.11 | 13533.87 |
| 4816.69 | 5007.86 |  | 1515.16 | 1983.58 |  | 22138.44 | 22592.69 |
| 1193.3 | 2543.69 |  | 3629.77 | 8079.23 |  | 8187.86 | 17505.27 |
| 2341.6 | 5461.42 |  | 1428.12 | 3023.12 |  | 20929.7 | 17471.35 |
| 4340.8 | 4616.15 |  | 2732.77 | 3250.78 |  | 13643.93 | 10396.91 |
| 15348.15 | 7973.66 |  | 1114.79 | 3467.65 |  | 8604.47 | 11132.94 |
| 4551.45 | 2980.81 |  | 3200.17 | 4151.15 |  | 7058.87 | 13739.59 |
| 3460.56 | 10098.32 |  | 1148.9 | 2045.2 |  | 2652.39 | 10997.46 |
| 14984.71 | 9237.08 |  | 3279.75 | 4486.51 |  | 2543.72 | 4395.53 |
| 2493.97 | 3971 |  | 2146.45 | 3731.09 |  | 6950.51 | 8070.06 |
| 15459.72 | 9040.38 |  | 2269.23 | 2839.04 |  | 13714.85 | 13124.21 |
| 4630.68 | 3317.17 |  | 2308.59 | 3142.61 |  | 4143.85 | 6955.5 |
| 907.7 | 5245.58 |  | 3961.06 | 4633.24 |  | 8134.04 | 12191.85 |
| 2478.44 | 3236.67 |  | 2072.76 | 2701.38 |  | 9547.63 | 14033.67 |
| 9956.02 | 11796.86 |  | 2310.16 | 2576.17 |  | 2883.2 | 4062.49 |
| 2417.56 | 4411.17 |  | 1830.32 | 1427.35 |  | 14016.94 | 15023.07 |
| 7299.56 | 9917.84 |  | 2130.35 | 2968.62 |  | 7885.8 | 6039.21 |
| 4497.8 | 5467.01 |  | 4372.64 | 8526.83 |  | 7530.42 | 7519.74 |
| 8703.69 | 3223.86 |  | 4909.6 | 6466.02 |  | 2520.49 | 3412.43 |
| 9669.22 | 9449.981 |  | 1889.55 | 2424.89 |  | 4627.69 | 3903.73 |
| 6107.98 | 5694.14 |  | 407.63 | 1166.5 |  | 12143.03 | 12817.3 |
| 3317.58 | 2995.25 |  | 4023.62 | 4239.39 |  | 6267.88 | 6427.79 |
| 2942.9 | 1431.76 |  | 3522.32 | 3571.7 |  | 3557.94 | 5772.82 |
| 4851.99 | 3831.4 |  | 1000.55 | 1188.31 |  | 9888.06 | 8015.26 |
| 2518.37 | 4821.55 |  | 1489.56 | 1652.64 |  | 9289.68 | 12102.76 |
| 5559.73 | 5498.02 |  | 1426.8 | 3284 |  | 8158.65 | 5945.31 |
| 7679.97 | 6562.76 |  | 3319.94 | 4164.54 |  | 13854.66 | 12152.39 |
| 4242.68 | 4335.59 |  | 1696.66 | 2727.58 |  | 10561.19 | 7475.19 |
| 5519.98 | 4429.21 |  | 1932.85 | 3379.09 |  | 8365.44 | 6674 |
| 10415.67 | 5845.88 |  | 2820.15 | 3294.69 |  | 11354.73 | 15218.35 |
| 11942.38 | 8819.35 |  | 1936.45 | 1405.71 |  | 11923.88 | 18911.8 |
| 13056.68 | 9720.981 |  | 647.55 | 1473.74 |  | 11660.66 | 14592.17 |
| 4101.08 | 8176.84 |  | 919.66 | 1630.18 |  | 2436.71 | 5713.61 |
| 3962.01 | 4913.61 |  | 1315.61 | 1726.55 |  | 10620.09 | 8802.93 |
| 4934.81 | 7868.44 |  | 394.8 | 1221.02 |  | 7735.11 | 4100.54 |
| 4756.06 | 5948.36 |  | 4048.86 | 5971.55 |  | 6927.37 | 9466.93 |
| 11938.37 | 6973.57 |  | 1702.48 | 2362.31 |  | 4876.04 | 5352.68 |
| 3602.44 | 3254.69 |  | 3485.81 | 4194.38 |  | 9398.05 | 9462.67 |
| 6584.3 | 5916.21 |  | 3117.31 | 3915.31 |  | 5011.07 | 4786.74 |
| 5010.63 | 7797.22 |  | 3276.97 | 3447.49 |  | 10990.26 | 5109.27 |
| 4813.04 | 6263.98 |  | 3523.69 | 6113.32 |  | 17696.05 | 15594.34 |
| 3032.84 | 5127.41 |  | 3203.39 | 2302.78 |  | 10059.05 | 7486.28 |
| 9625.44 | 10382.5 |  | 1726.84 | 2279.33 |  | 12445.99 | 9419.32 |
| 19870.16 | 11904.77 |  | 4538.82 | 3692.73 |  | 12277.55 | 7112.55 |
| 1571.88 | 4099.23 |  | 2179.29 | 2825.6 |  | 4079.48 | 12442.63 |
| 5742.37 | 3189.64 |  | 1489.37 | 2657.5 |  | 5338.22 | 7491.27 |
| 6253.17 | 7436.38 |  | 2573.86 | 2708.89 |  | 7875.52 | 7488.78 |
| 2121.99 | 3043.74 |  | 1870.47 | 3237.43 |  | 3853.75 | 8635.54 |
| 5334 | 3803.02 |  | 1792.72 | 3069.58 |  | 14867.46 | 11574.59 |
| 3742.81 | 3213.58 |  | 610.26 | 1102.37 |  | 5265.22 | 7115.7 |
| 16637.75 | 10920.46 |  | 1221.16 | 2120.39 |  | 2385.6 | 13496.15 |
| 9365.51 | 7121.23 |  | 2407.36 | 3014.18 |  | 3350.81 | 10814.92 |
| 18671.4 | 11527.45 |  | 2251.82 | 3374.65 |  | 2282.76 | 6021.32 |
| 3834.56 | 5003.12 |  | 2152.22 | 2160.14 |  | 1272.98 | 5228.23 |
| 2793.66 | 3729.6 |  | 3445.14 | 3672.15 |  | 2275.37 | 5768.55 |
| 3032.58 | 5028.36 |  | 2134.21 | 3525.7 |  | 5809.25 | 6346.63 |
| 9354.17 | 8353.87 |  | 3532.6 | 2184.67 |  | 2608.16 | 6855.63 |
| 2674.14 | 4067.12 |  | 844.72 | 1135.86 |  | 1851.68 | 6802.33 |
| 7850.35 | 5022.02 |  | 3208.66 | 5030.11 |  | 2869.02 | 15213.79 |
| 3357.4 | 3599.58 |  | 2491.03 | 2377.2 |  | 2351.52 | 6546.28 |
| 5184.31 | 2359.23 |  | 4692 | 6759.77 |  | 2712.65 | 6233.58 |
| 5409.81 | 3840.25 |  | 2555.93 | 3443.98 |  | 4290.12 | 8539.49 |
| 3071.02 | 2358.14 |  | 2288.5 | 5131.84 |  | 1962.46 | 5641.53 |
| 3374.9 | 1938.2 |  | 2278.43 | 4011.05 |  | 928.85 | 5800.25 |
| 2160.35 | 1464.56 |  | 4745.86 | 3177.81 |  | 1286.71 | 5662.74 |
| 9155.67 | 6425.79 |  | 1883.34 | 3605.32 |  | 4955.52 | 11118.11 |
| 3149.37 | 3903.72 |  | 2036.88 | 2068.34 |  | 5446.85 | 7259.9 |
| 1820.66 | 2022.84 |  | 2007.58 | 4138.84 |  | 2403.57 | 6779.36 |
| 2436 | 1406.91 |  | 1184.54 | 1470 |  | 2718.18 | 11394.68 |
| 3555.69 | 2491.29 |  | 2479.36 | 2493.77 |  | 2099.39 | 10025.61 |
| 1693.86 | 1941.35 |  | 707.15 | 897.63 |  | 1090.92 | 5702.47 |
| 6113.66 | 5354.61 |  | 2599.44 | 4210.65 |  | 1896.22 | 6789.75 |
| 1213.97 | 1792.77 |  | 1000.25 | 1797.44 |  | 1642.94 | 3831.86 |
| 1756.42 | 2669.48 |  | 2687.78 | 6025.64 |  | 1228.86 | 8583.92 |
| 1476.61 | 1815.04 |  | 1433.35 | 1545.49 |  | 2059.64 | 7684.9 |
| 3067.26 | 2825.6 |  | 1856.65 | 2388.05 |  | 3735.65 | 10594.81 |
| 5001.62 | 1800.68 |  | 771.72 | 1589.66 |  | 1313.63 | 5724.19 |
| 4499.13 | 2735.55 |  | 1614.99 | 2307.55 |  | 1402.03 | 6842.52 |
| 3345.33 | 1980.5 |  | 2682.22 | 3790.86 |  | 441.05 | 5008.79 |
| 2209.76 | 1591.63 |  | 1102.15 | 984.26 |  | 5886.78 | 12278.78 |
| 7241.46 | 5576.1 |  | 3848.43 | 3344.68 |  | 6749.21 | 9215.18 |
| 3338.95 | 2856.64 |  | 4011.23 | 6167.16 |  | 7007.69 | 12208.58 |
| 2213.74 | 2523.26 |  | 2264.95 | 6999.11 |  | 5636.06 | 10393.89 |
| 2488.02 | 1829.22 |  | 1083.34 | 3931.29 |  | 5648 | 6982.71 |
| 3416 | 1942.37 |  | 1855.06 | 2586.53 |  | 7065.16 | 12521.17 |
| 1822.03 | 2744.38 |  | 3294.57 | 4454.24 |  | 11932.9 | 16418.74 |
| 2069.91 | 2239.77 |  | 4977.35 | 6666.38 |  | 2477.66 | 7057.61 |
| 3215.84 | 3195.74 |  | 1674.75 | 2856.41 |  | 7642.29 | 10858.03 |
| 4935.91 | 3284.11 |  | 1013.22 | 1797.7 |  | 3082.1 | 8651.05 |
| 3919.94 | 2902.18 |  | 3373.42 | 2994.83 |  | 11135.31 | 23403.7 |
| 1810.86 | 1994.84 |  | 632.36 | 2087.3 |  | 3863.18 | 12227.52 |
| 2324.45 | 1434.9 |  | 1473.43 | 2556.09 |  | 4170.57 | 9769.89 |
| 1370.12 | 1475.74 |  | 2616.28 | 3410.32 |  | 8403.54 | 15947.29 |
| 2061.99 | 1697.63 |  | 830.22 | 846.88 |  | 3647.95 | 6094.31 |
| 1403.15 | 888.23 |  | 3057.52 | 2626.74 |  | 3797.5 | 8726.31 |
| 4003.79 | 3065.82 |  | 3402.4 | 3895.73 |  | 3624.61 | 7981.09 |
| 1814.09 | 2546.96 |  | 1979.32 | 1479.61 |  | 3629.95 | 7302.98 |
| 2008.39 | 2972.27 |  | 2978.57 | 3723.69 |  | 2211.03 | 8043.52 |
| 2282.41 | 2625.37 |  | 1498.52 | 1418.23 |  | 5178.07 | 16106.34 |
| 1605.44 | 3985.85 |  | 1504.85 | 2499.2 |  | 3636.62 | 9528.231 |
| 3025.98 | 2482.27 |  | 1734.42 | 2064.89 |  | 1859.22 | 4260.81 |
| 2885.03 | 2266.1 |  | 615.19 | 1851.32 |  | 3411.19 | 7875.26 |
| 2298.19 | 3680.79 |  | 3988.46 | 5250.39 |  | 4868.17 | 9047.64 |
| 3896.51 | 2200.96 |  | 1888.85 | 1865.13 |  | 2248.28 | 8390.89 |
| 3558.64 | 1701.17 |  | 571.48 | 891.97 |  | 3603.37 | 8684.481 |
| 4617.12 | 3185.25 |  | 384.1 | 764.83 |  | 12993.27 | 15202.33 |
| 1538.93 | 2361.52 |  | 1994.29 | 3813.58 |  | 5362.56 | 9332.731 |
| 2829.6 | 3096.3 |  | 531.23 | 1257.69 |  | 4744.34 | 14396.75 |
| 2338.23 | 2141.27 |  | 3413.94 | 3394.38 |  | 4443.66 | 11785.09 |
| 8124.97 | 3731.32 |  | 1653.73 | 2309.92 |  | 3750.38 | 9639.09 |
| 5375.23 | 4304.96 |  | 1852.67 | 2485.62 |  | 3549.86 | 7600.82 |
| 2998.32 | 3474.76 |  | 2901.89 | 2307.52 |  | 5081.44 | 12691.7 |
| 1437.93 | 2429.9 |  | 1588 | 3579.6 |  | 3667.86 | 10588.07 |
| 2011.37 | 2551.26 |  | 1884.24 | 3244.34 |  | 1205.74 | 3893.89 |
| 3322.12 | 3362.29 |  | 1543 | 2151.68 |  | 8168.91 | 9802.16 |
| 2538.06 | 1729.32 |  | 642.86 | 1515.83 |  | 4880.59 | 10097.29 |
| 3241.45 | 2148.15 |  | 2223.09 | 3577.06 |  | 3324.49 | 7926.73 |
| 2731.53 | 2084.1 |  | 3831.54 | 4395.72 |  | 884.9 | 2803.2 |
| 5447.48 | 3624.6 |  | 1505.11 | 1338.57 |  | 2407.68 | 7684.9 |
| 4099.31 | 2469.75 |  | 2028.2 | 2384.05 |  | 3656.89 | 11595.65 |
| 2030.04 | 2065.39 |  | 1477.14 | 1938.37 |  | 3258.31 | 7948.6 |
| 1855.84 | 2583.88 |  | 2609.26 | 2917.43 |  | 6888.41 | 3558.41 |
| 3963.42 | 1552.77 |  | 933.68 | 1653.47 |  | 6299.15 | 17017.33 |
| 2330.15 | 1575.4 |  | 4057.85 | 3792.19 |  | 1585.9 | 9490.38 |
| 5950.36 | 3091.08 |  | 1373.25 | 1555.9 |  | 2007.13 | 10937.46 |
| 5568.14 | 2868.9 |  | 584.84 | 794.13 |  | 5397.08 | 9246.27 |
| 3423.36 | 3069.15 |  | 771.48 | 3971.4 |  | 727.96 | 7909.48 |
| 2250.13 | 4749.19 |  | 3778.12 | 3652.45 |  | 1957.5 | 4641.9 |
| 2300.41 | 2850.76 |  | 2484.03 | 3439.7 |  | 1166.06 | 3424.92 |
| 2801.01 | 2444.82 |  | 2659.93 | 3693.56 |  | 3539.19 | 4053.58 |
| 3399.44 | 3742.02 |  | 1352.74 | 1892.63 |  | 8103.41 | 7193.07 |
| 5157.63 | 3148.61 |  | 2384.47 | 3853.87 |  | 3770.15 | 9594.86 |
| 4547.06 | 5215.78 |  | 2485.39 | 2509.17 |  | 3422.65 | 5111.84 |
| 1593.31 | 2412.66 |  | 1540.71 | 1884.78 |  | 5155.46 | 3035.27 |
| 1862.32 | 2214.86 |  | 2610.21 | 1931.26 |  | 1584.38 | 7637.78 |
| 3806.34 | 2674.75 |  | 2667.57 | 2746.53 |  | 5437.95 | 7176.96 |
| 4572.72 | 3419.78 |  | 1627.79 | 1168.58 |  | 1031.61 | 3238.25 |
| 3659.54 | 3381.76 |  | 1686.45 | 2392.95 |  | 18800.67 | 13577.96 |
| 2286.1 | 2225.81 |  | 2211.3 | 3815.7 |  | 4925.4 | 8182.89 |
| 2314.75 | 3983.47 |  | 1562.03 | 2673.04 |  | 12425.86 | 12425.86 |
| 2472.39 | 1845.25 |  | 1032.8 | 1755.11 |  | 24040.11 | 24040.11 |
| 3304.2 | 1663.31 |  | 432.62 | 1171.04 |  | 16535.71 | 9732.02 |
| 5273.3 | 2785.03 |  | 1948.68 | 1451.74 |  | 18442.55 | 18442.55 |
| 3181.6 | 2420.73 |  | 6823.26 | 4000 |  | 1423.29 | 7665.6 |
| 1319.05 | 2734.96 |  | 4920.89 | 6133.11 |  | 4556.29 | 4920.14 |
| 2828.92 | 2084.45 |  | 4446.93 | 3353.5 |  | 5021.07 | 2358.08 |
| 2619.23 | 1554.87 |  | 4761.84 | 5293.35 |  | 3118.75 | 9712.01 |
| 4890.1 | 5965.12 |  | 8562.31 | 3620.86 |  | 5712.03 | 7523.57 |
| 4909.93 | 2672.15 |  | 4064.6 | 2238.09 |  | 1860.01 | 6186.72 |
| 17747.98 | 2797.78 |  | 4890.91 | 3506.5 |  | 8657.4 | 8985.231 |
| 2172.74 | 2147.55 |  | 13614.53 | 6052.88 |  | 6434.26 | 4055.16 |
| 3410.71 | 3949.36 |  | 9287.89 | 5118.6 |  | 5864.42 | 8582.99 |
| 3689.77 | 2711.45 |  | 5884.1 | 5223.13 |  | 10072.19 | 13331.06 |
| 2882.3 | 3148.36 |  | 7952.44 | 7173.85 |  | 13601.12 | 16079.15 |
| 2428.5 | 1771.85 |  | 6169.56 | 3038.68 |  | 5124.69 | 7442.85 |
| 1131.35 | 2022.48 |  | 6212.75 | 4813.27 |  | 5477.91 | 5194.53 |
| 2205.2 | 3531.98 |  | 9172.22 | 5251.8 |  | 22203.04 | 14141.64 |
| 918.18 | 1199.41 |  | 3505.52 | 5019.76 |  | 34548.37 | 15228.98 |
| 2936.45 | 5739.58 |  | 4283.4 | 2170.11 |  | 2562.66 | 6966.12 |
| 2283.03 | 2434.78 |  | 4483.85 | 2219.01 |  | 3795.64 | 3278.42 |
| 2249.39 | 2785.03 |  | 2488.12 | 2855.48 |  | 2092.86 | 7988.97 |
| 1934.86 | 2813.88 |  | 8153.61 | 2142.43 |  | 11997.34 | 6085.95 |
| 2399.37 | 2455.52 |  | 3726.76 | 4141.67 |  | 6273.59 | 8000 |
| 1388.5 | 1791.33 |  | 4948.03 | 3625.73 |  | 3221.82 | 4445.44 |
| 1497.76 | 2331.87 |  | 3364.52 | 1904.03 |  | 19.77 | 5177.07 |
| 1279.4 | 2376.84 |  | 3119.1 | 2326.1 |  | 5800.98 | 4256.3 |
| 5122.18 | 4517.97 |  | 6433.09 | 5336.82 |  | 2775.72 | 10929.19 |
| 2326.54 | 3285.38 |  | 5831.03 | 5135.08 |  | 4877.42 | 6042.69 |
| 4524.68 | 2518.25 |  | 9358.28 | 6473.07 |  | 2222.53 | 5095.69 |
| 3481.65 | 2678.48 |  | 2586.28 | 3137.8 |  | 1526.44 | 9246.66 |
| 3513.12 | 2355.39 |  | 6198.23 | 3551.28 |  | 4622.1 | 7517.09 |
| 6371.97 | 3903.2 |  | 3240.12 | 2486.14 |  | 1769.72 | 3901.35 |
| 2094.16 | 2765.03 |  | 5142.1 | 4780.86 |  | 1268.87 | 4297.76 |
| 4398.95 | 3536.89 |  | 4433.42 | 5235.52 |  | 1925.21 | 5174 |
| 3011.28 | 2750.05 |  | 8962.76 | 4568.68 |  | 2890.65 | 6286.62 |
| 3756.95 | 4692.11 |  | 5078.94 | 3093.2 |  | 1411.41 | 7972.57 |
| 1660.25 | 2674.09 |  | 4000 | 3008.38 |  | 1963.63 | 13156.14 |
| 1440.81 | 1355.85 |  | 1718.97 | 1800.86 |  | 4084.48 | 9764.21 |
| 2677.33 | 3592.28 |  | 3308.67 | 1826.78 |  | 2828.97 | 5685.33 |
| 1483.69 | 2572.92 |  | 6839.77 | 3622.21 |  | 1255.43 | 6327.8 |
| 6835.66 | 5875.82 |  | 2527.04 | 2825.65 |  | 2602.52 | 10314.76 |
| 1496.65 | 2550.17 |  | 3023.8 | 5030.64 |  | 2035.75 | 4551.06 |
| 2067.28 | 3022.99 |  | 5980.01 | 10638.27 |  | 3170.05 | 13290.94 |
| 5060.12 | 3406.81 |  | 2305.68 | 6779.05 |  | 4500 | 10866.48 |
| 3179.17 | 3167.04 |  | 9148.26 | 4284.67 |  | 2000 | 10000 |
| 6042.85 | 4738.49 |  | 10030.55 | 4256.3 |  | 2000 | 5411.97 |
| 3766.56 | 3624.42 |  | 2902.2 | 3204.23 |  | 5000 | 14526.42 |
| 1443.21 | 2468.48 |  | 14981.96 | 11004.05 |  | 4243.09 | 18871.32 |
| 2150.16 | 1973.64 |  | 6000 | 12818.18 |  | 2500 | 8876.07 |
| 2378.48 | 2842.57 |  | 12048.83 | 5160.51 |  | 2100 | 16000 |
| 2025.24 | 2238.07 |  | 4353.67 | 8821.07 |  | 2345.5 | 7825.44 |
| 7993.21 | 4275.99 |  | 3677.49 | 7605.45 |  | 2000 | 9891.22 |
| 2795.09 | 4695.91 |  | 1126.55 | 2082.27 |  | 1991.74 | 9684.37 |
| 6208.9 | 4451.13 |  | 2388.79 | 3537.98 |  | 2000 | 18433.19 |
| 1166.61 | 1311.14 |  | 1601.51 | 4386.83 |  | 2056.42 | 13089.83 |
| 4826.52 | 3430.79 |  | 641.28 | 1615.14 |  | 703.33 | 2454.61 |
| 2422.9 | 2766.82 |  | 4283 | 4428.66 |  | 1897.11 | 17394.25 |
| 3335.83 | 3057.54 |  | 5210.85 | 2654.57 |  | 1210.54 | 2965.06 |
| 1824.29 | 2746.12 |  | 3330.71 | 2956.37 |  | 1440.2 | 16254.6 |
| 3271.82 | 2036.37 |  | 5043.16 | 5133.52 |  | 3186.72 | 9735.46 |
| 2282.21 | 1619.5 |  | 4562.88 | 4318.69 |  | 779.55 | 2744.61 |
| 4689.7 | 2957.25 |  | 1346.76 | 2566.58 |  | 6967.1 | 19462.03 |
| 2419.35 | 1778.07 |  | 4293.2 | 6175.68 |  | 9362.16 | 8787.69 |
| 2866.98 | 3156.07 |  | 7179.33 | 6947.41 |  | 5838 | 7364.1 |
| 3394.95 | 2161.15 |  | 6065.18 | 4931.91 |  | 6000 | 7000 |
| 2975.78 | 1509.95 |  | 3865.62 | 8870.13 |  | 6267.33 | 5497.45 |
| 2657.67 | 1546.41 |  | 3369.86 | 3260.87 |  | 13314.69 | 8688.08 |
| 7641.13 | 5922.73 |  | 1349.99 | 1388.6 |  | 2000 | 5500 |
| 6288.28 | 7613.21 |  | 10634.67 | 9159.77 |  | 2100 | 4500 |
| 7862.78 | 6508.05 |  | 5142.45 | 2605.39 |  | 16183.36 | 13109.12 |
| 8718.44 | 11206.05 |  | 9101.7 | 8147.58 |  | 10778.71 | 17810.18 |
| 9441.53 | 4927.11 |  | 2506.17 | 2485.38 |  | 9948.25 | 8000 |
| 4834.84 | 5134.09 |  | 8142.25 | 4607.4 |  | 21703.45 | 24571.29 |
| 4505.8 | 3992.84 |  | 3077.22 | 3324.47 |  | 8882.45 | 8882 |
| 2713.14 | 2558.44 |  | 2752.08 | 4487.05 |  | 10662.09 | 8509.66 |
| 1782.02 | 4065.48 |  | 1018.6 | 2294.27 |  | 9000 | 15518.22 |
| 2065.94 | 2966.81 |  | 13727.99 | 7430.22 |  | 5530.87 | 9965.65 |
| 3029.81 | 2770.05 |  | 21295.67 | 11295.12 |  | 11000 | 15781.31 |
| 3442.42 | 2011.46 |  | 8657.3 | 4070.84 |  | 6676.76 | 7164.29 |
| 4141.01 | 3932.55 |  | 564.68 | 2150.37 |  | 6000 | 7000 |
| 6586.32 | 5923.15 |  | 4640.49 | 1792.26 |  | 9627.45 | 8830.27 |
| 2019.25 | 2130.72 |  | 6800 | 7024.19 |  | 6000 | 9367.63 |
| 6464.15 | 5925.58 |  | 4405.55 | 3795.15 |  | 14000 | 14000 |
| 1490.3 | 2640.39 |  | 3178.78 | 3632.66 |  | 19434.78 | 10007.67 |
| 12266.13 | 7926.17 |  | 1541.9 | 3579.51 |  | 9000 | 7799.28 |
| 13279.97 | 6773.69 |  | 4022.34 | 4502.71 |  | 4362.81 | 8000 |
| 18852.37 | 14388.75 |  | 1104.65 | 4902.15 |  | 5912.83 | 10251.93 |
| 4339.11 | 3809.44 |  | 2827.18 | 1374.05 |  | 4000 | 3950.92 |
| 4972.26 | 4129.77 |  | 7292.02 | 4913.04 |  | 4654.7 | 9053.02 |
| 10208.35 | 9729.2 |  | 5316.76 | 3417.77 |  | 8369.5 | 13916.34 |
| 11929.6 | 3277.86 |  | 1922.03 | 3784.16 |  | 5323.36 | 3004.12 |
| 11185.33 | 7922.21 |  | 8933.19 | 9877.56 |  | 3103.81 | 18408.38 |
| 22716.66 | 14418.23 |  | 2691.48 | 3959.19 |  | 1908.98 | 5000 |
| 16006.1 | 8189.51 |  | 5490.73 | 5384.62 |  | 2000 | 5321.57 |
| 18066.76 | 13417.02 |  | 1801.42 | 2252.75 |  | 2757.77 | 2000 |
| 1487.69 | 1370.14 |  | 2792.85 | 2910.01 |  | 3023.41 | 9000 |
| 5217.97 | 3950.7 |  | 4857.1 | 3921.57 |  | 3415.21 | 17815.12 |
| 22366.94 | 12350.87 |  | 8265.05 | 4220.56 |  | 1857.15 | 8369.72 |
| 4159.85 | 2435.3 |  | 3500 | 1365.91 |  | 4134.41 | 20953.39 |
| 7402.22 | 9313.3 |  | 2645.6 | 1610.23 |  | 900 | 3561.42 |
| 28708.75 | 16078.39 |  | 3432.46 | 2047.23 |  | 2735.2 | 13092.58 |
| 16178.09 | 9781.18 |  | 4413.29 | 2380.94 |  | 2004.51 | 11808.42 |
| 7836.11 | 6893.67 |  | 1949.42 | 1234.6 |  | 771.91 | 3663.6 |
| 2477.83 | 4085.13 |  | 7138.41 | 3662.24 |  | 8876.83 | 15274.81 |
| 17936.27 | 10055.63 |  | 3702.95 | 2053.35 |  | 1594.09 | 2877.51 |
| 8771.37 | 5609.46 |  | 1453.79 | 885.31 |  | 2974.74 | 13655.8 |
| 3696.38 | 6569.79 |  | 1287.58 | 1872.11 |  | 5657.3 | 12265.3 |
| 4832.4 | 6231.36 |  | 2117.22 | 2030.14 |  | 823.62 | 4259.15 |
| 1839.28 | 4607.04 |  | 4507.37 | 2955.01 |  | 5000 | 20000 |
| 14285.39 | 8332.91 |  | 1391.5 | 2415.99 |  | 9000 | 10145.31 |
| 18895.87 | 12139.46 |  | 3484.31 | 697.35 |  | 4000 | 5196.27 |
| 3383.45 | 5173 |  | 5468.75 | 2344.34 |  | 9400.05 | 10457.05 |
| 5453.28 | 4047.53 |  | 8576.82 | 4770.58 |  | 4493.07 | 7270.39 |
| 11563.38 | 7181.28 |  | 3327.15 | 4405.63 |  | 8433.62 | 7018.92 |
| 11803.64 | 8192.85 |  | 800.55 | 783.04 |  | 5162.84 | 7060.72 |
| 6433.6 | 6585.12 |  | 5000 | 3438.49 |  | 2526.26 | 7845.21 |
| 9940.36 | 11078.73 |  | 1208.08 | 2202.05 |  | 2018.39 | 1500 |
| 12174.07 | 6366.53 |  | 2502.88 | 1740.31 |  | 6000 | 12000 |
| 9716.03 | 8749.27 |  | 5291.66 | 2851.59 |  | 3417.35 | 9096.58 |
| 23598.86 | 13183.92 |  | 6303.06 | 4533.42 |  | 12000 | 12043.52 |
| 11637.9 | 12998.48 |  | 2519.82 | 3311.1 |  | 11360.72 | 16173.91 |
| 3260.21 | 4937.91 |  | 4623.52 | 2883.3 |  | 4500 | 5069.79 |
| 18011.42 | 9445.79 |  | 1382.14 | 3474.85 |  | 13399.56 | 11345.31 |
| 4654.13 | 6751.38 |  | 2479.07 | 2288.55 |  | 13112.45 | 13867.91 |
| 2234.17 | 3030 |  | 2139.91 | 2803.67 |  | 13602.26 | 5443.33 |
| 9761.11 | 8232.2 |  | 2562.52 | 4017.71 |  | 4666.82 | 4581.09 |
| 13266.27 | 7914.96 |  | 3609.87 | 1952.63 |  | 5612.53 | 8416.92 |
| 12671.64 | 11453.66 |  | 2599.85 | 6154.79 |  | 15059.17 | 11889.96 |
| 17834.53 | 14426.25 |  | 3967.65 | 5287 |  | 5239.36 | 6200 |
| 20525.38 | 10070.41 |  | 2324.31 | 2574.01 |  | 13500 | 14000 |
| 9606.31 | 9037.85 |  | 7825.67 | 1389.52 |  | 16000 | 9000 |
| 18893.08 | 13574.93 |  | 2592.67 | 321.89 |  | 6000 | 6000 |
| 4653.56 | 4286.68 |  | 5346.99 | 5805.03 |  | 4152.49 | 10309.92 |
| 9511.39 | 5618.47 |  | 3595.21 | 4648.71 |  | 9153.02 | 7986.74 |
| 4652.26 | 6937.23 |  | 2733.49 | 2421.77 |  | 5812.64 | 7707.87 |
| 9353.32 | 5550.68 |  | 3370.79 | 4425.39 |  | 10051.93 | 5913.26 |
| 3682.35 | 3502.49 |  | 2397.11 | 1768.79 |  |  |  |
| 16385.32 | 9875.84 |  | 4410.57 | 3175.76 |  |  |  |
| 5975.41 | 5065.03 |  | 412.82 | 1655.25 |  |  |  |
| 12116.63 | 7329.04 |  | 875.32 | 2391.22 |  |  |  |
| 13442.84 | 7991.56 |  | 5041.02 | 2919.18 |  |  |  |
| 18360.16 | 9371.4 |  | 533.63 | 1302.41 |  |  |  |
| 12104.69 | 7585.12 |  | 479.68 | 1322.36 |  |  |  |
| 5109.16 | 7098.57 |  | 2335.91 | 1558.75 |  |  |  |
| 12750.92 | 9982.16 |  | 605.9 | 2235.42 |  |  |  |
| 1834.34 | 4545.75 |  | 4069.19 | 6688.45 |  |  |  |
| 10524.07 | 1842.76 |  | 2754.1 | 568.11 |  |  |  |
| 2584.11 | 5039.83 |  | 384.88 | 2104.31 |  |  |  |
| 3119.91 | 3992.12 |  | 2371.14 | 1370.98 |  |  |  |
| 4109.26 | 5204.22 |  | 3878 | 2623.3 |  |  |  |
| 5510.69 | 5941.57 |  | 1813.95 | 1762.95 |  |  |  |
| 2292.84 | 3814.95 |  | 6569.09 | 3000 |  |  |  |
| 6500.43 | 4526.06 |  | 6995.71 | 5000 |  |  |  |
| 3733.55 | 18177.37 |  | 3927.96 | 5051.7 |  |  |  |
| 18079.06 | 10410.32 |  | 2724.45 | 3025.48 |  |  |  |
| 25719.75 | 8452.59 |  | 5162.84 | 4081.76 |  |  |  |
| 9793.55 | 5920.83 |  | 2000 | 2650.93 |  |  |  |
| 20094.81 | 17201.56 |  | 1898.48 | 3790.38 |  |  |  |
| 17340.25 | 10228.75 |  | 6500 | 2816.2 |  |  |  |
| 1306.21 | 2573.89 |  | 2000 | 2597.39 |  |  |  |
| 2134.81 | 2382.52 |  | 1804.02 | 2915.99 |  |  |  |
| 2906.96 | 3822.14 |  | 16000 | 6099.03 |  |  |  |
| 23037.4 | 9064.94 |  | 10613.49 | 6727.62 |  |  |  |
| 10690.15 | 10315.04 |  | 2379.14 | 3361.76 |  |  |  |
| 7804.08 | 20702.79 |  | 15787.72 | 13000 |  |  |  |
| 10644.46 | 16275.85 |  | 7300 | 5500 |  |  |  |
| 10302.91 | 3362.89 |  | 3000 | 3834.87 |  |  |  |
| 8062.29 | 4765.84 |  | 1945.06 | 1107.21 |  |  |  |
| 2533.23 | 4779.62 |  | 8000 | 3000 |  |  |  |
| 3472.66 | 3186.93 |  | 6328.75 | 4486.11 |  |  |  |
| 3938.43 | 5056.97 |  | 9260.39 | 7655.09 |  |  |  |
| 4343.87 | 3342.43 |  | 3537.16 | 7170.14 |  |  |  |
| 8036.07 | 6327.76 |  | 8816.88 | 6084.61 |  |  |  |
| 6792.6 | 5288.62 |  | 4000 | 1700 |  |  |  |
| 3620.52 | 3894.32 |  | 9041.81 | 5992.48 |  |  |  |
| 10613.32 | 5239.84 |  | 20000 | 13000 |  |  |  |
| 8977.32 | 10145.18 |  | 4548.19 | 4991.15 |  |  |  |
| 2564.11 | 8380.45 |  | 11462.66 | 6711.96 |  |  |  |
| 4695.58 | 5611.88 |  | 6313.55 | 5735.18 |  |  |  |
| 5497.59 | 7219.52 |  | 10000 | 6000 |  |  |  |
| 7230.84 | 4750.23 |  | 5358.8 | 2000 |  |  |  |
| 3948.01 | 3549.48 |  | 12699.85 | 7900 |  |  |  |
| 692.81 | 8707.97 |  | 2500 | 3578.11 |  |  |  |
| 9650 | 5999.77 |  | 12682.97 | 10511.14 |  |  |  |
| 6138.19 | 3911.66 |  | 7517.22 | 3500 |  |  |  |
| 1631.91 | 3985.97 |  | 5000 | 4332.62 |  |  |  |
| 1987.43 | 1864.37 |  | 5778.64 | 4822.28 |  |  |  |
| 4067.44 | 3890.76 |  | 4800 | 5739.03 |  |  |  |
| 3226.51 | 2204.5 |  | 5594.85 | 2602.86 |  |  |  |
| 2782.12 | 3998.75 |  | 7569.44 | 4127.02 |  |  |  |
| 3450.92 | 1971.73 |  | 4800 | 2272.53 |  |  |  |
| 11292.95 | 3614.64 |  | 2128.89 | 944.19 |  |  |  |
| 3585.6 | 7265.46 |  | 6622.97 | 3479.86 |  |  |  |
| 8231.39 | 7866.77 |  | 2495.13 | 1630.51 |  |  |  |
| 9892.39 | 9091.86 |  | 2168.37 | 2329.57 |  |  |  |
| 3176.75 | 5542.76 |  | 2000 | 2000 |  |  |  |
| 2160.56 | 4419.86 |  | 1043.15 | 1714.72 |  |  |  |
| 4275.1 | 3694.54 |  | 8261.18 | 5680.73 |  |  |  |
| 6505.58 | 4548.46 |  | 3181.86 | 7260.44 |  |  |  |
| 1948.33 | 4443.72 |  | 8096.09 | 4250.07 |  |  |  |
| 4454.85 | 3642.33 |  | 2840.8 | 2203.19 |  |  |  |
| 2396.76 | 4122.62 |  | 6000 | 5514.34 |  |  |  |
| 3623.13 | 3260.53 |  | 3534.18 | 2096.49 |  |  |  |
| 2263.91 | 2393.19 |  | 5450.4 | 2786.57 |  |  |  |
| 2197.09 | 2461.4 |  | 5405.15 | 1705.19 |  |  |  |
| 2592.9 | 1593.87 |  | 3816.65 | 4324.38 |  |  |  |
| 3233.67 | 2422.03 |  | 4300.43 | 3132.43 |  |  |  |
| 2590 | 2590.65 |  | 7246 | 4961.29 |  |  |  |
| 3471.77 | 2886.76 |  | 7485.71 | 2500 |  |  |  |
| 2929.38 | 5119.36 |  | 11000 | 4000 |  |  |  |
| 1825.79 | 2175.55 |  | 3716.98 | 2985.17 |  |  |  |
| 12636.77 | 13573.07 |  | 14000 | 14226.53 |  |  |  |
| 6479.61 | 8311.13 |  | 5772.14 | 5846.58 |  |  |  |
| 13314.21 | 2936.49 |  | 4227.99 | 2919.06 |  |  |  |
| 2818.67 | 1715.7 |  | 3050.87 | 2258.78 |  |  |  |
| 10183.26 | 8780.7 |  | 9508.43 | 6047.92 |  |  |  |
| 12698.11 | 6151.07 |  | 6000 | 3200 |  |  |  |
| 2398.01 | 3134.23 |  | 6407.57 | 4000 |  |  |  |
| 2078.55 | 2672.74 |  | 10413.1 | 4151.22 |  |  |  |
| 2012.22 | 3787.7 |  | 9000 | 4100 |  |  |  |
| 3562.23 | 4733.11 |  | 3592.22 | 2965.06 |  |  |  |
| 1937.32 | 3237.9 |  | 17000 | 13952.39 |  |  |  |
| 1802.4 | 2225.89 |  | 2190.79 | 7437.9 |  |  |  |
| 4321.08 | 3576.5 |  | 8896.6 | 4238 |  |  |  |
| 4713.18 | 2891.32 |  | 6000 | 5700 |  |  |  |
| 2871.03 | 5757.99 |  | 15960.86 | 9244.84 |  |  |  |
| 2010.83 | 2749.8 |  | 4000 | 3930.53 |  |  |  |
| 16314.99 | 15167.94 |  | 6177.39 | 7881.91 |  |  |  |
| 1701.87 | 3156.73 |  | 4929.98 | 4166.86 |  |  |  |
| 4375.24 | 8982.45 |  | 8100 | 2900 |  |  |  |
| 2510.19 | 4763.83 |  | 6725.8 | 4033.46 |  |  |  |
| 2876.33 | 2371.35 |  | 7712.44 | 6131.03 |  |  |  |
| 3925.3 | 8937.59 |  | 5497.12 | 1860.96 |  |  |  |
| 2252.56 | 3003.16 |  | 5581.73 | 2949.24 |  |  |  |
| 5330.29 | 3500 |  | 6000 | 2900 |  |  |  |
| 2008.61 | 1488.53 |  | 4838.15 | 2000 |  |  |  |
| 2560.01 | 3875.98 |  | 1744.43 | 1731.59 |  |  |  |
| 2270.01 | 2051.69 |  | 7445.25 | 3311.83 |  |  |  |
| 2643.38 | 3488.13 |  | 2613.88 | 1997.94 |  |  |  |
| 3487.13 | 3171.94 |  | 1860.46 | 1581.56 |  |  |  |
| 3749.83 | 4105.95 |  | 3605.87 | 4261.83 |  |  |  |
| 8860.95 | 8168.72 |  | 1364.56 | 1477.07 |  |  |  |
| 3358.45 | 6096.49 |  | 5960.96 | 2115.46 |  |  |  |
| 6045.94 | 5281.62 |  | 5811.36 | 8951.35 |  |  |  |
| 9900.85 | 3965.68 |  | 8437.1 | 2537.96 |  |  |  |
| 6968.38 | 3421.25 |  | 8211.981 | 5183.6 |  |  |  |
| 7605.68 | 10652 |  | 5236.6 | 3808.43 |  |  |  |
| 8208.31 | 3950.59 |  | 2073.3 | 4997.8 |  |  |  |
| 2022.07 | 5243.47 |  | 3961.21 | 3385.31 |  |  |  |
| 2213.37 | 6023.82 |  | 6726.53 | 2509.78 |  |  |  |
| 2969.01 | 12203.74 |  | 3360.45 | 1988.33 |  |  |  |
| 3707.03 | 3122.01 |  | 3520.48 | 2848.36 |  |  |  |
| 4169.91 | 1907.9 |  | 5144.09 | 2597.53 |  |  |  |
| 1409.83 | 1859.18 |  | 3439.02 | 3722.07 |  |  |  |
| 7000 | 4000 |  | 3865.05 | 7058.02 |  |  |  |
| 1180.64 | 2262 |  | 6755.8 | 9287.83 |  |  |  |
| 2652.74 | 2709.11 |  | 12064.07 | 5167.76 |  |  |  |
| 3381.19 | 2511.11 |  | 5723.35 | 8826.92 |  |  |  |
| 1452.46 | 2452.55 |  | 16306.79 | 4410.83 |  |  |  |
| 2770.14 | 2045.76 |  | 9354.02 | 6000 |  |  |  |
| 3154.16 | 1945.72 |  | 6483.33 | 4247.95 |  |  |  |
| 2789.94 | 3604.88 |  | 8711.981 | 7273.25 |  |  |  |
| 1894.23 | 1364.72 |  | 3661.49 | 3500 |  |  |  |
| 1300 | 1524.45 |  | 3446.59 | 2995.34 |  |  |  |
| 1831 | 1913.05 |  | 9078.26 | 7983.47 |  |  |  |
| 38139.89 | 2096.81 |  | 11558.25 | 7460.23 |  |  |  |
| 3113.2 | 1419.01 |  | 9878.87 | 10000 |  |  |  |
| 1900 | 2178.83 |  | 2430.93 | 3500 |  |  |  |
| 2926.52 | 3321.78 |  | 4254.12 | 2997.2 |  |  |  |
| 2528.65 | 2312.03 |  | 9038.82 | 3900 |  |  |  |
| 4093.86 | 1528.27 |  | 11853.25 | 8485.3 |  |  |  |
| 2472.76 | 2818.47 |  | 12984.35 | 4052.89 |  |  |  |
| 2233.85 | 1449.77 |  | 7848.56 | 3000 |  |  |  |
| 2000 | 3285.64 |  | 12420.58 | 2158.21 |  |  |  |
| 3055.24 | 3636.03 |  | 7812.47 | 2645.3 |  |  |  |
| 2260.68 | 1277.5 |  | 9467.08 | 3609.91 |  |  |  |
| 6000 | 3889.47 |  | 5208.35 | 2738.91 |  |  |  |
| 2968.04 | 2315.54 |  | 5177.94 | 3386.8 |  |  |  |
| 3579.71 | 2000 |  | 6285.59 | 3448.25 |  |  |  |
| 1916.13 | 1546.81 |  | 5165.98 | 530.34 |  |  |  |
| 3484.4 | 3667.29 |  | 4366.35 | 2500 |  |  |  |
| 1854.4 | 960.36 |  | 3500 | 2811.48 |  |  |  |
| 1696.9 | 2217.05 |  | 1220.94 | 712.63 |  |  |  |
| 3361.4 | 2213.07 |  | 2149.69 | 2152.01 |  |  |  |
| 6170.67 | 6321.94 |  | 1687.24 | 813.52 |  |  |  |
| 5235.19 | 5721.59 |  | 5960.96 | 2755.16 |  |  |  |
| 16881.24 | 11036.98 |  | 5811.36 | 3397.56 |  |  |  |
| 6278.25 | 4000 |  | 8437.1 | 2247.45 |  |  |  |
| 18849.1 | 5941.17 |  | 8211.981 | 1736.94 |  |  |  |
| 21144.92 | 10961.56 |  | 5236.6 | 1030.89 |  |  |  |
| 28535.97 | 15415.62 |  | 2073.3 | 3730.61 |  |  |  |
| 1929.57 | 3841.53 |  | 3961.21 | 2221.51 |  |  |  |
| 2501.36 | 2517.18 |  | 6726.53 | 2500 |  |  |  |
| 24789.92 | 17907.1 |  | 3360.45 | 2045.05 |  |  |  |
| 15015.9 | 8004.39 |  | 3520.48 | 2666.47 |  |  |  |
| 1585.03 | 6153.6 |  | 5144.09 | 2861.17 |  |  |  |
| 26655.82 | 13672.36 |  | 3439.02 | 7756.22 |  |  |  |
| 19026.93 | 14271.81 |  | 3865.05 | 3794.65 |  |  |  |
| 26326.12 | 10638.1 |  | 6755.8 | 13857.57 |  |  |  |
| 19977.23 | 10488.78 |  | 12064.07 | 7512.45 |  |  |  |
| 26609.67 | 6424.6 |  | 5723.35 | 3543.57 |  |  |  |
| 8594.31 | 6318.38 |  | 16306.79 | 11440.64 |  |  |  |
| 19962.07 | 11349.61 |  | 9354.02 | 8900 |  |  |  |
| 6646.37 | 4467.46 |  | 6483.33 | 7951.09 |  |  |  |
| 5160.17 | 11146.59 |  | 8711.981 | 4515.93 |  |  |  |
| 6624.12 | 7622.59 |  | 3661.49 | 5563.48 |  |  |  |
| 10470.73 | 11507.85 |  | 3446.59 | 6442.21 |  |  |  |
| 21985.75 | 10326.23 |  | 9078.26 | 3078.06 |  |  |  |
| 9103.14 | 3689.7 |  | 11558.25 | 9315.46 |  |  |  |
| 5192.27 | 3027.8 |  | 9878.87 | 11353.23 |  |  |  |
| 8208.58 | 6028.55 |  | 2430.93 | 4320.85 |  |  |  |
| 6482.29 | 4202.79 |  | 4254.12 | 2138.79 |  |  |  |
| 27995.15 | 19076.35 |  | 9038.82 | 6684.64 |  |  |  |
| 22398.83 | 14790.16 |  | 11853.25 | 5000 |  |  |  |
| 17263.52 | 10226.23 |  | 12984.35 | 9459.15 |  |  |  |
| 13344.24 | 7833.69 |  | 7848.56 | 4322.1 |  |  |  |
| 9533.5 | 4987.35 |  | 12420.58 | 5533.93 |  |  |  |
| 12939.12 | 12782.13 |  | 7812.47 | 4277.43 |  |  |  |
| 17128.53 | 9585.42 |  | 9467.08 | 2392.01 |  |  |  |
| 6270.43 | 6222.34 |  | 5208.35 | 1843.67 |  |  |  |
| 6741.88 | 5902.32 |  | 5177.94 | 3467.01 |  |  |  |
| 6264.53 | 6661.56 |  | 6285.59 | 4669.23 |  |  |  |
| 9843.99 | 7899.71 |  | 5165.98 | 854.19 |  |  |  |
| 7830.44 | 5121.06 |  | 4366.35 | 2788.94 |  |  |  |
| 11911.75 | 4527.53 |  | 3500 | 1714.47 |  |  |  |
| 19726.75 | 8360.05 |  | 1220.94 | 1622.93 |  |  |  |
| 5333.99 | 5146.58 |  | 2149.69 | 1505.18 |  |  |  |
| 6621.72 | 5222.61 |  | 1687.24 | 1316.98 |  |  |  |
| 1658.39 | 2749.95 |  |  |  |  |  |  |
| 2497.94 | 3384.77 |  |  |  |  |  |  |
| 2500 | 3235.41 |  |  |  |  |  |  |
| 4084.25 | 4000 |  |  |  |  |  |  |
| 4000 | 4763.86 |  |  |  |  |  |  |
| 4790.66 | 3358.87 |  |  |  |  |  |  |
| 16581.31 | 10029.05 |  |  |  |  |  |  |
| 7735.83 | 2787.45 |  |  |  |  |  |  |
| 11323.42 | 7900 |  |  |  |  |  |  |
| 14335.17 | 7245.65 |  |  |  |  |  |  |
| 11289.82 | 8739.59 |  |  |  |  |  |  |
| 4884.48 | 3664.05 |  |  |  |  |  |  |
| 6777.57 | 5861.16 |  |  |  |  |  |  |
| 5033.64 | 2887.1 |  |  |  |  |  |  |
| 2653.99 | 2904.13 |  |  |  |  |  |  |
| 1540.33 | 1186.46 |  |  |  |  |  |  |
| 2487.28 | 2546.56 |  |  |  |  |  |  |
| 2316.75 | 1500 |  |  |  |  |  |  |
| 2904.71 | 3000 |  |  |  |  |  |  |
| 1375.14 | 2015.99 |  |  |  |  |  |  |
| 13662.93 | 8904.15 |  |  |  |  |  |  |
| 14386.51 | 7429.23 |  |  |  |  |  |  |
| 15606.92 | 3990.27 |  |  |  |  |  |  |
| 24789.92 | 9414.13 |  |  |  |  |  |  |
| 15015.9 | 6000 |  |  |  |  |  |  |
| 1585.03 | 5660 |  |  |  |  |  |  |
| 26655.82 | 9176.35 |  |  |  |  |  |  |
| 19026.93 | 9423.61 |  |  |  |  |  |  |
| 26326.12 | 19256.82 |  |  |  |  |  |  |
| 19977.23 | 5560.86 |  |  |  |  |  |  |
| 26609.67 | 13859.89 |  |  |  |  |  |  |
| 8594.31 | 2168.67 |  |  |  |  |  |  |
| 19962.07 | 5393.5 |  |  |  |  |  |  |
| 6646.37 | 6044.25 |  |  |  |  |  |  |
| 5160.17 | 8119.35 |  |  |  |  |  |  |
| 6624.12 | 5392.28 |  |  |  |  |  |  |
| 10470.73 | 2687.83 |  |  |  |  |  |  |
| 21985.75 | 17411.81 |  |  |  |  |  |  |
| 9103.14 | 5235.86 |  |  |  |  |  |  |
| 5192.27 | 4983.94 |  |  |  |  |  |  |
| 8208.58 | 2536.02 |  |  |  |  |  |  |
| 6482.29 | 3000 |  |  |  |  |  |  |
| 27995.15 | 11365.05 |  |  |  |  |  |  |
| 22398.83 | 7489.69 |  |  |  |  |  |  |
| 17263.52 | 15537.89 |  |  |  |  |  |  |
| 13344.24 | 12428.88 |  |  |  |  |  |  |
| 9533.5 | 8450.69 |  |  |  |  |  |  |
| 12939.12 | 10974.11 |  |  |  |  |  |  |
| 17128.53 | 6000 |  |  |  |  |  |  |
| 6270.43 | 3246.36 |  |  |  |  |  |  |
| 6741.88 | 3658.05 |  |  |  |  |  |  |
| 6264.53 | 2210.62 |  |  |  |  |  |  |
| 9843.99 | 6900 |  |  |  |  |  |  |
| 7830.44 | 3478.48 |  |  |  |  |  |  |
| 11911.75 | 2826.23 |  |  |  |  |  |  |
| 19726.75 | 12808.57 |  |  |  |  |  |  |
| 5333.99 | 8846.33 |  |  |  |  |  |  |
| 6621.72 | 8308.78 |  |  |  |  |  |  |
| 1658.39 | 2601 |  |  |  |  |  |  |
| 2497.94 | 4540 |  |  |  |  |  |  |
| 2500 | 4548.63 |  |  |  |  |  |  |
| 4084.25 | 3974.1 |  |  |  |  |  |  |
| 4000 | 4817.42 |  |  |  |  |  |  |
| 4790.66 | 5245.33 |  |  |  |  |  |  |
| 16581.31 | 6138.1 |  |  |  |  |  |  |
| 7735.83 | 6005.14 |  |  |  |  |  |  |
| 11323.42 | 8715.981 |  |  |  |  |  |  |
| 14335.17 | 5876.01 |  |  |  |  |  |  |
| 11289.82 | 6000 |  |  |  |  |  |  |
| 4884.48 | 4500 |  |  |  |  |  |  |
| 6777.57 | 2640.74 |  |  |  |  |  |  |
| 5033.64 | 2027.62 |  |  |  |  |  |  |
| 2653.99 | 2500 |  |  |  |  |  |  |
| 1540.33 | 2499.83 |  |  |  |  |  |  |
| 2487.28 | 1885.51 |  |  |  |  |  |  |
| 2316.75 | 2413.05 |  |  |  |  |  |  |
| 2904.71 | 3000 |  |  |  |  |  |  |
| 1375.14 | 1128.57 |  |  |  |  |  |  |
| 13662.93 | 9000 |  |  |  |  |  |  |
| 14386.51 | 4192.79 |  |  |  |  |  |  |
| 15606.92 | 9472.74 |  |  |  |  |  |  |
| 22214.36 | 15107.64 |  |  |  |  |  |  |
| 10344.23 | 6849.96 |  |  |  |  |  |  |
| 20278.95 | 4510.59 |  |  |  |  |  |  |
| 22692.71 | 14464.55 |  |  |  |  |  |  |
| 16618.66 | 10842.09 |  |  |  |  |  |  |
| 6434.45 | 6227.12 |  |  |  |  |  |  |
| 15202.91 | 8428.79 |  |  |  |  |  |  |
| 7170.01 | 3214.6 |  |  |  |  |  |  |
| 11767.1 | 4690.8 |  |  |  |  |  |  |
| 10386.66 | 8000 |  |  |  |  |  |  |
| 6386.19 | 3800 |  |  |  |  |  |  |
| 10033.43 | 6103.28 |  |  |  |  |  |  |
| 9071.2 | 4000 |  |  |  |  |  |  |
| 5695.54 | 6022.72 |  |  |  |  |  |  |
| 8038.98 | 4000 |  |  |  |  |  |  |
| 1897.94 | 1840.1 |  |  |  |  |  |  |
| 15650.07 | 5000 |  |  |  |  |  |  |
| 5175.35 | 2112.76 |  |  |  |  |  |  |
| 8858.32 | 4000 |  |  |  |  |  |  |
| 20789.25 | 8000 |  |  |  |  |  |  |
| 4453.27 | 5496.3 |  |  |  |  |  |  |
| 2144.65 | 1745.46 |  |  |  |  |  |  |
| 22214.36 | 9000 |  |  |  |  |  |  |
| 10344.23 | 3229.22 |  |  |  |  |  |  |
| 20278.95 | 8471.03 |  |  |  |  |  |  |
| 22692.71 | 6798.6 |  |  |  |  |  |  |
| 16618.66 | 5340.79 |  |  |  |  |  |  |
| 6434.45 | 6000 |  |  |  |  |  |  |
| 15202.91 | 8225.97 |  |  |  |  |  |  |
| 7170.01 | 7100.11 |  |  |  |  |  |  |
| 11767.1 | 6964.91 |  |  |  |  |  |  |
| 10386.66 | 10557.18 |  |  |  |  |  |  |
| 6386.19 | 3779.81 |  |  |  |  |  |  |
| 10033.43 | 6000 |  |  |  |  |  |  |
| 9071.2 | 3867.9 |  |  |  |  |  |  |
| 5695.54 | 4653.25 |  |  |  |  |  |  |
| 8038.98 | 4000 |  |  |  |  |  |  |
| 1897.94 | 2800.99 |  |  |  |  |  |  |
| 15650.07 | 6143.74 |  |  |  |  |  |  |
| 5175.35 | 2200 |  |  |  |  |  |  |
| 8858.32 | 6243.46 |  |  |  |  |  |  |
| 20789.25 | 7315.52 |  |  |  |  |  |  |
| 4453.27 | 2008.02 |  |  |  |  |  |  |
| 2144.65 | 2889.94 |  |  |  |  |  |  |
| 22214.36 | 12984.25 |  |  |  |  |  |  |
| 10344.23 | 4000 |  |  |  |  |  |  |
| 20278.95 | 10502.6 |  |  |  |  |  |  |
| 22692.71 | 4616.87 |  |  |  |  |  |  |
| 16618.66 | 7233.25 |  |  |  |  |  |  |
| 6434.45 | 9951.13 |  |  |  |  |  |  |
| 15202.91 | 6564.81 |  |  |  |  |  |  |
| 7170.01 | 7000 |  |  |  |  |  |  |
| 11767.1 | 3911.52 |  |  |  |  |  |  |
| 10386.66 | 9202.88 |  |  |  |  |  |  |
| 6386.19 | 3606.95 |  |  |  |  |  |  |
| 10033.43 | 6769.13 |  |  |  |  |  |  |
| 9071.2 | 4191.07 |  |  |  |  |  |  |
| 5695.54 | 5000 |  |  |  |  |  |  |
| 8038.98 | 6271.96 |  |  |  |  |  |  |
| 1897.94 | 2639.7 |  |  |  |  |  |  |
| 15650.07 | 7929.51 |  |  |  |  |  |  |
| 5175.35 | 3305.51 |  |  |  |  |  |  |
| 8858.32 | 4128.13 |  |  |  |  |  |  |
| 20789.25 | 13662.48 |  |  |  |  |  |  |
| 4453.27 | 2000 |  |  |  |  |  |  |
| 2144.65 | 1008.21 |  |  |  |  |  |  |
| 7114.54 | 11918.08 |  |  |  |  |  |  |
| 6574.38 | 5063.14 |  |  |  |  |  |  |
| 6359.98 | 5483.34 |  |  |  |  |  |  |
| 6855.65 | 15562.71 |  |  |  |  |  |  |
| 18000 | 15674.56 |  |  |  |  |  |  |
| 7283.38 | 7484.69 |  |  |  |  |  |  |
| 3600 | 3546.93 |  |  |  |  |  |  |
| 11331.95 | 6434.46 |  |  |  |  |  |  |
| 3500 | 6344.5 |  |  |  |  |  |  |
| 17433.47 | 8684.07 |  |  |  |  |  |  |
| 4436.44 | 4783.38 |  |  |  |  |  |  |
| 12880.14 | 6937.76 |  |  |  |  |  |  |
| 7748.58 | 3340.95 |  |  |  |  |  |  |
| 6000 | 4000 |  |  |  |  |  |  |
| 11261.87 | 9386.29 |  |  |  |  |  |  |
| 8000 | 6593.11 |  |  |  |  |  |  |
| 9855.17 | 7350.39 |  |  |  |  |  |  |
| 6519.4 | 7038.76 |  |  |  |  |  |  |
| 6241.01 | 3274.6 |  |  |  |  |  |  |
| 20000 | 8195.55 |  |  |  |  |  |  |
| 21844.04 | 9151.09 |  |  |  |  |  |  |
| 7857.3 | 3072.84 |  |  |  |  |  |  |
| 7000 | 3876.73 |  |  |  |  |  |  |
| 2428.41 | 1571.74 |  |  |  |  |  |  |
| 19061.91 | 5000 |  |  |  |  |  |  |
| 482.13 | 6299.44 |  |  |  |  |  |  |
| 5066.48 | 6865.39 |  |  |  |  |  |  |
| 6881.79 | 14668.71 |  |  |  |  |  |  |
| 6680.96 | 6000 |  |  |  |  |  |  |
| 10888.83 | 4000 |  |  |  |  |  |  |
| 20268.26 | 7121.31 |  |  |  |  |  |  |
| 3548.61 | 5060.31 |  |  |  |  |  |  |
| 1956.24 | 4427.44 |  |  |  |  |  |  |
| 7200 | 7231.07 |  |  |  |  |  |  |
| 2901.72 | 3015.02 |  |  |  |  |  |  |
| 2360.99 | 3541.24 |  |  |  |  |  |  |
| 4112.33 | 2896.24 |  |  |  |  |  |  |
| 1712.57 | 1537.21 |  |  |  |  |  |  |
| 2518.26 | 2506.16 |  |  |  |  |  |  |
| 4951.96 | 2500 |  |  |  |  |  |  |
| 2069.07 | 675.01 |  |  |  |  |  |  |
| 1777.42 | 2068.66 |  |  |  |  |  |  |
| 5760.04 | 2066.91 |  |  |  |  |  |  |
| 14901.89 | 9817.3 |  |  |  |  |  |  |
| 8298.02 | 4288.89 |  |  |  |  |  |  |
| 12000 | 7073.65 |  |  |  |  |  |  |
| 13633.69 | 9987.47 |  |  |  |  |  |  |
| 7325.85 | 1612.22 |  |  |  |  |  |  |
| 4512.44 | 3246.45 |  |  |  |  |  |  |
| 5994.03 | 6148.8 |  |  |  |  |  |  |
| 13558.62 | 4299.43 |  |  |  |  |  |  |
| 19396.34 | 15800 |  |  |  |  |  |  |
| 9570.71 | 4843.6 |  |  |  |  |  |  |
| 9301.04 | 6000 |  |  |  |  |  |  |
| 6871.15 | 3905.59 |  |  |  |  |  |  |
| 5749.97 | 6470.25 |  |  |  |  |  |  |
| 21466.57 | 14791.52 |  |  |  |  |  |  |
| 5000 | 5000 |  |  |  |  |  |  |
| 17300.42 | 6500 |  |  |  |  |  |  |
| 3088.7 | 8680.9 |  |  |  |  |  |  |
| 13075.99 | 6000 |  |  |  |  |  |  |
| 5344.59 | 6526.68 |  |  |  |  |  |  |
| 7460.86 | 5219 |  |  |  |  |  |  |
| 4266.94 | 6311.03 |  |  |  |  |  |  |
| 9424.3 | 6000 |  |  |  |  |  |  |
| 8000 | 7000 |  |  |  |  |  |  |
| 12774.89 | 5259.49 |  |  |  |  |  |  |
| 6321.05 | 2772.54 |  |  |  |  |  |  |
| 20299.39 | 8500 |  |  |  |  |  |  |
| 13626.79 | 8925.4 |  |  |  |  |  |  |
| 3984.62 | 2146.6 |  |  |  |  |  |  |
| 6988.69 | 1763.53 |  |  |  |  |  |  |
| 2223.26 | 2617.79 |  |  |  |  |  |  |
| 22027.42 | 10706.77 |  |  |  |  |  |  |
| 12560.86 | 11013 |  |  |  |  |  |  |
| 8482.58 | 13000 |  |  |  |  |  |  |
| 13730.29 | 10573.06 |  |  |  |  |  |  |
| 11000 | 8835.26 |  |  |  |  |  |  |
| 14000 | 13203.5 |  |  |  |  |  |  |
| 4888.61 | 1174.08 |  |  |  |  |  |  |
| 2575.48 | 2500 |  |  |  |  |  |  |
| 15938.87 | 12492.71 |  |  |  |  |  |  |
| 4978.58 | 2000 |  |  |  |  |  |  |
| 2624.52 | 4500 |  |  |  |  |  |  |
| 4249.18 | 2121.73 |  |  |  |  |  |  |
| 3206.62 | 2000 |  |  |  |  |  |  |
| 9664.8 | 7423.94 |  |  |  |  |  |  |
| 1547.86 | 2884.07 |  |  |  |  |  |  |
| 2705.93 | 3860.29 |  |  |  |  |  |  |
| 1834.2 | 1328.63 |  |  |  |  |  |  |
| 2116.13 | 1753.5 |  |  |  |  |  |  |
| 6787.1 | 2182.04 |  |  |  |  |  |  |
| 7500 | 4803.29 |  |  |  |  |  |  |
| 14181.33 | 7000 |  |  |  |  |  |  |

**HeLa cells**

| max dyn1 | max dynab |  | max dyn2 | max dynab |  | max Dyn1_mCherry | max dyn1_EGFP |
| --- | --- | --- | --- | --- | --- | --- | --- |
| 2979.09 | 6815.98 |  | 6135.1 | 5070.76 |  | 3363.21 | 5274.06 |
| 8144.69 | 9943.94 |  | 6875.69 | 6844.89 |  | 1869.78 | 3044.593 |
| 2510.93 | 3045.25 |  | 4388.54 | 2692.89 |  | 3053.08 | 3143.054 |
| 4212.34 | 3061.73 |  | 4338.65 | 4919.6 |  | 2872.91 | 5671.342 |
| 4229.41 | 5016.47 |  | 2832.68 | 2877.08 |  | 2161.56 | 3794.58 |
| 3833.23 | 4263.66 |  | 3193.03 | 2633.48 |  | 1672.56 | 5081.593 |
| 2325.03 | 4192.68 |  | 3948.14 | 3117.61 |  | 3077.47 | 5719.451 |
| 2069.46 | 4209.22 |  | 6704.63 | 11038.47 |  | 11257.33 | 10749.63 |
| 1534.84 | 4482.05 |  | 4741.23 | 2349.25 |  | 2131.37 | 5895.898 |
| 6933.29 | 5946.08 |  | 2519.22 | 2996.51 |  | 4753.18 | 5999.557 |
| 2810.94 | 2636.1 |  | 4500.13 | 3243.03 |  | 4365.22 | 4321.631 |
| 4176.52 | 5042.39 |  | 1600.11 | 1522.31 |  | 5189.56 | 9041.327 |
| 1996.54 | 4051.84 |  | 5992.91 | 3869.05 |  | 3758.47 | 4491.914 |
| 2417.06 | 3508.45 |  | 3656.35 | 3806.84 |  | 4374.43 | 5672.15 |
| 2221.92 | 7572.73 |  | 3644.88 | 5217.71 |  | 1378.04 | 4250.991 |
| 11000 | 11000 |  | 3118.73 | 2380.5 |  | 2124.27 | 2886.31 |
| 1104.95 | 4864.11 |  | 4943.18 | 3647.1 |  | 4171.01 | 4906.914 |
| 6244.52 | 3804.4 |  | 3761.33 | 4227 |  | 16759.98 | 8263.27 |
| 5337.98 | 2189.22 |  | 2336.43 | 2374.05 |  | 5753.84 | 7698.953 |
| 1484.9 | 6250.49 |  | 2905.53 | 1560.05 |  | 7258.28 | 8527.093 |
| 1736.56 | 7310.32 |  | 4576.86 | 4830.47 |  | 6083.9 | 9383.42 |
| 2355.75 | 2848.99 |  | 3737.16 | 3396.21 |  | 3678.57 | 3693.278 |
| 1920.07 | 2259.96 |  | 7391.76 | 6334.78 |  | 1866.3 | 3114.524 |
| 3018.85 | 2967.96 |  | 3813.18 | 5051.79 |  | 2941.6 | 7264.429 |
| 2269.92 | 4747.44 |  | 10689.14 | 4976.57 |  | 4976.25 | 8030.208 |
| 2011.45 | 2130.28 |  | 2075.54 | 4693.8 |  | 5163.74 | 4887.577 |
| 2634.21 | 2722.97 |  | 2494.29 | 1725.8 |  | 4890.46 | 6743.982 |
| 3064.53 | 3896.55 |  | 2650.4 | 3137.12 |  | 4022 | 6035.481 |
| 1601.94 | 4402.04 |  | 1248.24 | 2419.2 |  | 3562.02 | 4076.117 |
| 6718.03 | 5514.76 |  | 3625.71 | 3615.92 |  | 6131.86 | 10058.37 |
| 5704.72 | 6483.94 |  | 5562.03 | 6056.24 |  | 3870.63 | 6433.564 |
| 3902.45 | 3993.58 |  | 5978.03 | 3913.41 |  | 6517.7 | 6151.405 |
| 2284.69 | 5029.17 |  | 2484.44 | 3115.08 |  | 4915.62 | 6625.414 |
| 1927.11 | 5973.53 |  | 4506.55 | 4230.77 |  | 3387.33 | 4302.119 |
| 1873.56 | 2581.84 |  | 1506.15 | 2268.56 |  | 2476.23 | 2296.885 |
| 2859.21 | 4263.37 |  | 1769.89 | 2866.51 |  | 1717.01 | 1625.123 |
| 2744.01 | 4149.7 |  | 2029.97 | 3636.36 |  | 5571.11 | 8189.19 |
| 1351.11 | 2583.9 |  | 2191.45 | 2040.1 |  | 1988.53 | 3719.129 |
| 3492.2 | 2761.13 |  | 1519.39 | 4199.72 |  | 2263.23 | 5033.043 |
| 4230.09 | 3273.34 |  | 3449.5 | 6705.3 |  | 2403.65 | 4290.373 |
| 4375.97 | 4964.94 |  | 7847.49 | 7390.56 |  | 4351.69 | 4368.002 |
| 959.9 | 2346.1 |  | 8471.37 | 8450.04 |  | 6070.04 | 4525.588 |
| 4194.79 | 3416.09 |  | 6074.07 | 7095.4 |  | 1371.68 | 2481.3 |
| 1213.1 | 2776.37 |  | 3637.31 | 5288.41 |  | 6673.58 | 9468.609 |
| 2782.06 | 3623.99 |  | 3813.09 | 4396.63 |  | 3840.42 | 4529.088 |
| 2448.04 | 2677.64 |  | 6155.97 | 7456.93 |  | 7918.09 | 5119.002 |
| 1689.34 | 2130.55 |  | 1389.17 | 5365.85 |  | 13559.15 | 12590.67 |
| 2829.21 | 6362.16 |  | 3387.92 | 6610.32 |  | 4798.25 | 3822.448 |
| 1556.3 | 3166.64 |  | 2614.52 | 7299.97 |  | 6464.25 | 5417.353 |
| 1578.62 | 3775.75 |  | 11295.44 | 15215.67 |  | 4265.47 | 8130.099 |
| 2478.67 | 6145.29 |  | 4271.86 | 4534.63 |  | 9232.84 | 8388.958 |
| 1029.87 | 3739.52 |  | 3995.47 | 5522.92 |  | 3239.93 | 7748.575 |
| 2381.8 | 3162.59 |  | 3907.05 | 15998.25 |  | 8158.61 | 10723.35 |
| 2308.96 | 4627.83 |  | 2543.15 | 6236.98 |  | 4042.74 | 7066.12 |
| 2592.47 | 3606.52 |  | 2528.21 | 4206.05 |  | 7546.44 | 9733.117 |
| 4588.69 | 4369.84 |  | 5323.49 | 4299.76 |  | 3419.83 | 4577.407 |
| 2688.25 | 3026.64 |  | 2535.54 | 5598.02 |  | 7404.8 | 10197.84 |
| 3976 | 3027.03 |  | 2533.4 | 5440.94 |  | 5094.72 | 9313.204 |
| 3147.86 | 4070.96 |  | 6660.65 | 7894.61 |  | 6999.16 | 5694.981 |
| 2168.08 | 1839.21 |  | 5533.55 | 8165.42 |  | 9417.7 | 5305.722 |
| 4060.8 | 2927.98 |  | 6184.59 | 7742.48 |  | 9396.12 | 15588.97 |
| 1713.73 | 3110.61 |  | 1596.43 | 4194.75 |  | 3062.23 | 8158.77 |
| 1714.08 | 3931.6 |  | 3682.13 | 4967.16 |  | 4251.4 | 8278.619 |
| 1254.08 | 2783.67 |  | 4750.42 | 7135.51 |  | 4655.87 | 8306.509 |
| 1554.53 | 2961.81 |  | 3311.34 | 5176.37 |  | 3087.68 | 5942.28 |
| 1950.94 | 4113.63 |  | 5523.12 | 6779.98 |  | 4731.15 | 6180.526 |
| 1041.9 | 3020.53 |  | 3497.58 | 5880.99 |  | 5710.64 | 7156.805 |
| 4219.94 | 3644.29 |  | 2687.38 | 4145.18 |  | 17070.78 | 12070.32 |
| 3341.96 | 3023.02 |  | 3804.14 | 4730.17 |  | 7542.82 | 4900.439 |
| 5402.73 | 4849.91 |  | 3652.73 | 5241.33 |  | 1890.6 | 2678.853 |
| 4752.06 | 3620.87 |  | 4484.5 | 2906.22 |  | 6528.3 | 7267.783 |
| 4053.95 | 3658.57 |  | 6268.08 | 7423.87 |  | 11194.07 | 12059.93 |
| 1544.61 | 2101.16 |  | 2552.5 | 4991.71 |  | 4362.66 | 4846.601 |
| 6445.77 | 2847.28 |  | 2298.44 | 5464.29 |  | 6085.62 | 9243.374 |
| 3536.07 | 2317.82 |  | 1596.3 | 4652.09 |  | 5334.32 | 6312.103 |
| 4057.5 | 3016.89 |  | 3291.88 | 3854.3 |  | 4947.61 | 3472.287 |
| 4757.5 | 5481.33 |  | 2904.25 | 5958.28 |  | 5446.21 | 9976.72 |
| 3438.4 | 3104.08 |  | 5313.46 | 4845.41 |  | 7817.73 | 7163.06 |
| 2808.88 | 4561.13 |  | 6195.07 | 7503.72 |  | 10003.05 | 10758.09 |
| 10004.94 | 2635.25 |  | 4622.11 | 5363.88 |  | 7834.59 | 12307.16 |
| 2060.74 | 3061.09 |  | 4075.57 | 5382.02 |  | 6177.06 | 8166.216 |
| 1855.23 | 2419.41 |  | 9375.78 | 8823.89 |  | 6148.52 | 8225.691 |
| 3720.65 | 2354.07 |  | 3938.48 | 4331.33 |  | 7403.7 | 8074.006 |
| 4117.3 | 2943.07 |  | 4212.64 | 6647.38 |  | 8348.14 | 8245.803 |
| 7320.53 | 5245.44 |  | 4171.24 | 6572.04 |  | 5994.19 | 4703.787 |
| 4833.92 | 4885.21 |  | 16097.51 | 9448.59 |  | 8850.39 | 11278.71 |
| 1452.45 | 2923.71 |  | 5870.23 | 9344.89 |  | 3497.37 | 5059.971 |
| 4316.09 | 3706.44 |  | 2302.72 | 9958.08 |  | 8780.87 | 8457.912 |
| 3314.6 | 3011.33 |  | 4396.95 | 9572.02 |  | 3047.77 | 7285.829 |
| 4344.14 | 7368.09 |  | 2346.38 | 8934.49 |  | 10446.03 | 11881.51 |
| 870.85 | 2621.14 |  | 2005.4 | 12281.77 |  | 5235.51 | 6637.318 |
| 4928.46 | 4487.53 |  | 2421.21 | 9759.11 |  | 6909.32 | 10254.67 |
| 6020.78 | 3860.12 |  | 596.45 | 4720.48 |  | 5282.1 | 9282.763 |
| 3443.05 | 4377.21 |  | 7020.94 | 11655.4 |  | 4591.73 | 17088.51 |
| 3417.3 | 1866.33 |  | 1205.8 | 4950.97 |  | 5356.43 | 3421.968 |
| 6185.16 | 4016.39 |  | 3473.64 | 12195.04 |  | 6711.01 | 6278.843 |
| 5245.44 | 3250.31 |  | 2115.49 | 10229.28 |  | 6247.76 | 5584.811 |
| 3615.04 | 3639.29 |  | 2173.21 | 7075.33 |  | 5583.66 | 9875.44 |
| 1252.62 | 2559.82 |  | 2886.62 | 11101.68 |  | 4810.14 | 10957.44 |
| 2123.83 | 1693.02 |  | 3373.66 | 13931.03 |  | 7843.39 | 8292.036 |
| 1542.41 | 3695.18 |  | 1765.25 | 17771.65 |  | 3854.71 | 11189.79 |
| 4052.42 | 2131.54 |  | 2129.07 | 7540.62 |  | 3423.61 | 4990.065 |
| 3044.73 | 4837.22 |  | 1530.03 | 6471.24 |  | 5526.03 | 6562.998 |
| 3254.55 | 2768.27 |  | 3576.64 | 8446.51 |  | 5906.58 | 11147.09 |
| 3867.92 | 2440.1 |  | 1707.51 | 6576.19 |  | 5645.32 | 9583.252 |
| 4206.31 | 2458.4 |  | 1297.95 | 7400.95 |  | 2953.67 | 4884.478 |
| 1978.54 | 1549.93 |  | 2128.23 | 6809.88 |  | 9366.77 | 11859.66 |
| 2813.23 | 3689.25 |  | 1847.77 | 9214.07 |  | 7443.58 | 10415.49 |
| 2683.13 | 5023.8 |  | 1981.77 | 7418.25 |  | 6159.97 | 4781.594 |
| 2129.57 | 1658.24 |  | 2994.89 | 8504.12 |  | 11130.75 | 12756.98 |
| 4439.5 | 3119.91 |  | 4341.89 | 12558.89 |  | 2899.46 | 3560.709 |
| 914.26 | 1250.91 |  | 3235.22 | 7776.96 |  | 4699.27 | 6190.205 |
| 2205.25 | 2735.99 |  | 2554.97 | 11801.19 |  | 6197.14 | 10441.96 |
| 4627.29 | 3677.14 |  | 3877.81 | 11999.78 |  | 4817.8 | 6579.068 |
| 3189.13 | 5543.9 |  | 2424.52 | 7145.69 |  | 10454.75 | 14972.99 |
| 5571.51 | 3851.05 |  | 4062.11 | 6944.03 |  | 2591.57 | 6585.725 |
| 1897.38 | 1507.32 |  | 2453.48 | 13110.15 |  | 7279.56 | 6339.657 |
| 987.82 | 2696.04 |  | 2725.78 | 9296.01 |  | 2821.86 | 6251.835 |
| 1693.57 | 2223.6 |  | 1491.97 | 10689.01 |  | 5175.58 | 9354.243 |
| 1722.19 | 3584.51 |  | 2309.99 | 7528.46 |  | 6032.29 | 7604.333 |
| 2321.02 | 3504.72 |  | 3452.67 | 10494.28 |  | 4293.46 | 6643.356 |
| 1795.89 | 1182.11 |  | 2674.06 | 7217.96 |  | 3782.55 | 14780.44 |
| 4987.07 | 5841.52 |  | 5608.47 | 13820.09 |  | 6102.16 | 14286.15 |
| 2939.41 | 3910.75 |  | 5061.86 | 13809.22 |  | 4544.58 | 7284.33 |
| 3028.81 | 6101.41 |  | 2580.18 | 7215.4 |  | 2117.8 | 3481.548 |
| 2481.7 | 1892.06 |  | 3856.07 | 9668.42 |  | 6624.07 | 7140.439 |
| 2339.6 | 3072.53 |  | 2557.31 | 11164.22 |  | 6374.46 | 7252.543 |
| 1888.55 | 2214.63 |  | 2054.62 | 8452.55 |  | 6100.69 | 8432.68 |
| 3484.32 | 6890.88 |  | 3466.63 | 12911.79 |  | 4556.75 | 7796.453 |
| 2172.24 | 2511.48 |  | 4243.1 | 9789.7 |  | 3708.9 | 7178.034 |
| 5839.5 | 6417.23 |  | 1690.24 | 7381.02 |  | 4973.15 | 7525.977 |
| 1668.19 | 2165.1 |  | 1419.01 | 3923.64 |  | 4527.58 | 5930.273 |
| 2393.72 | 2870.25 |  | 21558.56 | 20751.84 |  | 3387.26 | 12150.51 |
| 1099.31 | 2445.54 |  | 10290.99 | 10978.69 |  | 4070.02 | 4762.896 |
| 2537.38 | 4305.37 |  | 9666.29 | 7661.89 |  | 3783.95 | 8060.276 |
| 1869.99 | 2220.53 |  | 3781.15 | 9177.31 |  | 5434.95 | 15701.48 |
| 3420.2 | 3166.61 |  | 6182.66 | 7323.68 |  | 5490.35 | 7673.671 |
| 1861.19 | 1944.07 |  | 8277.64 | 8176.9 |  | 4765.7 | 6132.953 |
| 2434.3 | 3798.21 |  | 6825.67 | 9015.97 |  | 3815.75 | 8700.265 |
| 5968.15 | 11938.51 |  | 5887.35 | 5769.24 |  | 2532.57 | 8060.248 |
| 5401.9 | 5320.53 |  | 4831.77 | 6153.15 |  | 8781.65 | 13890.13 |
| 4566.5 | 4304.27 |  | 3879.17 | 11020.62 |  | 2603.3 | 17649.23 |
| 3528.04 | 3105.67 |  | 9852.88 | 9521.12 |  | 3598.56 | 6949.822 |
| 3451.5 | 2684.02 |  | 6953.01 | 9906.7 |  | 9301.43 | 11860.06 |
| 2687.55 | 2749.35 |  | 3626.77 | 8035.46 |  | 7204.79 | 15377.24 |
| 5250.13 | 3235.36 |  | 6147.46 | 4518.92 |  | 8110.99 | 10959.07 |
| 3805.53 | 4294.15 |  | 6854.33 | 11451.12 |  | 9298.15 | 18475.92 |
| 4250.92 | 3159.2 |  | 8476.8 | 19626.68 |  | 14780.44 | 14956.98 |
| 2804.97 | 3392.03 |  | 10273.72 | 16253.16 |  | 6668.93 | 9749.03 |
| 2455.78 | 1910.07 |  | 11798.71 | 8981.09 |  | 8039.81 | 8460.233 |
| 5038.32 | 6631.95 |  | 12223.31 | 10514.6 |  | 3930.8 | 7494.11 |
| 5435.26 | 4098.8 |  | 10420.52 | 10500.89 |  | 7850.07 | 14345.88 |
| 6023.97 | 3710.38 |  | 5258.93 | 7409.83 |  | 3959.53 | 8736.429 |
| 3612.92 | 2385.59 |  | 13308.13 | 6791.01 |  | 4415.82 | 10615.49 |
| 1842.14 | 1904.01 |  | 7535.33 | 14560.9 |  | 8141.33 | 7910.042 |
| 1713.8 | 1686.22 |  | 5464.54 | 10858.67 |  | 5872.67 | 8970.356 |
| 1348.61 | 2037.13 |  | 3062.65 | 6393.08 |  | 5350.5 | 5983.204 |
| 3663.48 | 2533.9 |  | 7419.88 | 11533.66 |  | 10450.49 | 9946.302 |
| 1816.77 | 2007.93 |  | 8720.66 | 11415.72 |  | 5069.82 | 3863.305 |
| 5282.2 | 3175.1 |  | 8085.57 | 9689.37 |  | 11613.14 | 14272.9 |
| 3492.89 | 6145.76 |  | 8521.27 | 8814.94 |  | 5846.53 | 12925.48 |
| 1189.91 | 6311.23 |  | 12245.61 | 11888.77 |  | 15994.21 | 19352.96 |
| 1850.77 | 2575.52 |  | 3782.15 | 8621.13 |  | 4278.31 | 6194.718 |
| 1817.37 | 4600.78 |  | 13655.51 | 15344.58 |  | 17089.96 | 22808.14 |
| 1352.14 | 4706.37 |  | 4843.75 | 6086.59 |  | 14535.67 | 14456.72 |
| 6521.57 | 6172.27 |  | 7321.93 | 7225.77 |  | 9066.6 | 16012.56 |
| 1927.31 | 3676.9 |  | 11475.12 | 9060.44 |  | 7191.06 | 4660.178 |
| 2804.9 | 4061.58 |  | 8950.61 | 9047.47 |  | 1788.68 | 4439.455 |
| 869.51 | 2621.61 |  | 5715.57 | 8732.07 |  | 4021.47 | 9380.46 |
| 3338.06 | 3009.26 |  | 6805.9 | 8038.62 |  | 7956.63 | 8910.427 |
| 713.76 | 2313.88 |  | 9618.46 | 11515.06 |  | 8734.38 | 13175.23 |
| 1866.53 | 4128.79 |  | 14806.79 | 13643.52 |  | 4057.57 | 7706.794 |
| 3811.98 | 4852.14 |  | 1249.05 | 6911.92 |  | 15641.75 | 14667.22 |
| 4666.12 | 3591.91 |  | 6789.88 | 9518.56 |  | 11032.68 | 16972.46 |
| 1095.86 | 3511.52 |  | 7885.35 | 9615.21 |  | 5694.32 | 8679.556 |
| 2663.59 | 4075.97 |  | 2759.3 | 6921.76 |  | 5411.28 | 6733.866 |
| 4847.25 | 3902.2 |  | 8823.94 | 9049.55 |  | 10406.02 | 8110.015 |
| 959.67 | 3689.1 |  | 4142.44 | 10078.9 |  | 8528.37 | 11109.39 |
| 1812.17 | 6026.68 |  | 23910.32 | 21349.03 |  | 10940.82 | 10526.27 |
| 1541.63 | 2956.89 |  | 6611.58 | 9403.55 |  | 4463.47 | 4822.11 |
| 3246.31 | 4128.86 |  | 14732.83 | 12737.64 |  | 8320.05 | 11104.78 |
| 1867.88 | 4362.68 |  | 5945.63 | 7000.34 |  | 8213.66 | 5966.051 |
| 1504.83 | 2733.83 |  | 1967.27 | 6356.8 |  | 8178.76 | 13167.02 |
| 1604.48 | 4055.68 |  | 5259.25 | 6726.65 |  | 11633.96 | 15765.79 |
| 2266.1 | 2001.61 |  | 5569.33 | 5831.47 |  | 6388.86 | 8718.046 |
| 2109.1 | 2132.32 |  | 2260.47 | 5733.23 |  | 5219.17 | 6242.661 |
| 2061.92 | 2351.38 |  | 3897.74 | 10675.66 |  | 16278.15 | 10307.16 |
| 3606.09 | 4236.87 |  | 11549.24 | 12380.78 |  | 9566.58 | 19551.21 |
| 4213.45 | 2689.81 |  | 10163.63 | 9660.87 |  | 3708.69 | 7776.902 |
| 1311.74 | 3291.28 |  | 4247.09 | 11643.57 |  | 9306.38 | 5615.469 |
| 2398.92 | 3671.51 |  | 9615.38 | 8772.21 |  | 4589.41 | 15500.4 |
| 2574.39 | 1349.82 |  | 3228.53 | 9193.61 |  | 6531.88 | 10396.99 |
| 2777.87 | 4326.84 |  | 3001.98 | 6376.6 |  | 3151.9 | 9254.014 |
| 1383.42 | 1823.26 |  | 5407.08 | 5822.23 |  | 4342.76 | 5371.505 |
| 3119.05 | 2790.64 |  | 4497.83 | 11128.27 |  | 9396.981 | 17213.12 |
| 2801.01 | 3865.36 |  | 3622.17 | 8151.31 |  | 5776.82 | 11563.91 |
| 4393.14 | 2573.5 |  | 7941.91 | 7519.04 |  | 7493.98 | 12929.48 |
| 2991.47 | 3937.11 |  | 4777.72 | 4072.08 |  | 5094.6 | 10510.22 |
| 894.89 | 1726.48 |  | 3765.74 | 7660.97 |  | 3363.21 | 5274.06 |
| 3596.91 | 3873.93 |  | 8281.35 | 12578.24 |  | 1869.78 | 3044.59 |
| 1916.02 | 4906.11 |  | 6867.62 | 10039.86 |  | 3053.08 | 3143.05 |
| 2025.68 | 3583.83 |  | 8699.481 | 12252.81 |  | 2872.91 | 5671.34 |
| 1832.37 | 5271.55 |  | 10656.31 | 8884.19 |  | 2161.56 | 3794.58 |
| 2074.2 | 6405.55 |  | 6430.16 | 9654.88 |  | 1672.56 | 5081.59 |
| 2417.74 | 3016.62 |  | 9418.78 | 11364.31 |  | 3077.47 | 5719.45 |
| 1236.27 | 1794.88 |  | 9755.59 | 12766.94 |  | 11257.33 | 10749.63 |
| 1655.72 | 2699.44 |  | 8971.55 | 18284.32 |  | 2131.37 | 5895.9 |
| 1807 | 1327.71 |  | 1079.41 | 3494.74 |  | 4753.18 | 5999.56 |
| 1245.96 | 5062.86 |  | 7889.15 | 11931.56 |  | 4365.22 | 4321.63 |
| 2871.55 | 2528.99 |  | 9464.72 | 11513.74 |  | 5189.56 | 9041.33 |
| 1684.96 | 2619.33 |  | 6885.54 | 12896.89 |  | 3758.47 | 4491.91 |
| 1858.78 | 3199.22 |  | 5899.58 | 10447.95 |  | 4374.43 | 5672.15 |
| 1874.99 | 2532.73 |  | 7369.22 | 4473.24 |  | 1378.04 | 4250.99 |
| 1069.32 | 4398.21 |  | 4454.93 | 10653.87 |  | 2124.27 | 2886.31 |
| 1918.48 | 3856.74 |  | 6089.4 | 8315.39 |  | 4171.01 | 4906.91 |
| 3221.35 | 4221.98 |  | 4098.02 | 8597.29 |  | 16759.98 | 8263.27 |
| 2392.89 | 1821.13 |  | 12141.63 | 12830.47 |  | 5753.84 | 7698.95 |
| 4144.51 | 4215.89 |  | 10720.1 | 14221.09 |  | 7258.28 | 8527.09 |
| 2403.16 | 3729.57 |  | 12861.73 | 12281 |  | 6083.9 | 9383.42 |
| 1434.01 | 1324.2 |  | 5243.16 | 6456.92 |  | 3678.57 | 3693.28 |
| 2865.41 | 1458.87 |  | 4126.51 | 9104.32 |  | 1866.3 | 3114.52 |
| 2053.43 | 2644.76 |  | 12025.91 | 7899.62 |  | 2941.6 | 7264.43 |
| 1867.5 | 2020.32 |  | 3666.78 | 4883.06 |  | 4976.25 | 8030.21 |
| 2383.56 | 4070 |  | 6769.92 | 13068.6 |  | 5163.74 | 4887.58 |
| 6200.18 | 3387.89 |  | 2715.35 | 6135.14 |  | 4890.46 | 6743.98 |
| 7640.34 | 4020.01 |  | 7377.2 | 14541.59 |  | 4022 | 6035.48 |
| 1179.26 | 1091.63 |  | 2360.38 | 6183.71 |  | 3562.02 | 4076.12 |
| 1319.43 | 963.66 |  | 10242.26 | 11404.42 |  | 6131.86 | 10058.37 |
| 2168.18 | 2081.63 |  | 9582.39 | 15582.17 |  | 3870.63 | 6433.56 |
| 2125.24 | 2278.01 |  | 8832.57 | 11796.23 |  | 6517.7 | 6151.41 |
| 6381.89 | 4743.73 |  | 3942.63 | 6501.14 |  | 4915.62 | 6625.41 |
| 1760.77 | 1926.17 |  | 1211.52 | 3734.13 |  | 3387.33 | 4302.12 |
| 2316.61 | 2028.35 |  | 11037.34 | 11194.03 |  | 2476.23 | 2296.89 |
| 8307.46 | 4965.86 |  | 7360.05 | 11559.43 |  | 1717.01 | 1625.12 |
| 2359.17 | 2160.53 |  | 8009.24 | 11010.55 |  | 5571.11 | 8189.19 |
| 4002.94 | 3102.93 |  | 11107.88 | 18873.21 |  | 1988.53 | 3719.13 |
| 3939.85 | 2461.64 |  | 12293.27 | 10409.8 |  | 2263.23 | 5033.04 |
| 3011.11 | 3000.21 |  | 3281.36 | 7040.81 |  | 2403.65 | 4290.37 |
| 4105.77 | 2605.9 |  | 8767.64 | 12470.96 |  | 4351.69 | 4368 |
| 3788.35 | 4498.89 |  | 7866.89 | 12275.02 |  | 6070.04 | 4525.59 |
| 2157.42 | 2621.65 |  | 13126.1 | 12014.62 |  | 1371.68 | 2481.3 |
| 3212.27 | 1820.29 |  | 8472.87 | 10318.95 |  | 6673.58 | 9468.61 |
| 1444.85 | 1194.06 |  | 10428.52 | 15445.83 |  | 3840.42 | 4529.09 |
| 2769.35 | 2153.47 |  | 4840.03 | 8417.39 |  | 7918.09 | 5119 |
| 2683.87 | 4422.42 |  | 4398.15 | 6663.13 |  | 13559.15 | 12590.67 |
| 2112.94 | 2378.87 |  | 9201.21 | 9817.69 |  | 4798.25 | 3822.45 |
| 2655.12 | 2612.07 |  | 9016.25 | 8046.98 |  | 6464.25 | 5417.35 |
| 2742.17 | 1641.45 |  | 8999.2 | 10578.8 |  | 4265.47 | 8130.1 |
| 2066.46 | 2113.81 |  | 5218.33 | 8539.06 |  | 9232.84 | 8388.96 |
| 3101.92 | 3944.58 |  | 5961.79 | 10267.02 |  | 3239.93 | 7748.58 |
| 2076.16 | 3778.27 |  | 2165.69 | 7449.19 |  | 8158.61 | 10723.34 |
| 11915.48 | 10438.04 |  | 9722.2 | 14241.7 |  | 4042.74 | 7066.12 |
| 5786.11 | 7051.37 |  | 8123.61 | 6778.08 |  | 7546.44 | 9733.12 |
| 2801.68 | 3993.48 |  | 3600.46 | 7136.25 |  | 3419.83 | 4577.41 |
| 2587.66 | 5170.38 |  | 5144.91 | 8839.02 |  | 7404.8 | 10197.84 |
| 8995.3 | 6425.52 |  | 7599.29 | 9079.61 |  | 5094.72 | 9313.2 |
| 4238.27 | 3686.23 |  | 13352.72 | 11749.01 |  | 6999.16 | 5694.98 |
| 5014.05 | 3242.24 |  | 8104.58 | 11442.57 |  | 9417.7 | 5305.72 |
| 2945.28 | 4556.46 |  | 8047.1 | 10884.48 |  | 9396.12 | 15588.97 |
| 2721.43 | 3706.59 |  | 8618.74 | 12824.83 |  | 3062.23 | 8158.77 |
| 5232.28 | 4234.7 |  | 8645.16 | 10271.41 |  | 4251.4 | 8278.62 |
| 3114.28 | 8005.73 |  | 6462.05 | 14771.03 |  | 4655.87 | 8306.51 |
| 7002.95 | 8291.68 |  | 9414.66 | 23142.8 |  | 3087.68 | 5942.28 |
| 5734.99 | 5380.58 |  | 9723.68 | 9074.59 |  | 4731.15 | 6180.53 |
| 7035.63 | 5983.98 |  | 10596.34 | 13538.76 |  | 5710.64 | 7156.8 |
| 8691.37 | 7802.01 |  | 9728.97 | 9542.15 |  | 17070.78 | 12070.32 |
| 4177.02 | 9140.93 |  | 10982.25 | 19737.16 |  | 7542.82 | 4900.44 |
| 3818.04 | 5580.17 |  | 8363.75 | 13528.83 |  | 1890.6 | 2678.85 |
| 2546.99 | 3296.96 |  | 16117.08 | 18808.7 |  | 6528.3 | 7267.78 |
| 2796.01 | 3563.17 |  | 8351.3 | 12216.76 |  | 11194.07 | 12059.92 |
| 3797.61 | 4721.02 |  | 7681.91 | 12794.51 |  | 4362.66 | 4846.6 |
| 2286.05 | 3259.1 |  | 2516.74 | 7939.89 |  | 6085.62 | 9243.37 |
| 4276.91 | 5484.7 |  | 6403.68 | 10714.57 |  | 5334.32 | 6312.1 |
| 1672.03 | 3458.62 |  | 13485.69 | 10529.49 |  | 4947.61 | 3472.29 |
| 2462.7 | 3671.59 |  | 11022.81 | 15301.8 |  | 5446.21 | 9976.72 |
| 1888.67 | 6230.68 |  | 8931.91 | 12912.75 |  | 7817.73 | 7163.06 |
| 2428.53 | 7521.08 |  | 6786.6 | 11415.97 |  | 10003.05 | 10758.09 |
| 5641 | 4624.95 |  | 7235.77 | 7711.41 |  | 7834.59 | 12307.16 |
| 3118.1 | 2049.05 |  | 7388.74 | 7754.69 |  | 6177.06 | 8166.22 |
| 2827.25 | 4205.92 |  | 13728.88 | 7803.61 |  | 6148.52 | 8225.69 |
| 3085.48 | 4290.66 |  | 4244.95 | 6772.88 |  | 7403.7 | 8074.01 |
| 1473.75 | 2832.49 |  | 5789.62 | 9102.15 |  | 8348.14 | 8245.8 |
| 1686.66 | 2640.43 |  | 20375.63 | 12954.59 |  | 5994.19 | 4703.79 |
| 4401.1 | 4760.31 |  | 2820.83 | 4752.03 |  | 8850.39 | 11278.71 |
| 5875.96 | 3402.97 |  | 5961.64 | 10640.54 |  | 3497.37 | 5059.97 |
| 2539.35 | 4513.6 |  | 4896.43 | 6231.86 |  | 8780.87 | 8457.91 |
| 2532.65 | 5240.03 |  | 4477.12 | 6429.66 |  | 3047.77 | 7285.83 |
| 4876.68 | 6108.97 |  | 3690.28 | 6273.87 |  | 10446.03 | 11881.51 |
| 14046.2 | 16429.95 |  | 5073.8 | 12152.18 |  | 5235.51 | 6637.32 |
| 7165.99 | 8297.3 |  | 2151.47 | 5295.73 |  | 6909.32 | 10254.67 |
| 8525.37 | 8550.45 |  | 6613.46 | 7109.58 |  | 5282.1 | 9282.76 |
| 5229.06 | 10805.88 |  | 1500 | 3474.1 |  | 4591.73 | 17088.51 |
| 10838.89 | 6005.48 |  | 7004.3 | 8287.69 |  | 5356.43 | 3421.97 |
| 6231.04 | 12081.62 |  | 6400 | 6434.16 |  | 6711.01 | 6278.84 |
| 8636.71 | 8874.34 |  | 2000 | 5328.45 |  | 6247.76 | 5584.81 |
| 7878.43 | 5063.74 |  | 1803.24 | 2963.73 |  | 5583.66 | 9875.44 |
| 4369.2 | 7257.13 |  | 2500 | 3000 |  | 4810.14 | 10957.44 |
| 6915.87 | 8715.17 |  | 4780.69 | 4780 |  | 7843.39 | 8292.04 |
| 12113.86 | 8153.91 |  | 5554.23 | 3234.25 |  | 3854.71 | 11189.79 |
| 4722.45 | 6017.84 |  | 3000 | 5003.62 |  | 3423.61 | 4990.07 |
| 5744.1 | 7653.56 |  | 1343.03 | 1663.97 |  | 5526.03 | 6563 |
| 11922.93 | 10104.22 |  | 1756.37 | 1106.5 |  | 5906.58 | 11147.09 |
| 7856.48 | 10443.46 |  | 2539.68 | 2382.5 |  | 5645.32 | 9583.25 |
| 5878.21 | 8150.22 |  | 820.08 | 2812.79 |  | 2953.67 | 4884.48 |
| 5780.95 | 5572.47 |  | 3764.72 | 7562.1 |  | 9366.77 | 11859.66 |
| 5733.18 | 5211.26 |  | 3758.14 | 12707.5 |  | 7443.58 | 10415.49 |
| 1100.07 | 3250.06 |  | 5893.74 | 5982.26 |  | 6159.97 | 4781.59 |
| 1824.17 | 5146.45 |  | 6149.79 | 4208.2 |  | 11130.75 | 12756.98 |
| 1351.26 | 2745.97 |  | 2785.74 | 7754.25 |  | 2899.46 | 3560.71 |
| 6183.88 | 3232.06 |  | 2802 | 6269.33 |  | 4699.27 | 6190.2 |
| 2688.27 | 3333.61 |  | 4031.53 | 6869.8 |  | 6197.14 | 10441.96 |
| 6212.12 | 4883.82 |  | 4553.53 | 7549.13 |  | 4817.8 | 6579.07 |
| 9732.66 | 8814.06 |  | 2070.45 | 4978.12 |  | 10454.75 | 14972.99 |
| 137.35 | 898.51 |  | 6812.02 | 886.13 |  | 2591.57 | 6585.73 |
| 485.12 | 902.37 |  | 5580.52 | 7788.3 |  | 7279.56 | 6339.66 |
| 775.15 | 1289.17 |  | 2482.32 | 7934.84 |  | 2821.86 | 6251.83 |
| 241.21 | 394.93 |  | 2529.26 | 4996.93 |  | 5175.58 | 9354.24 |
| 194.92 | 639.61 |  | 6194.74 | 8123.82 |  | 6032.29 | 7604.33 |
| 114.2 | 483.25 |  | 3500 | 4085.82 |  | 4293.46 | 6643.36 |
| 215.64 | 667.98 |  | 1313.28 | 4497.01 |  | 3782.55 | 14780.43 |
| 396.85 | 1013.15 |  | 67252.1 | 6293.06 |  | 6102.16 | 14286.15 |
| 251.4 | 651.1 |  | 4903.86 | 4634.8 |  | 4544.58 | 7284.33 |
| 296.09 | 469.14 |  | 8873.66 | 5600.16 |  | 2117.8 | 3481.55 |
| 364.22 | 451.18 |  | 5950.18 | 7606.89 |  | 6624.07 | 7140.44 |
| 521.08 | 956.59 |  | 20985.27 | 5809.67 |  | 6374.46 | 7252.54 |
| 2831.09 | 5137.11 |  | 15560.98 | 8273.231 |  | 6100.69 | 8432.68 |
| 2152.06 | 2386.39 |  | 5961.79 | 9180.15 |  | 4556.75 | 7796.45 |
| 4950 | 3862.57 |  | 2784.99 | 6636.17 |  | 3708.9 | 7178.03 |
| 2406.85 | 4666.72 |  | 4310.93 | 12105.8 |  | 4973.15 | 7525.98 |
| 2414.86 | 2998.34 |  | 1706.14 | 3760.61 |  | 4527.58 | 5930.27 |
| 1776 | 2424.79 |  | 2077.63 | 6395.71 |  | 3387.26 | 12150.51 |
| 1499.55 | 3544.14 |  | 1229.01 | 7678.77 |  | 4070.02 | 4762.9 |
| 3084.88 | 6525.08 |  | 4261.43 | 9157.42 |  | 3783.95 | 8060.28 |
| 2551.93 | 4950.08 |  | 1861.04 | 7248.76 |  | 5434.95 | 15701.48 |
| 1157.23 | 4234.72 |  | 3377.88 | 6585.15 |  | 5490.35 | 7673.67 |
| 2662.94 | 4721.42 |  | 12929.45 | 8564.36 |  | 4765.7 | 6132.95 |
| 4000 | 5357.29 |  | 5932.78 | 6646.15 |  | 3815.75 | 8700.26 |
| 4585.58 | 2869.67 |  | 7423.33 | 18156.05 |  | 2532.57 | 8060.25 |
| 2766.59 | 4361.56 |  | 4306.61 | 8658.19 |  | 8781.65 | 13890.13 |
| 3817.31 | 2131.79 |  | 8647.05 | 7949.54 |  | 2603.3 | 17649.23 |
| 3699.95 | 4830.26 |  | 6581.2 | 16731.5 |  | 3598.56 | 6949.82 |
| 1776.12 | 4365.34 |  | 18004.72 | 7640.28 |  | 9301.43 | 11860.06 |
| 3626.71 | 5888.97 |  | 7731.2 | 12611.84 |  | 7204.79 | 15377.24 |
| 2975.87 | 5481.62 |  | 11694.87 | 4741.55 |  | 8110.99 | 10959.07 |
| 3443.57 | 5173.38 |  | 6413.72 | 2645.4 |  | 9298.15 | 18475.92 |
| 2731.94 | 2485.28 |  | 5000 | 7000 |  | 14780.44 | 14956.98 |
| 7807.28 | 4680.22 |  | 2237.22 | 4513.62 |  | 6668.93 | 9749.03 |
| 6429.21 | 8396.97 |  | 4406.01 | 6771.95 |  | 8039.81 | 8460.231 |
| 3591.83 | 3533.65 |  | 15127.41 | 15255.19 |  | 3930.8 | 7494.11 |
| 2403.97 | 1875.41 |  | 1772.43 | 6743.7 |  | 7850.07 | 14345.88 |
| 1499.47 | 3095.01 |  | 13981.85 | 18640.15 |  | 3959.53 | 8736.43 |
| 3502.75 | 2786.16 |  | 6284.43 | 10432.95 |  | 4415.82 | 10615.49 |
| 9439.63 | 5655.06 |  | 10935.17 | 7185.2 |  | 8141.33 | 7910.04 |
| 3269.72 | 3473.61 |  | 5785.81 | 4878.14 |  | 5872.67 | 8970.35 |
| 3154.91 | 2535.44 |  | 12000 | 12758.99 |  | 5350.5 | 5983.2 |
| 1886.32 | 1421.16 |  | 1250.04 | 8633.481 |  | 10450.49 | 9946.3 |
| 2661.31 | 1486.17 |  | 10016.97 | 3108.84 |  | 5069.82 | 3863.31 |
| 1726.03 | 893.61 |  | 1216.22 | 4191.57 |  | 11613.14 | 14272.9 |
| 1239.38 | 4813.93 |  | 4556.04 | 6356.43 |  | 5846.53 | 12925.48 |
| 2515.32 | 2137.87 |  | 6137.93 | 8971.65 |  | 15994.21 | 19352.96 |
| 2207.28 | 3031.05 |  | 3725.57 | 6895.77 |  | 4278.31 | 6194.72 |
| 2819.94 | 2857.44 |  | 6747.15 | 5246.55 |  | 17089.96 | 22808.14 |
| 7424.92 | 7676.45 |  | 9776.94 | 11724.01 |  | 14535.67 | 14456.72 |
| 2222 | 2221.48 |  | 4455.2 | 7264.22 |  | 9066.6 | 16012.56 |
| 1388.37 | 4257.67 |  | 2574.16 | 3624.89 |  | 7191.06 | 4660.18 |
| 2307.04 | 4663.91 |  | 9293.1 | 6437.98 |  | 1788.68 | 4439.45 |
| 1494.74 | 2505.07 |  | 13765.04 | 11975.75 |  | 4021.47 | 9380.46 |
| 2186.97 | 5534.33 |  | 3709.85 | 4099.29 |  | 7956.63 | 8910.43 |
| 3456.99 | 4741.24 |  | 6625.68 | 10140.92 |  | 8734.38 | 13175.23 |
| 6409.73 | 8285.76 |  | 626.13 | 7906.57 |  | 4057.57 | 7706.79 |
| 5758.27 | 3107.4 |  | 4315.87 | 11842.69 |  | 15641.75 | 14667.22 |
| 4495.22 | 5446.86 |  | 4813.45 | 6129.85 |  | 11032.68 | 16972.46 |
| 6083.84 | 3884.59 |  | 6172.85 | 5745.44 |  | 5694.32 | 8679.56 |
| 3964.49 | 4478.65 |  | 3004.5 | 9528.2 |  | 5411.28 | 6733.87 |
| 3792.99 | 3386.04 |  | 2154.72 | 3417.53 |  | 10406.02 | 8110.02 |
| 3127.21 | 2395.95 |  | 17993.38 | 10702.98 |  | 8528.37 | 11109.39 |
| 2107.6 | 7405.05 |  | 9257.21 | 9312.54 |  | 10940.82 | 10526.27 |
| 7395.88 | 9944.29 |  | 9066.981 | 6714.62 |  | 4463.47 | 4822.11 |
| 3847.37 | 3070.41 |  | 4691.88 | 13230.74 |  | 8320.05 | 11104.78 |
| 1850.27 | 7090.22 |  | 1596.87 | 6822.05 |  | 8213.66 | 5966.05 |
| 9836.92 | 9613.61 |  | 4412.99 | 993.26 |  | 8178.76 | 13167.02 |
| 1261.7 | 4179.09 |  | 3155.01 | 5253.62 |  | 11633.96 | 15765.79 |
| 2938.98 | 4491.85 |  | 3296.22 | 11555.89 |  | 6388.86 | 8718.05 |
| 5430.98 | 6973.25 |  | 8760.72 | 112117.3 |  | 5219.17 | 6242.66 |
| 434.04 | 945.4 |  | 5556.1 | 8067.86 |  | 16278.15 | 10307.16 |
| 431.21 | 897.74 |  | 8460.79 | 13092.18 |  | 9566.58 | 19551.2 |
| 2115.26 | 6343.71 |  | 3638.22 | 15951.62 |  | 3708.69 | 7776.9 |
| 3181.35 | 4193.31 |  | 3211.62 | 13092.17 |  | 9306.38 | 5615.47 |
| 2205.13 | 3097.11 |  | 1928.11 | 4270.32 |  | 4589.41 | 15500.4 |
| 2000 | 3087.7 |  | 5533.53 | 7839.15 |  | 6531.88 | 10396.99 |
| 7630.83 | 6056.11 |  | 5396.41 | 6671.59 |  | 3151.9 | 9254.01 |
| 3952.73 | 5592.83 |  | 6421.01 | 4146.22 |  | 4342.76 | 5371.5 |
| 1577.91 | 2685.27 |  | 2876.78 | 7340.88 |  | 9396.981 | 17213.12 |
| 4453.58 | 3663.99 |  | 3860.81 | 4732.64 |  | 5776.82 | 11563.91 |
| 3500 | 4825.06 |  | 2744.57 | 5389.87 |  | 7493.98 | 12929.48 |
| 2911.21 | 3968.68 |  | 3477.53 | 7379.12 |  | 5094.6 | 10510.22 |
| 2500 | 4250 |  | 4010.13 | 7153.48 |  | 7795.02 | 14667.47 |
| 1563.54 | 3371.16 |  | 5038.35 | 11049.95 |  | 11173.14 | 19302.68 |
| 2300 | 3000 |  | 9789.95 | 12361.84 |  | 6102.13 | 18159.7 |
| 2408.76 | 3338.37 |  | 3849.44 | 7294.16 |  | 7238.78 | 9166.11 |
| 1077.23 | 2350.24 |  | 2904 | 6829.28 |  | 4384.31 | 9940.87 |
| 2900 | 2900 |  | 2592.95 | 4392.15 |  | 9240.64 | 9076.56 |
| 5000 | 4862.76 |  | 5698.71 | 3222.89 |  | 2923.74 | 3294.53 |
| 3790.64 | 6061.84 |  | 2799.09 | 8464.34 |  | 6384.63 | 3843.73 |
| 2448.58 | 4581.69 |  | 3561.83 | 11159.68 |  | 3362.23 | 9605.46 |
| 4365.71 | 2000 |  | 10118.59 | 7819.64 |  | 4774.85 | 7381.04 |
| 4058.06 | 1460.77 |  | 6645.65 | 7370.02 |  | 5397.24 | 7808.77 |
| 3500 | 3821.81 |  | 5737.63 | 7999.02 |  | 3359.81 | 13675.14 |
| 4363.25 | 5272.7 |  | 10696.74 | 11242.87 |  | 10460.61 | 14466.93 |
| 3800 | 4226.49 |  | 3136.55 | 8550.17 |  | 2840.75 | 8036.89 |
| 1482.42 | 6505.78 |  | 7719.82 | 6189.84 |  | 3309.55 | 6108.11 |
| 1887.34 | 3855.93 |  | 1855.52 | 8555.91 |  | 10242.43 | 11681.78 |
| 3919.21 | 6304.81 |  | 3927.53 | 8164.66 |  | 10815.22 | 13810.77 |
| 4788.16 | 10122.05 |  | 12667.48 | 8973.41 |  | 7165.02 | 11042.41 |
| 13000 | 7909.85 |  | 1797.97 | 6065.42 |  | 7091.62 | 10542.96 |
| 6148.69 | 6963.13 |  | 9521.731 | 5208 |  | 3581.73 | 8810.88 |
| 2710.07 | 10666.52 |  | 3481.3 | 10056.25 |  | 4094.71 | 5344.13 |
| 5100.13 | 6320.74 |  | 4135.74 | 5596.66 |  | 4045.74 | 5838.05 |
| 9490.64 | 6076.59 |  | 5688.4 | 5600 |  | 3997.95 | 6434.8 |
| 4849.62 | 3512.49 |  | 2992.94 | 9273.83 |  | 10894.78 | 19576.06 |
| 6421.78 | 3813.76 |  | 3363.62 | 4619.42 |  | 2857.27 | 7567.09 |
| 5431.55 | 6606.31 |  | 2820.12 | 6594.9 |  | 4034.16 | 7281.6 |
| 6172.41 | 4374.79 |  | 4562.68 | 9527.56 |  | 5170.24 | 9586.14 |
| 4063.39 | 4000 |  | 4121.59 | 1900.87 |  | 5881 | 10040.32 |
| 1268 | 4000 |  | 6718.11 | 5436.55 |  | 5404.12 | 7307.66 |
| 2173.46 | 3087.7 |  | 6511.93 | 7231.54 |  | 4886.56 | 12679.34 |
| 5989.61 | 5800 |  | 1541.4 | 5248.89 |  | 14136.38 | 20850.37 |
| 4328.8 | 3713.46 |  | 3361.8 | 2446.45 |  | 3203.31 | 9267.13 |
| 3072.21 | 1415.71 |  | 8090.58 | 10831.95 |  | 7340.16 | 16371.01 |
| 3692.32 | 3651.27 |  | 8207.19 | 6763.21 |  | 6152.25 | 11301.81 |
| 5036.1 | 2000 |  | 6156.54 | 6678.91 |  | 7553.38 | 9097.481 |
| 3221.63 | 3228.32 |  | 7753.71 | 9098.67 |  | 4753.85 | 8493.8 |
| 2758.73 | 4387.39 |  | 9209.981 | 4459.17 |  | 4338.43 | 6373.96 |
| 3825.92 | 2458.74 |  | 6396.53 | 3331.27 |  | 6638.11 | 9266.44 |
| 2332.31 | 4364.55 |  | 9700.13 | 7442.5 |  | 3984.75 | 6849.87 |
| 1696.36 | 1600 |  | 11679.43 | 7835.35 |  | 5642.05 | 9741.72 |
| 862.22 | 1611.54 |  | 4562.68 | 7967.52 |  | 5609.37 | 13100.78 |
| 3030.67 | 4858.73 |  | 4121.59 | 5424.27 |  | 5058.75 | 11811.36 |
| 3790.64 | 3000 |  | 6718.11 | 4131.43 |  | 12114.36 | 11095.86 |
| 3407.98 | 2788.51 |  | 6511.93 | 3675.27 |  | 10158.95 | 6230.16 |
| 2474.62 | 5892.12 |  | 1541.4 | 2433.11 |  | 4878.84 | 4525.62 |
| 2985.74 | 4670.47 |  | 3361.8 | 11195.39 |  | 6703.85 | 6410.72 |
| 4202.68 | 4759.73 |  | 8090.58 | 6152.54 |  | 14255.5 | 10316.58 |
| 4363.25 | 3271.92 |  | 8207.19 | 11722.99 |  | 6028.35 | 4718.45 |
| 5054.09 | 3700 |  | 6156.54 | 10724.49 |  | 8456.1 | 7580.85 |
| 3116.08 | 4378.94 |  | 7753.71 | 8863.01 |  | 11930.49 | 8048.76 |
| 3114.91 | 1851.7 |  | 9209.981 | 6519.06 |  | 4533.92 | 8819.99 |
| 3777.41 | 3515.97 |  | 6396.53 | 3672.29 |  | 12596.61 | 12173.34 |
| 6148.69 | 4657.62 |  | 9700.13 | 17969.6 |  | 7037.79 | 6332.45 |
| 10074.94 | 1000 |  | 11679.43 | 16640.45 |  | 8524.01 | 7473.8 |
| 6123.96 | 2805.02 |  | 3586.34 | 2134.32 |  | 7988.81 | 8252.83 |
| 9490.64 | 6076.59 |  | 4543.75 | 3601.22 |  | 12138.55 | 8358.99 |
| 6670.11 | 6245.42 |  | 4013.21 | 3530.96 |  | 19185.2 | 13381.44 |
| 5901.31 | 3813.76 |  | 1918.5 | 1414.39 |  | 7405.64 | 6240.35 |
| 5431.55 | 6229.99 |  | 1258.31 | 167.84 |  | 8339.55 | 6216.88 |
| 2950 | 3061.47 |  | 3690.84 | 2144.13 |  | 12186.79 | 9663.38 |
| 2845.23 | 4115.61 |  | 5233.51 | 8526.91 |  | 9972.75 | 11079.12 |
| 6895.56 | 6000 |  | 4145.21 | 4937.41 |  | 5474.89 | 4770.85 |
| 2658.92 | 2755.13 |  | 763.95 | 1000 |  | 14347.94 | 9208.96 |
| 6967.82 | 4400.16 |  | 3522.48 | 4528.71 |  | 5449.76 | 5498.01 |
| 2617.68 | 1794.99 |  | 7503.95 | 8654.08 |  | 5020.48 | 3299.85 |
| 3089.83 | 2255.31 |  | 5132.03 | 6344.55 |  | 6566.01 | 5732.14 |
| 4448.95 | 3090.37 |  | 3348.59 | 3988.56 |  | 6490.53 | 6833.59 |
| 2517.98 | 3739.6 |  | 2877.02 | 8761.78 |  | 4306.27 | 3075.75 |
| 4532.45 | 1822.71 |  | 3540.48 | 3962.74 |  | 7301.28 | 5940.39 |
| 686.5 | 1783.96 |  | 8440.07 | 4000 |  | 13924.39 | 10301.71 |
| 3478.28 | 3171.86 |  | 10552.62 | 18260.85 |  | 5747.91 | 8459.02 |
| 1143.07 | 2670.65 |  | 5976.87 | 5044.96 |  | 5590.68 | 8197.24 |
| 3515.17 | 5412.34 |  | 4162 | 7635.12 |  | 3109.33 | 3609.51 |
| 1511.9 | 3385.66 |  | 3884.75 | 5679.44 |  | 7791.7 | 4215.93 |
| 3353.3 | 1596.16 |  | 11998.23 | 4568.11 |  | 1800.38 | 2381.83 |
| 3450.24 | 6739.14 |  | 16566 | 16566.45 |  | 1543.63 | 6338.14 |
| 5023.15 | 3068.09 |  | 2998.9 | 8000 |  | 11781.41 | 4000 |
| 5519.11 | 4039.93 |  | 5445.68 | 5000 |  | 6956.95 | 5299.34 |
| 3302.84 | 3895.88 |  | 11865.57 | 19117.76 |  | 4598.41 | 2618.38 |
| 7193.72 | 7070.83 |  | 6151.57 | 6265.78 |  | 4182.21 | 3891.25 |
| 12187.22 | 5771.36 |  | 5171.36 | 9123.41 |  | 3268.97 | 4151.49 |
| 5255.04 | 8065.33 |  | 7000 | 4000 |  | 4705.5 | 3774.53 |
| 2754.87 | 2270.35 |  | 3968.33 | 13967.8 |  | 4257.09 | 3856.52 |
| 5219.14 | 3824.99 |  | 3000 | 7000 |  | 2352.71 | 6327.71 |
| 3737.64 | 3314 |  | 2343.18 | 5998.43 |  | 2531.88 | 3799.42 |
| 1833.05 | 2770.08 |  | 3616.14 | 1483.38 |  | 7457.18 | 4194.12 |
| 3831.9 | 5247.83 |  | 3519.46 | 6792.6 |  | 1378.43 | 3747.4 |
| 2950 | 3000 |  | 2000 | 1680.16 |  | 3310.47 | 2040.41 |
| 2845.23 | 5993.69 |  | 1554.2 | 1939.34 |  | 3217.27 | 4255.56 |
| 6895.56 | 7026.43 |  | 1320.52 | 2585.49 |  | 1544.65 | 3164.76 |
| 2658.92 | 3102.19 |  | 2227.95 | 3567.85 |  | 1320.33 | 4717.87 |
| 6967.82 | 3093.87 |  | 5466.09 | 2906.33 |  | 833.1 | 4055.67 |
| 2617.68 | 3716.74 |  | 3834.8 | 4715.37 |  | 1965.22 | 2139.2 |
| 3089.83 | 5524.78 |  | 2162.73 | 3342.09 |  | 2641.44 | 1092.82 |
| 4448.95 | 4420.39 |  | 4043.77 | 1896.41 |  | 5280.08 | 7969.74 |
| 2517.98 | 2224.27 |  | 4000 | 4884.65 |  | 3388.35 | 7230.28 |
| 4532.45 | 3205.75 |  | 3538.28 | 6259.74 |  | 11873.89 | 6382.08 |
| 686.5 | 3105.63 |  | 2014.09 | 5679.5 |  | 5299.76 | 9196.97 |
| 3478.28 | 4725.72 |  | 2000 | 5169.74 |  | 5827.98 | 6504.44 |
| 1143.07 | 2670.65 |  | 4500.55 | 7145.06 |  | 1561.49 | 5095.37 |
| 3515.17 | 5412.34 |  | 5986.39 | 5757.4 |  | 7000 | 5500 |
| 1511.9 | 3616.11 |  | 9412.55 | 9508.17 |  | 6227.14 | 10347.92 |
| 3353.3 | 3639.36 |  | 3545.3 | 4204.47 |  | 3966.22 | 6512.5 |
| 3450.24 | 6739.14 |  | 6067.67 | 8842.91 |  | 5614.22 | 8835.05 |
| 5023.15 | 1957.15 |  | 5208.31 | 7502.27 |  | 1641.87 | 3496.86 |
| 5519.11 | 6620.59 |  | 13980.37 | 8704.51 |  | 3658.41 | 15560.54 |
| 3302.84 | 5252.62 |  | 17976.27 | 16000 |  | 6274.65 | 8728.32 |
| 7193.72 | 3182.31 |  | 4411.78 | 8225.92 |  | 2286.93 | 1011.73 |
| 12187.22 | 8544.15 |  | 2160.84 | 7407.34 |  | 2732.97 | 11840.88 |
| 5255.04 | 4416.51 |  | 11000 | 15000 |  | 2441.63 | 4259.43 |
| 2754.87 | 5634.86 |  | 10127.61 | 18309.78 |  | 3964.5 | 6922.92 |
| 5219.14 | 4245.16 |  | 5683.57 | 8000 |  | 7076.35 | 10743.43 |
| 3737.64 | 6583.79 |  | 5710.72 | 9000 |  | 2519.73 | 1238.9 |
| 1833.05 | 3726.46 |  | 9091.49 | 9091 |  | 3231.26 | 11228.6 |
| 3831.9 | 5247.83 |  | 2937.08 | 8582.47 |  | 3729.01 | 7694.59 |
|  |  |  | 2670.62 | 5914.54 |  | 4274.58 | 3613.55 |
|  |  |  |  |  |  | 1414.75 | 5392.14 |
|  |  |  |  |  |  | 11168.58 | 8992.84 |
|  |  |  |  |  |  | 2473.32 | 4186.49 |
|  |  |  |  |  |  | 4809.17 | 2573.21 |
|  |  |  |  |  |  | 2532.06 | 6183.84 |
|  |  |  |  |  |  | 3739.12 | 3245.09 |
|  |  |  |  |  |  | 1676.38 | 4485.74 |
|  |  |  |  |  |  | 4659.73 | 3061.58 |
|  |  |  |  |  |  | 6055.39 | 5518.49 |
|  |  |  |  |  |  | 4185.31 | 12497 |
|  |  |  |  |  |  | 6202.88 | 6200 |
|  |  |  |  |  |  | 5475.56 | 1596.87 |
|  |  |  |  |  |  | 2840.17 | 3021.44 |
|  |  |  |  |  |  | 2804.4 | 9386.8 |
|  |  |  |  |  |  | 2160.89 | 9387.88 |
|  |  |  |  |  |  | 3684.8 | 5114.86 |
|  |  |  |  |  |  | 2612.39 | 7299.28 |
|  |  |  |  |  |  | 5025.24 | 13036.78 |
|  |  |  |  |  |  | 6791 | 8697.9 |
|  |  |  |  |  |  | 11125.19 | 15012.21 |
|  |  |  |  |  |  | 9647.61 | 6590.1 |
|  |  |  |  |  |  | 6000 | 8000 |
|  |  |  |  |  |  | 4553.52 | 3971.02 |
|  |  |  |  |  |  | 13319.42 | 15060.17 |
|  |  |  |  |  |  | 8398.54 | 13958.08 |
|  |  |  |  |  |  | 7422.87 | 5037.6 |
|  |  |  |  |  |  | 7356.85 | 2545.9 |
|  |  |  |  |  |  | 5949.62 | 7588.64 |
|  |  |  |  |  |  | 3930.44 | 6709.08 |
|  |  |  |  |  |  | 5912.34 | 9647.68 |
|  |  |  |  |  |  | 1523.47 | 10343.47 |
|  |  |  |  |  |  | 4979.24 | 7592.51 |
|  |  |  |  |  |  | 7455.35 | 2763.65 |
|  |  |  |  |  |  | 898.86 | 6178.11 |
|  |  |  |  |  |  | 1760.14 | 2329.72 |
|  |  |  |  |  |  | 2407.43 | 11649.51 |
|  |  |  |  |  |  | 9678.16 | 13904.61 |
|  |  |  |  |  |  | 4303.57 | 6618.72 |
|  |  |  |  |  |  | 2873.06 | 10991.27 |
|  |  |  |  |  |  | 6812.21 | 10791.98 |
|  |  |  |  |  |  | 3000 | 4392.59 |
|  |  |  |  |  |  | 4000 | 10072.25 |
|  |  |  |  |  |  | 3805.13 | 6483.16 |
|  |  |  |  |  |  | 5540.14 | 15004.52 |
|  |  |  |  |  |  | 15520.37 | 18744.37 |
|  |  |  |  |  |  | 4817.56 | 7780.38 |
|  |  |  |  |  |  | 3787.81 | 7761.94 |
|  |  |  |  |  |  | 4075.85 | 6933.84 |
|  |  |  |  |  |  | 2950.3 | 4183.74 |
|  |  |  |  |  |  | 8757.07 | 11499.37 |
|  |  |  |  |  |  | 2662 | 7816.64 |
|  |  |  |  |  |  | 8407.231 | 11593.8 |
|  |  |  |  |  |  | 4746.3 | 11308.95 |
|  |  |  |  |  |  | 2001.67 | 8967.61 |
|  |  |  |  |  |  | 6773.97 | 3529.73 |
|  |  |  |  |  |  | 6597.85 | 5898.88 |
|  |  |  |  |  |  | 16363.87 | 3974.31 |
|  |  |  |  |  |  | 3604.82 | 18488.02 |
|  |  |  |  |  |  | 6370.99 | 13064.39 |
|  |  |  |  |  |  | 5939.8 | 9993.24 |
|  |  |  |  |  |  | 4245.04 | 6759.4 |
|  |  |  |  |  |  | 3367.69 | 8134.04 |
|  |  |  |  |  |  | 8989.03 | 17122.83 |
|  |  |  |  |  |  | 8045.07 | 2508.13 |
|  |  |  |  |  |  | 3815.28 | 1042.71 |
|  |  |  |  |  |  | 2816.41 | 5587.44 |
|  |  |  |  |  |  | 3757.34 | 13799.8 |
|  |  |  |  |  |  | 2878.98 | 4055.94 |
|  |  |  |  |  |  | 3990.46 | 9557.3 |
|  |  |  |  |  |  | 6020.54 | 15782.97 |
|  |  |  |  |  |  | 7704.36 | 3733.71 |
|  |  |  |  |  |  | 6006.32 | 4412.85 |
|  |  |  |  |  |  | 6007.67 | 3486.09 |
|  |  |  |  |  |  | 4375.41 | 8742.44 |
|  |  |  |  |  |  | 11956.32 | 4808.71 |
|  |  |  |  |  |  | 8426.481 | 8301.07 |
|  |  |  |  |  |  | 3456.13 | 3726.09 |
|  |  |  |  |  |  | 11449.32 | 7106.89 |
|  |  |  |  |  |  | 9920.7 | 5679.78 |
|  |  |  |  |  |  | 14818.69 | 6926.53 |
|  |  |  |  |  |  | 6405.01 | 7768.63 |
|  |  |  |  |  |  | 6296.33 | 4178.33 |
|  |  |  |  |  |  | 4590.34 | 6702.69 |
|  |  |  |  |  |  | 4276.5 | 7690.34 |
|  |  |  |  |  |  | 2000 | 2799.83 |
|  |  |  |  |  |  | 2002.82 | 2212.93 |
|  |  |  |  |  |  | 1117.36 | 3830.53 |
|  |  |  |  |  |  | 4441.56 | 9527.47 |
|  |  |  |  |  |  | 3200 | 3434.49 |
|  |  |  |  |  |  | 7569.78 | 6878.16 |
|  |  |  |  |  |  | 7392.51 | 6090.82 |
|  |  |  |  |  |  | 4800 | 4000 |
|  |  |  |  |  |  | 2345.39 | 2866.22 |
|  |  |  |  |  |  | 1726.11 | 3453.37 |
|  |  |  |  |  |  | 3000 | 8107.31 |
|  |  |  |  |  |  | 6000 | 9083.15 |
|  |  |  |  |  |  | 9456.96 | 16082.76 |
|  |  |  |  |  |  | 4767.6 | 4344.94 |
|  |  |  |  |  |  | 16926.35 | 14914.92 |
|  |  |  |  |  |  | 4810.71 | 10406.47 |
|  |  |  |  |  |  | 2919.18 | 8000 |
|  |  |  |  |  |  | 4481.39 | 8932.06 |
|  |  |  |  |  |  | 15000 | 15714.94 |
|  |  |  |  |  |  | 4819.69 | 10588.8 |
|  |  |  |  |  |  | 2362 | 2300 |
|  |  |  |  |  |  | 4113.97 | 4664.25 |
|  |  |  |  |  |  | 5488.47 | 4539.25 |
|  |  |  |  |  |  | 7858.73 | 4563.8 |
|  |  |  |  |  |  | 5500 | 4435.16 |
|  |  |  |  |  |  | 7287.81 | 3800 |
|  |  |  |  |  |  | 11000 | 10839.66 |
|  |  |  |  |  |  | 6817.34 | 7000 |
|  |  |  |  |  |  | 8858.94 | 8119.65 |
|  |  |  |  |  |  | 4666.41 | 6842.98 |
|  |  |  |  |  |  | 12000 | 6000 |
|  |  |  |  |  |  | 5107.75 | 3423.39 |
|  |  |  |  |  |  | 7000 | 7000 |
|  |  |  |  |  |  | 7700.19 | 7423.83 |
|  |  |  |  |  |  | 2235.3 | 2044.51 |
|  |  |  |  |  |  | 1472.48 | 4089.29 |
|  |  |  |  |  |  | 2462.15 | 3393.57 |
|  |  |  |  |  |  | 9671.74 | 5820.08 |
|  |  |  |  |  |  | 3961.86 | 3200 |
|  |  |  |  |  |  | 5595.33 | 6735.1 |
|  |  |  |  |  |  | 4466.38 | 3184.56 |
|  |  |  |  |  |  | 4800 | 4842.42 |
|  |  |  |  |  |  | 5477.73 | 2191.22 |
|  |  |  |  |  |  | 4961.64 | 6714.59 |
|  |  |  |  |  |  | 5238.41 | 6037.81 |
|  |  |  |  |  |  | 6196.14 | 12559.32 |
|  |  |  |  |  |  | 3538.81 | 10414.53 |
|  |  |  |  |  |  | 6802.91 | 7917.06 |
|  |  |  |  |  |  | 4000 | 11000 |
|  |  |  |  |  |  | 4000 | 4000 |
|  |  |  |  |  |  | 3051.16 | 8278.91 |
|  |  |  |  |  |  | 4400 | 8178.44 |
|  |  |  |  |  |  | 10953.38 | 11694.84 |
|  |  |  |  |  |  | 6874.9 | 6500 |
|  |  |  |  |  |  | 3381.91 | 6500.47 |
|  |  |  |  |  |  | 9111.78 | 9420.35 |
|  |  |  |  |  |  | 10620.81 | 9524.65 |
|  |  |  |  |  |  | 3200.37 | 5172.74 |
|  |  |  |  |  |  | 10480.41 | 7828.47 |
|  |  |  |  |  |  | 12480.11 | 4040.28 |
|  |  |  |  |  |  | 10000 | 5000 |
|  |  |  |  |  |  | 11873.99 | 8680.02 |
|  |  |  |  |  |  | 8111.14 | 5572.09 |
|  |  |  |  |  |  | 7593.02 | 10719.16 |
|  |  |  |  |  |  | 12746.57 | 7869.72 |
|  |  |  |  |  |  | 4500 | 4000 |
|  |  |  |  |  |  | 7000 | 7000 |
|  |  |  |  |  |  | 3562.75 | 3370.07 |
