# Supplementary material for: Uncoupling of dynamin polymerization and GTPase activity revealed by the conformation-specific nanobody dynab
Source: eLife. 2017 Oct 12;6:e25197. doi: 10.7554/eLife.25197 (PMC5658065; doi:10.7554/eLife.25197)

**Figure 5 panel C**

Transferrin internalization data

| **dyn1** | **dyn1+dynab** | **dyn2** | **dyn2+dynab** | **dynab** | **NT** |
| --- | --- | --- | --- | --- | --- |
| 14571 | 9209 | 15074 | 9591 | 7472 | 18231 |
| 7766 | 10281 | 11770 | 7561 | 5260 | 17421 |
| 9452 | 13073 | 13262 | 5064 | 4261 | 19943 |
| 15126 | 18499 | 20799 | 7462 | 10902 | 20197 |
| 8919 | 13855 | 22706 | 5015 | 5387 | 25381 |
| 11615 | 9067 | 11478 | 5443 | 5356 | 25185 |
| 9592 | 10874 | 18742 | 4318 | 8753 | 12185 |
| 13267 | 13277 | 17075 | 7576 | 9010 | 24731 |
| 16474 | 10511 | 17197 | 8371 | 9152 | 19000 |
| 11396 | 6624 | 26083 | 7557 |  | 20612 |
| 18860 | 7345 | 11378 | 8862 |  | 17512 |
| 9084 | 13772 | 14586 | 10242 |  | 26388 |
| 8159 | 14423 | 12936 | 15596 |  | 27551 |
| 9317 | 12049 | 16789 | 12898 |  |  |
| 8278 | 15792 | 14613 | 14100 |  |  |
| 11563 | 22913 | 15187 | 11623 |  |  |
| 8057 | 18257 | 19186 | 16984 |  |  |
|  | 18539 | 22398 | 12772 |  |  |
|  | 15944 | 20874 | 12957 |  |  |
|  | 14770 | 17841 | 10589 |  |  |
|  | 17235 | 21090 | 11891 |  |  |
|  | 13812 |  | 13145 |  |  |
|  | 15926 |  | 8225 |  |  |
|  |  |  | 19158 |  |  |
|  |  |  | 24143 |  |  |
|  |  |  | 19443 |  |  |

**Statistical report**:


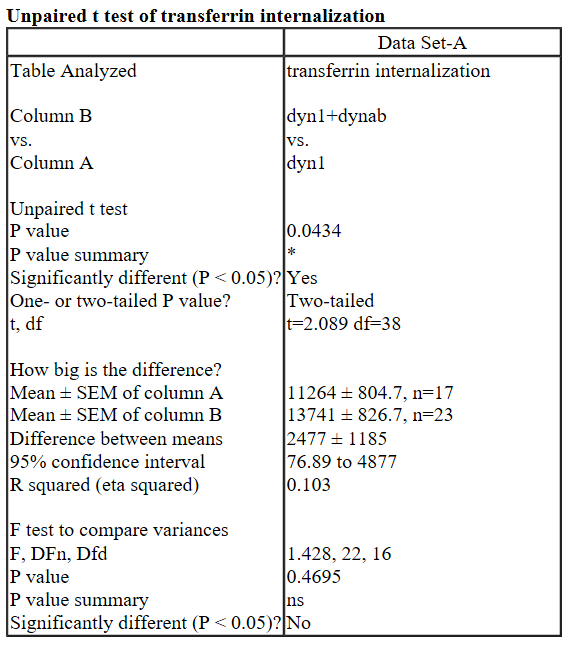


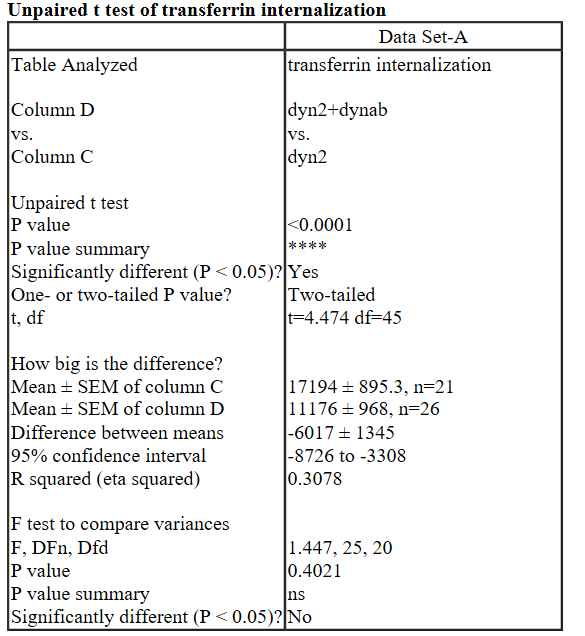


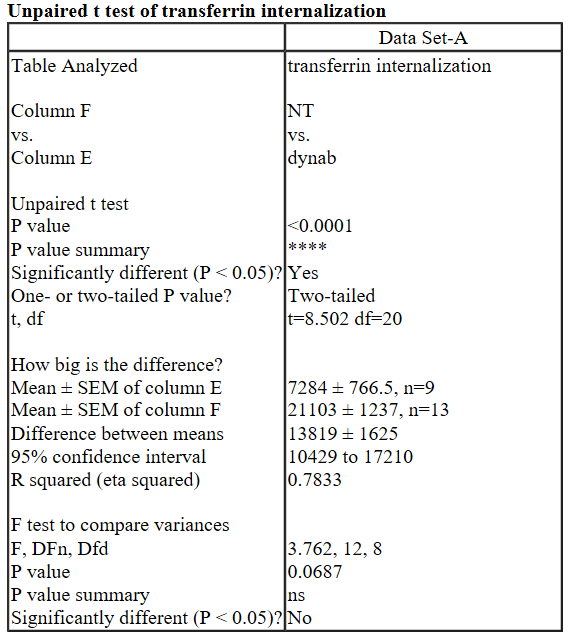


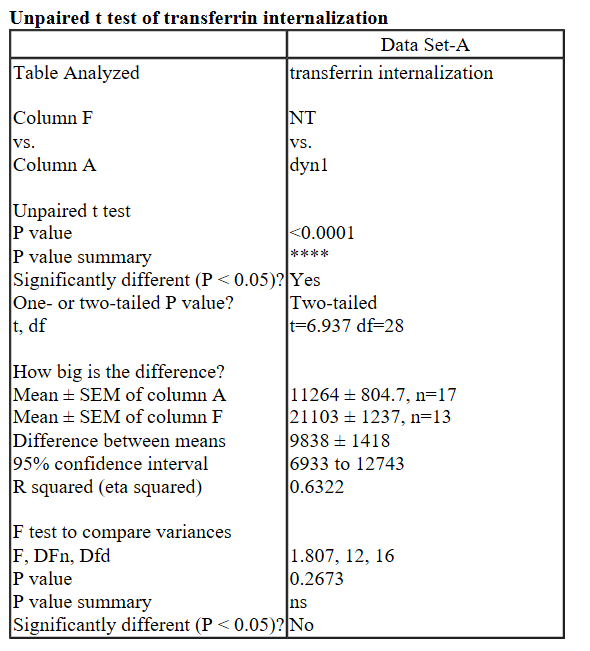


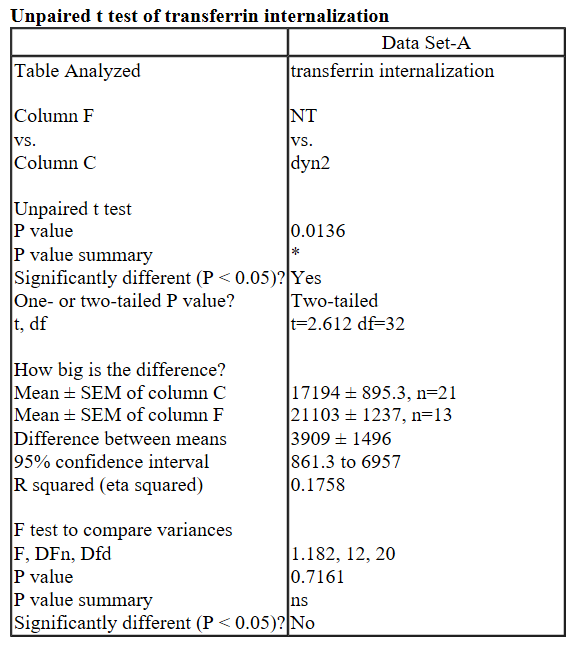


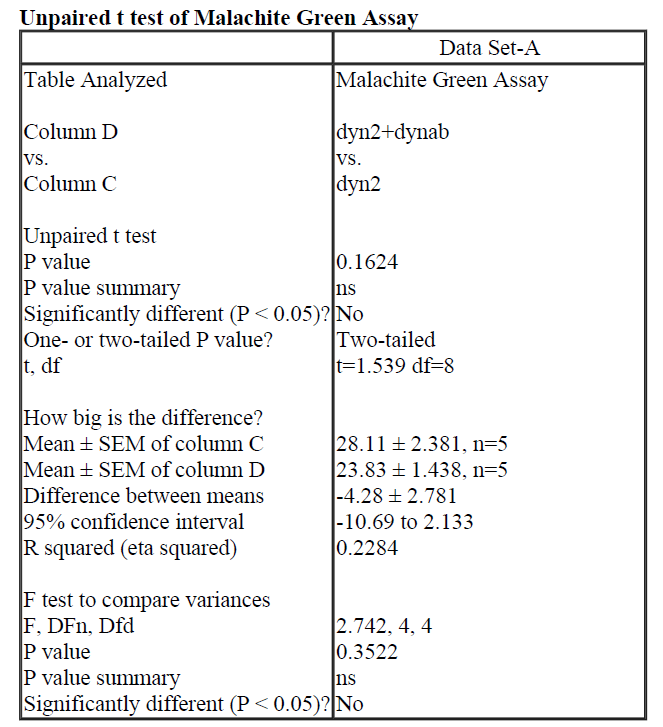

Supplement: Figure 5—source data 1. [file elife-25197-fig5-data1.docx]
