# Supplementary material for: Uncoupling of dynamin polymerization and GTPase activity revealed by the conformation-specific nanobody dynab
Source: eLife. 2017 Oct 12;6:e25197. doi: 10.7554/eLife.25197 (PMC5658065; doi:10.7554/eLife.25197)

**Figure 6 panel C**

EGF internalization data

| dyn1 | dyn1+dynab | dyn2 | dyn2+dynab | dynab | NT |
| --- | --- | --- | --- | --- | --- |
| 6233 | 5040 | 3441 | 10411 | 5311 | 8098 |
| 7257 | 4455 | 5665 | 7663 | 6915 | 10323 |
| 6066 | 4823 | 6967 | 6922 | 9356 | 10390 |
| 5466 | 5023 | 4542 | 7225 | 9357 | 8920 |
| 5314 | 4326 | 3870 | 6890 | 8621 | 8774 |
| 5238 | 5702 | 4079 | 10748 | 4981 | 11520 |
| 8348 | 5928 | 4261 | 6513 | 4713 | 5295 |
| 6989 | 7529 | 4622 | 7918 | 4997 | 7857 |
| 9346 | 6558 | 3981 | 8631 | 6954 | 9578 |
| 6441 | 5139 | 4521 | 6005 | 6587 | 9046 |
| 6033 | 5184 | 5058 | 5527 | 5551 | 7155 |
| 6995 | 6835 | 6092 | 6807 | 8657 | 8516 |
| 7223 | 6393 | 6465 | 5160 | 6924 | 10271 |
| 6170 | 5559 | 7647 | 6724 | 5588 | 5826 |
| 6055 | 5494 | 4721 | 5487 | 5109 | 4411 |
| 6957 | 4709 | 6006 | 5741 | 6915 | 7837 |
| 6314 | 9008 | 9236 | 5188 | 9352 | 5791 |
| 6143 | 6851 | 5639 | 5221 | 4881 | 5949 |
| 6363 | 5218 | 6660 | 5236 | 5548 | 7056 |
| 6809 | 5359 | 5754 | 5548 | 9580 | 8680 |
| 6853 | 5363 | 4821 | 5706 | 11348 | 8668 |
| 10036 | 6464 | 6796 | 7291 | 4994 | 7664 |
| 7122 | 7194 | 5532 | 8016 | 5239 | 7929 |
| 9794 | 9211 | 5282 | 8095 | 5078 | 8594 |
| 5367 | 7221 |  | 6281 | 6097 | 9652 |
| 6490 | 6059 |  | 5713 | 8548 |  |
| 7028 | 7496 |  | 7352 | 9650 |  |
| 7707 | 10014 |  | 6490 | 8680 |  |
| 8564 |  |  | 4594 | 8234 |  |

**Statistical report**:


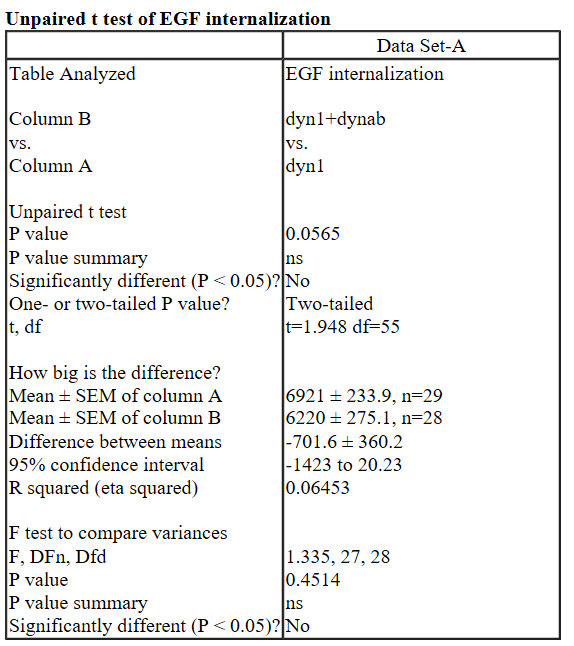


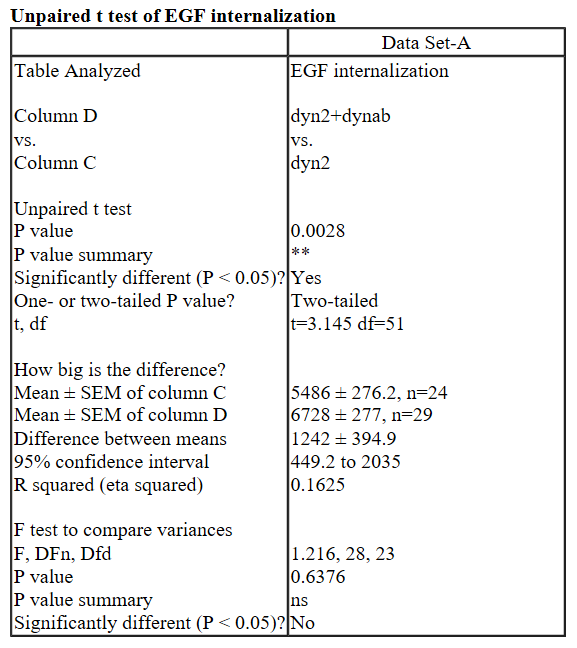


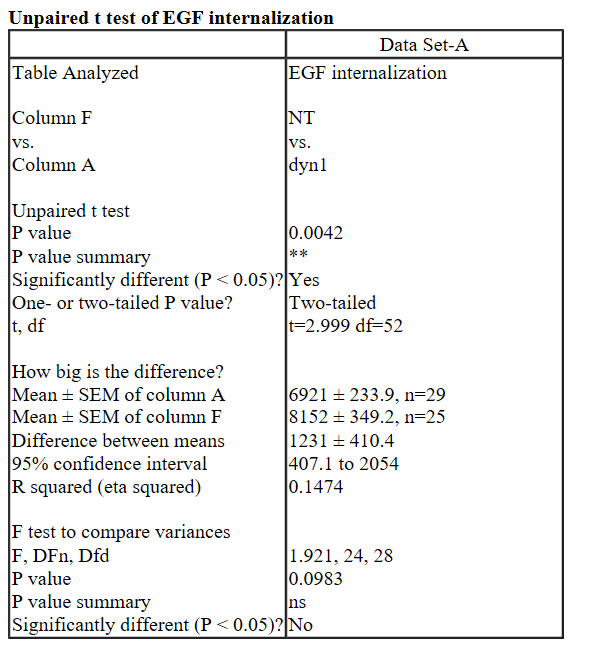


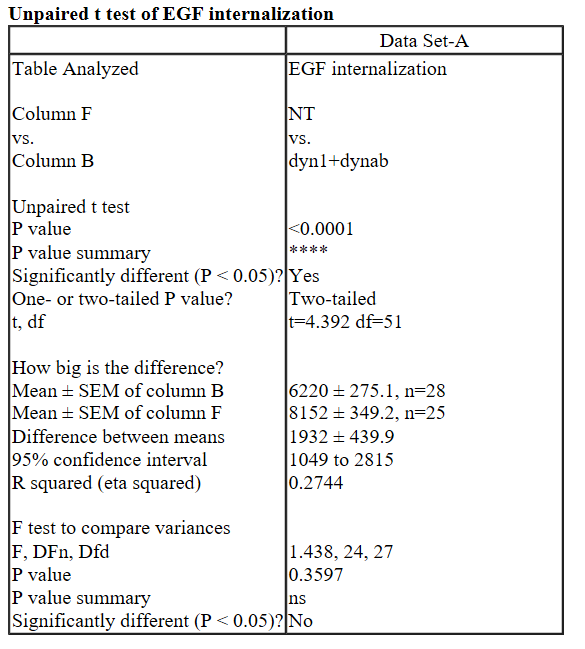


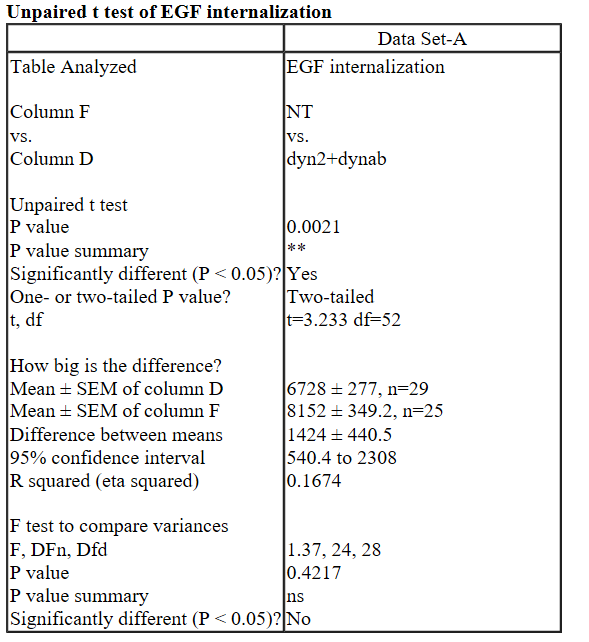


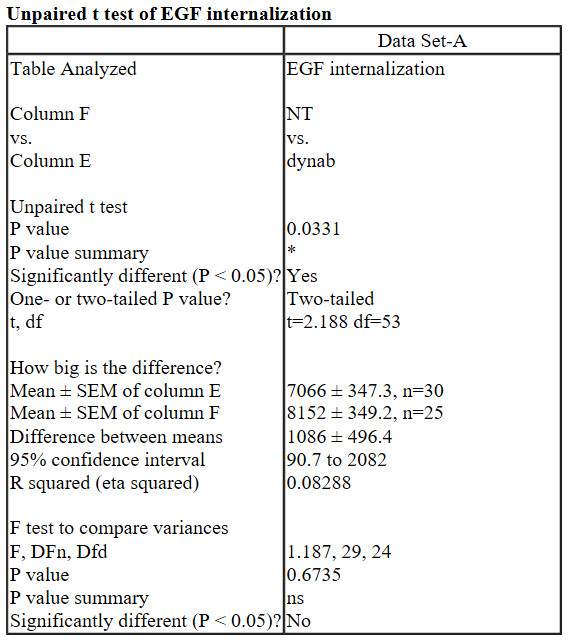

Supplement: Figure 6—source data 1. [file elife-25197-fig6-data1.docx]
