# Supplementary material for: Uncoupling of dynamin polymerization and GTPase activity revealed by the conformation-specific nanobody dynab
Source: eLife. 2017 Oct 12;6:e25197. doi: 10.7554/eLife.25197 (PMC5658065; doi:10.7554/eLife.25197)

AUTOMATIC EVENT ANALYSIS

PostUTrack_1

General purpose

PostuTrack is a Matlab code that post-processes the output from the software uTrack in order to obtain "events" associated to proteins marked and visualized using confocal light microscopy.

Overview of the algorithm in *postuTrack.m*

The code start from the detection of "bright spots" performed by uTrack at each frame of an image sequence.

We define EVENTS, as a set of consecutive frames where a bright spot is observed in the same location.

---

1) **INPUT**: The .tiff stack image files of both channels (A3 and Dynamin) and the two .mat files
2) Each spot detected is a candidate event. When a spot is processed several actions might be taken:
   a. The spot has spatial correspondence with event N, i.e, there was a spot detected in the same location in previous frames, and therefore this spot is part of a sequence. Then it is added to the event N.
      REMARK1: The spot is associated to a given event if it was present in previous frames, and there is not a temporal gap (where spot was not detected) larger than a user defined threshold (***MaxMissFrAllowed***, with default value 2 frames)
      REMARK2: The spot is considered to be "in the same location" as previous spots, if it has moved laterally between two consecutive frames less than a user defined threshold (***MaxLatDispl***, with default value 10 pixels)
   b. The spot has no spatial correspondence with close events (in space and time) and therefore is considered as a new potential event to which new spots in coming frames can be joined.
3) After obtaining the events formed by series of spots that where in the same location, and visible, an additional temporal check in minimum duration is performed. Those events that last less than a user-defined threshold are discarded (***MinDuration***, with default value 10 frames).
4) Surviving events are checked, to avoid events with very low peak intensity. Therefore, those events with a maximum peak intensity lower than a % of the maximum intensity observed in the sequence (with background subtracted), are considered as noise. A user-defined threshold controls the % (***Ithres***, with default value 40%).
5) Surviving events that pass all the thresholds described above are checked again to make sure that there is correlation between A3 and Dynamin channels, and that the A3 profile shows a Gaussian shape.

Summary of algorithm Thresholds:

**Name**	**Default Value**	**Description**
**ProfRadius**	2 px	Radius to calculate the average of the spot intensity and obtain gray-level profiles
**MaxLatDispl**	5 px	Maximum displacement of the maximum intensity pixel of an event allowed between frames

Summary of user critical Thresholds:

**Name**	**Default Value**	**Description**
**MinDuration**	10 frames<br>(has to be >= 6)	Minimum duration of an event allowed
**MaxMissFrAllowed**	2 frames	Maximum number of consecutive frames of an event where the spot is not detected
**Ithres**	0.40	The maximum intensity of a candidate profile has be larger than Ithres * the maximum intensity of the highest profile in the sequence
**CorrThres**	0.45	Minimum correlation between Dynamin and A3 channel
**FitThres**	0.60	Minimum goodness-of-fit of the A3 profile with a mixture Gaussian function

CLASSIFICATION OF EVENTS

Type	Thresholds passed	SAVED
1	**MaxLatDispl**<br>**MinDuration**<br>**MaxMissFrAllowed**<br>**Ithres**	Yes
2	**MaxLatDispl**<br>**MinDuration**<br>**MaxMissFrAllowed**<br>**Ithres**<br>**CorrThres**	Yes
3	**MaxLatDispl**<br>**MinDuration**<br>**MaxMissFrAllowed**<br>**Ithres**<br>**CorrThres**<br>**FitThres**	Yes

All the events are classified and save in a Matlab (.mat file), for subsequent analysis of the profiles. A report is created with the name (ReportDetection.txt). The report summarizes the thresholds used in the analysis and the number of events that was discarded due to each one of them.

NOTE: We are using a Gaussian fitting to a double mixture-Gaussian function. To fit two Gaussians we need at least 3 data points for each of them, which results in a minimum of 6 data points. Therefore the minimum duration of an event (**MinDuration**) has to be 6.

postUtrack_2_GenerateProfiles

General purpose

GenerateProfiles is a Matlab code that reads the output of postuTrack and produces the gray-intensity profiles of A3 and Dynamin events detected. Profiles are saved in image fails for a final user validation.

1) The code asks which type of events (see Type in table above).
2) Process the profile
    a. Open the intensity profiles for both channels
    b. Fits a double mixture-Gaussian to each channels and stores the parameters
    c. For each channel, obtains the maximum for the peak of each Gaussian and calculates the temporal delay between both.
    d. Plots the original profiles (dashed lines), the fitted profiles (solid line) and the times of the Peak maximum (vertical lines).
    e. Saves each figure as a .jpg file.

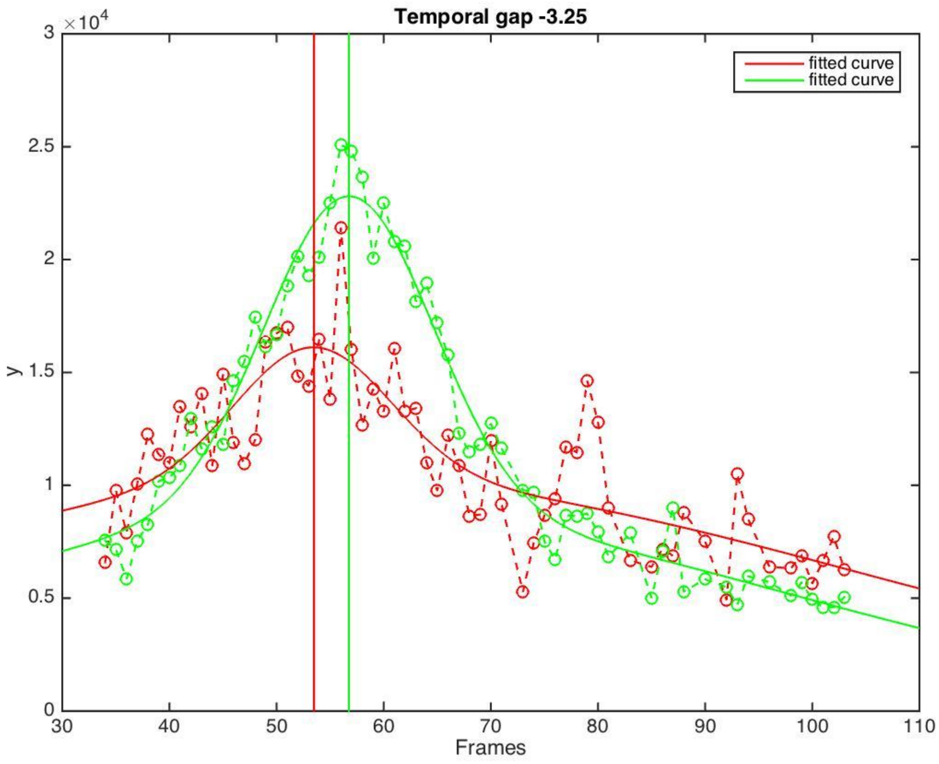

Supplement: Supplementary file 1. [file elife-25197-supp1.pdf]
