# Supplementary material for: Uncoupling of dynamin polymerization and GTPase activity revealed by the conformation-specific nanobody dynab
Source: eLife. 2017 Oct 12;6:e25197. doi: 10.7554/eLife.25197 (PMC5658065; doi:10.7554/eLife.25197)
Supplement: Transparent reporting form [file elife-25197-transrepform.pdf]

eLIFE
1st Floor
24 Hills Road
Cambridge CB2 1JP, UK
P 01223 855340
W elifesciences.org
T @elife

eLife's transparent reporting form

We encourage authors to provide detailed information *within their submission* to facilitate the interpretation and replication of experiments. If you have any questions, please contact us: editorial@elifesciences.org.

Sample-size estimation

- You should state whether an appropriate sample size was computed when the study was being designed

There was no computer-based estimation of sample size.

- You should state the statistical method of sample size computation and any required assumptions

There was no statistical method involved in determining the sample size.

- If no explicit power analysis was used, you should describe how you decided what sample (replicate) size (number) to use

We used very large numbers (above 100 events analysed, for more than 5 independent cells).

Please outline where this information can be found within the submission (e.g., page numbers or figure legends), or explain why this information doesn't apply to your submission:

See for each point below

Replicates

- You should report how often each experiment was performed

Details for each experiments, where applicable, are reported in the material and methods section (pages 22 to 29).

- You should include a definition of biological versus technical replication

Biological replicates are made with independent samples made on different days. Technical replicates are duplications of experiment done the same day with the same sample.

- The data obtained should be provided and sufficient information should be provided to indicate the number of independent biological and/or technical replicates

Details for each experiment, where applicable, are reported in the material and methods section (pages 22 to 29) and/or figure legends (Fig.4).

- If you encountered any outliers, you should describe how these were handled

The outliers came from errors in the fitting, thus they were excluded from the graphs.

- Criteria for exclusion/inclusion of data should be clearly stated

Details in the material and methods section (pages 22 to 29).

- High-throughput sequence data should be uploaded before submission, with a private link for reviewers provided (these are available from both GEO and ArrayExpress)

No high-throughput sequence data was used.

Please outline where this information can be found within the submission (e.g., page numbers or figure legends), or explain why this information doesn't apply to your submission:

Statistical reporting

- Statistical analysis methods should be described and justified

Data and statistical analysis is described in the text (pag.10-12; 28-29) and statistical reports are available in the source data files and in Fig. 1 and 4.

- Raw data should be presented in figures whenever informative to do so (typically when N per group is less than 10)
- For each experiment, you should identify the statistical tests used, exact values of N, definitions of center, methods of multiple test correction, and dispersion and precision measures (e.g., mean, median, SD, SEM, confidence intervals; and, for the major substantive results, a measure of effect size (e.g., Pearson's r, Cohen's d)

Full results of statistical analysis available in available in source data files and part of it is in Fig 1 and 4 legends.

- Report exact p-values wherever possible alongside the summary statistics and 95% confidence intervals. These should be reported for all key questions and not only when the p-value is less than 0.05.

p-values are reported, where necessary, in Fig.1 and 4, full results of statistical analysis are available in in source data files.

Please outline where this information can be found within the submission (e.g., page numbers or figure legends), or explain why this information doesn't apply to your submission:

(For large datasets, or papers with a very large number of statistical tests, you may upload a single table file with tests, Ns, etc., with reference to page numbers in the manuscript.)

eLIFE
1st Floor
24 Hills Road
Cambridge CB2 1JP, UK
P 01223 855340
W elifesciences.org
T @elife

Additional data files ("source data")

- We encourage you to upload relevant additional data files, such as numerical data that are represented as a graph in a figure, or as a summary table

Source data files for graphs is provided

- Where provided, these should be in the most useful format, and they can be uploaded as "Source data" files linked to a main figure or table

Source data files for graphs is provided

- Include model definition files including the full list of parameters used

NA

Include code used for data analysis (e.g., R, MatLab)

Matlab code is available in the submission files and will be uploaded in open source archive

- Avoid stating that data files are "available upon request"

Please indicate the figures or tables for which source data files have been provided:

eLife Sciences Publications, Ltd is a limited liability non-profit non-stock corporation incorporated in the State of Delaware, USA, with company number 5030732, and is registered in the UK with company number FC030576 and branch number BR015634 at the address 1st Floor, 24 Hills Road, Cambridge CB2 1JP | August 2014
